# Quelling the Geometry Factor Effect in Quantum Chemical Calculations of ^13^C NMR Chemical Shifts with the Aid of the pecG-*n* (*n* = 1, 2) Basis Sets

**DOI:** 10.3390/ijms251910588

**Published:** 2024-10-01

**Authors:** Yuriy Yu. Rusakov, Valentin A. Semenov, Irina L. Rusakova

**Affiliations:** A. E. Favorsky Irkutsk Institute of Chemistry, Siberian Branch of the Russian Academy of Sciences, Favorsky St. 1, 664033 Irkutsk, Russia; rusakov82@mail.ru (Y.Y.R.);

**Keywords:** pecG-1, pecG-2, PEC, equilibrium geometry, ^13^C NMR, chemical shift, shielding constant, DFT, coupled clusters, natural products

## Abstract

A root factor for the accuracy of all quantum chemical calculations of nuclear magnetic resonance (NMR) chemical shifts is the quality of the molecular equilibrium geometry used. In turn, this quality depends largely on the basis set employed at the geometry optimization stage. This parameter represents the main subject of the present study, which is a continuation of our recent work, where new pecG-*n* (*n* = 1, 2) basis sets for the geometry optimization were introduced. A goal of this study was to compare the performance of our geometry-oriented pecG-*n* (*n* = 1, 2) basis sets against the other basis sets in massive calculations of ^13^C NMR shielding constants/chemical shifts in terms of their efficacy in reducing geometry factor errors. The testing was carried out with both large-sized biologically active natural products and medium-sized compounds with complicated electronic structures. The former were treated using the computation protocol based on the density functional theory (DFT) and considered in the theoretical benchmarking, while the latter were treated using the computational scheme based on the upper-hierarchy coupled cluster (CC) methods and were used in the practical benchmarking involving the comparison with experimental NMR data. Both the theoretical and practical analyses showed that the pecG-1 and pecG-2 basis sets resulted in substantially reduced geometry factor errors in the calculated ^13^C NMR chemical shifts/shielding constants compared to their commensurate analogs, with the pecG-2 basis set being the best of all the considered basis sets.

## 1. Introduction

Practically every contemporary chemical research study involves NMR experiments. Due to this, ^13^C NMR spectrum analysis has now become one of the indispensable physical chemical tools for studying the structure and dynamics of large natural products. However, as is frequently the case for large compounds, the proper assignment of NMR signals is never a simple problem to solve [1,2]. Of utmost importance is precise quantum chemical modeling of the ^13^C NMR spectra [3]. Indeed, quantum chemical calculations may be of great assistance in resolving the NMR problem, but if and only if they are carried out properly at a sufficient level of electronic structure theory. In this sense, NMR quantum chemical modeling is full of nuances, starting from the quality of the equilibrium geometry, on top of which the NMR calculations are performed, to the computational protocol applied for the calculation of NMR chemical shifts [4,5].

Out of the many computational factors affecting the accuracy of the final values of the ^13^C NMR shielding constants/chemical shifts, the quality of the equilibrium geometry is one of the most important issues, despite its seemingly inconspicuous influence. Overall, the problem of the equilibrium geometry factor in chemical shift calculations has been recognized for some time now [6,7,8,9] and continues to emerge in modern NMR computational studies every now and then [10,11,12]. As a matter of fact, the quality of the equilibrium geometry is strictly dependent on the level of the electronic structure theory and on the one-electron basis set used at the geometry optimization stage. As for the level of theory, there is a distinct hierarchy of methods with clearly defined computational scaling factors, levels of electron correlation covering, and innate pros and cons [5]. Thus, one can be totally lucid as to what to expect from a particular method. For the basis sets, on the contrary, the issue is far more complicated. 

There were only a few works showing a strong dependence of the equilibrium geometry parameters on the basis set used at the geometry optimization stage [13,14,15]. In particular, Helgaker et al. [13] found an interesting relationship where bond lengths noticeably contract with improvements in the basis sets. This must be very important for geometry-dependent molecular properties such as NMR shielding constants. However, there was no systematic investigation of this issue until our recent studies. The first thing we showed was the dependence of the ^31^P chemical shifts on the basis set used on phosphorus atoms in the geometry optimization stage [16]. The study indicated a considerable variation in the average absolute error of calculated phosphorus chemical shifts compared to experimental data due to the geometry factor effect. The effect was thoroughly explored from the standpoint of the most effective polarization of the phosphorus 3p-shell, and new geometry-oriented pecG-*n* (*n* = 1, 2) basis sets for phosphorus atoms were developed on the basis of the property-energy consistent (PEC) method that was proposed earlier by Rusakov and Rusakova [17].

In our next paper [18], we proposed the pecG-*n* (*n* = 1, 2) basis sets for hydrogen and p-elements with 2–3 periods. The new basis sets, although rather moderate in size, turned out to be capable of giving equilibrium geometries of very high quality, which were comparable to those provided by considerably larger energy-optimized basis sets. The pecG-*n* basis sets were shown to be very efficient in reducing the geometry factor error in different second-order molecular properties, including static polarizabilities, static magnetizabilities, and NMR shielding constants [18]. The calculation of ^1^H, ^13^C, ^15^N, ^31^P, ^19^F, and ^29^Se NMR shielding constants was based on the coupled clusters singles and doubles method (CCSD) [19,20] and the CCSD equilibrium geometries. Taking into consideration the wide scope of the testing job and its high computational demand, only a limited number of rather small molecules were selected for each nucleus. Therefore, what is really important now for the pecG-*n* basis sets is to obtain indisputable proof of their effectiveness for NMR chemical shifts calculations with the aid of extensive and austere testing carried out at different levels of electron theory for a wide variety of challenging molecules, starting from very specific compounds with strong electron correlation effects to structurally entangled biologically active naturally occurring species. In this paper, we present the first such study performed for ^13^C NMR shielding constants/chemical shifts, as these are the most utilized in contemporary NMR analyses, specifically in NMR studies of large compounds of biological interest.

This paper is structured as follows. The introduction is followed by the “Results and Discussion” section starts from a summary on the PEC method and the pecG-*n* (*n* = 1, 2) basis sets in particular, with a strong emphasis on their distinction from standard energy-optimized basis sets. After that, the two-fold analysis of the performance of the pecG-*n* basis sets is presented, namely, the analysis comprises the theoretical study carried out without resorting to the experimental data, and the practical study based on the comparison of the calculated data with experimental data. The paper ends with a description of the computational details (“Materials and Methods”), conclusions, and the reference list.

## 2. Results and Discussion

### 2.1. The PEC Method and the pecG-n (n = 1, 2) Basis Sets

The property-energy consistent (PEC) method was introduced in our earlier paper [17]. This algorithm consists of the optimization of basis sets in relation to a certain molecular property, provided that the least possible total molecular energy is achieved. Namely, the basis set exponents are randomly generated around the starting basis set via Monte Carlo simulations. The generated arrays are verified based on whether they give the property of interest within a desired diapason or not. Of all the sets that provide the property in the desired range, only one set is selected, namely, the one that gives the lowest energy. The optimization of a property under an energetic constraint represents a nonlinear problem with multiple solutions, which is not correctly solvable by means of standard optimization techniques based on directed searches, like numerical Newton-like methods. In this sense, the PEC approach is the most suitable one for the nonlinear optimizations due to the fact that it performs a random search.

The pecG-*n* (*n* = 1, 2) basis sets [16,18] were created using the PEC algorithm. The optimization of these basis sets for a particular atom requires the energy-constrained minimization of the target function, representing a molecular energy gradient, relative to the lengths of the selected bonds that involve a particular atom. In fact, it is assumed that we can find a set of exponents which provide bond lengths as close to the ideal equilibrium values as possible, under the condition that they give the lowest molecular energy. The first tests carried out for the geometry-oriented pecG-*n* basis sets showed the expediency of the idea for a wide variety of molecular properties. For example, theoretical testing performed at the CCSD level of theory revealed that our second-level basis set, pecG-2, provided equilibrium geometries of the same quality as those obtained with the cc-pVQZ [21,22] basis set, resulting in almost the same mean absolute percentage errors (MAPE) for shielding constants as that for the data calculated using the cc-pVQZ geometries [18]. In addition, the pecG-2 basis set is approximately 1.5 times smaller than the cc-pVQZ basis set. Considering the fact that the main limiting factor for calculations of large molecules of biological interest is the basis set used, the computational benefit from using pecG-2 instead of the cc-pVQZ basis set is evident. 

It is also worth noting that the optimization of the pecG-*n* basis sets was completely based on the molecular calculations. Moreover, not one but numerous fitting molecules were considered in the case of each atom, except for hydrogen.

In this paper, we juxtapose our pecG-*n* basis sets with the two most popular series, namely the Dunning’s series and the Pople’s series. These represent what we call here standard energy-optimized basis sets. This means that only the energy minimum condition was pursued while optimizing their exponents. 

The Dunning’s basis sets, (aug)-cc-pVXZ (X = D, T, Q, …) [21,22], are representatives of the basis sets developed based only on atomic calculations. In particular, the cc-pVXZ basis sets for second-period atoms were developed based on the so-called “basic” (sp) primitive set, which was determined from atomic Hartree–Fock (HF) energy minimization. The exponents for the so-called correlation functions were then obtained from the correlated calculations on the atoms, specifically from the configuration interaction single and double excitation (CISD) wave function calculations. The d-functions of cc-pVXZ are too diffuse and, apparently, do not effectively describe the orbital polarization effect.

The Pople’s basis sets, *K-LMNG* [23,24], can be attributed to a more advanced group of basis sets, whose optimization at least partially involved molecular calculations. These can be augmented by various numbers of sets of polarization and diffuse functions. Here, the *K* and *L*, *M*, and *N* represent integers that denote the number of Gaussian primitives used to expand the inner-shell atomic orbitals and the inner- and outer-components of the valence shell functions, respectively. The Gaussian exponents for the inner- and valence-shell functions of the *K-LMNG* basis sets were obtained by minimizing the unrestricted Hartree–Fock (UHF) energy of the atomic ground state. The polarization sets (e.g., (2d,2p) or (3df,3pd)) were obtained by adding the functions with higher angular quantum numbers, with the exponents being the average of the exponents optimized for typical molecules incorporating a particular element. Thus, the Pople’s basis sets expanded with the polarization functions represent the result of mixed atomic and molecular energy-based optimizations.

Overall, by choosing the Dunning’s and Pople’s series, we took into consideration the representatives of two cardinally distinct families of basis sets, namely, those of correlation consistent and polarization consistent basis sets, respectively.

For later convenience, in Table 1, we give the compositions of the basis sets to be used and discussed further in this paper.

As one can see from Table 1, the pecG-1 and pecG-2 basis sets have the same composition and size as the 6-31G(2d,2p) and 6-311G(3df,3pd) basis sets; therefore, the latter can be thought of as direct competitors to our basis sets.

### 2.2. Theoretical Analysis of the pecG-n (n = 1, 2) Basis Sets

**Figure 1:**
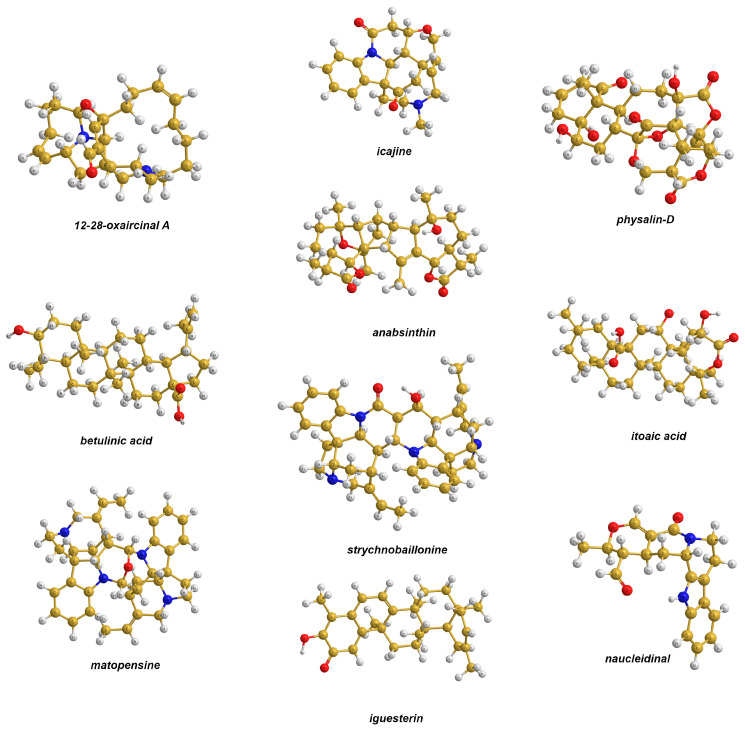
Compounds used in theoretical analysis (set **1**). Blue, red, yellow and gray balls represent nitrogen, oxygen, carbon and hydrogen atoms, respectively.

To carry out a theoretical examination of the performance of the pecG-*n* (*n* = 1, 2) basis sets against the other basis sets from the perspective of the geometry factor for the accuracy of calculations of ^13^C NMR shielding constants, we considered a set of ten natural products. These will be referred to as the molecules from set **1**. Their structures are presented in Figure 1.

As one can see from Figure 1, set **1** includes rather large spatially bulky compounds with up to 90 atoms each. These natural products are produced by living organisms such as marine sponges, fungi, and plants. Practically all of them represent very important compounds with potential biological activity. In particular, 12,28-oxaircinal A was isolated from three collections of an Indonesian sponge of the genus *Acanthostrongylophora*, together with 13 known manzamine alkaloids, which are known to have activity against infectious, tropical parasitic, and Alzheimer’s diseases [25]. Icajine represents one of the *Strychnos icaja* alkaloids, which is detected specifically in the stem, root, and collar bark of *S. icaja* and commonly possesses specific anti-plasmodial activity [26,27]. Physalin D is found in a fraction from the aerial parts of *Physalis angulate*, known in Brazil as *camapu*, which is a branched annual shrub that belongs to the Solanaceae family. Extracts from this plant have been used in traditional folk medicine to treat tumors. Physalin D was found to exhibit inhibitory activity against *Mycobacterium tuberculosis* [28]. Betulinic acid originates from lupane. It is a pentacyclic triterpene, a group characterized by cytotoxic properties, which can be isolated from plants (e.g., *Spirostachys africana*) or synthesized [29]. The anticancer property of betulinic acid and its derivatives was extensively studied [30,31,32,33]. Anabsinthin is a sesquiterpene lactone that can be extracted from the aerial parts of *Artemisia absinthium* L., commonly known as *wormwood*, which is a yellow, flowering, perennial plant that is distributed throughout various parts of Europe and Siberia and is used for its antiparasitic effects, as well as to treat anorexia and indigestion [34]. Itoaic acid or 2β,11β-dihydroxy-3,4-secofriedelolactone-27-oic acid represents a rare naturally occurring triterpenoid with a 3,4-seco-friedelolactone skeleton and potential anti-inflammatory activity against COX-2, which was isolated from *Flacouritaceae* plants [35]. Matopensine is a symmetrical bisindole alkaloid that can be extracted from the roots of *Strychnos matopensis* and *Strychnos kasengaensis*, which are plants from eastern Africa. Matopensine-type alkaloids were found to exhibited potent and selective activities against *Plasmodium* [36]. Strychnobaillonine is an asymmetrical bisindole alkaloid found in the roots of liana *Strychnos Icaja*, which is mainly used by local populations of Africa as an arrow or ordeal poison and as a treatment for skin diseases and chronic, persistent malaria [37]. Iguesterine was isolated from the root bark of *Catha cassinoides* [38]; it has cytostatic activity against HeLa cells [39]. Naucleidinal was isolated from the roots of *Nauclea Orientalis* and showed significant cytotoxic activity against both HeLa and KB cell lines [40].

These compounds pose a challenging task for NMR analysis because they have multiple chiral centers and a large number of carbon atoms with close electronic environments; hence, there are many carbon nuclei with almost equivalent chemical shifts. As a result, most of their ^13^C NMR spectra present a superposition of the individual second- or even higher-order multiplets, forming complex patterns, which are very difficult to analyze. Thus, it is very important to take into account as many factors of accuracy as possible within feasible limits. In this respect, the geometric factor that can change the calculated values of carbon chemical shifts within a couple ppm, depending on the basis set used at the geometry optimization stage, is very important. 

We investigated the performance of the pecG-*n* basis sets on the carbon shielding constants of natural products from set **1**, which in total gave us 292 values that resulted in very solid statistics. The equilibrium geometries of the compounds of set **1** were obtained at the DFT(M06-2X) [41] level of theory while taking into account solvent effects, using the different basis sets listed in Table 1. The solvent effects were calculated using the IEF-PCM model parametrized for chloroform as the solvent for 12-28-oxaircinal, icajine, iguesterin, itoaic acid, matopensine, naucleidinal, and strychnobaillonine; acetonitrile as the solvent for anabsinthin; pyridine as the solvent for betulinic acid; and dimethylsulfoxide as the solvent for physalin D.

We used the M06-2X functional because it was proven to give equilibrium geometrical parameters in close proximity to those obtained using high-quality ab initio methods [16,42,43], such as the coupled cluster singles and doubles with non-iterative perturbative triples (CCSD(T)) method [44]. Moreover, should a biological system of set **1** contain significant dispersion interactions, the M06-2X functional could be practically useful, as this functional was found to be highly successful at describing dispersion interactions for neutral molecular systems due to its portion of Grimme’s long-range dispersion corrections with an s6 scaling factor of 0.06 [43].

The equilibrium geometries for the reference values were obtained using the cc-pVQZ basis set. As one can see from Table 1, the quadruple-ζ quality cc-pVQZ basis set is considerably large, with as many as 30 and 55 functions for hydrogen and the second-row atoms, respectively. This makes the equilibrium geometries obtained with the cc-pVQZ basis set a good reference. 

The NMR shielding constants were calculated using the GIAO-DFT(B97-2) [45,46] method with the specialized pecS-2 basis set [47,48,49] for all atoms. The pecS-*n* basis sets were specifically optimized for the ^1^H, ^13^C, ^15^N, ^17^O, and ^31^P NMR shielding constant/chemical shift calculations via the PEC algorithm and were presented in our previous papers [47,48]. Overall, the pecS-*n* (*n* = 1, 2) basis sets are rather small in size, consisting of only 5/14 and 18/34 functions for hydrogen and the second-row atoms, respectively, and demonstrate a better accuracy compared to the other commensurate shielding-oriented basis sets [49]. Thus, our NMR-oriented basis sets embody a fine balance between size and accuracy, and hence, were our present choice.

For the method, the hybrid B97-2 functional was also chosen deliberately, as this functional or its modifications were found to be among the best and the most popular functionals for predicting isotropic NMR shielding constants [48,50,51,52]. Moreover, the B97-2 functional was used in the optimization of the pecS-*n* basis sets; therefore, the combination of the B97-2 functional with the second-level pecS-2 basis set must be an appropriate approach for the calculation of NMR chemical shifts using the DFT methodology.

**Figure 2:**
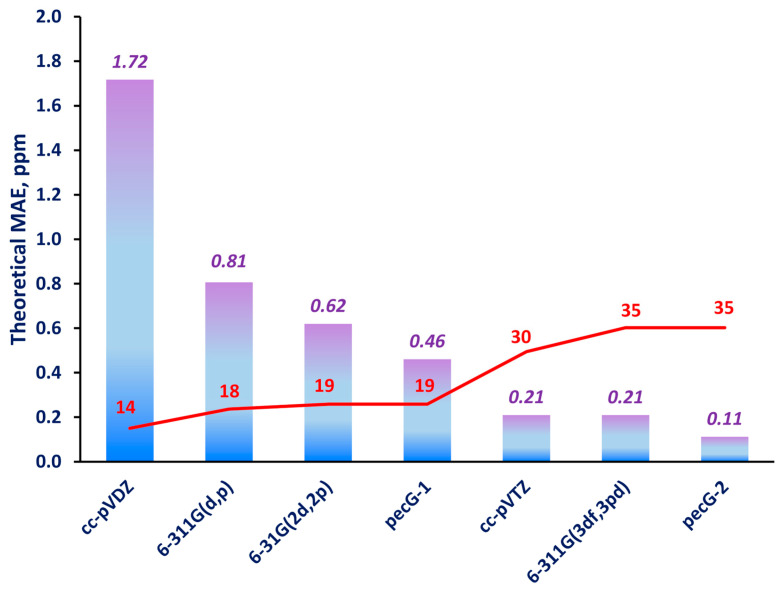
MAEs for the ^13^C NMR shielding constants calculated for set **1** using the equilibrium geometries obtained using different basis sets (listed along the abscissa) compared to the corresponding reference theoretical data. The red numbers indicate the sizes of the basis sets for the elements of the second period. In these calculations, only the lowest energy conformers were taken into account.

The mean absolute errors (MAEs) were estimated for the 292 carbon shielding constants of the molecules in set **1** that were calculated using the equilibrium geometries obtained using different basis sets against the reference theoretical shielding constants. These are presented in Figure 2.

As one can see from Figure 2, our pecG-1 and pecG-2 basis sets demonstrated noticeably better performance compared to the commensurate Pople-style basis sets, 6-31G(2d,2p) and 6-311G(3df,3pd), respectively. Namely, pecG-1 produced an approximately 1.3 times smaller MAE than that of the 6-31G(2d,2p) basis set, while the pecG-2 basis set turned out to be the best of all, and had an MAE that was two-fold smaller than that of the 6-311G(3df,3pd) basis set. 

The cc-pVDZ and 6-311G(d,p) basis sets are commonly used in the geometry optimizations performed nowadays, though, in accordance with our results, these basis sets appear to be the worst ones to use to obtain equilibrium geometries for calculations of carbon shielding constants. Accordingly, taking into account the subtlety with which the entangled ^13^C NMR spectra of natural products are modeled, we would not recommend using the cc-pVDZ and 6-311G(d,p) basis sets for geometry optimization of such compounds. 

The cc-pVTZ basis set is also one of the most popular basis sets for structure optimization; it is considered a rather high-quality basis set for describing molecular electronic structures. As can be seen from Table 1, its size is approximately one and a half times larger than that of the pecG-1 basis set and has only five fewer functions than the pecG-2 and 6-311G(3df,3pd) basis sets. The cc-pVTZ basis set can be seen as having a quality equal to that of the 6-311G(3df,3pd) basis set; *ergo*, out of the two, the former is preferable due to its fewer number of basis set functions. At the same time, the pecG-2 basis set, being only five functions larger, has a two-fold higher accuracy compared to the cc-pVTZ and 6-311G(3df,3pd) basis sets. Selecting between the cc-pVTZ and pecG-2 basis sets is a complicated matter that depends on the willingness to shift the balance towards either decreasing the computational costs or increasing the accuracy of the NMR calculations. Anyway, whenever one deals with natural products or other complicated systems with highly entangled NMR spectra, high-precision modeling of the ^13^C NMR spectra is required, and the pecG-2 basis set would apparently be a better choice for the geometry optimization at the expense of the increased computational costs compared to the cc-pVTZ basis set.

In spite of our original intention to devote this section to theoretical testing, a comparison with the experimental data for the molecules of set **1** was also possible, as their experimental ^13^C NMR chemical shifts are available [25,28,34,35,37,38,40,53,54,55,56]. Therefore, we evaluated scaled chemical shifts $\tilde{\delta }\left(\sigma_{i},\alpha \right)$ from the carbon shielding constants $\sigma_{i}$ using a physically meaningful linear regression model. This means that we applied the least squares method (LSM) with a slope equal exactly to −1:

(1) $$\tilde{\delta }\left(\sigma_{i},\alpha \right)=-\sigma_{i}+\alpha$$

In the LSM, the coefficient *α* is found from the minimization of the sum of squared residuals:

(2) $$S=\sum_{i=1}^{n}{\left(\delta_{exp,i}-\tilde{\delta }\left(\sigma_{i},\alpha \right)\right)}^{2}\to min$$

to give the following expression:

(3) $$\alpha =\frac{1}{n}\sum_{i=1}^{n}\left(\delta_{exp,i}+\sigma_{i}\right)$$

Should one consider the parameter α as representing an approximated shielding constant of a standard, the expression (1) becomes closely reminiscent of a well-known simplified IUPAC expression [57,58] for NMR chemical shifts, which is defined as the difference between the nuclear shielding constant of a standard and that of the compound under study. That is why our choice was this form of a linear model. It should be mentioned that we did not resort to a classical evaluation of the chemical shifts via the simplified IUPAC formula, as this brings about a systematic error due to the inaccuracy of the calculations of the carbon shielding constant of the reference compound. 

In order to assess the accuracy of the calculated $\tilde{\delta }$ against the experimental measurements, we first calculated the MAE for each compound from set 1 in virtue of the fact that their NMR data were recorded under different experimental conditions (solvent, temperature, etc.). Their resulting MAEs were then averaged to give the mean MAE for all ten compounds. In these calculations, all meaningful conformers were taken into account and Boltzmann averaging was performed. 

What we obtained did not quite fit into the picture that one might have expected. We observed a sporadic behavior of the MAEs of the basis sets used at the geometry optimization stage. That is, all mean MAEs were oscillating around the value of 2.4 ppm within a 0.05 ppm-wide range, without a clear tendency based on the qualities of the considered basis sets. For example, the cc-pVDZ, cc-pVTZ, and cc-pVQZ geometries gave almost the same MAEs for carbon chemical shifts.

In our opinion, the reason for such behavior may lie in many ambiguous factors that were not accounted for. The DFT method can fail to cover all the needed portions of the electron correlation, which might be crucial for some representatives of set 1 to correctly compare with the experimental data. Another complementing reason can be connected with the need for explicit accounting of solute–solvent interactions for some of the molecules, while for others, a simple polarization continuum model is sufficient. In these circumstances, we can see that a blunt comparison of the DFT results with the experiment results without considering many factors of accuracy will make the conclusions about so subtle an effect as the geometry factor pointless.

In this regard, we believe that the first and an insidious factor hampering a proper comparison of the theoretical values with experimental values is the lack of high-quality descriptions of the electron correlation effects in the considered systems. In our minds, in order to make the analysis of the performance of our basis sets based on the comparison with experimental data more valid, high-quality ab initio methods such as CCSD or CCSD(T) are the most important, while the other factors of accuracy are the second most important.

The coupled clusters singles and doubles (CCSD) method is a highly accurate ab initio correlated method which covers the electron correlation by approx. 98.3% and has a scaling computational factor of N^6^, with N being the number of basis set functions [59]. The CCSD(T) scheme that, on top of the CCSD, takes into account the triple excitations within the noniterative perturbative treatment, covers the electron correlation already by 99.7%, and has a computational scaling factor of N^7^ [59]. Ideally, it would have represented a great test if it were possible to apply these methods to the molecules from test set 1, but, unfortunately, these methods are prohibitive for systems of such a large size. Therefore, we introduced a set of systems with moderate sizes that possess very intricate electronic structures and represent a challenge to any computational tool. Eventually, we carried out an analysis based on the reference experimental data that are presented in the Section 2.3.

### 2.3. Analysis of the Performance of the pecG-n (n = 1, 2) Basis Sets Based on a Comparison of the Theoretical Data with Experimental Data

**Figure 3:**
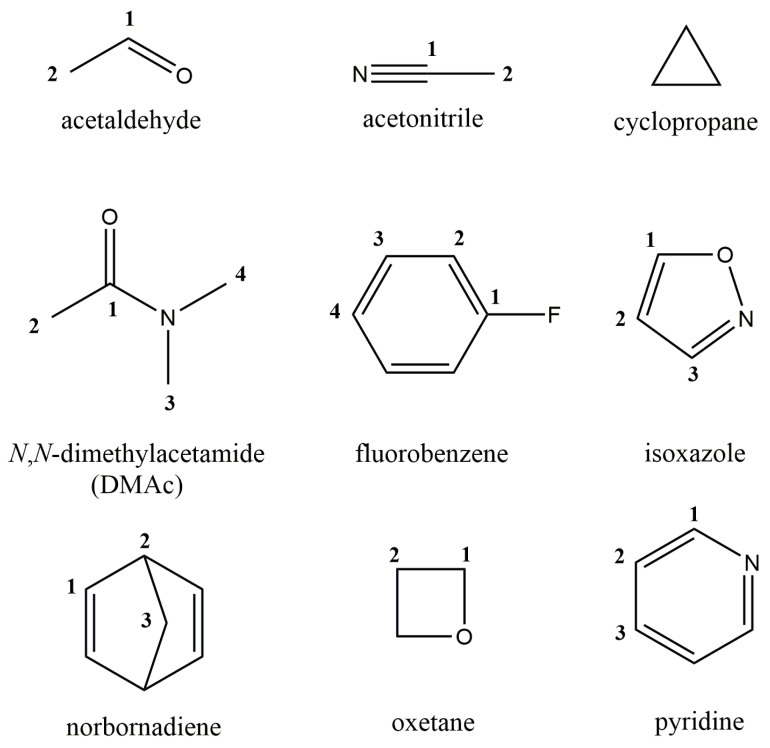
Compounds used in the analysis based on the comparison with experimental data (set **2**).

To perform the experiment-based test, we chose nine molecules from the DELTA50 set introduced in the paper by Cohen et al. [60]. These nine molecules formed test set **2**, which is shown in Figure 3. One can see that set **2** contains a wide variety of molecules with very difficult electronic structures, including, for example, molecules such as cyclopropane and oxetane, whose specific ring brings about a substantial steric strain. Different hybridization states of carbon atoms in bonding and their unique electron environment in each compound result in a wide variety of experimental ^13^C NMR chemical shifts, ranging from approx. −3 to 200 ppm. All measurements of carbon chemical shifts were carried out in CDCl_3_ and referenced to TMS at 0.00 ppm by Cohen et al. [60].

We performed calculations of the equilibrium geometries of the molecules of set **2** at the CCSD level of theory using different basis sets. This time, in view of the particularly demanding computational tasks, not all the basis sets from Table 1 were used in the geometry optimizations, i.e., apart from our basis sets, pecG-*n*, we only included into consideration the basis sets with the same size, the 6-31G(2d,2p) and 6-311G(3df,3pd) basis sets, and one very popular basis set, 6-311G(d,p). All the molecules of set **2** represent highly symmetric rigid structures so there was no need to carry out a conformational analysis. All geometry optimizations were performed taking into account the solvent effects. 

The gas phase calculations of the carbon shielding constants of set **2** were carried out using the GIAO-CCSD(T) method and the pecS-2 basis set on all atoms except for fluorine, for which, the pcS-2 basis set [61] was used. In view of the unavailability of the computational codes for coupled cluster calculations of shielding constants that account for solvent effects, the solvent corrections to the carbon shielding constants were taken into account at the GIAO-DFT(B97-2)/aug-cc-pV5Z level of theory. Unfortunately, the vibrational effects were not taken into account due to the extremely demanding computational costs needed for the computations within the coupled cluster method, while the DFT level was not used deliberately as it cannot be thought of as a trustworthy method for such electronically complicated molecules as those included in set **2**.

Overall, the computational scheme based on the CCSD(T) method for the property calculations performed on the equilibrium geometries obtained using the CCSD method represents a very accurate approach in terms of the treatment of electron correlation effects; therefore, in our opinion, this would constitute a proper benchmarking for our pecG-*n* basis sets based on the comparison with experimental data.

The ^13^C NMR chemical shifts were calculated from the corresponding shielding constants in accordance with the LSM method with the slope equal to −1, as was described in the Section 2.2 (see Equations (1)–(3)). The computed ^13^C NMR chemical shifts and the corresponding experimental data are presented in Table 2 below.

As one can see from Table 2, most of the calculated data are in very good agreement with the experimental data; however, in some exceptional cases, the theoretical values noticeably deviated from the experimental data, specifically, the NMR chemical shifts of C_1_ of DMAc and fluorobenzene. Apparently, these compounds have extremely complicated electron structures with carbon C_1_ being strongly involved in specific electron interactions. For example, DMAc is a representative of systems where the amino substituent at the C_1_ atom results in strong n ⟶ π* interaction, implying that the nitrogen atom donates the density of a lone pair (n) of electrons into the empty antibonding π* orbital of the nearby carbonyl group C_1_ = O, which typically causes a weakening and stretch of the carbon–oxygen bond [62]. At the same time, fluorobenzene is remarkable due to a considerable inductive electron withdrawal effect from the *ipso* carbon (C_1_) by fluorine, making the C_1_-F bond highly polarized and the C_1_ carbon lacking electrons to such an extent that it actually becomes the most de-shielded one in the system. This perturbation propagates along the ring, resulting in a considerable concertation of negative charge at the *ortho* position, making the C_2_ carbon the most shielded one. The electronic structure of fluorobenzene is so complicated that its electron-density difference plot is astoundingly similar to that for the phenyl cation with a nearby negative charge [63]! Evidently, a higher level of coupled cluster theory is needed for such complicated systems at all levels of calculation, not to mention the need to take into account explicit solute–solvent interactions. 

**Figure 4:**
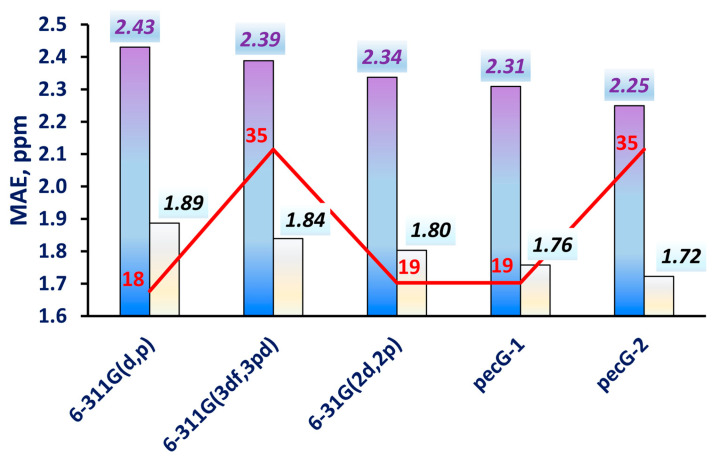
MAEs for the ^13^C NMR chemical shifts calculated for test set **2** using equilibrium geometries obtained using different basis sets (listed along the abscissa) against the corresponding experimental data. The second bars show the altered statistical figures evaluated without taking into account the chemical shift of C_1_ of DMAc and fluorobenzene. The red numbers indicate the sizes of the basis sets for the second period elements.

In this part of work, however, our interest lay in testing the pecG-*n* basis sets using a very high level of electronic theory for highly complicated electronic systems. It was not our intention to investigate all factors of accuracy for abnormal cases specifically, even though we obtained a couple of noticeably underestimated values. In fact, these values did not change the tendency for the basis sets even a bit. This can be clearly seen in Figure 4, where we plotted the calculated MAEs from Table 2 together with the MAEs calculated without taking into account a certain “particularly problematic” C_1_ carbon of DMAc and fluorobenzene. 

**Figure 5:**
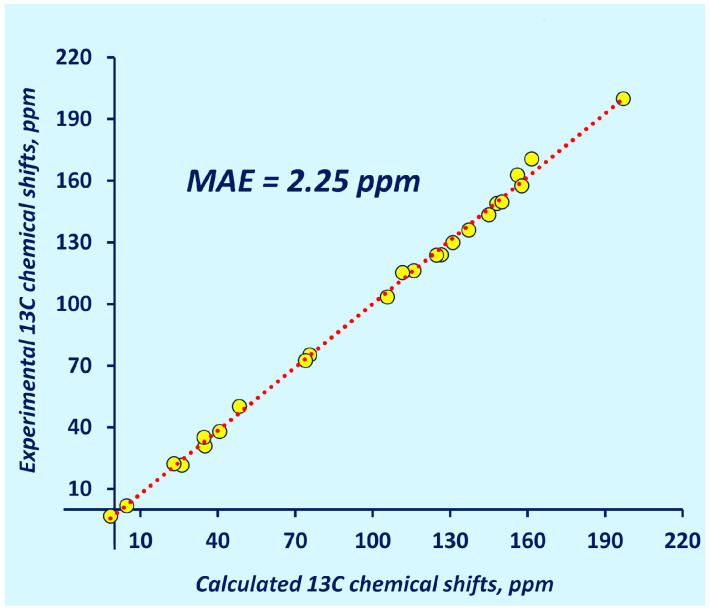
Correlation plot for the ^13^C NMR shielding constants of test set **2** calculated at the GIAO-CCSD(T)/pecS-2 level using equilibrium geometries obtained at the CCSD/pecG-2 level against the corresponding experimental data.

As one can see in Figure 4, the pecG-1 and pecG-2 basis sets gave equilibrium geometries that resulted in carbon chemical shifts with a higher accuracy than those calculated using the geometries obtained using the direct analogs of our basis sets, the 6-31G(2d,2p) and 6-311G(3df,3pd) basis sets, respectively. Meantime, the 6-311G(d,p) basis set repeated its own behavior at the DFT level where it was among the worst basis set for geometry optimization for NMR calculations. Thus, based on the presented benchmark calculations, we corroborated the superiority of the pecG-1 and pecG-2 basis sets over their analogs, with the pecG-2 basis set being the best for the geometry optimizations preceding carbon NMR chemical shifts calculations. To demonstrate the performance of the pecG-2 basis set, the correlation plot of the theoretical GIAO-CCSD(T)/pecS-2 chemical shifts calculated using the CCSD/pecG-2 equilibrium geometries for test set **2** against the experimental data is shown in Figure 5.

## 3. Materials and Methods

Optimizations of structures from test set **1** and set **2** were carried out accordingly, at the DFT and CCSD levels of theory in the Gaussian program [64]. All equilibrium geometries were obtained while taking into account solvent effects. For this purpose, the integral equation formalism of the polarizable continuum model, the IEF-PCM [65,66], was used. The IEF-PCM was parametrized in accordance with the solvents defined in the experimental papers. For the specification for each compound, please see the “Results and Discussion” section. All equilibrium geometries, obtained at different levels of electron theory and using different basis sets, are presented in the Supplementary Materials. 

All GIAO-DFT calculations of ^13^C NMR shielding constants, either with or without accounting for the solvent effects, were conducted in the Gaussian program, while all gas phase CCSD(T) calculations of ^13^C NMR shieldings were carried out in the CFOUR program [67]. All calculated NMR shielding constants are presented in the Supplementary Materials (Tables S1–S15).

## 4. Conclusions

We investigated the performance of our recently proposed pecG-*n* (*n* = 1, 2) basis sets in geometry optimization, using the example of ^13^C NMR shielding constants/chemical shifts calculations. They were able to improve the final accuracy of the calculated NMR data compared to the other basis sets commonly used at the geometry optimization stage, such as Dunning’ cc-pVXZ (X = D, T) and the Pople-style 6-31G(2d,2p), 6-311G(3df,3pd), and 6-311G(d,p) basis sets.

The theoretical analysis was based on a comparison of the carbon shielding constants calculated at the GIAO-DFT(B97-2)//DFT(M06-2X) level for a wide variety of natural products (set **1**) with the reference data obtained using the equilibrium geometries calculated using the cc-pVQZ basis set. Most importantly, this analysis showed that the pecG-1 and pecG-2 basis sets gave substantially better equilibrium geometries than their direct Pople-style analogs with the same size, the 6-31G(2d,2p) and 6-311G(3df,3pd) basis sets, respectively, and, in fact, they produced a substantially smaller geometry factor error in the calculated carbon shielding constants. Out of all considered basis sets, the pecG-2 basis set was found to be the best, providing an MAE of only 0.11 ppm for the shielding constants of the molecules of test set **1**.

The practical analysis was based upon a comparison of the calculated ^13^C NMR chemical shifts with experimental data. This analysis involved a highly demanding computational protocol using the CCSD level of theory for the geometry optimizations and the GIAO-CCSD(T) level for the shielding constant calculations. This analysis was performed on a set of very electronically complicated systems (set **2**) and revealed the same pattern that was observed in the theoretical analysis: the pecG-*n* (*n* = 1, 2) basis sets showed considerably superior efficacy in quelling the geometry factor error in the ^13^C NMR chemical shift calculations, with the pecG-2 basis set being the best. The MAE achieved with the pecG-2 basis set for test set **2** compared to the experimental data was found to be 2.25 ppm.

Based on the obtained results, we strongly recommend using the pecG-2 basis set in the geometry optimizations for ^13^C NMR chemical shifts calculations whenever highly precision modeling is required, like in the case of large-sized natural products. On the other hand, if there is a strong limitation on the basis set functions to be treated, the pecG-1 basis set is recommended. Basically, the pecG-1 and pecG-2 basis sets were conceived so as to provide the smallest geometry factor error in molecular property calculations; thus, as they are moderate in size, they are the best for the geometry optimizations performed as part of NMR spectra modeling. In this work, this fact was successfully corroborated using the example of ^13^C NMR chemical shift calculations.

## Figures and Tables

**Table 1:** Compositions of basis sets considered in theoretical and practical analyses.

Basis Set	Element	Contracted Composition	Number of Contracted Basis Functions
Rusakov’s pecG-*n* series			
pecG-1	H	[2s2p]	8
	B-F	[3s2p2d]	19
pecG-2	H	[3s3p1d]	17
	B-F	[4s3p3d1f]	35
Dunning’s cc-pVXZ series			
cc-pVDZ	H	[2s1p]	5
	B-F	[3s2p1d]	14
cc-pVTZ	H	[3s2p1d]	14
	B-F	[4s3p2d1f]	30
cc-pVQZ	H	[4s3p2d1f]	30
	B-F	[5s4p3d2f1g]	55
Pople-style *K-LMNG*(*x*,*y*) series			
6-311G(d,p)	H	[3s1p]	6
	B-F	[4s3p1d]	18
6-31G(2d,2p)	H	[2s2p]	8
	B-F	[3s2p2d]	19
6-311G(3df,3pd)	H	[3s3p1d]	17
	B-F	[4s3p3d1f]	35

**Table 2:** Calculated and experimental ^13^C NMR chemical shifts (in ppm) of molecules in set **2**.

Molecule	No. of Carbon Atoms ^1^	6-311G(d,p)	6-31G(2d,2p)	6-311G(3df,3pd)	pecG-1	pecG-2	Exp. ^2^
Acetaldehyde	1	196.19	197.17	196.25	196.37	196.97	199.97
	2	34.92	34.70	35.09	34.90	35.02	30.99
Acetonitrile	1	4.95	4.84	4.85	4.61	4.65	1.91
	2	115.07	115.73	115.57	115.91	115.93	116.33
Cyclopropane		−1.61	−1.66	−1.37	−1.63	−1.57	−3.15
DMAc	1	160.74	161.03	161.06	160.69	161.50	170.66
	2	25.58	25.25	26.12	25.34	26.10	21.58
	3	40.35	39.75	40.60	39.86	40.61	38.05
	4	34.32	33.74	34.61	33.88	34.60	35.20
Fluorobenzene	1	155.98	156.06	155.61	156.09	155.93	162.86
	2	111.85	111.65	111.51	111.72	111.49	115.32
	3	131.15	130.89	131.09	131.00	130.94	129.96
	4	127.11	126.88	126.52	126.89	126.55	123.98
Isoxazole	1	158.00	158.72	157.55	158.28	157.62	157.64
	2	106.04	105.96	105.78	105.95	105.65	103.47
	3	148.72	149.18	148.16	148.83	147.96	149.02
Norbornadiene	1	145.04	145.27	145.17	145.05	144.97	143.43
	2	48.28	48.27	48.29	48.63	48.27	50.26
	3	74.67	74.19	75.34	75.13	75.50	75.32
Oxetane	1	73.80	74.05	73.96	73.87	73.94	72.55
	2	23.41	23.09	23.46	23.41	22.91	22.35
Pyridine	1	150.18	150.07	149.91	150.29	149.98	149.74
	2	125.15	124.97	124.99	124.95	124.69	123.78
	3	137.53	137.41	137.21	137.33	137.11	136.09
*α*		196.12	196.82	197.81	197.18	198.54	
MAE		2.43	2.34	2.39	2.31	2.25	

**^1^** For the enumeration of atoms, see Figure 2. ^2^ For experimental data, see reference [60].

## Data Availability

The original contributions presented in the study are included in the article and Supplementary Materials, further inquiries can be directed to the corresponding author.

## Supplementary Materials

The following supporting information can be downloaded at: https://www.mdpi.com/article/10.3390/ijms251910588/s1.
