# Supplementary material for: Quelling the Geometry Factor Effect in Quantum Chemical Calculations of 13C NMR Chemical Shifts with the Aid of the pecG-n (n = 1, 2) Basis Sets
Source: Int J Mol Sci. 2024 Oct 1;25(19):10588. doi: 10.3390/ijms251910588 (PMC11477434; doi:10.3390/ijms251910588)
Supplement: Supplementary file 1 [file ijms-25-10588-s001.zip › ijms-3232300-supplementary.pdf]

Supplementary Materials

The pecG-1 and pecG-2 basis sets in Gaussian format:

pecG-1 for H 4

pecG-1 for C 5

pecG-1 for B 6

pecG-1 for O 7

pecG-1 for N 8

pecG-1 for F 9

pecG-1 for Al 11

pecG-1 for Si 13

pecG-1 for P 15

pecG-1 for S 17

pecG-1 for Cl 20

pecG-2 for H 22

pecG-2 for C 23

pecG-2 for B 24

pecG-2 for O 26

pecG-2 for N 27

pecG-2 for F 29

pecG-2 for Al 30

pecG-2 for Si 33

pecG-2 for P 36

pecG-2 for S 39

pecG-2 for Cl 42

Equilibrium geometries of molecules from set **1** calculated at the **DFT(M06-2X)** level of theory with different ***basis sets:***

6-31G(2d,2p) 45

6-311G(3df,3pd) 80

6-311G(d,p) 115

cc-pVDZ 150

cc-pVTZ 185

cc-pVQZ 220

pecG-1 255

pecG-2 290

Equilibrium geometries of molecules from set **2** calculated at the **CCSD** level of theory with different ***basis sets:***

6-31G(2d,2p) 325

6-311G(3df,3pd) 328

6-311G(d,p) 332

pecG-1 336

pecG-2 340

Table S1. ^{13}C NMR shielding constants of **12-28-oxaircinal A** calculated at the GIAO-DFT(B97-2)/pecS-2 level using the equilibrium geometries obtained within the DFT(M06-2X) method with different basis sets. 344

Table S2-A. ^{13}C NMR shielding constants of **anabsinthin (conf. 1)** calculated at the GIAO-DFT(B97-2)/pecS-2 level using the equilibrium geometries obtained within the DFT(M06-2X) method with different basis sets. 344

Table S2-B. ^{13}C NMR shielding constants of **anabsinthin (conf. 2)** calculated at the GIAO-DFT(B97-2)/pecS-2 level using the equilibrium geometries obtained within the DFT(M06-2X) method with different basis sets. 345

Table S3. ^{13}C NMR shielding constants of **betulinic acid** calculated at the GIAO-DFT(B97-2)/pecS-2 level using the equilibrium geometries obtained within the DFT(M06-2X) method with different basis sets. 346

Table S4-A. ^{13}C NMR shielding constants of **icajine (conf. 1)** calculated at the GIAO-DFT(B97-2)/pecS-2 level using the equilibrium geometries obtained within the DFT(M06-2X) method with different basis sets. 347

Table S4-B. ^{13}C NMR shielding constants of **icajine (conf. 2)** calculated at the GIAO-DFT(B97-2)/pecS-2 level using the equilibrium geometries obtained within the DFT(M06-2X) method with different basis sets...348

Table S5. ^{13}C NMR shielding constants of **iguesterin** calculated at the GIAO-DFT(B97-2)/pecS-2 level using the equilibrium geometries obtained within the DFT(M06-2X) method with different basis sets...349

Table S6-A. ^{13}C NMR shielding constants of **itoaic acid (conf. 1)** calculated at the GIAO-DFT(B97-2)/pecS-2 level using the equilibrium geometries obtained within the DFT(M06-2X) method with different basis sets...350

Table S6-B. ^{13}C NMR shielding constants of **itoaic acid (conf. 2)** calculated at the GIAO-DFT(B97-2)/pecS-2 level using the equilibrium geometries obtained within the DFT(M06-2X) method with different basis sets...351

Table S7. ^{13}C NMR shielding constants of **matopensine** calculated at the GIAO-DFT(B97-2)/pecS-2 level using the equilibrium geometries obtained within the DFT(M06-2X) method with different basis sets...352

Table S8. ^{13}C NMR shielding constants of **naucleidinal** calculated at the GIAO-DFT(B97-2)/pecS-2 level using the equilibrium geometries obtained within the DFT(M06-2X) method with different basis sets...353

Table S9. ^{13}C NMR shielding constants of **physalin D** calculated at the GIAO-DFT(B97-2)/pecS-2 level using the equilibrium geometries obtained within the DFT(M06-2X) method with different basis sets...354

Table S10-A. ^{13}C NMR shielding constants of **strychnobaillonine (conf. 1)** calculated at the GIAO-DFT(B97-2)/pecS-2 level using the equilibrium geometries obtained within the DFT(M06-2X) method with different basis sets...355

Table S10-B. ^{13}C NMR shielding constants of **strychnobaillonine (conf. 2)** calculated at the GIAO-DFT(B97-2)/pecS-2 level using the equilibrium geometries obtained within the DFT(M06-2X) method with different basis sets...356

Table S11. ^{13}C NMR shielding constants and chemical shifts (in ppm) of molecules in set **2** calculated at the GIAO-CCSD(T)/pecS-2 level on equilibrium geometries obtained at the CCSD level with 6-311G(d,p) basis set 357

Table S12. ^{13}C NMR shielding constants and chemical shifts (in ppm) of molecules in set **2** calculated at the GIAO-CCSD(T)/pecS-2 level on equilibrium geometries obtained at the CCSD level with 6-31G(2d,2p) basis set 358

Table S13. ^{13}C NMR shielding constants and chemical shifts (in ppm) of molecules in set **2** calculated at the GIAO-CCSD(T)/pecS-2 level on equilibrium geometries obtained at the CCSD level with 6-311G(3df,3pd) basis set...360

Table S14. ^{13}C NMR shielding constants and chemical shifts (in ppm) of molecules in set **2** calculated at the GIAO-CCSD(T)/pecS-2 level on equilibrium geometries obtained at the CCSD level with pecG-1 basis set. 361

Table S15. ^{13}C NMR shielding constants and chemical shifts (in ppm) of molecules in set **2** calculated at the GIAO-CCSD(T)/pecS-2 level on equilibrium geometries obtained at the CCSD level with pecG-2 basis set 362

The pecG-1 and pecG-2 basis sets in Gaussian format. (Please, provide a proper citation for the pecG-n basis sets in accordance with the following reference: *J. Chem. Theory Comput.* 2024, 20, 15, 6661–6673. DOI: 10.1021/acs.jctc.4c00772)

pecG-1 for H

```
H    0
S    4   1.00
     1.726773E+01          1.155435E-02
     2.584771E+00          8.188898E-02
     5.911789E-01          2.602482E-01
     1.512939E-01          2.558521E-01
S    1   1.00
     1.512939E-01          1.000000E+00
P    1   1.00
     1.996236E+00          1.000000E+00
P    1   1.00
```

3.635271E-01 1.000000E+00

****

pecG-1 for C

C 0

S 9 1.00

7.028930E+03 6.482732E-04

1.050132E+03 5.005043E-03

2.407022E+02 2.526347E-02

6.864043E+01 9.525953E-02

2.237852E+01 2.593621E-01

7.962079E+00 4.448051E-01

2.871624E+00 3.095424E-01

5.973216E-01 1.787317E-02

2.092910E-01 -2.519475E-03

S 9 1.00

7.028930E+03 -1.410937E-04

1.050132E+03 -1.122494E-03

2.407022E+02 -5.490821E-03

6.864043E+01 -2.244635E-02

2.237852E+01 -6.409612E-02

7.962079E+00 -1.416848E-01

2.871624E+00 -1.505093E-01

5.973216E-01 4.206730E-01

2.092910E-01 6.941325E-01

S 1 1.00

2.092910E-01 1.000000E+00

P 4 1.00

1.063810E+01 3.475641E-02

2.273078E+00 1.732268E-01

6.305201E-01 4.497840E-01

1.685179E-01 5.177860E-01

P 1 1.00

1.685179E-01 1.000000E+00

D 1 1.00

1.183679E+00 1.000000E+00

D 1 1.00

3.747963E-01 1.000000E+00

****

pecG-1 for B

B 0

S 9 1.00

4.726026E+03 6.635245E-04

7.156500E+02 5.060070E-03

1.650713E+02 2.529878E-02

4.722895E+01 9.512311E-02

1.533606E+01 2.593029E-01

5.396592E+00 4.482586E-01

1.917319E+00 3.106380E-01

3.742255E-01 1.615707E-02

1.301850E-01 -4.332378E-03

S 9 1.00

4.726026E+03 -1.147264E-04

7.156500E+02 -8.783357E-04

1.650713E+02 -4.377611E-03

4.722895E+01 -1.726097E-02

1.533606E+01 -5.068152E-02

5.396592E+00 -1.089786E-01

1.917319E+00 -1.261777E-01

3.742255E-01 3.613034E-01

1.301850E-01 3.174867E-01

S 1 1.00

1.301850E-01 1.000000E+00

P 4 1.00

6.767905E+00 2.343805E-02

1.408396E+00 1.122166E-01

4.018558E-01 2.960340E-01

1.097371E-01 1.781733E-01

P 1 1.00

1.097371E-01 1.000000E+00

D 1 1.00

8.521566E-01 1.000000E+00

D 1 1.00

3.002842E-01 1.000000E+00

****

pecG-1 for O

O 0

S 9 1.00

1.187638E+04 7.020137E-04

1.760696E+03 5.511086E-03

3.954833E+02 2.859489E-02

1.109622E+02 1.086225E-01

3.573035E+01 2.925222E-01

1.282155E+01 4.437143E-01

4.818687E+00 2.636934E-01

8.866683E-01 1.336894E-02

2.357453E-01 -1.913528E-03

S 9 1.00

1.187638E+04 -1.616751E-04

1.760696E+03 -1.317019E-03

3.954833E+02 -6.498204E-03

1.109622E+02 -2.775318E-02

3.573035E+01 -7.483140E-02

1.282155E+01 -1.773312E-01

4.818687E+00 -1.011830E-01

8.866683E-01 7.174312E-01

2.357453E-01 4.216525E-01

S 1 1.00

2.357453E-01 1.000000E+00

P 4 1.00

1.924716E+01 4.009372E-02

4.163032E+00 2.092887E-01

1.125766E+00 4.985574E-01

2.990367E-01 4.698264E-01

P 1 1.00

2.990367E-01 1.000000E+00

D 1 1.00

2.170627E+00 1.000000E+00

D 1 1.00

7.865325E-01 1.000000E+00

****

pecG-1 for N

N 0

S 9 1.00

9.282091E+03 6.725557E-04

1.406047E+03 5.113770E-03

3.242008E+02 2.582270E-02

9.149231E+01 9.979961E-02

2.936748E+01 2.731790E-01

1.048187E+01 4.455481E-01

3.835237E+00 2.913829E-01
7.556584E-01 1.373052E-02
1.824534E-01 -1.569631E-03
S 9 1.00
9.282091E+03 -1.532553E-04
1.406047E+03 -1.166651E-03
3.242008E+02 -5.815043E-03
9.149231E+01 -2.384896E-02
2.936748E+01 -7.040850E-02
1.048187E+01 -1.562964E-01
3.835237E+00 -1.483071E-01
7.556584E-01 6.564361E-01
1.824534E-01 5.056478E-01
S 1 1.00
1.824534E-01 1.000000E+00
P 4 1.00
1.360983E+01 4.394905E-02
2.871692E+00 2.130442E-01
8.032364E-01 5.192709E-01
1.789075E-01 4.476646E-01
P 1 1.00
1.789075E-01 1.000000E+00
D 1 1.00
1.720422E+00 1.000000E+00
D 1 1.00
4.734005E-01 1.000000E+00
****

pecG-1 for F

F 0
S 9 1.00

1.474184E+04	7.296880E-04
2.129806E+03	5.882802E-03
4.679122E+02	3.141812E-02
1.287375E+02	1.207225E-01
4.072743E+01	3.229485E-01
1.440802E+01	4.434853E-01
5.555100E+00	2.114500E-01
1.168950E+00	1.013541E-02
3.314980E-01	-1.203501E-03

S 9 1.00

1.474184E+04	-1.665195E-04
2.129806E+03	-1.395666E-03
4.679122E+02	-7.078791E-03
1.287375E+02	-3.082664E-02
4.072743E+01	-8.508857E-02
1.440802E+01	-1.881744E-01
5.555100E+00	-7.018763E-02
1.168950E+00	6.920690E-01
3.314980E-01	4.503822E-01

S 1 1.00

3.314980E-01	1.000000E+00

P 4 1.00

2.317718E+01	4.441540E-02
4.990951E+00	2.304943E-01
1.336524E+00	5.058996E-01
3.397009E-01	4.410690E-01

P 1 1.00

3.397009E-01	1.000000E+00

D 1 1.00

2.977783E+00	1.000000E+00

D 1 1.00

9.165046E-01 1.000000E+00

****

pecG-1 for Al

Al 0

S 12 1.00

6.263265E+04 2.980853E-04

9.503888E+03 2.283826E-03

2.173098E+03 1.173868E-02

6.168124E+02 4.725590E-02

2.010375E+02 1.480129E-01

7.219961E+01 3.361668E-01

2.787771E+01 4.156387E-01

1.145196E+01 1.847466E-01

3.217161E+00 1.049797E-02

1.141872E+00 -1.192612E-03

1.733109E-01 4.147670E-04

6.713403E-02 -2.075594E-04

S 12 1.00

6.263265E+04 -7.749348E-05

9.503888E+03 -5.915258E-04

2.173098E+03 -3.113791E-03

6.168124E+02 -1.248403E-02

2.010375E+02 -4.256367E-02

7.219961E+01 -1.056669E-01

2.787771E+01 -1.978039E-01

1.145196E+01 -7.311128E-02

3.217161E+00 5.487629E-01

1.141872E+00 5.665793E-01

1.733109E-01 2.575164E-02

6.713403E-02 -8.436955E-03

S 12 1.00

6.263265E+04 1.762864E-05

9.503888E+03 1.335286E-04

2.173098E+03 7.120058E-04

6.168124E+02 2.802409E-03

2.010375E+02 9.843299E-03

7.219961E+01 2.438473E-02

2.787771E+01 4.728008E-02

1.145196E+01 1.784292E-02

3.217161E+00 -1.683837E-01

1.141872E+00 -2.856890E-01

1.733109E-01 6.097632E-01

6.713403E-02 5.395240E-01

S 1 1.00

6.713403E-02 1.000000E+00

P 8 1.00

2.680894E+02 3.841167E-03

6.265447E+01 3.022184E-02

1.951429E+01 1.260634E-01

7.036996E+00 3.132508E-01

2.622560E+00 4.488983E-01

9.592527E-01 2.758336E-01

2.326292E-01 1.827349E-02

6.731160E-02 -2.013393E-03

P 8 1.00

2.680894E+02 -7.157894E-04

6.265447E+01 -5.441809E-03

1.951429E+01 -2.472735E-02

7.036996E+00 -6.099299E-02

2.622560E+00 -9.390426E-02

9.592527E-01 -3.639118E-02

2.326292E-01 4.186603E-01

6.731160E-02 6.960880E-01

P 1 1.00

6.731160E-02 1.000000E+00

D 1 1.00

7.436225E-01 1.000000E+00

D 1 1.00

1.925993E-01 1.000000E+00

****

pecG-1 for Si

Si 0

S 12 1.00

7.951173E+04 2.665588E-04

1.191887E+04 2.072274E-03

2.703690E+03 1.072013E-02

7.700177E+02 4.306201E-02

2.507455E+02 1.369631E-01

8.981569E+01 3.209567E-01

3.448110E+01 4.191901E-01

1.408657E+01 2.028639E-01

3.984099E+00 1.325720E-02

1.454233E+00 -1.861792E-03

2.548178E-01 6.432901E-04

1.071707E-01 -3.293505E-04

S 12 1.00

7.951173E+04 -7.213354E-05

1.191887E+04 -5.479959E-04

2.703690E+03 -2.969333E-03

7.700177E+02	-1.169250E-02
2.507455E+02	-4.074254E-02
8.981569E+01	-1.036200E-01
3.448110E+01	-1.999457E-01
1.408657E+01	-9.444317E-02
3.984099E+00	5.341733E-01
1.454233E+00	5.884339E-01
2.548178E-01	3.334335E-02
1.071707E-01	-9.917880E-03

S 12 1.00

7.951173E+04	1.839206E-05
1.191887E+04	1.428807E-04
2.703690E+03	7.308785E-04
7.700177E+02	3.001792E-03
2.507455E+02	1.027345E-02
8.981569E+01	2.687592E-02
3.448110E+01	5.320629E-02
1.408657E+01	2.674458E-02
3.984099E+00	-1.870003E-01
1.454233E+00	-3.198266E-01
2.548178E-01	5.399435E-01
1.071707E-01	6.181387E-01

S 1 1.00

1.071707E-01	1.000000E+00

P 8 1.00

3.328757E+02	3.556486E-03
7.890898E+01	2.767015E-02
2.477577E+01	1.181277E-01
8.963413E+00	3.047437E-01
3.371365E+00	4.520851E-01

1.256315E+00 2.777635E-01

3.198156E-01 1.925149E-02

9.695066E-02 -2.068967E-03

P 8 1.00

3.328757E+02 -7.938296E-04

7.890898E+01 -6.107588E-03

2.477577E+01 -2.717617E-02

8.963413E+00 -7.160198E-02

3.371365E+00 -1.143089E-01

1.256315E+00 -3.143204E-02

3.198156E-01 4.383573E-01

9.695066E-02 6.420094E-01

P 1 1.00

9.695066E-02 1.000000E+00

D 1 1.00

1.017669E+00 1.000000E+00

D 1 1.00

2.895477E-01 1.000000E+00

****

pecG-1 for P (Proper citation for this basis set: *J. Chem. Phys.* 160, 084109 (2024). DOI: 10.1063/5.0193227)

P 0

S 12 1.00

9.637522E+04 2.502937E-04

1.442094E+04 1.909639E-03

3.373922E+03 9.543702E-03

9.987463E+02 3.710608E-02

3.165913E+02 1.340088E-01

1.039364E+02 3.427757E-01

3.830046E+01 4.280217E-01

1.577327E+01 1.870635E-01

4.682116E+00	1.324289E-02
1.763034E+00	-2.164621E-03
3.197050E-01	5.846407E-04
1.044095E-01	-2.432153E-04

S 12 1.00

9.637522E+04	-6.698556E-05
1.442094E+04	-5.174174E-04
3.373922E+03	-2.664946E-03
9.987463E+02	-1.029956E-02
3.165913E+02	-4.027587E-02
1.039364E+02	-1.132657E-01
3.830046E+01	-2.134997E-01
1.577327E+01	-8.153624E-02
4.682116E+00	5.299669E-01
1.763034E+00	6.028190E-01
3.197050E-01	2.884243E-02
1.044095E-01	-6.471668E-03

S 12 1.00

9.637522E+04	1.763024E-05
1.442094E+04	1.353432E-04
3.373922E+03	6.958355E-04
9.987463E+02	2.683075E-03
3.165913E+02	1.042304E-02
1.039364E+02	3.040783E-02
3.830046E+01	5.783546E-02
1.577327E+01	2.612163E-02
4.682116E+00	-1.964684E-01
1.763034E+00	-3.354099E-01
3.197050E-01	6.296758E-01
1.044095E-01	5.500596E-01

S 1 1.00

1.044095E-01 1.000000E+00

P 8 1.00

4.272177E+02 3.165575E-03

1.037720E+02 2.432229E-02

3.323294E+01 1.037259E-01

1.218404E+01 3.010395E-01

4.451771E+00 4.862120E-01

1.661366E+00 3.186021E-01

4.325547E-01 2.253436E-02

1.354318E-01 -2.427999E-03

P 8 1.00

4.272177E+02 -7.304252E-04

1.037720E+02 -4.545074E-03

3.323294E+01 -2.119023E-02

1.218404E+01 -5.996469E-02

4.451771E+00 -1.149261E-01

1.661366E+00 -2.306700E-02

4.325547E-01 4.584161E-01

1.354318E-01 7.699420E-01

P 1 1.00

1.354318E-01 1.000000E+00

D 1 1.00

1.484645E+00 1.000000E+00

D 1 1.00

3.658861E-01 1.000000E+00

****

pecG-1 for S

S 0

S 12 1.00

1.182093E+05	2.340139E-04
1.661890E+04	1.999945E-03
3.614701E+03	1.058872E-02
1.031236E+03	4.156372E-02
3.433347E+02	1.281836E-01
1.261194E+02	2.997711E-01
4.943525E+01	4.161641E-01
2.027292E+01	2.359676E-01
5.753575E+00	1.864753E-02
2.177005E+00	-3.019342E-03
3.974195E-01	6.815510E-04
1.100739E-01	-2.620908E-04

S 12 1.00

1.182093E+05	-6.507434E-05
1.661890E+04	-5.468246E-04
3.614701E+03	-2.973541E-03
1.031236E+03	-1.170641E-02
3.433347E+02	-3.908632E-02
1.261194E+02	-9.855648E-02
4.943525E+01	-1.969372E-01
2.027292E+01	-1.250336E-01
5.753575E+00	5.017286E-01
2.177005E+00	6.213574E-01
3.974195E-01	3.562669E-02
1.100739E-01	-7.017811E-03

S 12 1.00

1.182093E+05	1.883151E-05
1.661890E+04	1.602018E-04
3.614701E+03	8.713370E-04
1.031236E+03	3.436574E-03

3.433347E+02 1.159023E-02
1.261194E+02 2.937544E-02
4.943525E+01 6.040571E-02
2.027292E+01 4.009023E-02
5.753575E+00 -2.059994E-01
2.177005E+00 -3.990319E-01
3.974195E-01 8.356746E-01
1.100739E-01 3.901580E-01

S 1 1.00
1.100739E-01 1.000000E+00

P 8 1.00
3.791642E+02 4.885410E-03
9.167966E+01 3.518088E-02
3.014854E+01 1.362776E-01
1.139725E+01 3.258048E-01
4.495385E+00 4.489005E-01
1.768319E+00 2.408489E-01
5.459110E-01 1.419538E-02
1.749347E-01 -3.309003E-04

P 8 1.00
3.791642E+02 -1.268297E-03
9.167966E+01 -9.017597E-03
3.014854E+01 -3.769274E-02
1.139725E+01 -9.033206E-02
4.495385E+00 -1.371273E-01
1.768319E+00 -8.928536E-03
5.459110E-01 4.904210E-01
1.749347E-01 5.987739E-01

P 1 1.00
1.749347E-01 1.000000E+00

D   1   1.00

1.736568E+00   1.000000E+00

D   1   1.00

4.880816E-01   1.000000E+00

****

pecG-1 for Cl

Cl   0

S   12   1.00

1.337206E+05	2.313500E-04
1.984004E+04	1.782418E-03
4.541401E+03	9.207895E-03
1.279009E+03	3.787280E-02
4.136238E+02	1.235623E-01
1.476423E+02	2.981197E-01
5.702050E+01	4.204364E-01
2.317175E+01	2.409860E-01
6.364597E+00	1.829181E-02
2.412443E+00	-3.199325E-03
4.376558E-01	6.657590E-04
1.112330E-01	-2.456593E-04

S   12   1.00

1.337206E+05	-6.474912E-05
1.984004E+04	-5.015209E-04
4.541401E+03	-2.629388E-03
1.279009E+03	-1.076870E-02
4.136238E+02	-3.787320E-02
1.476423E+02	-9.938490E-02
5.702050E+01	-2.014192E-01
2.317175E+01	-1.258364E-01
6.364597E+00	5.428079E-01

2.412443E+00 5.855352E-01
4.376558E-01 2.565960E-02
1.112330E-01 -4.408084E-03
S 12 1.00
1.337206E+05 1.949836E-05
1.984004E+04 1.511238E-04
4.541401E+03 7.948204E-04
1.279009E+03 3.335115E-03
4.136238E+02 1.140596E-02
1.476423E+02 3.118904E-02
5.702050E+01 6.285062E-02
2.317175E+01 4.631295E-02
6.364597E+00 -2.522715E-01
2.412443E+00 -3.650333E-01
4.376558E-01 9.322351E-01
1.112330E-01 2.833856E-01
S 1 1.00
1.112330E-01 1.000000E+00
P 8 1.00
4.249864E+02 5.072620E-03
1.022170E+02 3.739230E-02
3.258414E+01 1.525374E-01
1.198047E+01 3.572361E-01
4.707612E+00 4.430563E-01
1.854449E+00 1.945424E-01
5.961951E-01 4.951847E-03
2.061989E-01 1.008077E-03
P 8 1.00
4.249864E+02 -1.351142E-03
1.022170E+02 -1.022727E-02

3.258414E+01 -4.360900E-02

1.198047E+01 -1.038372E-01

4.707612E+00 -1.456609E-01

1.854449E+00 5.382886E-02

5.961951E-01 5.558390E-01

2.061989E-01 5.827293E-01

P 1 1.00

2.061989E-01 1.000000E+00

D 1 1.00

2.473901E+00 1.000000E+00

D 1 1.00

5.808990E-01 1.000000E+00

****

pecG-2 for H

H 0

S 5 1.00

4.599711E+01 3.348681E-03

6.881382E+00 2.521331E-02

1.559462E+00 1.127840E-01

4.275825E-01 1.352883E-01

1.229409E-01 7.879914E-01

S 1 1.00

4.275825E-01 1.000000E+00

S 1 1.00

1.229409E-01 1.000000E+00

P 1 1.00

2.469925E+00 1.000000E+00

P 1 1.00

7.261691E-01 1.000000E+00

P 1 1.00

2.128443E-01 1.000000E+00

D 1 1.00

1.220191E+00 1.000000E+00

****

pecG-2 for C

C 0

S 10 1.00

8.757205E+03 5.139484E-04

1.326860E+03 3.942490E-03

3.033548E+02 1.998259E-02

8.689093E+01 7.637863E-02

2.849378E+01 2.171664E-01

1.017148E+01 4.287923E-01

3.777179E+00 3.743583E-01

1.246816E+00 5.214200E-02

5.105216E-01 -8.493932E-03

1.849029E-01 2.458428E-03

S 10 1.00

8.757205E+03 -1.136534E-04

1.326860E+03 -8.781482E-04

3.033548E+02 -4.471050E-03

8.689093E+01 -1.780003E-02

2.849378E+01 -5.358961E-02

1.017148E+01 -1.245132E-01

3.777179E+00 -1.815224E-01

1.246816E+00 5.414136E-02

5.105216E-01 5.455031E-01

1.849029E-01 5.171546E-01

S 1 1.00

1.246816E+00 1.000000E+00

S 1 1.00

1.849029E-01 1.000000E+00

P 5 1.00

1.988232E+01 1.292312E-02

4.709002E+00 7.289498E-02

1.322009E+00 2.552811E-01

4.239080E-01 5.032530E-01

1.249000E-01 3.678601E-01

P 1 1.00

4.239080E-01 1.000000E+00

P 1 1.00

1.249000E-01 1.000000E+00

D 1 1.00

2.041172E+00 1.000000E+00

D 1 1.00

6.705304E-01 1.000000E+00

D 1 1.00

2.068041E-01 1.000000E+00

F 1 1.00

9.378990E-01 1.000000E+00

****

pecG-2 for B

B 0

S 10 1.00

6.064453E+03 4.891256E-04

9.148559E+02 3.764374E-03

2.101565E+02 1.909043E-02

6.006938E+01 7.327738E-02

1.968165E+01 2.083731E-01

6.991208E+00 4.083524E-01

2.602587E+00	3.736299E-01
9.207801E-01	6.973631E-02
3.385802E-01	-2.546272E-03
1.237673E-01	7.895423E-04

S 10 1.00

6.064453E+03	-8.472549E-05
9.148559E+02	-6.475426E-04
2.101565E+02	-3.279784E-03
6.006938E+01	-1.292045E-02
1.968165E+01	-3.973836E-02
6.991208E+00	-8.910905E-02
2.602587E+00	-1.321259E-01
9.207801E-01	-3.957847E-02
3.385802E-01	4.079915E-01
1.237673E-01	2.776656E-01

S 1 1.00

9.207801E-01	1.000000E+00

S 1 1.00

1.237673E-01	1.000000E+00

P 5 1.00

1.326425E+01	7.918269E-03
3.162231E+00	4.291723E-02
8.639470E-01	1.583641E-01
2.760646E-01	3.209049E-01
8.257178E-02	1.300808E-01

P 1 1.00

2.760646E-01	1.000000E+00

P 1 1.00

8.257178E-02	1.000000E+00

D 1 1.00

1.347953E+00 1.000000E+00

D 1 1.00

4.186305E-01 1.000000E+00

D 1 1.00

1.290718E-01 1.000000E+00

F 1 1.00

5.774737E-01 1.000000E+00

****

pecG-2 for O

O 0

S 10 1.00

1.532828E+04 5.104141E-04

2.289902E+03 4.021769E-03

5.174770E+02 2.059899E-02

1.470943E+02 7.944272E-02

4.797002E+01 2.266328E-01

1.718093E+01 4.263798E-01

6.306310E+00 3.597604E-01

1.852502E+00 4.396196E-02

6.949805E-01 -5.305331E-03

2.015742E-01 1.633684E-03

S 10 1.00

1.532828E+04 -1.186568E-04

2.289902E+03 -9.367625E-04

5.174770E+02 -4.758611E-03

1.470943E+02 -1.919777E-02

4.797002E+01 -5.878630E-02

1.718093E+01 -1.388649E-01

6.306310E+00 -1.836396E-01

1.852502E+00 1.272338E-01

6.949805E-01 7.075278E-01

2.015742E-01 3.049130E-01

S 1 1.00

1.852502E+00 1.000000E+00

S 1 1.00

2.015742E-01 1.000000E+00

P 5 1.00

3.331631E+01 1.745153E-02

7.693515E+00 1.022618E-01

2.270698E+00 3.032953E-01

7.346163E-01 5.007657E-01

2.079195E-01 3.157323E-01

P 1 1.00

7.346163E-01 1.000000E+00

P 1 1.00

2.079195E-01 1.000000E+00

D 1 1.00

5.137776E+00 1.000000E+00

D 1 1.00

1.237850E+00 1.000000E+00

D 1 1.00

3.410607E-01 1.000000E+00

F 1 1.00

1.500525E+00 1.000000E+00

****

pecG-2 for N

N 0

S 10 1.00

1.155668E+04 5.110734E-04

1.705988E+03 4.043512E-03

3.903394E+02 2.055156E-02
1.103356E+02 8.011582E-02
3.577422E+01 2.277878E-01
1.270419E+01 4.268538E-01
4.617906E+00 3.487164E-01
1.285102E+00 3.956177E-02
5.196358E-01 -6.437849E-03
1.494185E-01 1.564056E-03

S 10 1.00

1.155668E+04 -1.133013E-04
1.705988E+03 -9.117380E-04
3.903394E+02 -4.622593E-03
1.103356E+02 -1.869795E-02
3.577422E+01 -5.828416E-02
1.270419E+01 -1.346804E-01
4.617906E+00 -1.823357E-01
1.285102E+00 1.189462E-01
5.196358E-01 6.623345E-01
1.494185E-01 3.266679E-01

S 1 1.00

1.285102E+00 1.000000E+00

S 1 1.00

1.494185E-01 1.000000E+00

P 5 1.00

2.354833E+01 1.744842E-02
5.431571E+00 9.927950E-02
1.553404E+00 3.070979E-01
4.915450E-01 5.158355E-01
1.362691E-01 2.880036E-01

P 1 1.00

4.915450E-01 1.000000E+00

P 1 1.00

1.362691E-01 1.000000E+00

D 1 1.00

3.527951E+00 1.000000E+00

D 1 1.00

1.035310E+00 1.000000E+00

D 1 1.00

2.490997E-01 1.000000E+00

F 1 1.00

1.176136E+00 1.000000E+00

****

pecG-2 for F

F 0

S 10 1.00

1.969934E+04 5.104428E-04

2.890541E+03 3.986684E-03

6.606998E+02 2.057457E-02

1.818765E+02 8.374396E-02

5.828637E+01 2.373460E-01

2.088519E+01 4.277700E-01

7.756684E+00 3.446985E-01

2.096912E+00 3.700777E-02

7.921501E-01 -5.478410E-03

2.553894E-01 1.805454E-03

S 10 1.00

1.969934E+04 -1.195323E-04

2.890541E+03 -9.567656E-04

6.606998E+02 -4.939225E-03

1.818765E+02 -2.033464E-02

5.828637E+01 -6.329415E-02
2.088519E+01 -1.454458E-01
7.756684E+00 -1.777993E-01
2.096912E+00 2.168498E-01
7.921501E-01 6.461238E-01
2.553894E-01 2.887376E-01
S 1 1.00
2.096912E+00 1.000000E+00
S 1 1.00
2.553894E-01 1.000000E+00
P 5 1.00
4.266286E+01 1.778138E-02
9.834802E+00 1.048373E-01
2.893537E+00 3.186658E-01
8.758003E-01 5.162679E-01
2.394958E-01 2.924261E-01
P 1 1.00
8.758003E-01 1.000000E+00
P 1 1.00
2.394958E-01 1.000000E+00
D 1 1.00
7.110846E+00 1.000000E+00
D 1 1.00
1.650970E+00 1.000000E+00
D 1 1.00
4.431937E-01 1.000000E+00
F 1 1.00
1.841984E+00 1.000000E+00
****

pecG-2 for Al

Al 0

S 15 1.00

2.060622E+05	6.719909E-05
3.061290E+04	5.355831E-04
6.974484E+03	2.768140E-03
1.977398E+03	1.152600E-02
6.437403E+02	4.034466E-02
2.326996E+02	1.160180E-01
9.037288E+01	2.646256E-01
3.713204E+01	3.913667E-01
1.568155E+01	2.832091E-01
5.951353E+00	4.231603E-02
2.507022E+00	-2.611537E-03
1.021810E+00	1.727821E-03
2.825714E-01	-7.393193E-04
1.488336E-01	5.044898E-04
5.900703E-02	-1.206915E-04

S 15 1.00

2.060622E+05	-1.775096E-05
3.061290E+04	-1.376554E-04
6.974484E+03	-7.250605E-04
1.977398E+03	-3.068563E-03
6.437403E+02	-1.074872E-02
2.326996E+02	-3.288044E-02
9.037288E+01	-8.223587E-02
3.713204E+01	-1.570929E-01
1.568155E+01	-1.675340E-01
5.951353E+00	1.276627E-01
2.507022E+00	5.792274E-01
1.021810E+00	4.283303E-01

2.825714E-01	2.762105E-02
1.488336E-01	-7.250235E-03
5.900703E-02	1.171474E-03

S 15 1.00

2.060622E+05	4.012991E-06
3.061290E+04	3.179322E-05
6.974484E+03	1.613923E-04
1.977398E+03	6.940028E-04
6.437403E+02	2.481580E-03
2.326996E+02	7.472978E-03
9.037288E+01	1.874012E-02
3.713204E+01	3.635347E-02
1.568155E+01	4.033434E-02
5.951353E+00	-3.510296E-02
2.507022E+00	-1.828650E-01
1.021810E+00	-2.588631E-01
2.825714E-01	1.015613E-01
1.488336E-01	6.283527E-01
5.900703E-02	4.292799E-01

S 1 1.00

2.825714E-01	1.000000E+00

S 1 1.00

5.900703E-02	1.000000E+00

P 9 1.00

4.427826E+02	1.638004E-03
1.036560E+02	1.354692E-02
3.338709E+01	6.101323E-02
1.231760E+01	1.865489E-01
4.891972E+00	3.500872E-01
1.984784E+00	3.996111E-01

7.752297E-01 1.912197E-01
2.005694E-01 7.725564E-03
5.968126E-02 -2.617979E-04
P 9 1.00
4.427826E+02 -2.966596E-04
1.036560E+02 -2.513763E-03
3.338709E+01 -1.104732E-02
1.231760E+01 -3.645979E-02
4.891972E+00 -6.659861E-02
1.984784E+00 -8.956453E-02
7.752297E-01 5.115671E-03
2.005694E-01 4.891244E-01
5.968126E-02 6.157183E-01
P 1 1.00
7.752297E-01 1.000000E+00
P 1 1.00
5.968126E-02 1.000000E+00
D 1 1.00
1.107508E+00 1.000000E+00
D 1 1.00
3.109116E-01 1.000000E+00
D 1 1.00
1.116031E-01 1.000000E+00
F 1 1.00
2.577153E-01 1.000000E+00
****

pecG-2 for Si

Si 0
S 15 1.00
2.596413E+05 6.119909E-05

3.898958E+04	4.755370E-04
8.847420E+03	2.484803E-03
2.487125E+03	1.047556E-02
8.087775E+02	3.667182E-02
2.917459E+02	1.073011E-01
1.129780E+02	2.499411E-01
4.639935E+01	3.856206E-01
1.966803E+01	3.012889E-01
7.817117E+00	5.482195E-02
3.276549E+00	-2.107659E-03
1.350290E+00	1.834907E-03
4.226587E-01	-7.839259E-04
2.144015E-01	4.641237E-04
8.363757E-02	-1.154299E-04

S 15 1.00

2.596413E+05	-1.628722E-05
3.898958E+04	-1.267753E-04
8.847420E+03	-6.658054E-04
2.487125E+03	-2.836684E-03
8.087775E+02	-1.006734E-02
2.917459E+02	-3.085673E-02
1.129780E+02	-7.943893E-02
4.639935E+01	-1.542435E-01
1.966803E+01	-1.818137E-01
7.817117E+00	8.339699E-02
3.276549E+00	5.714203E-01
1.350290E+00	4.706354E-01
4.226587E-01	3.734001E-02
2.144015E-01	-6.134327E-03
8.363757E-02	1.242562E-03

S 15 1.00

2.596413E+05 4.164783E-06

3.898958E+04 3.186341E-05

8.847420E+03 1.700275E-04

2.487125E+03 7.226248E-04

8.087775E+02 2.551676E-03

2.917459E+02 7.893035E-03

1.129780E+02 2.027595E-02

4.639935E+01 4.085706E-02

1.966803E+01 4.829946E-02

7.817117E+00 -2.459209E-02

3.276549E+00 -2.022944E-01

1.350290E+00 -3.054854E-01

4.226587E-01 7.276532E-02

2.144015E-01 6.742951E-01

8.363757E-02 4.231524E-01

S 1 1.00

4.226587E-01 1.000000E+00

S 1 1.00

8.363757E-02 1.000000E+00

P 9 1.00

4.768263E+02 1.981018E-03

1.117954E+02 1.621360E-02

3.591036E+01 7.252661E-02

1.324922E+01 2.159617E-01

5.218687E+00 3.876229E-01

2.134711E+00 3.842038E-01

8.492718E-01 1.371036E-01

2.746023E-01 2.125885E-03

8.563368E-02 9.880130E-04

P 9 1.00

4.768263E+02 -4.258546E-04

1.117954E+02 -3.478933E-03

3.591036E+01 -1.565422E-02

1.324922E+01 -4.939537E-02

5.218687E+00 -8.896064E-02

2.134711E+00 -9.741856E-02

8.492718E-01 4.264965E-02

2.746023E-01 5.202745E-01

8.563368E-02 5.749857E-01

P 1 1.00

8.492718E-01 1.000000E+00

P 1 1.00

8.563368E-02 1.000000E+00

D 1 1.00

1.900106E+00 1.000000E+00

D 1 1.00

4.746225E-01 1.000000E+00

D 1 1.00

1.715561E-01 1.000000E+00

F 1 1.00

4.216546E-01 1.000000E+00

****

pecG-2 for P (Proper citation for this basis set: *J. Chem. Phys.* 160, 084109 (2024). DOI: 10.1063/5.0193227)

P 0

S 15 1.00

3.045367E+05 5.696072E-05

4.685982E+04 4.246568E-04

1.091974E+04 2.255890E-03

3.150159E+03 8.849759E-03

1.062425E+03	3.175938E-02
3.550266E+02	1.124466E-01
1.265555E+02	2.553633E-01
5.520377E+01	3.370352E-01
2.509589E+01	3.121828E-01
1.072175E+01	7.595532E-02
4.032690E+00	-8.548371E-05
1.687462E+00	1.316239E-03
6.392740E-01	-3.657178E-04
2.593587E-01	1.313312E-04
9.231337E-02	-3.855570E-05

S 15 1.00

3.045367E+05	-1.578944E-05
4.685982E+04	-1.140663E-04
1.091974E+04	-6.233031E-04
3.150159E+03	-2.447577E-03
1.062425E+03	-8.687315E-03
3.550266E+02	-3.302503E-02
1.265555E+02	-8.162952E-02
5.520377E+01	-1.352949E-01
2.509589E+01	-1.853864E-01
1.072175E+01	4.276975E-02
4.032690E+00	5.585780E-01
1.687462E+00	4.793466E-01
6.392740E-01	6.130200E-02
2.593587E-01	-5.985775E-03
9.231337E-02	1.745025E-03

S 15 1.00

3.045367E+05	4.163705E-06
4.685982E+04	3.084029E-05

1.091974E+04	1.697176E-04
3.150159E+03	6.596316E-04
1.062425E+03	2.365662E-03
3.550266E+02	8.884083E-03
1.265555E+02	2.253960E-02
5.520377E+01	3.594466E-02
2.509589E+01	5.356072E-02
1.072175E+01	-1.537886E-02
4.032690E+00	-1.943992E-01
1.687462E+00	-3.361408E-01
6.392740E-01	9.140270E-02
2.593587E-01	6.916086E-01
9.231337E-02	4.092180E-01

S 1 1.00

6.392740E-01	1.000000E+00

S 1 1.00

9.231337E-02	1.000000E+00

P 9 1.00

4.638615E+02	2.657715E-03
1.118096E+02	2.083077E-02
3.612305E+01	8.938175E-02
1.318119E+01	2.719074E-01
4.875632E+00	4.518999E-01
1.866572E+00	3.362361E-01
6.002790E-01	4.827537E-02
2.208753E-01	-6.561835E-03
7.695694E-02	2.080827E-03

P 9 1.00

4.638615E+02	-5.607912E-04
1.118096E+02	-4.327121E-03

3.612305E+01 -1.922648E-02

1.318119E+01 -6.056502E-02

4.875632E+00 -1.052393E-01

1.866572E+00 -7.030646E-02

6.002790E-01 2.509121E-01

2.208753E-01 4.210820E-01

7.695694E-02 5.029072E-01

P 1 1.00

6.002790E-01 1.000000E+00

P 1 1.00

7.695694E-02 1.000000E+00

D 1 1.00

3.157596E+00 1.000000E+00

D 1 1.00

6.526927E-01 1.000000E+00

D 1 1.00

2.084180E-01 1.000000E+00

F 1 1.00

5.947649E-01 1.000000E+00

****

pecG-2 for S

S 0

S 15 1.00

3.834743E+05 5.430235E-05

5.410085E+04 4.486598E-04

1.211729E+04 2.366015E-03

3.446410E+03 9.640368E-03

1.144730E+03 3.299293E-02

4.207364E+02 9.567473E-02

1.654608E+02 2.256901E-01

6.896900E+01	3.697443E-01
2.957085E+01	3.325950E-01
1.245821E+01	8.267930E-02
5.082521E+00	1.061511E-04
2.124538E+00	1.617944E-03
7.788963E-01	-6.516450E-04
3.458198E-01	2.623479E-04
1.183089E-01	-7.196217E-05

S 15 1.00

3.834743E+05	-1.493426E-05
5.410085E+04	-1.235321E-04
1.211729E+04	-6.515876E-04
3.446410E+03	-2.691159E-03
1.144730E+03	-9.301602E-03
4.207364E+02	-2.820562E-02
1.654608E+02	-7.312290E-02
6.896900E+01	-1.453650E-01
2.957085E+01	-1.973443E-01
1.245821E+01	1.697975E-02
5.082521E+00	5.408279E-01
2.124538E+00	5.258636E-01
7.788963E-01	5.397465E-02
3.458198E-01	-1.456241E-03
1.183089E-01	7.801927E-04

S 15 1.00

3.834743E+05	4.329743E-06
5.410085E+04	3.625018E-05
1.211729E+04	1.898347E-04
3.446410E+03	7.848197E-04
1.144730E+03	2.694930E-03

	4.207364E+02	8.256958E-03
	1.654608E+02	2.121777E-02
	6.896900E+01	4.380096E-02
	2.957085E+01	5.951403E-02
	1.245821E+01	-3.891435E-03
	5.082521E+00	-2.250525E-01
	2.124538E+00	-3.578330E-01
	7.788963E-01	5.808424E-02
	3.458198E-01	8.065237E-01
	1.183089E-01	3.276095E-01
S	1 1.00	
	7.788963E-01	1.000000E+00
S	1 1.00	
	1.183089E-01	1.000000E+00
P	9 1.00	
	5.635566E+02	2.520497E-03
	1.330427E+02	2.034629E-02
	4.244063E+01	9.098774E-02
	1.562430E+01	2.586518E-01
	6.082340E+00	4.399830E-01
	2.398878E+00	3.446723E-01
	8.087534E-01	5.513249E-02
	3.291157E-01	-5.932249E-03
	1.130662E-01	1.702035E-03
P	9 1.00	
	5.635566E+02	-6.522143E-04
	1.330427E+02	-5.334838E-03
	4.244063E+01	-2.453725E-02
	1.562430E+01	-7.231182E-02
	6.082340E+00	-1.308281E-01

2.398878E+00 -7.962598E-02
8.087534E-01 2.340294E-01
3.291157E-01 5.506658E-01
1.130662E-01 3.565693E-01
P 1 1.00
8.087534E-01 1.000000E+00
P 1 1.00
1.130662E-01 1.000000E+00
D 1 1.00
3.285035E+00 1.000000E+00
D 1 1.00
7.459837E-01 1.000000E+00
D 1 1.00
2.698783E-01 1.000000E+00
F 1 1.00
6.707579E-01 1.000000E+00
****

pecG-2 for Cl

Cl 0
S 15 1.00
4.636925E+05 4.816224E-05
6.812223E+04 3.817724E-04
1.559421E+04 1.995454E-03
4.281796E+03 8.756239E-03
1.400457E+03 2.962860E-02
5.149020E+02 8.692990E-02
2.014161E+02 2.108039E-01
8.337909E+01 3.603683E-01
3.577536E+01 3.365392E-01
1.575939E+01 1.000224E-01

6.197023E+00	3.784080E-03
2.619565E+00	5.808632E-04
9.292480E-01	-1.756602E-04
4.078138E-01	3.819567E-05
1.445756E-01	-2.205453E-05

S 15 1.00

4.636925E+05	-1.350240E-05
6.812223E+04	-1.079799E-04
1.559421E+04	-5.686584E-04
4.281796E+03	-2.454718E-03
1.400457E+03	-8.682288E-03
5.149020E+02	-2.600840E-02
2.014161E+02	-6.991550E-02
8.337909E+01	-1.425865E-01
3.577536E+01	-2.008537E-01
1.575939E+01	-1.501063E-02
6.197023E+00	5.227899E-01
2.619565E+00	5.594012E-01
9.292480E-01	6.414773E-02
4.078138E-01	-3.933715E-03
1.445756E-01	1.317544E-03

S 15 1.00

4.636925E+05	3.943688E-06
6.812223E+04	3.294708E-05
1.559421E+04	1.729757E-04
4.281796E+03	7.287624E-04
1.400457E+03	2.651901E-03
5.149020E+02	8.017662E-03
2.014161E+02	2.111443E-02
8.337909E+01	4.517979E-02

3.577536E+01 6.419204E-02
1.575939E+01 6.170535E-03
6.197023E+00 -2.252473E-01
2.619565E+00 -3.940855E-01
9.292480E-01 9.590277E-02
4.078138E-01 7.889166E-01
1.445756E-01 3.308177E-01
S 1 1.00
9.292480E-01 1.000000E+00
S 1 1.00
1.445756E-01 1.000000E+00
P 9 1.00
6.622090E+02 2.426739E-03
1.587259E+02 1.943533E-02
5.123106E+01 8.672206E-02
1.900856E+01 2.533043E-01
7.410545E+00 4.489957E-01
2.956015E+00 3.552635E-01
1.075845E+00 6.016968E-02
4.097916E-01 -4.187396E-03
1.353053E-01 1.363768E-03
P 9 1.00
6.622090E+02 -6.507195E-04
1.587259E+02 -4.845570E-03
5.123106E+01 -2.108831E-02
1.900856E+01 -6.552273E-02
7.410545E+00 -1.220786E-01
2.956015E+00 -1.066861E-01
1.075845E+00 2.189891E-01
4.097916E-01 5.010555E-01

1.353053E-01 3.949247E-01

P 1 1.00

1.075845E+00 1.000000E+00

P 1 1.00

1.353053E-01 1.000000E+00

D 1 1.00

3.888140E+00 1.000000E+00

D 1 1.00

9.491956E-01 1.000000E+00

D 1 1.00

3.741550E-01 1.000000E+00

F 1 1.00

7.399645E-01 1.000000E+00

****

Equilibrium geometries of molecules from set **1** calculated at the **DFT(M06-2X)** level of theory with **6-31G(2d,2p)** basis set within the IEF-PCM model. Cartesian coordinates are given for standard orientation in form (atomic label, X, Y, Z). The X, Y, Z are given in Å.

12-28-oxaircinal (*E* = -1271.8982918 Hartree), solvent: Chloroform

O	1.591417000	-0.781026000	1.828519000
O	-0.212147000	4.731885000	1.485805000
N	1.766154000	-0.503682000	-0.459019000
N	-2.178128000	0.473694000	-1.532012000
C	0.069567000	1.170013000	-0.862269000
C	0.392719000	-0.132838000	-0.099080000
C	0.398968000	-0.090881000	1.456692000
C	-0.582607000	2.288537000	-0.013379000
C	1.459189000	1.617026000	-1.381222000
C	2.489947000	0.764593000	-0.626272000
C	-0.855293000	0.805828000	-2.029115000

C	2.175063000	-1.349022000	0.645667000
C	-2.107937000	2.170103000	0.183844000
C	0.079686000	2.387136000	1.332165000
C	0.508821000	1.307510000	1.992747000
C	-0.851445000	-0.793437000	2.015295000
C	-2.825768000	1.691338000	-1.068851000
C	3.664675000	-1.508271000	0.864986000
C	3.798815000	0.695619000	-1.360374000
C	-2.965510000	-0.317847000	-2.463394000
C	-0.833897000	-2.318258000	1.823513000
C	4.353102000	-2.331167000	-0.224051000
C	0.194103000	3.705393000	1.977795000
C	4.472213000	-0.360897000	-1.811605000
C	4.105318000	-1.814795000	-1.652641000
C	-4.281458000	-0.819142000	-1.856878000
C	-2.191591000	-2.922390000	2.073784000
C	-4.167664000	-1.335632000	-0.420575000
C	-3.152053000	-2.468962000	-0.231488000
C	-3.177056000	-2.989275000	1.179016000
H	-0.295265000	-0.920270000	-0.411906000
H	-0.410370000	3.229310000	-0.546693000
H	1.638699000	2.684223000	-1.241448000
H	1.541797000	1.399949000	-2.450373000
H	2.677570000	1.238657000	0.352606000
H	-0.891693000	1.639205000	-2.754927000
H	-0.431627000	-0.060251000	-2.547658000
H	1.723379000	-2.338292000	0.480825000
H	-2.487233000	3.144350000	0.502736000
H	-2.322976000	1.464945000	0.989374000
H	0.959444000	1.404177000	2.978264000

H	-1.717772000	-0.389089000	1.483072000
H	-0.981432000	-0.556473000	3.075474000
H	-3.870723000	1.488288000	-0.831005000
H	-2.812857000	2.469569000	-1.853259000
H	3.791163000	-2.003518000	1.830425000
H	4.121879000	-0.520343000	0.953498000
H	4.229753000	1.680120000	-1.531111000
H	-2.348178000	-1.177414000	-2.743477000
H	-3.182824000	0.232389000	-3.395552000
H	-0.089512000	-2.754448000	2.492244000
H	-0.516177000	-2.558417000	0.806618000
H	4.012670000	-3.369696000	-0.164915000
H	5.427349000	-2.341284000	-0.020395000
H	0.686053000	3.701237000	2.969712000
H	5.414470000	-0.158767000	-2.313162000
H	3.057207000	-1.964542000	-1.908625000
H	4.699232000	-2.412936000	-2.346575000
H	-4.660591000	-1.615395000	-2.505300000
H	-5.033622000	-0.025908000	-1.887441000
H	-2.378454000	-3.302734000	3.073795000
H	-5.154064000	-1.681383000	-0.094872000
H	-3.892056000	-0.513520000	0.248198000
H	-2.157673000	-2.100584000	-0.494603000
H	-3.392757000	-3.281713000	-0.927946000
H	-4.122584000	-3.428087000	1.491181000

Anabsinthin (Conformer 1, E = -1618.1404934 Hartree), solvent: Acetonitrile

O	-2.291884000	1.202575000	-2.211006000
O	-2.559039000	-1.938435000	0.609870000
O	2.507369000	0.069862000	3.076389000

O	3.027736000	-1.664077000	-1.198740000
O	-3.771118000	-3.388574000	1.786355000
O	4.462368000	-2.153187000	-2.832309000
C	-1.635449000	0.181802000	-0.232098000
C	-1.962878000	1.680424000	0.040594000
C	-0.481289000	2.133172000	-0.015779000
C	-1.136119000	0.534266000	-1.670356000
C	-0.531600000	-0.124464000	0.813349000
C	0.148410000	1.246496000	1.071633000
C	-2.773863000	2.113950000	-1.196298000
C	-0.059365000	1.603059000	-1.406565000
C	-2.801809000	-0.809039000	-0.262311000
C	1.678579000	1.034367000	0.964381000
C	0.601744000	-1.031962000	0.415216000
C	-4.175572000	-0.296497000	0.166951000
C	-4.294403000	1.953762000	-1.031436000
C	1.775323000	-0.389501000	0.445778000
C	-0.710689000	-0.544355000	-2.636221000
C	-4.843581000	0.535323000	-0.915090000
C	2.510220000	1.245787000	2.272518000
C	-2.526843000	3.564438000	-1.616492000
C	-4.877230000	-1.606686000	0.523270000
C	3.088771000	-1.017675000	0.101504000
C	0.374098000	-2.479336000	0.111954000
C	3.989644000	1.530709000	1.962842000
C	-3.726831000	-2.434426000	1.061467000
C	4.276178000	-0.067597000	-0.018079000
C	4.806303000	0.407097000	1.325291000
C	1.975540000	2.430845000	3.082801000
C	-6.045590000	-1.526406000	1.489769000

C	5.226843000	-0.912561000	-0.865141000
C	4.251498000	-1.650678000	-1.764334000
C	6.299291000	-0.176428000	-1.647898000
H	-2.445872000	1.900949000	0.993412000
H	-0.304700000	3.197695000	0.138053000
H	-1.026535000	-0.518174000	1.705675000
H	-0.145542000	1.635714000	2.044792000
H	0.941628000	1.173133000	-1.413095000
H	-0.103375000	2.353208000	-2.195444000
H	-2.902098000	-1.217695000	-1.273416000
H	2.084364000	1.754459000	0.242769000
H	-4.065651000	0.291086000	1.087829000
H	-4.778111000	2.439123000	-1.884306000
H	-4.582932000	2.530148000	-0.145876000
H	-1.472543000	-1.315374000	-2.763447000
H	-0.528551000	-0.089460000	-3.612527000
H	0.217410000	-1.007478000	-2.297628000
H	-4.731400000	0.015278000	-1.871846000
H	-5.916670000	0.600191000	-0.714102000
H	-3.009833000	3.748843000	-2.577890000
H	-2.966158000	4.241902000	-0.880056000
H	-1.470973000	3.806109000	-1.712136000
H	-5.190775000	-2.098930000	-0.408119000
H	3.332595000	-1.799240000	0.832955000
H	-0.319238000	-2.602910000	-0.724728000
H	1.300591000	-2.998427000	-0.127195000
H	-0.102037000	-2.959428000	0.970832000
H	4.470351000	1.796279000	2.908479000
H	4.032877000	2.422135000	1.328325000
H	3.968184000	0.795230000	-0.624337000

H	4.858901000	-0.445590000	2.009679000
H	5.829666000	0.771051000	1.195303000
H	1.026092000	2.188605000	3.560646000
H	1.826930000	3.307591000	2.446695000
H	2.684876000	2.688709000	3.870587000
H	-5.742731000	-1.032939000	2.415548000
H	-6.402138000	-2.526823000	1.736374000
H	-6.870159000	-0.962424000	1.052207000
H	1.594883000	-0.222893000	3.177487000
H	5.685070000	-1.676001000	-0.220946000
H	6.840575000	-0.870792000	-2.291032000
H	7.012206000	0.299510000	-0.973621000
H	5.851401000	0.595004000	-2.277689000

Anabsinthin (Conformer 2, *E* = -1618.1406789 Hartree), **solvent: Acetonitrile**

O	-2.171354000	2.390669000	-0.857655000
O	-2.593955000	-1.816022000	-0.826964000
O	1.529264000	-1.943709000	2.873656000
O	3.025956000	-0.324090000	-2.027888000
O	-3.872886000	-3.636787000	-0.902107000
O	4.437894000	0.638723000	-3.459332000
C	-1.589086000	0.292472000	-0.053665000
C	-1.872201000	1.248835000	1.143500000
C	-0.375012000	1.570314000	1.389927000
C	-1.047609000	1.489808000	-0.903105000
C	-0.521646000	-0.673134000	0.523941000
C	0.197171000	0.165210000	1.619822000
C	-2.644821000	2.420320000	0.508474000
C	0.057453000	2.077730000	-0.005455000

C	-2.785391000	-0.384646000	-0.726465000
C	1.709183000	-0.016458000	1.385742000
C	0.584000000	-1.120245000	-0.402785000
C	-4.149847000	-0.237070000	-0.055328000
C	-4.172626000	2.246531000	0.536182000
C	1.775184000	-0.691327000	0.031432000
C	-0.642532000	1.312845000	-2.346246000
C	-4.770068000	1.126625000	-0.310271000
C	2.390802000	-0.873062000	2.489313000
C	-2.344475000	3.775984000	1.151493000
C	-4.898833000	-1.428158000	-0.652114000
C	3.089338000	-0.849222000	-0.677014000
C	0.320773000	-1.950776000	-1.618292000
C	3.717375000	-1.509576000	2.028122000
C	-3.784922000	-2.444531000	-0.809604000
C	4.223499000	-0.051396000	-0.035009000
C	4.740968000	-0.643062000	1.281107000
C	2.590788000	-0.031609000	3.741045000
C	-6.079439000	-1.968928000	0.134889000
C	5.197650000	0.055288000	-1.204552000
C	4.233491000	0.174014000	-2.374106000
C	6.211825000	1.184041000	-1.160971000
H	-2.364567000	0.801921000	2.008158000
H	-0.168546000	2.259958000	2.208370000
H	-1.045454000	-1.539013000	0.937707000
H	-0.082796000	-0.183188000	2.613783000
H	1.046491000	1.725755000	-0.298268000
H	0.049959000	3.163010000	-0.101525000
H	-2.880140000	-0.014669000	-1.752887000
H	2.214427000	0.955398000	1.380672000

H	-4.038484000	-0.411710000	1.022793000
H	-4.624594000	3.191595000	0.220672000
H	-4.459194000	2.104111000	1.583687000
H	-0.451811000	2.298165000	-2.777622000
H	0.276267000	0.728678000	-2.413148000
H	-1.420062000	0.832046000	-2.942525000
H	-4.657894000	1.365648000	-1.372728000
H	-5.843950000	1.081880000	-0.107861000
H	-2.775618000	3.811974000	2.155049000
H	-1.279877000	3.982148000	1.232348000
H	-2.803368000	4.567497000	0.556064000
H	-5.212711000	-1.167271000	-1.672627000
H	3.370857000	-1.905682000	-0.768370000
H	-0.135458000	-2.899360000	-1.323379000
H	-0.397412000	-1.463809000	-2.282924000
H	1.234890000	-2.146801000	-2.178142000
H	3.458140000	-2.362471000	1.391294000
H	4.194514000	-1.938655000	2.913266000
H	3.834803000	0.957720000	0.139486000
H	5.605555000	-1.279088000	1.066954000
H	5.106553000	0.162217000	1.923429000
H	2.922099000	-0.664975000	4.565764000
H	1.658607000	0.458426000	4.030902000
H	3.343967000	0.739000000	3.565653000
H	-6.469589000	-2.869615000	-0.339817000
H	-6.879501000	-1.229594000	0.187083000
H	-5.776404000	-2.222244000	1.152916000
H	1.339358000	-2.457414000	2.078676000
H	5.709554000	-0.908913000	-1.329067000
H	6.914968000	1.034130000	-0.340673000

H	5.710203000	2.142988000	-1.014965000
H	6.770922000	1.229025000	-2.095908000

Betulinic acid (*E* = -1397.3135817 Hartree), solvent: Pyridine

C	4.188671000	1.198931000	0.354691000
C	3.159431000	0.291390000	-0.354513000
C	3.791190000	-1.109700000	-0.370436000
C	5.248039000	-0.776302000	-0.740066000
C	5.555947000	0.476280000	0.103138000
C	1.716979000	0.264065000	0.143315000
C	0.840565000	-0.708029000	-0.705351000
C	1.541954000	-2.082600000	-0.876284000
C	3.007788000	-1.999522000	-1.331533000
C	1.070448000	1.647234000	0.212989000
C	-0.365011000	1.593538000	0.750128000
C	-1.248496000	0.534554000	0.069127000
C	-0.549142000	-0.861185000	0.049373000
C	-2.738007000	0.549229000	0.558263000
C	-3.505728000	-0.454566000	-0.352035000
C	-2.907333000	-1.857345000	-0.231938000
C	-1.447343000	-1.874931000	-0.685098000
C	-3.308698000	1.959912000	0.296347000
C	-4.829677000	2.028360000	0.396772000
C	-5.501173000	1.037908000	-0.535614000
C	-5.058496000	-0.420335000	-0.279556000
C	4.227850000	2.632823000	-0.121469000
C	4.261110000	3.643903000	0.744031000
C	4.286040000	2.870945000	-1.607178000
C	3.840004000	-1.745342000	1.013158000
O	3.716372000	-1.181701000	2.072031000

O	4.110665000	-3.060260000	0.947245000
C	0.662825000	-0.154629000	-2.137513000
C	-0.325016000	-1.391509000	1.485664000
C	-2.877140000	0.257439000	2.067300000
C	-5.632484000	-1.281936000	-1.416722000
C	-5.646967000	-0.947153000	1.035176000
O	-6.900723000	1.220854000	-0.383658000
H	3.982109000	1.200108000	1.426042000
H	3.155106000	0.591544000	-1.409036000
H	5.275059000	-0.542346000	-1.808543000
H	5.939803000	-1.601072000	-0.560579000
H	6.276311000	1.129886000	-0.391116000
H	5.990192000	0.189354000	1.062071000
H	1.755362000	-0.128742000	1.163233000
H	0.979307000	-2.679197000	-1.599223000
H	1.526533000	-2.648959000	0.058680000
H	3.087603000	-1.570329000	-2.334120000
H	3.435709000	-3.001934000	-1.378331000
H	1.086172000	2.114765000	-0.777938000
H	1.667969000	2.296135000	0.857852000
H	-0.816225000	2.580379000	0.631636000
H	-0.317508000	1.412285000	1.827672000
H	-1.338519000	0.834620000	-0.980311000
H	-3.279137000	-0.117386000	-1.377377000
H	-3.462851000	-2.561887000	-0.853644000
H	-2.999586000	-2.226175000	0.793401000
H	-1.438607000	-1.668896000	-1.759161000
H	-1.037990000	-2.881493000	-0.554216000
H	-3.006183000	2.282964000	-0.708052000
H	-2.875869000	2.674171000	1.000950000

H	-5.175021000	3.033447000	0.142263000
H	-5.169437000	1.837199000	1.419013000
H	-5.204296000	1.287714000	-1.567240000
H	4.339701000	4.672874000	0.412030000
H	4.206299000	3.469989000	1.812993000
H	3.316658000	2.672808000	-2.074363000
H	4.562375000	3.901369000	-1.830240000
H	5.009426000	2.204559000	-2.088062000
H	4.175479000	-3.381764000	1.857740000
H	0.184822000	0.820665000	-2.179818000
H	1.629081000	-0.048739000	-2.631815000
H	0.076640000	-0.839260000	-2.751828000
H	-1.212027000	-1.906620000	1.851426000
H	0.493853000	-2.109542000	1.528225000
H	-0.092749000	-0.604714000	2.201657000
H	-2.010792000	0.627248000	2.616540000
H	-3.746717000	0.764222000	2.485411000
H	-2.983673000	-0.802557000	2.294470000
H	-5.171840000	-1.032134000	-2.376785000
H	-5.486496000	-2.347042000	-1.232831000
H	-6.712105000	-1.133132000	-1.505836000
H	-6.733431000	-1.005695000	0.947369000
H	-5.270791000	-1.950651000	1.248668000
H	-5.422064000	-0.313430000	1.890906000
H	-7.346955000	0.745664000	-1.089725000

Icajine (Conformer 1, *E* = -1187.5663057 Hartree), solvent: Chloroform

O	-0.132853000	-2.877580000	1.208602000
O	2.077037000	3.020692000	0.723198000
O	-2.540816000	3.194359000	-0.430717000

N	-1.446206000	1.195292000	-0.504274000
N	2.100458000	-2.078695000	-0.560696000
C	-0.153145000	0.518646000	-0.314567000
C	0.209382000	-1.741155000	0.962886000
C	1.375852000	-1.727299000	-1.772480000
C	-0.131647000	-1.754389000	-1.502434000
C	-0.485749000	-0.993993000	-0.203046000
C	-1.995251000	-0.977118000	-0.019304000
C	-2.854478000	-2.023998000	0.265377000
C	-4.226201000	-1.775530000	0.318479000
C	-4.718711000	-0.497175000	0.079732000
C	-3.866270000	0.566126000	-0.213172000
C	-2.503235000	0.302877000	-0.252531000
C	1.202482000	-1.026557000	1.868083000
C	1.743097000	0.299517000	1.317140000
C	0.502408000	1.110296000	0.929187000
C	0.718622000	2.633810000	0.775852000
C	2.836706000	2.660750000	-0.405949000
C	3.160188000	1.203981000	-0.561050000
C	2.732301000	0.165867000	0.156701000
C	3.217893000	-1.220105000	-0.204646000
C	-0.125809000	3.201126000	-0.384493000
C	-1.502725000	2.562513000	-0.438449000
C	2.347403000	-3.496154000	-0.415199000
H	0.475677000	0.704369000	-1.186391000
H	1.624448000	-2.405033000	-2.599446000
H	1.684544000	-0.727911000	-2.085726000
H	-0.478781000	-2.783857000	-1.378140000
H	-0.681833000	-1.321566000	-2.342731000
H	-2.453643000	-3.011020000	0.452751000

H	-4.909968000	-2.583894000	0.543591000
H	-5.785957000	-0.317678000	0.120578000
H	-4.236872000	1.562013000	-0.396632000
H	1.983433000	-1.747425000	2.115205000
H	0.675253000	-0.812736000	2.804180000
H	2.255316000	0.819849000	2.131083000
H	-0.231538000	0.983282000	1.734929000
H	0.371405000	3.107738000	1.695053000
H	3.775390000	3.213813000	-0.305358000
H	2.377902000	3.027652000	-1.334421000
H	3.850543000	1.007732000	-1.379106000
H	3.707573000	-1.676229000	0.662834000
H	3.975972000	-1.153797000	-0.999328000
H	-0.247106000	4.278172000	-0.295564000
H	0.363015000	2.987752000	-1.339211000
H	3.086274000	-3.875233000	-1.135792000
H	1.410280000	-4.040379000	-0.549701000
H	2.703295000	-3.703781000	0.596770000

Icajine (Conformer 2, *E* = -1187.5668475 Hartree), solvent: Chloroform

O	-0.032211000	-2.780713000	1.305707000
O	1.592883000	2.919605000	-0.589125000
O	-2.887291000	3.000518000	-0.211842000
N	-1.545577000	1.178224000	-0.361813000
N	2.202753000	-2.013834000	-0.479860000
C	-0.195691000	0.583704000	-0.283147000
C	0.277597000	-1.645603000	1.017806000
C	1.507048000	-1.632874000	-1.698418000
C	0.000240000	-1.700083000	-1.463846000
C	-0.426213000	-0.954748000	-0.175052000

C	-1.935313000	-1.061777000	-0.057423000
C	-2.712226000	-2.192628000	0.126164000
C	-4.100871000	-2.066431000	0.116690000
C	-4.688697000	-0.823357000	-0.088083000
C	-3.918328000	0.322378000	-0.278806000
C	-2.535477000	0.181547000	-0.250546000
C	1.243605000	-0.867380000	1.900191000
C	1.791833000	0.428283000	1.284091000
C	0.551056000	1.237324000	0.872884000
C	0.756572000	2.715220000	0.530957000
C	2.966956000	2.687947000	-0.338656000
C	3.364972000	1.245940000	-0.494887000
C	2.841690000	0.229835000	0.190521000
C	3.319723000	-1.172607000	-0.097641000
C	-0.583387000	3.384879000	0.207066000
C	-1.774256000	2.510239000	-0.156575000
C	2.435198000	-3.434356000	-0.347757000
H	0.344448000	0.814218000	-1.205537000
H	1.798656000	-0.614358000	-1.963071000
H	1.789947000	-2.273599000	-2.544411000
H	-0.318786000	-2.740137000	-1.349981000
H	-0.544058000	-1.284983000	-2.316736000
H	-2.235639000	-3.150220000	0.286678000
H	-4.721620000	-2.941185000	0.262583000
H	-5.768181000	-0.736053000	-0.102749000
H	-4.366568000	1.289189000	-0.436332000
H	0.691634000	-0.603164000	2.809025000
H	2.024259000	-1.564471000	2.207947000
H	2.288263000	0.987463000	2.087926000
H	-0.120419000	1.224527000	1.742365000

H	1.207589000	3.217753000	1.397857000
H	3.222136000	3.053398000	0.667273000
H	3.511468000	3.301016000	-1.056925000
H	4.126700000	1.027893000	-1.237335000
H	3.757629000	-1.608157000	0.807642000
H	4.117549000	-1.143304000	-0.855331000
H	-0.934864000	3.965607000	1.061450000
H	-0.426669000	4.096960000	-0.605370000
H	3.182104000	-3.811075000	-1.061614000
H	1.496813000	-3.970859000	-0.501973000
H	2.774128000	-3.655178000	0.667218000

Iguesterin (*E* = -1238.6566452 Hartree), **solvent: Chloroform**

O	-6.515985000	0.828964000	-0.980628000
O	-5.263011000	2.642962000	0.407868000
C	1.664835000	0.209647000	0.439460000
C	1.052709000	-1.234116000	0.556100000
C	3.207686000	0.145140000	0.640981000
C	3.970699000	-0.892440000	-0.236299000
C	1.736260000	-2.153821000	-0.474477000
C	1.017908000	1.062140000	1.547436000
C	3.247444000	-2.246633000	-0.264085000
C	-0.452824000	-1.126279000	0.310083000
C	-1.275967000	-0.085816000	1.067721000
C	-0.492045000	1.217176000	1.361630000
C	3.905432000	1.516121000	0.541199000
C	1.294422000	0.850807000	-0.911740000
C	4.184739000	-0.422531000	-1.689983000
C	1.284356000	-1.881256000	1.944163000
C	5.362431000	-1.105703000	0.379394000

C	4.371220000	1.915807000	-0.835572000
C	-2.607327000	0.240401000	0.392565000
C	4.497860000	1.035816000	-1.825838000
C	-1.094763000	-1.963501000	-0.527049000
C	-1.724948000	-0.730896000	2.420174000
C	-3.246427000	-0.772002000	-0.440038000
C	-2.495336000	-1.834899000	-0.831704000
C	4.703501000	3.370494000	-1.003025000
C	-3.269779000	1.387275000	0.673619000
C	-4.617168000	-0.599626000	-0.901345000
C	-4.617025000	1.617166000	0.193424000
C	-5.255923000	0.547613000	-0.593704000
C	-5.286552000	-1.661989000	-1.726569000
H	3.341608000	-0.188422000	1.677032000
H	1.322172000	-3.162831000	-0.397862000
H	1.513088000	-1.802818000	-1.486268000
H	1.254205000	0.647187000	2.529525000
H	1.437091000	2.069842000	1.541324000
H	3.683858000	-2.853893000	-1.064795000
H	3.458376000	-2.789438000	0.660926000
H	-0.667690000	1.914771000	0.540451000
H	-0.913601000	1.686940000	2.253770000
H	3.260075000	2.308696000	0.928736000
H	4.778669000	1.524400000	1.205780000
H	0.220340000	0.798804000	-1.099602000
H	1.582245000	1.905582000	-0.913309000
H	1.788306000	0.378126000	-1.755233000
H	5.013417000	-1.008917000	-2.106556000
H	3.324524000	-0.679027000	-2.318577000
H	2.341475000	-2.060241000	2.130949000

H	0.917949000	-1.285580000	2.772540000
H	0.770879000	-2.845589000	1.973834000
H	5.288881000	-1.339137000	1.445325000
H	5.991309000	-0.220692000	0.260011000
H	5.865243000	-1.941103000	-0.115663000
H	4.844253000	1.378552000	-2.797416000
H	-0.562092000	-2.754556000	-1.034735000
H	-0.884183000	-0.876357000	3.092927000
H	-2.208139000	-1.693724000	2.245121000
H	-2.438569000	-0.061004000	2.902596000
H	-2.938179000	-2.593187000	-1.467044000
H	5.443067000	3.687423000	-0.260832000
H	3.814522000	3.989241000	-0.842721000
H	5.098156000	3.582673000	-1.997082000
H	-2.845358000	2.174604000	1.282335000
H	-5.278375000	-2.624803000	-1.210225000
H	-4.776591000	-1.798020000	-2.683807000
H	-6.319681000	-1.386127000	-1.926885000
H	-6.681785000	1.718644000	-0.621619000

Itoaic Acid (Conformer 1, *E* = -1622.9341401), solvent: Chloroform

C	-3.413288000	-1.105542000	-0.279555000
C	-2.563041000	0.163396000	0.063176000
C	-1.257222000	-0.111711000	0.904681000
C	-0.417540000	-1.136817000	0.063354000
C	-1.212748000	-2.428468000	-0.129514000
C	-2.488690000	-2.157511000	-0.916110000
C	-0.433070000	1.205729000	1.015288000
C	1.056816000	1.004579000	1.321189000
C	1.783605000	0.045401000	0.363139000

C	1.068393000	-1.346638000	0.483144000
C	3.302587000	-0.068337000	0.758368000
C	4.096708000	-1.285825000	0.182462000
C	3.242752000	-2.558567000	-0.037779000
C	1.778407000	-2.363689000	-0.432866000
C	4.133414000	1.249340000	0.657688000
C	5.078777000	1.525768000	-0.546076000
C	5.765422000	0.236257000	-1.014007000
C	4.789551000	-0.926039000	-1.146560000
C	-3.395788000	1.327971000	0.617025000
C	-4.457879000	1.915497000	-0.325165000
C	-5.718474000	1.070831000	-0.306622000
O	-5.669337000	-0.175599000	-0.754160000
C	-4.474977000	-0.729218000	-1.353252000
O	-4.791980000	3.213229000	0.087187000
O	-6.748461000	1.504807000	0.148502000
C	-5.013464000	-1.883684000	-2.186480000
C	-4.145403000	-1.724358000	0.921015000
C	-1.605190000	-0.579842000	2.335066000
O	-0.944749000	2.127497000	1.967938000
C	1.166582000	-1.921290000	1.911818000
C	5.172639000	-1.657028000	1.220249000
C	6.143994000	2.515490000	-0.057392000
C	4.388234000	2.175653000	-1.753374000
C	1.686306000	0.620700000	-1.053539000
O	1.527271000	1.956185000	-1.049196000
O	1.773363000	0.026332000	-2.100074000
H	-2.181879000	0.509057000	-0.910606000
H	-0.358957000	-0.693816000	-0.941235000
H	-1.441996000	-2.904313000	0.828162000

H	-0.628106000	-3.152406000	-0.696225000
H	-3.041170000	-3.090641000	-1.051125000
H	-2.197452000	-1.813278000	-1.916855000
H	-0.519566000	1.728412000	0.059096000
H	1.165028000	0.625057000	2.341241000
H	1.507737000	1.995447000	1.315597000
H	3.230253000	-0.250146000	1.835749000
H	3.274811000	-3.165038000	0.869939000
H	3.736899000	-3.166133000	-0.802144000
H	1.294252000	-3.337282000	-0.333347000
H	1.686543000	-2.059885000	-1.473166000
H	4.774017000	1.257211000	1.544372000
H	3.474717000	2.114211000	0.775832000
H	6.562303000	-0.033573000	-0.314530000
H	6.257747000	0.426620000	-1.973573000
H	5.330589000	-1.812921000	-1.491979000
H	4.031842000	-0.709755000	-1.904843000
H	-2.739480000	2.163906000	0.839571000
H	-3.883115000	1.073696000	1.563725000
H	-4.061216000	1.957533000	-1.346957000
H	-4.046396000	0.011162000	-2.035732000
H	-5.714773000	3.166576000	0.376211000
H	-4.246681000	-2.288882000	-2.843164000
H	-5.397691000	-2.685574000	-1.553796000
H	-5.833471000	-1.513396000	-2.801460000
H	-4.647444000	-0.975753000	1.535383000
H	-4.911741000	-2.419135000	0.570287000
H	-3.465477000	-2.285429000	1.558769000
H	-2.567895000	-0.181306000	2.654955000
H	-0.869413000	-0.237927000	3.065857000

H	-1.646897000	-1.663024000	2.430447000
H	-0.824451000	1.754347000	2.846492000
H	0.653211000	-2.886671000	1.935903000
H	0.722812000	-1.297529000	2.677809000
H	2.198059000	-2.102820000	2.208202000
H	5.809515000	-0.813954000	1.490978000
H	4.701863000	-2.021980000	2.137119000
H	5.814625000	-2.453479000	0.831884000
H	6.850970000	2.745376000	-0.859151000
H	5.685928000	3.453978000	0.268582000
H	6.706839000	2.101829000	0.783578000
H	5.151634000	2.494344000	-2.468636000
H	3.724856000	1.486936000	-2.277786000
H	3.818838000	3.057899000	-1.452958000
H	1.546033000	2.240547000	-1.974283000

Itoaic Acid (Conformer 2, *E* = -1622.9333399), solvent: Chloroform

C	-3.486878000	-1.102159000	0.156228000
C	-2.638540000	0.208153000	0.016195000
C	-1.239604000	0.182155000	0.746550000
C	-0.488175000	-1.056280000	0.148277000
C	-1.261494000	-2.337227000	0.462382000
C	-2.614592000	-2.310307000	-0.234332000
C	-0.418718000	1.450887000	0.371874000
C	1.083613000	1.323386000	0.647576000
C	1.744481000	0.135328000	-0.070368000
C	1.042094000	-1.175108000	0.406866000
C	3.279333000	0.063609000	0.260100000
C	3.977488000	-1.277217000	-0.198032000
C	3.018836000	-2.179864000	-0.988674000
C	1.626271000	-2.393548000	-0.370368000

C	4.062386000	1.302763000	-0.208653000
C	5.565709000	1.275744000	0.117765000
C	6.194132000	-0.046622000	-0.408837000
C	5.170415000	-0.936080000	-1.110344000
C	-3.441277000	1.485943000	0.299270000
C	-4.613925000	1.779634000	-0.648777000
C	-5.842158000	0.982907000	-0.250139000
O	-5.806497000	-0.339935000	-0.319430000
C	-4.667221000	-1.051090000	-0.857214000
O	-4.937239000	3.142936000	-0.593781000
O	-6.830333000	1.535310000	0.168223000
C	-5.261146000	-2.396006000	-1.251725000
C	-4.070589000	-1.327688000	1.559521000
C	-1.404011000	0.169379000	2.282181000
O	-0.866607000	2.642811000	1.001092000
C	1.292876000	-1.424305000	1.908391000
C	4.525861000	-2.088939000	0.995357000
C	5.798142000	1.426147000	1.624527000
C	6.216724000	2.468418000	-0.589741000
C	1.502878000	0.376389000	-1.573401000
O	1.736135000	1.653710000	-1.928415000
O	1.110494000	-0.416700000	-2.391392000
H	-2.376008000	0.253760000	-1.052273000
H	-0.572078000	-0.932050000	-0.936528000
H	-1.392419000	-2.475407000	1.538427000
H	-0.707974000	-3.210454000	0.112374000
H	-3.161628000	-3.232164000	-0.021810000
H	-2.436938000	-2.286590000	-1.317082000
H	-0.560084000	1.637276000	-0.700097000
H	1.254074000	1.230309000	1.723934000

H	1.533296000	2.265867000	0.341658000
H	3.302166000	0.086833000	1.354125000
H	3.501965000	-3.154423000	-1.116666000
H	2.900422000	-1.771432000	-1.991563000
H	1.653456000	-3.249186000	0.310805000
H	0.951438000	-2.667429000	-1.183177000
H	3.629971000	2.202659000	0.238170000
H	3.957822000	1.425414000	-1.287512000
H	6.641105000	-0.601902000	0.421099000
H	7.013921000	0.179862000	-1.095853000
H	5.640435000	-1.869775000	-1.435241000
H	4.807835000	-0.443973000	-2.019364000
H	-2.788090000	2.346920000	0.196190000
H	-3.817356000	1.518956000	1.326832000
H	-4.329531000	1.521634000	-1.676269000
H	-4.334039000	-0.546376000	-1.769332000
H	-5.824770000	3.185789000	-0.209318000
H	-4.558891000	-2.981430000	-1.841067000
H	-5.554555000	-2.973705000	-0.373580000
H	-6.151555000	-2.217280000	-1.854117000
H	-4.521442000	-0.425479000	1.974705000
H	-4.852876000	-2.088909000	1.518701000
H	-3.312526000	-1.677715000	2.257100000
H	-2.316377000	0.684722000	2.584464000
H	-0.575868000	0.675880000	2.783401000
H	-1.440001000	-0.834606000	2.699412000
H	-0.734632000	2.549804000	1.949923000
H	0.718745000	-2.299163000	2.221289000
H	0.998794000	-0.595847000	2.546495000
H	2.335687000	-1.639840000	2.119535000

H	4.940041000	-1.457742000	1.783945000
H	3.761347000	-2.724758000	1.441582000
H	5.323398000	-2.752963000	0.649668000
H	5.337007000	0.617292000	2.195265000
H	6.868734000	1.417815000	1.846388000
H	5.384859000	2.371518000	1.988028000
H	6.100156000	2.384702000	-1.673923000
H	5.763016000	3.411162000	-0.270824000
H	7.286273000	2.512546000	-0.367014000
H	1.548585000	1.714411000	-2.876003000

Matopensine (E= -1766.926531), solvent: Chloroform

O	-0.000014000	-0.000025000	2.581554000
N	-1.319667000	-0.896838000	0.921444000
N	-3.857566000	2.144284000	-1.041091000
C	-2.079272000	0.327311000	1.221983000
C	-3.340258000	0.198432000	0.330979000
C	-1.146753000	1.532323000	1.037888000
C	-0.187206000	-1.157474000	1.786635000
C	-3.129744000	0.868185000	-1.069391000
C	-0.994009000	1.984409000	-0.449622000
C	-1.660874000	1.002252000	-1.431691000
C	-4.593730000	0.914967000	0.879744000
C	-3.454475000	-1.307984000	0.229732000
C	-2.237754000	-1.891219000	0.607285000
C	-5.069539000	1.791223000	-0.299157000
C	-1.698822000	3.299091000	-0.703349000
C	-3.162848000	3.253918000	-0.358508000
C	-4.510288000	-2.102876000	-0.163709000
C	-2.070551000	-3.268215000	0.629926000

C	-1.173650000	4.374887000	-1.290528000
C	-4.357187000	-3.495255000	-0.156680000
C	-3.154079000	-4.061988000	0.240997000
C	0.221413000	4.569260000	-1.803042000
H	-2.406834000	0.297773000	2.270694000
H	-1.570955000	2.362146000	1.607093000
H	-0.436762000	-1.951550000	2.499630000
H	-3.638990000	0.239902000	-1.808148000
H	0.061768000	2.068269000	-0.696695000
H	-1.561014000	1.401479000	-2.443653000
H	-1.191195000	0.017261000	-1.403649000
H	-4.330289000	1.531923000	1.742944000
H	-5.355355000	0.208833000	1.211508000
H	-5.725134000	1.211271000	-0.956004000
H	-5.609639000	2.686634000	0.011909000
H	-3.649595000	4.185609000	-0.657040000
H	-3.278123000	3.177315000	0.734685000
H	-5.454173000	-1.656535000	-0.459992000
H	-1.126523000	-3.717627000	0.920379000
H	-1.842478000	5.217593000	-1.454363000
H	-5.181241000	-4.130307000	-0.454542000
H	-3.046921000	-5.140064000	0.250996000
H	0.185803000	4.896557000	-2.845570000
H	0.742963000	5.353045000	-1.245292000
H	0.826431000	3.665978000	-1.750629000
N	1.319641000	0.896819000	0.921462000
N	3.857598000	-2.144268000	-1.041059000
C	2.079252000	-0.327328000	1.221991000
C	3.340248000	-0.198425000	0.331005000
C	1.146739000	-1.532343000	1.037870000

C	0.187182000	1.157439000	1.786658000
C	3.129765000	-0.868175000	-1.069370000
C	0.994025000	-1.984420000	-0.449649000
C	1.660902000	-1.002250000	-1.431697000
C	4.593722000	-0.914944000	0.879788000
C	3.454447000	1.307992000	0.229761000
C	2.237715000	1.891211000	0.607301000
C	5.069555000	-1.791199000	-0.299104000
C	1.698858000	-3.299093000	-0.703375000
C	3.162873000	-3.253908000	-0.358493000
C	4.510252000	2.102898000	-0.163673000
C	2.070490000	3.268205000	0.629932000
C	1.173726000	-4.374886000	-1.290596000
C	4.357131000	3.495275000	-0.156652000
C	3.154010000	4.061993000	0.241009000
C	-0.221305000	-4.569267000	-1.803197000
H	2.406801000	-0.297801000	2.270706000
H	1.570941000	-2.362169000	1.607072000
H	0.436735000	1.951505000	2.499665000
H	3.639020000	-0.239886000	-1.808115000
H	-0.061745000	-2.068286000	-0.696745000
H	1.561065000	-1.401471000	-2.443664000
H	1.191215000	-0.017263000	-1.403657000
H	4.330278000	-1.531900000	1.742986000
H	5.355336000	-0.208800000	1.211559000
H	5.725158000	-1.211244000	-0.955941000
H	5.609656000	-2.686606000	0.011972000
H	3.649636000	-4.185593000	-0.657015000
H	3.278118000	-3.177307000	0.734704000
H	5.454145000	1.656570000	-0.459948000

H	1.126452000	3.717604000	0.920374000
H	1.842574000	-5.217578000	-1.454416000
H	5.181179000	4.130338000	-0.454508000
H	3.046836000	5.140067000	0.251002000
H	-0.185626000	-4.896549000	-2.845727000
H	-0.742879000	-5.353065000	-1.245490000
H	-0.826335000	-3.665993000	-1.750809000

Naucleidinal (*E* = -1108.9940687 Hartree), solvent: Chloroform

O	-3.455560000	1.451524000	1.304669000
O	-1.232384000	-2.000317000	1.020385000
O	-6.792995000	-0.118048000	-1.570420000
N	-3.004836000	-3.223575000	0.316754000
N	-5.078669000	-2.943367000	-2.713953000
C	-4.340030000	-3.290008000	-0.283347000
C	-4.568132000	-0.830082000	-0.095073000
C	-5.239911000	-2.163820000	0.198561000
C	-5.398135000	0.375679000	0.318195000
C	-4.140591000	-3.313446000	-1.774320000
C	-3.249622000	-0.817955000	0.625016000
C	-2.133922000	-4.311810000	-0.122527000
C	-2.985395000	-3.714110000	-2.380708000
C	-2.404521000	-2.037706000	0.672635000
C	-1.773601000	-4.151749000	-1.610469000
C	-4.502490000	1.627491000	0.344137000
C	-3.195410000	-3.565796000	-3.794824000
C	-2.823024000	0.265951000	1.280576000
C	-4.509311000	-3.064431000	-3.963633000
C	-6.590602000	0.558610000	-0.593451000
C	-5.213311000	2.909702000	0.722744000

C	-2.405593000	-3.800352000	-4.927713000
C	-5.039082000	-2.781872000	-5.222026000
C	-2.929036000	-3.523421000	-6.177520000
C	-4.232411000	-3.016005000	-6.321969000
H	-4.780910000	-4.241019000	0.036988000
H	-4.406687000	-0.764872000	-1.177595000
H	-6.213266000	-2.240870000	-0.289566000
H	-5.397803000	-2.269872000	1.275671000
H	-5.792021000	0.251036000	1.337376000
H	-2.675515000	-5.247820000	0.033962000
H	-1.244219000	-4.312508000	0.501123000
H	-1.377224000	-5.097445000	-1.990688000
H	-0.974295000	-3.409778000	-1.712012000
H	-4.027420000	1.727678000	-0.639589000
H	-5.910644000	-2.407489000	-2.513481000
H	-1.913241000	0.260673000	1.867005000
H	-7.301357000	1.356311000	-0.312699000
H	-5.904837000	3.218475000	-0.061373000
H	-5.767899000	2.780239000	1.654200000
H	-4.478486000	3.702529000	0.861390000
H	-1.399271000	-4.188825000	-4.822392000
H	-6.044081000	-2.393747000	-5.331609000
H	-2.328948000	-3.697577000	-7.061870000
H	-4.613498000	-2.806706000	-7.313661000

Physalin D (*E* = -1913.1327978 Hartree), solvent: Dimethylsulfoxide

C	-6.150911000	0.490112000	-0.597572000
C	-5.533981000	-0.859538000	-0.382352000
C	-4.014460000	-0.817076000	-0.544486000
C	-3.387595000	0.348425000	0.278875000

C	-3.968823000	1.614171000	-0.366988000
C	-5.437713000	1.619866000	-0.589590000
C	-3.388191000	-2.169984000	-0.214272000
C	-1.876026000	-2.123314000	-0.359808000
C	-1.242145000	-1.023404000	0.503574000
C	-1.842253000	0.354863000	0.138074000
O	-3.296617000	2.567030000	-0.701131000
C	0.304003000	-1.061346000	0.383335000
C	1.114617000	-0.774804000	1.664820000
C	2.368236000	-0.037705000	1.219154000
C	1.774015000	0.770684000	0.060676000
C	1.215257000	2.126835000	0.580832000
C	-0.222804000	2.405870000	0.159838000
C	-1.219735000	1.522419000	0.934911000
O	0.807267000	-1.116145000	2.770949000
O	0.784204000	-0.079185000	-0.523149000
O	0.662051000	-2.333482000	-0.083496000
C	3.533690000	-0.978267000	0.837503000
C	3.143182000	-2.140220000	-0.103324000
C	1.894741000	-2.928161000	0.292210000
C	4.569938000	-0.118503000	0.113049000
C	4.003162000	0.351301000	-1.208678000
C	2.738341000	1.213231000	-1.075386000
C	2.240276000	3.133006000	0.056207000
O	3.187536000	2.524023000	-0.674357000
O	2.275107000	4.296357000	0.331162000
C	3.088820000	-1.846232000	-1.596310000
O	3.705285000	-0.750997000	-2.072428000
C	2.041634000	1.363226000	-2.422987000
O	2.604469000	-2.616786000	-2.378396000

C	4.123162000	-1.542342000	2.132227000
O	-3.687695000	-0.627529000	-1.922605000
O	-3.780979000	-2.538711000	1.096489000
C	-3.838000000	0.334461000	1.756572000
O	1.317213000	2.211447000	1.994235000
H	-7.217919000	0.528253000	-0.790231000
H	-5.938944000	-1.569692000	-1.110142000
H	-5.800901000	-1.243826000	0.605728000
H	-5.882803000	2.584555000	-0.801208000
H	-3.793729000	-2.882616000	-0.944451000
H	-1.626971000	-1.959300000	-1.411429000
H	-1.476932000	-3.098829000	-0.084382000
H	-1.458709000	-1.247608000	1.555439000
H	-1.640266000	0.515566000	-0.924275000
H	2.714401000	0.602635000	2.024822000
H	-0.351813000	2.277693000	-0.915212000
H	-0.407087000	3.461631000	0.378920000
H	-2.009285000	2.181255000	1.283295000
H	-0.743811000	1.143446000	1.843888000
H	3.972800000	-2.856947000	-0.038171000
H	1.909175000	-3.138905000	1.365718000
H	1.901051000	-3.877501000	-0.237958000
H	4.839366000	0.749654000	0.720288000
H	5.480032000	-0.694605000	-0.072616000
H	4.735180000	0.942497000	-1.759898000
H	2.772557000	1.708327000	-3.156020000
H	1.247469000	2.107585000	-2.354342000
H	1.615005000	0.422013000	-2.757293000
H	4.489185000	-0.727249000	2.759964000
H	3.379385000	-2.096702000	2.708301000

H	4.959584000	-2.208835000	1.912474000
H	-4.313908000	-0.001165000	-2.302225000
H	-3.399775000	-3.401981000	1.284154000
H	-3.453868000	1.207502000	2.283653000
H	-4.924319000	0.360693000	1.840024000
H	-3.483682000	-0.562894000	2.257897000
H	1.104173000	3.121745000	2.236486000

Strychnobaillonine (Conformer 1, *E* = -1919.5604105 Hartree), solvent: Chloroform

O	1.206517000	2.866520000	1.686721000
O	-1.243761000	3.446499000	0.810281000
N	-2.377763000	1.612470000	0.103383000
N	-4.179653000	-2.421856000	-1.219692000
N	1.062123000	-0.657609000	0.360307000
N	5.081666000	-0.131665000	-1.537609000
C	-5.461101000	3.196162000	1.492524000
C	-6.372904000	2.252530000	1.030466000
C	-5.931319000	1.163274000	0.281457000
C	-4.580236000	1.036572000	0.006580000
C	-3.680374000	1.986950000	0.478241000
C	-4.098314000	3.082820000	1.224649000
C	-3.886622000	-0.049925000	-0.778036000
C	-2.394045000	0.430287000	-0.790818000
C	-1.242636000	2.324351000	0.320475000
C	0.018493000	1.561200000	-0.047111000
C	-0.174420000	0.107633000	0.449671000
C	-1.251539000	-0.570143000	-0.427621000
C	-4.501874000	-0.251058000	-2.192239000
C	-5.024229000	-1.707267000	-2.178597000
C	-4.089336000	-1.454157000	-0.122144000

C	-3.028248000	-1.784710000	0.908038000
C	-1.680882000	-1.921225000	0.198836000
C	0.161696000	-4.440448000	-0.099867000
C	-1.014524000	-4.069343000	-0.953108000
C	-1.800701000	-2.998839000	-0.847496000
C	-2.883887000	-2.722957000	-1.849560000
C	1.308196000	2.278682000	0.408504000
C	2.478519000	1.297998000	0.496562000
C	2.207666000	0.007406000	-0.278329000
C	1.583774000	-1.418244000	1.407510000
C	3.358279000	-1.019663000	-0.127913000
C	2.937792000	-1.702539000	1.154966000
C	0.968455000	-1.884677000	2.563335000
C	1.717017000	-2.678479000	3.437373000
C	3.042436000	-2.993287000	3.174788000
C	3.661070000	-2.493522000	2.022851000
C	3.857188000	1.912432000	0.212339000
C	4.900312000	0.903712000	0.697580000
C	4.761174000	-0.359064000	-0.127003000
C	4.684635000	-1.397236000	-2.164922000
C	3.466530000	-1.952743000	-1.359823000
C	4.374359000	1.008238000	-2.152534000
C	4.132743000	2.204318000	-1.245490000
C	4.164260000	3.431971000	-1.767785000
C	3.855248000	4.730664000	-1.083019000
H	-3.389775000	3.815331000	1.574982000
H	-2.998860000	-0.986139000	1.658684000
H	-5.074088000	-1.439172000	0.356438000
H	-6.058011000	-1.735363000	-1.823078000
H	-5.297065000	0.468121000	-2.387387000

H	-3.020557000	-3.576656000	-2.517068000
H	-6.637164000	0.426772000	-0.088260000
H	-7.426656000	2.365848000	1.249705000
H	-5.812319000	4.041443000	2.071016000
H	0.056004000	1.518923000	-1.144305000
H	-0.504307000	0.140087000	1.495848000
H	1.092371000	-4.334944000	-0.666773000
H	0.255125000	-3.833097000	0.799382000
H	-2.184827000	0.801483000	-1.798702000
H	-0.917582000	-2.213453000	0.914858000
H	-2.548388000	-1.879580000	-2.481861000
H	-1.226562000	-4.755956000	-1.770569000
H	-3.745308000	-0.111190000	-2.966396000
H	-4.990986000	-2.186552000	-3.157866000
H	-3.278429000	-2.716271000	1.418909000
H	-0.731098000	-0.793916000	-1.364161000
H	0.095206000	-5.488317000	0.202667000
H	1.527635000	3.046000000	-0.348761000
H	0.435866000	3.450470000	1.648928000
H	3.100993000	5.282978000	-1.649869000
H	3.477091000	4.600253000	-0.069680000
H	4.742806000	5.368267000	-1.039443000
H	4.433125000	3.520388000	-2.819001000
H	4.955841000	1.336166000	-3.018538000
H	3.403823000	0.681880000	-2.556371000
H	4.742574000	0.674637000	1.754738000
H	5.911816000	1.298421000	0.579294000
H	3.936737000	2.830726000	0.795504000
H	2.496989000	0.997456000	1.551893000
H	1.975041000	0.201385000	-1.326145000

H	-0.060641000	-1.642632000	2.798305000
H	1.246386000	-3.048587000	4.339986000
H	3.600860000	-3.611607000	3.865135000
H	4.702535000	-2.719712000	1.820643000
H	3.655554000	-2.979766000	-1.043285000
H	5.518594000	-2.099631000	-2.096299000
H	2.537969000	-1.953720000	-1.934768000
H	4.467966000	-1.246361000	-3.222970000
H	5.471135000	-1.113990000	0.233928000

Strychnobaillonine (Conformer 2, *E* = -1919.5615687 Hartree), solvent: Chloroform

O	1.264672000	2.776918000	1.714548000
O	-1.100447000	3.452110000	0.688707000
N	-2.303851000	1.619483000	0.106231000
N	-4.651402000	-2.387600000	-0.224906000
N	1.105925000	-0.755736000	0.295642000
N	5.232128000	-0.180028000	-1.409215000
C	-5.359992000	3.638846000	0.856613000
C	-6.300671000	2.702522000	0.442226000
C	-5.887931000	1.464411000	-0.048516000
C	-4.533949000	1.185973000	-0.126907000
C	-3.603319000	2.138686000	0.286416000
C	-3.993158000	3.374854000	0.786870000
C	-3.844489000	-0.086377000	-0.567186000
C	-2.376997000	0.394960000	-0.721941000
C	-1.142401000	2.297758000	0.282097000
C	0.098611000	1.484213000	-0.045511000
C	-0.117416000	0.031264000	0.434216000
C	-1.234315000	-0.608114000	-0.422772000
C	-4.397703000	-0.684169000	-1.876274000

C	-5.312525000	-1.812953000	-1.392837000
C	-4.082450000	-1.237356000	0.495670000
C	-2.805124000	-1.618788000	1.233453000
C	-1.721616000	-1.933156000	0.194649000
C	-0.080195000	-3.193556000	-2.102577000
C	-1.535863000	-3.438067000	-1.827236000
C	-2.257766000	-2.926807000	-0.829047000
C	-3.658924000	-3.418741000	-0.546625000
C	1.390374000	2.179373000	0.443035000
C	2.534421000	1.176732000	0.568106000
C	2.280828000	-0.073460000	-0.273751000
C	1.591115000	-1.593764000	1.303991000
C	3.418720000	-1.119322000	-0.146533000
C	2.948025000	-1.878515000	1.073953000
C	0.935681000	-2.124002000	2.409049000
C	1.650065000	-2.974070000	3.257411000
C	2.980859000	-3.284754000	3.017723000
C	3.638262000	-2.725734000	1.916152000
C	3.933846000	1.781196000	0.382449000
C	4.937712000	0.733535000	0.865754000
C	4.828755000	-0.479021000	-0.034559000
C	4.863342000	-1.409098000	-2.118885000
C	3.574010000	-1.967277000	-1.436504000
C	4.553053000	0.989991000	-1.998138000
C	4.281185000	2.141870000	-1.044504000
C	4.344172000	3.394278000	-1.500868000
C	4.006528000	4.656539000	-0.762886000
H	-3.260043000	4.100205000	1.098944000
H	-2.493995000	-0.788434000	1.874655000
H	-4.832817000	-0.873837000	1.203385000

H	-6.282859000	-1.409974000	-1.083064000
H	-4.903116000	0.066588000	-2.485532000
H	-3.598917000	-4.099839000	0.311516000
H	-6.620297000	0.731405000	-0.366505000
H	-7.356310000	2.934152000	0.501416000
H	-5.689684000	4.596948000	1.238529000
H	0.156474000	1.449545000	-1.142660000
H	-0.410666000	0.058983000	1.491861000
H	0.418193000	-2.676531000	-1.280630000
H	0.432589000	-4.144341000	-2.268512000
H	-2.273081000	0.726887000	-1.761239000
H	-0.859928000	-2.400038000	0.676949000
H	-4.032454000	-4.013291000	-1.382285000
H	-2.031050000	-4.141425000	-2.493679000
H	-3.576456000	-1.109102000	-2.460671000
H	-5.501117000	-2.577537000	-2.146825000
H	-2.997644000	-2.472288000	1.887014000
H	-0.756061000	-0.828235000	-1.379118000
H	0.061108000	-2.599793000	-3.012377000
H	1.647438000	2.937376000	-0.311325000
H	0.530722000	3.402446000	1.639477000
H	3.543107000	4.473243000	0.206084000
H	4.899393000	5.268930000	-0.607355000
H	3.312201000	5.260024000	-1.353305000
H	4.664067000	3.535314000	-2.531758000
H	5.167779000	1.354287000	-2.825694000
H	3.596564000	0.687249000	-2.451763000
H	4.720925000	0.449112000	1.898759000
H	5.958513000	1.119410000	0.823523000
H	3.996230000	2.668017000	1.014054000

H	2.488976000	0.836872000	1.611082000
H	2.083291000	0.175392000	-1.317486000
H	-0.098232000	-1.885380000	2.625921000
H	1.148888000	-3.392058000	4.121857000
H	3.513051000	-3.947174000	3.687651000
H	4.683544000	-2.950316000	1.733336000
H	3.692451000	-3.024035000	-1.192801000
H	5.676407000	-2.130978000	-2.012083000
H	2.690913000	-1.876522000	-2.072574000
H	4.734590000	-1.211913000	-3.183690000
H	5.507349000	-1.263812000	0.321957000

Equilibrium geometries of molecules from set 1 calculated at the **DFT(M06-2X)** level of theory with **6-311G(3df,3pd)** basis set within the IEF-PCM model. Cartesian coordinates are given for standard orientation in form (atomic label, X, Y, Z). The X, Y, Z are given in Å.

12-28-oxaircinal (*E* = -1272.230678 Hartree), solvent: Chloroform

O	1.589700000	-0.786084000	1.822954000
O	-0.191677000	4.722645000	1.510344000
N	1.767561000	-0.500613000	-0.458643000
N	-2.185792000	0.487229000	-1.534957000
C	0.066448000	1.168139000	-0.860252000
C	0.391844000	-0.135916000	-0.101413000
C	0.395679000	-0.099836000	1.454199000
C	-0.581851000	2.284711000	-0.007164000
C	1.452353000	1.615135000	-1.385975000
C	2.488443000	0.766106000	-0.638445000
C	-0.860738000	0.811092000	-2.026332000
C	2.182612000	-1.347255000	0.641951000
C	-2.106784000	2.171058000	0.190237000
C	0.077107000	2.376664000	1.338874000

C	0.499984000	1.295696000	1.995446000
C	-0.849530000	-0.808817000	2.014396000
C	-2.827696000	1.702603000	-1.062808000
C	3.671448000	-1.491732000	0.867472000
C	3.789863000	0.695022000	-1.383565000
C	-2.976540000	-0.305476000	-2.460604000
C	-0.834667000	-2.331246000	1.810973000
C	4.374957000	-2.311412000	-0.212159000
C	0.197037000	3.689478000	1.994786000
C	4.467825000	-0.359723000	-1.823845000
C	4.121893000	-1.815119000	-1.645176000
C	-4.288689000	-0.807367000	-1.849544000
C	-2.193269000	-2.932195000	2.058280000
C	-4.168071000	-1.327699000	-0.416701000
C	-3.159109000	-2.467406000	-0.239688000
C	-3.178915000	-2.993080000	1.167451000
H	-0.290818000	-0.922161000	-0.418191000
H	-0.408753000	3.223591000	-0.538067000
H	1.631432000	2.680386000	-1.248819000
H	1.527675000	1.396595000	-2.452933000
H	2.686741000	1.242617000	0.333905000
H	-0.891484000	1.645544000	-2.747431000
H	-0.442062000	-0.053265000	-2.545861000
H	1.739535000	-2.336816000	0.475484000
H	-2.481988000	3.143151000	0.513070000
H	-2.324477000	1.463093000	0.988363000
H	0.944780000	1.387298000	2.982289000
H	-1.719337000	-0.400628000	1.497856000
H	-0.966199000	-0.583339000	3.076376000
H	-3.870127000	1.503326000	-0.823189000

H	-2.812875000	2.483089000	-1.841455000
H	3.796352000	-1.982011000	1.833027000
H	4.117142000	-0.501285000	0.956602000
H	4.212407000	1.677853000	-1.573361000
H	-2.362699000	-1.162665000	-2.744229000
H	-3.199168000	0.246139000	-3.387948000
H	-0.096226000	-2.773478000	2.478105000
H	-0.517771000	-2.566869000	0.796181000
H	4.052235000	-3.352405000	-0.144653000
H	5.446606000	-2.299618000	-0.007515000
H	0.678228000	3.673935000	2.989121000
H	5.400669000	-0.153951000	-2.338051000
H	3.079346000	-1.985781000	-1.900784000
H	4.726027000	-2.410743000	-2.328527000
H	-4.669408000	-1.601074000	-2.496091000
H	-5.038543000	-0.015386000	-1.873409000
H	-2.379859000	-3.317427000	3.054848000
H	-5.151691000	-1.669123000	-0.086463000
H	-3.881010000	-0.511846000	0.250067000
H	-2.166802000	-2.107324000	-0.508907000
H	-3.412691000	-3.274281000	-0.934381000
H	-4.121561000	-3.432488000	1.481423000

Anabsinthin (Conformer 1, *E* = -1618.5731115 Hartree), solvent: Acetonitrile

O	-2.318974000	1.158295000	-2.235880000
O	-2.550567000	-1.944211000	0.630941000
O	2.414429000	0.216584000	3.112719000
O	3.053000000	-1.654480000	-1.202514000
O	-3.737850000	-3.381760000	1.838732000
O	4.517633000	-2.167751000	-2.795264000

C	-1.640120000	0.168029000	-0.250796000
C	-1.970055000	1.668632000	0.000936000
C	-0.492592000	2.125797000	-0.078803000
C	-1.157376000	0.498124000	-1.699137000
C	-0.524781000	-0.114535000	0.788136000
C	0.147510000	1.262940000	1.018550000
C	-2.797039000	2.080536000	-1.230915000
C	-0.081175000	1.571198000	-1.461404000
C	-2.805022000	-0.824506000	-0.250290000
C	1.677866000	1.057731000	0.916813000
C	0.612582000	-1.021886000	0.407722000
C	-4.171128000	-0.308727000	0.194922000
C	-4.313619000	1.916468000	-1.046242000
C	1.781296000	-0.377722000	0.433549000
C	-0.742354000	-0.591821000	-2.653997000
C	-4.860392000	0.502275000	-0.887041000
C	2.488284000	1.327690000	2.225455000
C	-2.562795000	3.524981000	-1.673245000
C	-4.865292000	-1.613014000	0.581839000
C	3.091731000	-1.018489000	0.104211000
C	0.393023000	-2.473942000	0.130781000
C	3.984910000	1.542691000	1.951522000
C	-3.706956000	-2.435065000	1.106646000
C	4.294095000	-0.089555000	0.015028000
C	4.787145000	0.380911000	1.371177000
C	1.978903000	2.589397000	2.928726000
C	-6.014883000	-1.513488000	1.566180000
C	5.252494000	-0.949591000	-0.805637000
C	4.289298000	-1.664843000	-1.733625000
C	6.360144000	-0.228614000	-1.548923000

H	-2.441579000	1.902050000	0.953678000
H	-0.319853000	3.191644000	0.052761000
H	-1.007739000	-0.492065000	1.691301000
H	-0.147371000	1.664546000	1.983486000
H	0.917244000	1.142024000	-1.466563000
H	-0.129136000	2.305920000	-2.261160000
H	-2.920811000	-1.245876000	-1.251215000
H	2.083344000	1.758256000	0.179967000
H	-4.048457000	0.291215000	1.102761000
H	-4.805752000	2.376494000	-1.905112000
H	-4.592475000	2.512623000	-0.174113000
H	-1.508284000	-1.357078000	-2.769817000
H	-0.560954000	-0.146842000	-3.632607000
H	0.181593000	-1.057083000	-2.315229000
H	-4.773968000	-0.038202000	-1.832206000
H	-5.925616000	0.575462000	-0.661561000
H	-3.067130000	3.694087000	-2.623924000
H	-2.986911000	4.207844000	-0.936319000
H	-1.512263000	3.766675000	-1.795594000
H	-5.196454000	-2.116778000	-0.334311000
H	3.311035000	-1.809960000	0.829433000
H	-0.303008000	-2.617279000	-0.697025000
H	1.319387000	-2.991308000	-0.102133000
H	-0.073730000	-2.939748000	0.999932000
H	4.440330000	1.820141000	2.903228000
H	4.077853000	2.409528000	1.293115000
H	4.017925000	0.774640000	-0.599544000
H	4.785102000	-0.461078000	2.066995000
H	5.823471000	0.710529000	1.278689000
H	1.016362000	2.419780000	3.405731000

H	1.871193000	3.412279000	2.220758000
H	2.683107000	2.886809000	3.703844000
H	-5.687886000	-1.027349000	2.484939000
H	-6.388867000	-2.503915000	1.817372000
H	-6.832600000	-0.934160000	1.141468000
H	1.490116000	0.017917000	3.290699000
H	5.675459000	-1.722834000	-0.153202000
H	6.928321000	-0.928569000	-2.158125000
H	7.041958000	0.250737000	-0.848929000
H	5.943464000	0.535899000	-2.204175000

Anabsinthin (Conformer 2, *E* = -1618.5730168 Hartree), solvent: Acetonitrile

O	-2.182792000	2.335780000	-1.005151000
O	-2.595002000	-1.862042000	-0.734075000
O	1.526900000	-1.740449000	3.005091000
O	3.043710000	-0.452452000	-2.001134000
O	-3.858361000	-3.688878000	-0.703547000
O	4.465000000	0.399323000	-3.484908000
C	-1.592941000	0.291108000	-0.082415000
C	-1.876714000	1.315447000	1.055033000
C	-0.382120000	1.657194000	1.277691000
C	-1.057448000	1.436628000	-1.003406000
C	-0.520620000	-0.632691000	0.550665000
C	0.192160000	0.271691000	1.594694000
C	-2.656922000	2.442763000	0.355814000
C	0.046475000	2.078306000	-0.144969000
C	-2.790258000	-0.428016000	-0.707736000
C	1.703598000	0.076126000	1.378797000
C	0.588978000	-1.133788000	-0.341490000
C	-4.148557000	-0.252265000	-0.033507000

C	-4.182519000	2.263443000	0.398294000
C	1.775210000	-0.681277000	0.070704000
C	-0.656121000	1.177846000	-2.433499000
C	-4.782738000	1.086745000	-0.363174000
C	2.385740000	-0.699941000	2.540415000
C	-2.363749000	3.833491000	0.918901000
C	-4.895619000	-1.479863000	-0.550877000
C	3.092657000	-0.886421000	-0.617799000
C	0.331345000	-2.038669000	-1.501786000
C	3.710160000	-1.368149000	2.126699000
C	-3.778183000	-2.494864000	-0.670901000
C	4.225591000	-0.052066000	-0.025578000
C	4.735969000	-0.554779000	1.327871000
C	2.585278000	0.229885000	3.725889000
C	-6.058753000	-1.977533000	0.285861000
C	5.206919000	-0.029939000	-1.192545000
C	4.253012000	0.013275000	-2.373007000
C	6.227432000	1.091334000	-1.211588000
H	-2.363571000	0.919648000	1.944343000
H	-0.178004000	2.395426000	2.050096000
H	-1.037599000	-1.472750000	1.017425000
H	-0.092752000	-0.010592000	2.605409000
H	1.032903000	1.710729000	-0.416435000
H	0.039185000	3.153404000	-0.306083000
H	-2.895149000	-0.116398000	-1.749314000
H	2.206183000	1.043790000	1.313957000
H	-4.027386000	-0.362098000	1.049218000
H	-4.636994000	3.180591000	0.019093000
H	-4.462653000	2.192603000	1.451983000
H	-0.462574000	2.135905000	-2.916527000

H	0.258237000	0.588493000	-2.469805000
H	-1.434956000	0.668528000	-2.999073000
H	-4.694123000	1.255552000	-1.438633000
H	-5.849676000	1.047757000	-0.137141000
H	-2.783973000	3.919231000	1.921533000
H	-1.303056000	4.054232000	0.975247000
H	-2.837458000	4.583014000	0.285989000
H	-5.227863000	-1.280540000	-1.576751000
H	3.370969000	-1.944829000	-0.638526000
H	-0.124458000	-2.964357000	-1.147418000
H	-0.383445000	-1.597659000	-2.197111000
H	1.245831000	-2.270891000	-2.042148000
H	3.452861000	-2.259719000	1.549308000
H	4.182278000	-1.733255000	3.039876000
H	3.843581000	0.966370000	0.077736000
H	5.597719000	-1.204053000	1.160182000
H	5.098447000	0.288955000	1.915825000
H	2.925228000	-0.339948000	4.589593000
H	1.652037000	0.730761000	3.983380000
H	3.329837000	0.989471000	3.490676000
H	-6.471727000	-2.890014000	-0.139283000
H	-6.847713000	-1.228879000	0.327400000
H	-5.731036000	-2.190924000	1.302966000
H	1.351356000	-2.337405000	2.269757000
H	5.712163000	-1.001256000	-1.248471000
H	6.902066000	1.003554000	-0.361689000
H	5.729766000	2.059418000	-1.159197000
H	6.816313000	1.057753000	-2.125872000

Betulinic Acid (E = -1397.6733967 Hartree), solvent: Pyridine

C	4.183179000	1.207832000	0.350447000
C	3.155691000	0.294135000	-0.351112000
C	3.796501000	-1.101740000	-0.372300000
C	5.246125000	-0.756908000	-0.756255000
C	5.552840000	0.498551000	0.080620000
C	1.715543000	0.257936000	0.151696000
C	0.841164000	-0.713870000	-0.697789000
C	1.548792000	-2.084247000	-0.865696000
C	3.010982000	-1.994531000	-1.327894000
C	1.066161000	1.637485000	0.229716000
C	-0.367045000	1.578527000	0.769493000
C	-1.249418000	0.525993000	0.080417000
C	-0.551980000	-0.869669000	0.052815000
C	-2.740849000	0.540818000	0.564312000
C	-3.507220000	-0.455362000	-0.353694000
C	-2.910948000	-1.858561000	-0.243905000
C	-1.449748000	-1.875219000	-0.690778000
C	-3.305186000	1.953350000	0.304755000
C	-4.825350000	2.027521000	0.393133000
C	-5.496425000	1.038508000	-0.537979000
C	-5.060428000	-0.420462000	-0.281175000
C	4.210729000	2.642503000	-0.121652000
C	4.256882000	3.649381000	0.744318000
C	4.242092000	2.883807000	-1.606195000
C	3.865723000	-1.741653000	1.007732000
O	3.730781000	-1.189459000	2.067482000
O	4.171339000	-3.048023000	0.938132000
C	0.665367000	-0.162015000	-2.129635000
C	-0.338794000	-1.412045000	1.483560000
C	-2.890392000	0.242720000	2.070864000

C	-5.633768000	-1.281694000	-1.417033000
C	-5.648209000	-0.946711000	1.032875000
O	-6.896444000	1.230441000	-0.390545000
H	3.989427000	1.206388000	1.421328000
H	3.146379000	0.593246000	-1.401969000
H	5.258319000	-0.523933000	-1.822427000
H	5.945872000	-1.573112000	-0.582931000
H	6.255874000	1.157308000	-0.426145000
H	6.004258000	0.220186000	1.031472000
H	1.757806000	-0.137771000	1.166874000
H	0.988687000	-2.685746000	-1.582470000
H	1.541356000	-2.643224000	0.070334000
H	3.084063000	-1.566100000	-2.328131000
H	3.441311000	-2.993385000	-1.376510000
H	1.079066000	2.110038000	-0.755890000
H	1.661177000	2.280384000	0.878526000
H	-0.815994000	2.564211000	0.657110000
H	-0.317599000	1.389494000	1.842567000
H	-1.336429000	0.832685000	-0.963548000
H	-3.281454000	-0.112222000	-1.373702000
H	-3.460823000	-2.553645000	-0.876498000
H	-3.008803000	-2.237962000	0.773642000
H	-1.437689000	-1.663093000	-1.760551000
H	-1.044642000	-2.881303000	-0.563314000
H	-2.994502000	2.279923000	-0.692986000
H	-2.878489000	2.660247000	1.016317000
H	-5.162936000	3.030244000	0.128364000
H	-5.173783000	1.845172000	1.410977000
H	-5.202677000	1.284814000	-1.567493000
H	4.329722000	4.677531000	0.413459000

H	4.218942000	3.472637000	1.811888000
H	3.262588000	2.695444000	-2.049953000
H	4.521908000	3.910499000	-1.830585000
H	4.947561000	2.213100000	-2.101545000
H	4.252440000	-3.378467000	1.843208000
H	0.166937000	0.800028000	-2.175005000
H	1.630535000	-0.033333000	-2.613967000
H	0.102414000	-0.857851000	-2.748506000
H	-1.250401000	-1.864365000	1.861940000
H	0.425116000	-2.185157000	1.509837000
H	-0.043487000	-0.645918000	2.194726000
H	-2.011795000	0.571184000	2.621403000
H	-3.738419000	0.777406000	2.492087000
H	-3.038989000	-0.811392000	2.288468000
H	-5.165515000	-1.039564000	-2.372790000
H	-5.494757000	-2.344005000	-1.226065000
H	-6.709476000	-1.127324000	-1.512586000
H	-6.729057000	-1.042336000	0.935358000
H	-5.245069000	-1.933579000	1.261319000
H	-5.452178000	-0.297614000	1.880986000
H	-7.354271000	0.790291000	-1.109138000

Icajine (Conformer 1, E = -1187.8836583 Hartree), solvent: Chloroform

O	-0.147519000	-2.873358000	1.215584000
O	2.090735000	3.009180000	0.724947000
O	-2.510683000	3.210035000	-0.444251000
N	-1.439683000	1.201479000	-0.495188000
N	2.082377000	-2.088405000	-0.569240000
C	-0.150906000	0.515786000	-0.308144000
C	0.200253000	-1.742634000	0.964453000

C	1.355448000	-1.733591000	-1.777232000
C	-0.149959000	-1.750330000	-1.501663000
C	-0.494518000	-0.993100000	-0.198998000
C	-2.002806000	-0.965679000	-0.013937000
C	-2.869722000	-2.004640000	0.266543000
C	-4.237724000	-1.747707000	0.318281000
C	-4.720209000	-0.468045000	0.082051000
C	-3.861282000	0.587930000	-0.206915000
C	-2.501368000	0.316624000	-0.245777000
C	1.199280000	-1.034971000	1.864616000
C	1.745311000	0.287510000	1.315520000
C	0.509679000	1.104878000	0.931921000
C	0.732352000	2.626849000	0.773007000
C	2.863935000	2.646129000	-0.392879000
C	3.172143000	1.187552000	-0.551948000
C	2.733888000	0.151139000	0.155489000
C	3.204552000	-1.237344000	-0.213147000
C	-0.100861000	3.192313000	-0.394628000
C	-1.481796000	2.567182000	-0.447352000
C	2.323431000	-3.505915000	-0.420914000
H	0.474200000	0.697738000	-1.179758000
H	1.592871000	-2.415959000	-2.600618000
H	1.667923000	-0.740169000	-2.095940000
H	-0.505070000	-2.775096000	-1.384331000
H	-0.697505000	-1.307322000	-2.335603000
H	-2.481413000	-2.995792000	0.449901000
H	-4.925901000	-2.551073000	0.540097000
H	-5.784577000	-0.281334000	0.121615000
H	-4.228316000	1.583836000	-0.390357000
H	1.974890000	-1.760095000	2.105079000

H	0.676869000	-0.821842000	2.800818000
H	2.260709000	0.800688000	2.128847000
H	-0.219144000	0.985193000	1.739766000
H	0.380716000	3.105024000	1.685457000
H	3.805210000	3.186051000	-0.275106000
H	2.422586000	3.021431000	-1.323290000
H	3.860299000	0.991038000	-1.369286000
H	3.694420000	-1.697190000	0.648665000
H	3.956393000	-1.174692000	-1.010589000
H	-0.212162000	4.268633000	-0.309719000
H	0.388163000	2.969893000	-1.344153000
H	3.087401000	-3.877311000	-1.115825000
H	1.396079000	-4.050203000	-0.594862000
H	2.641513000	-3.719439000	0.599859000

Icajine (Conformer 2, *E* = -1187.8847939 Hartree), solvent: Chloroform

O	-0.196570000	-2.760506000	1.281833000
O	1.911221000	2.859611000	-0.535925000
O	-2.718799000	3.145159000	-0.027644000
N	-1.521819000	1.247422000	-0.369902000
N	2.070008000	-2.116098000	-0.478441000
C	-0.197731000	0.589961000	-0.342378000
C	0.165196000	-1.645128000	0.987514000
C	1.416208000	-1.692189000	-1.704666000
C	-0.095560000	-1.700497000	-1.497623000
C	-0.499523000	-0.934478000	-0.218037000
C	-2.009596000	-0.970901000	-0.079610000
C	-2.838390000	-2.064699000	0.088497000
C	-4.215788000	-1.868676000	0.130181000
C	-4.744236000	-0.592217000	-0.004249000

C	-3.923313000	0.516698000	-0.179867000
C	-2.551193000	0.304033000	-0.211791000
C	1.135214000	-0.896822000	1.884226000
C	1.771621000	0.353198000	1.266687000
C	0.598456000	1.225746000	0.791873000
C	0.895060000	2.686348000	0.429381000
C	3.218563000	2.519650000	-0.114046000
C	3.526183000	1.068840000	-0.349494000
C	2.863744000	0.076676000	0.234835000
C	3.232295000	-1.352338000	-0.074162000
C	-0.346822000	3.367939000	-0.173870000
C	-1.646493000	2.590697000	-0.165438000
C	2.218764000	-3.547974000	-0.349311000
H	0.317497000	0.788888000	-1.284433000
H	1.753958000	-0.685778000	-1.950861000
H	1.687545000	-2.334366000	-2.550421000
H	-0.458179000	-2.723881000	-1.392407000
H	-0.606318000	-1.260136000	-2.355613000
H	-2.414195000	-3.052009000	0.196660000
H	-4.874728000	-2.714838000	0.264535000
H	-5.815825000	-0.450131000	0.025744000
H	-4.328010000	1.508547000	-0.284345000
H	0.565375000	-0.590751000	2.765749000
H	1.868884000	-1.623329000	2.228878000
H	2.259427000	0.900951000	2.080239000
H	-0.082408000	1.283565000	1.648885000
H	1.194742000	3.206527000	1.345445000
H	3.344697000	2.777890000	0.944644000
H	3.895492000	3.146872000	-0.689212000
H	4.312157000	0.833419000	-1.057423000

H	3.629970000	-1.825888000	0.827450000
H	4.035852000	-1.369684000	-0.822641000
H	-0.551646000	4.325942000	0.293812000
H	-0.114068000	3.563013000	-1.222791000
H	2.981125000	-3.952122000	-1.027561000
H	1.267605000	-4.033794000	-0.560736000
H	2.490971000	-3.795498000	0.676778000

Iguesterin (*E* = -1238.9729571 Hartree), solvent: Chloroform

O	-6.509497000	0.829373000	-0.984707000
O	-5.245471000	2.649104000	0.408812000
C	1.660503000	0.207503000	0.443478000
C	1.049873000	-1.237437000	0.550743000
C	3.203588000	0.144765000	0.640036000
C	3.966132000	-0.890725000	-0.238845000
C	1.732883000	-2.149615000	-0.484845000
C	1.016211000	1.049375000	1.558961000
C	3.242674000	-2.243077000	-0.274584000
C	-0.455264000	-1.129348000	0.308671000
C	-1.276969000	-0.090067000	1.067196000
C	-0.492436000	1.207475000	1.376229000
C	3.897420000	1.516600000	0.537941000
C	1.289309000	0.859386000	-0.900949000
C	4.184316000	-0.417475000	-1.689336000
C	1.285067000	-1.893699000	1.932921000
C	5.354012000	-1.109207000	0.380662000
C	4.374091000	1.915377000	-0.833797000
C	-2.604460000	0.241323000	0.391189000
C	4.503394000	1.038643000	-1.821782000
C	-1.097895000	-1.961518000	-0.528846000

C	-1.734213000	-0.742210000	2.413800000
C	-3.244476000	-0.768236000	-0.441086000
C	-2.496570000	-1.829808000	-0.832755000
C	4.713230000	3.368174000	-0.993593000
C	-3.263445000	1.386740000	0.671545000
C	-4.614368000	-0.592748000	-0.899986000
C	-4.608991000	1.621536000	0.192117000
C	-5.249751000	0.554068000	-0.593783000
C	-5.286249000	-1.652828000	-1.722943000
H	3.341221000	-0.186290000	1.672858000
H	1.321442000	-3.157100000	-0.412646000
H	1.510784000	-1.794285000	-1.492273000
H	1.253792000	0.626286000	2.534251000
H	1.433407000	2.054907000	1.560239000
H	3.677822000	-2.841720000	-1.079150000
H	3.453244000	-2.791305000	0.644037000
H	-0.665954000	1.913797000	0.565908000
H	-0.912247000	1.664764000	2.272439000
H	3.248187000	2.307173000	0.914577000
H	4.764269000	1.531352000	1.206620000
H	0.217709000	0.811865000	-1.090401000
H	1.576768000	1.911734000	-0.891507000
H	1.783008000	0.395773000	-1.746160000
H	5.009845000	-1.004306000	-2.104273000
H	3.325877000	-0.667671000	-2.317839000
H	2.342307000	-2.046957000	2.126070000
H	0.897036000	-1.319647000	2.762770000
H	0.797675000	-2.869232000	1.945247000
H	5.273672000	-1.354464000	1.440985000
H	5.981972000	-0.224476000	0.276175000

H	5.857978000	-1.937199000	-0.120468000
H	4.857664000	1.380658000	-2.788981000
H	-0.568939000	-2.751063000	-1.038713000
H	-0.897746000	-0.892804000	3.086192000
H	-2.218716000	-1.700422000	2.231594000
H	-2.444707000	-0.073105000	2.896123000
H	-2.939690000	-2.586559000	-1.466345000
H	5.447818000	3.676618000	-0.246273000
H	3.826776000	3.987386000	-0.837337000
H	5.114583000	3.580929000	-1.982289000
H	-2.839998000	2.171771000	1.280965000
H	-5.273317000	-2.614456000	-1.209748000
H	-4.781840000	-1.784335000	-2.681090000
H	-6.318544000	-1.379372000	-1.917093000
H	-6.699105000	1.716142000	-0.637462000

Itoaic Acid (Conformer 1, *E* = -1623.3690218 Hartree), solvent: Chloroform

C	-3.415945000	-1.103171000	-0.276233000
C	-2.561805000	0.165002000	0.055581000
C	-1.258550000	-0.110233000	0.901584000
C	-0.419906000	-1.136089000	0.061618000
C	-1.215198000	-2.426979000	-0.126911000
C	-2.494723000	-2.159597000	-0.907020000
C	-0.435376000	1.206920000	1.011661000
C	1.055104000	1.009093000	1.309937000
C	1.780339000	0.046890000	0.354986000
C	1.065802000	-1.344931000	0.480246000
C	3.298175000	-0.066639000	0.753993000
C	4.093361000	-1.285666000	0.184118000
C	3.239841000	-2.556490000	-0.047582000
C	1.772533000	-2.366139000	-0.431466000

C	4.131075000	1.249008000	0.657170000
C	5.083762000	1.523242000	-0.539387000
C	5.775503000	0.234637000	-0.996382000
C	4.803592000	-0.928532000	-1.135495000
C	-3.383255000	1.341576000	0.598978000
C	-4.459267000	1.915775000	-0.333328000
C	-5.716777000	1.070176000	-0.296607000
O	-5.667025000	-0.168921000	-0.756769000
C	-4.477672000	-0.734364000	-1.352224000
O	-4.786526000	3.217925000	0.070721000
O	-6.739352000	1.493856000	0.176336000
C	-5.022158000	-1.892588000	-2.173285000
C	-4.148297000	-1.711006000	0.928177000
C	-1.605789000	-0.577838000	2.330936000
O	-0.944765000	2.123414000	1.971041000
C	1.166858000	-1.916015000	1.908800000
C	5.156899000	-1.660000000	1.231991000
C	6.141796000	2.516889000	-0.047718000
C	4.400603000	2.167340000	-1.752155000
C	1.685655000	0.615917000	-1.063972000
O	1.516273000	1.948561000	-1.071776000
O	1.782631000	0.016742000	-2.102919000
H	-2.179131000	0.499669000	-0.917520000
H	-0.362930000	-0.698142000	-0.941803000
H	-1.437271000	-2.903925000	0.828391000
H	-0.635016000	-3.146608000	-0.698724000
H	-3.047123000	-3.091504000	-1.030199000
H	-2.208934000	-1.824320000	-1.909322000
H	-0.529111000	1.731917000	0.060810000
H	1.169208000	0.636639000	2.328821000

H	1.501947000	1.998905000	1.295633000
H	3.221182000	-0.246069000	1.828091000
H	3.278441000	-3.172542000	0.850028000
H	3.729108000	-3.150849000	-0.821673000
H	1.293443000	-3.337579000	-0.317584000
H	1.670977000	-2.075220000	-1.471952000
H	4.764340000	1.255759000	1.545689000
H	3.475208000	2.113388000	0.767948000
H	6.564237000	-0.031014000	-0.290318000
H	6.273550000	0.422949000	-1.950649000
H	5.350455000	-1.813753000	-1.467332000
H	4.058421000	-0.717766000	-1.903692000
H	-2.722794000	2.180392000	0.783468000
H	-3.850078000	1.114305000	1.559384000
H	-4.077248000	1.953838000	-1.357403000
H	-4.048465000	-0.003599000	-2.040312000
H	-5.703439000	3.194067000	0.374073000
H	-4.260884000	-2.305312000	-2.827894000
H	-5.407638000	-2.685349000	-1.534459000
H	-5.839981000	-1.522976000	-2.787647000
H	-4.642097000	-0.958219000	1.539994000
H	-4.918354000	-2.400284000	0.582964000
H	-3.471818000	-2.272151000	1.564693000
H	-2.565559000	-0.178934000	2.649952000
H	-0.871490000	-0.237332000	3.059355000
H	-1.647837000	-1.658052000	2.425241000
H	-0.783496000	1.779040000	2.852854000
H	0.644383000	-2.873369000	1.936500000
H	0.734850000	-1.288314000	2.674469000
H	2.195790000	-2.107319000	2.197934000

H	5.790926000	-0.820163000	1.509115000
H	4.676378000	-2.024315000	2.140958000
H	5.798799000	-2.455590000	0.848302000
H	6.850970000	2.744521000	-0.845047000
H	5.678002000	3.452735000	0.270297000
H	6.698881000	2.108689000	0.796568000
H	5.166951000	2.480769000	-2.462791000
H	3.741095000	1.478399000	-2.275335000
H	3.832496000	3.049575000	-1.457955000
H	1.543680000	2.235243000	-1.995050000

Itoaic Acid (Conformer 2, *E* = -1623.3690375 Hartree), solvent: Chloroform

C	-3.487063000	-1.100954000	0.154514000
C	-2.634729000	0.205336000	0.011215000
C	-1.240552000	0.176499000	0.751116000
C	-0.489072000	-1.058912000	0.147678000
C	-1.262227000	-2.339131000	0.459751000
C	-2.617048000	-2.310770000	-0.231628000
C	-0.420964000	1.448448000	0.388253000
C	1.081525000	1.318942000	0.654558000
C	1.740554000	0.135054000	-0.070670000
C	1.040725000	-1.177798000	0.403354000
C	3.276446000	0.063643000	0.258555000
C	3.974225000	-1.277418000	-0.199095000
C	3.017362000	-2.176638000	-0.992884000
C	1.625082000	-2.392309000	-0.377880000
C	4.060010000	1.300026000	-0.212012000
C	5.561572000	1.273740000	0.116567000
C	6.190544000	-0.049009000	-0.404349000
C	5.169544000	-0.938190000	-1.107442000

C	-3.426699000	1.492025000	0.279982000
C	-4.608790000	1.779164000	-0.656111000
C	-5.833795000	0.987278000	-0.244780000
O	-5.800034000	-0.332059000	-0.331515000
C	-4.664800000	-1.053147000	-0.861703000
O	-4.924895000	3.144546000	-0.609600000
O	-6.813037000	1.534974000	0.191314000
C	-5.263916000	-2.396152000	-1.248512000
C	-4.074694000	-1.319572000	1.555958000
C	-1.409892000	0.154950000	2.284834000
O	-0.867298000	2.629945000	1.038267000
C	1.292935000	-1.431394000	1.902651000
C	4.519374000	-2.092444000	0.992843000
C	5.792360000	1.430812000	1.621394000
C	6.211833000	2.463673000	-0.593356000
C	1.498077000	0.384745000	-1.571885000
O	1.729446000	1.662268000	-1.924245000
O	1.106829000	-0.402100000	-2.391532000
H	-2.366588000	0.243818000	-1.052551000
H	-0.574463000	-0.933536000	-0.933560000
H	-1.388255000	-2.482384000	1.532392000
H	-0.712773000	-3.209077000	0.103021000
H	-3.163579000	-3.228416000	-0.012342000
H	-2.443197000	-2.292030000	-1.312243000
H	-0.570196000	1.649359000	-0.677125000
H	1.256359000	1.219997000	1.726724000
H	1.528280000	2.260847000	0.351479000
H	3.298223000	0.088389000	1.349087000
H	3.500135000	-3.148685000	-1.120106000
H	2.901701000	-1.767339000	-1.993097000

H	1.651997000	-3.247924000	0.298661000
H	0.952984000	-2.662339000	-1.190742000
H	3.629898000	2.200215000	0.229718000
H	3.960464000	1.418161000	-1.288928000
H	6.632804000	-0.601281000	0.426621000
H	7.009983000	0.176133000	-1.088418000
H	5.639194000	-1.871460000	-1.425626000
H	4.812140000	-0.449351000	-2.016924000
H	-2.772045000	2.343915000	0.142372000
H	-3.783972000	1.553855000	1.309811000
H	-4.336533000	1.518524000	-1.682739000
H	-4.328517000	-0.556977000	-1.774092000
H	-5.805852000	3.214019000	-0.219217000
H	-4.565964000	-2.986992000	-1.833339000
H	-5.560758000	-2.965772000	-0.369531000
H	-6.150464000	-2.215042000	-1.851908000
H	-4.512968000	-0.413754000	1.970730000
H	-4.864553000	-2.068987000	1.513635000
H	-3.323426000	-1.678358000	2.251989000
H	-2.319406000	0.670629000	2.584787000
H	-0.585060000	0.655955000	2.790238000
H	-1.449216000	-0.848544000	2.694813000
H	-0.655381000	2.570897000	1.973382000
H	0.726349000	-2.310368000	2.208341000
H	0.991529000	-0.611278000	2.543045000
H	2.334460000	-1.639734000	2.112372000
H	4.919053000	-1.464646000	1.787851000
H	3.758693000	-2.738191000	1.424886000
H	5.324600000	-2.743588000	0.648652000
H	5.334705000	0.624423000	2.193335000

H	6.861011000	1.426457000	1.841466000
H	5.377021000	2.375246000	1.977995000
H	6.096392000	2.376232000	-1.675029000
H	5.756840000	3.404014000	-0.276978000
H	7.278751000	2.507891000	-0.369250000
H	1.543966000	1.733488000	-2.870675000

Matopensine (*E* = -1767.3721344 Hartree), solvent: Chloroform

O	-0.000012000	-0.000015000	2.588380000
N	-1.334465000	-0.890806000	0.943709000
N	-3.824770000	2.172287000	-1.043348000
C	-2.077485000	0.346131000	1.227651000
C	-3.335034000	0.226368000	0.331596000
C	-1.131474000	1.539259000	1.041222000
C	-0.196226000	-1.156482000	1.797664000
C	-3.112495000	0.889266000	-1.068988000
C	-0.969655000	1.985002000	-0.446216000
C	-1.642688000	1.005370000	-1.424904000
C	-4.582960000	0.956334000	0.871840000
C	-3.462759000	-1.277521000	0.232817000
C	-2.256680000	-1.872525000	0.622076000
C	-5.044847000	1.835217000	-0.308966000
C	-1.657108000	3.306269000	-0.710012000
C	-3.121645000	3.281645000	-0.370261000
C	-4.520725000	-2.060852000	-0.170192000
C	-2.105292000	-3.250104000	0.645877000
C	-1.117098000	4.366970000	-1.305794000
C	-4.382594000	-3.452928000	-0.162683000
C	-3.191363000	-4.030722000	0.246642000
C	0.279741000	4.539589000	-1.818259000

H	-2.411682000	0.327813000	2.272124000
H	-1.547875000	2.372963000	1.605106000
H	-0.443407000	-1.948841000	2.509588000
H	-3.626050000	0.268113000	-1.806714000
H	0.084533000	2.054533000	-0.690586000
H	-1.534004000	1.394901000	-2.437354000
H	-1.184578000	0.017998000	-1.389014000
H	-4.316937000	1.571145000	1.732330000
H	-5.351967000	0.260164000	1.199743000
H	-5.701534000	1.263087000	-0.966887000
H	-5.573161000	2.735332000	0.000023000
H	-3.595652000	4.211834000	-0.682069000
H	-3.241439000	3.216964000	0.719996000
H	-5.455823000	-1.606216000	-0.475456000
H	-1.170351000	-3.709929000	0.942405000
H	-1.773039000	5.215441000	-1.479519000
H	-5.208809000	-4.078679000	-0.468574000
H	-3.095740000	-5.108165000	0.256330000
H	0.247838000	4.864679000	-2.859098000
H	0.810866000	5.314472000	-1.262062000
H	0.870961000	3.630315000	-1.764761000
N	1.334462000	0.890797000	0.943737000
N	3.824771000	-2.172268000	-1.043362000
C	2.077476000	-0.346147000	1.227665000
C	3.335031000	-0.226375000	0.331619000
C	1.131465000	-1.539271000	1.041213000
C	0.196211000	1.156462000	1.797681000
C	3.112496000	-0.889246000	-1.068980000
C	0.969655000	-1.984992000	-0.446231000
C	1.642691000	-1.005346000	-1.424903000

C	4.582950000	-0.956359000	0.871855000
C	3.462765000	1.277514000	0.232872000
C	2.256687000	1.872518000	0.622138000
C	5.044844000	-1.835213000	-0.308971000
C	1.657107000	-3.306257000	-0.710040000
C	3.121647000	-3.281639000	-0.370295000
C	4.520740000	2.060848000	-0.170110000
C	2.105310000	3.250097000	0.645974000
C	1.117088000	-4.366954000	-1.305820000
C	4.382619000	3.452924000	-0.162566000
C	3.191390000	4.030717000	0.246767000
C	-0.279761000	-4.539566000	-1.818262000
H	2.411667000	-0.327845000	2.272140000
H	1.547861000	-2.372983000	1.605089000
H	0.443384000	1.948812000	2.509619000
H	3.626053000	-0.268080000	-1.806692000
H	-0.084532000	-2.054521000	-0.690605000
H	1.534010000	-1.394862000	-2.437359000
H	1.184581000	-0.017975000	-1.389000000
H	4.316919000	-1.571190000	1.732327000
H	5.351957000	-0.260199000	1.199781000
H	5.701533000	-1.263065000	-0.966876000
H	5.573159000	-2.735333000	0.000000000
H	3.595652000	-4.211823000	-0.682122000
H	3.241446000	-3.216977000	0.719962000
H	5.455837000	1.606213000	-0.475377000
H	1.170372000	3.709921000	0.942509000
H	1.773024000	-5.215426000	-1.479559000
H	5.208841000	4.078678000	-0.468435000
H	3.095776000	5.108161000	0.256483000

H	-0.247877000	-4.864629000	-2.859110000
H	-0.810870000	-5.314467000	-1.262074000
H	-0.870984000	-3.630297000	-1.764726000

Naucleidinal (*E* = -1109.2960951 Hartree), solvent: Chloroform

O	-3.453255000	1.451885000	1.330558000
O	-1.243723000	-2.000252000	1.030680000
O	-6.788990000	-0.027003000	-1.565205000
N	-3.009104000	-3.205660000	0.295124000
N	-5.074925000	-2.960422000	-2.741464000
C	-4.343302000	-3.270287000	-0.306034000
C	-4.563791000	-0.806440000	-0.098507000
C	-5.241651000	-2.141174000	0.167643000
C	-5.393814000	0.390777000	0.337073000
C	-4.139220000	-3.309935000	-1.795452000
C	-3.251150000	-0.806460000	0.632886000
C	-2.140370000	-4.298421000	-0.138444000
C	-2.980216000	-3.702710000	-2.394504000
C	-2.411500000	-2.030563000	0.674249000
C	-1.769052000	-4.128882000	-1.619943000
C	-4.501045000	1.642679000	0.375868000
C	-3.188970000	-3.576839000	-3.808896000
C	-2.825505000	0.266498000	1.302350000
C	-4.507965000	-3.098798000	-3.987503000
C	-6.593256000	0.596945000	-0.557095000
C	-5.210492000	2.918258000	0.771079000
C	-2.393953000	-3.815434000	-4.935430000
C	-5.038880000	-2.845195000	-5.249807000
C	-2.918637000	-3.568003000	-6.188105000
C	-4.228248000	-3.085120000	-6.342323000

H	-4.786892000	-4.216026000	0.017826000
H	-4.392663000	-0.720980000	-1.175687000
H	-6.204801000	-2.208427000	-0.336415000
H	-5.417943000	-2.256669000	1.238677000
H	-5.776737000	0.248882000	1.355463000
H	-2.689670000	-5.229070000	0.004564000
H	-1.257485000	-4.310920000	0.490428000
H	-1.366302000	-5.068585000	-2.000803000
H	-0.977768000	-3.381232000	-1.714183000
H	-4.028067000	1.754220000	-0.604954000
H	-5.947138000	-2.492791000	-2.553052000
H	-1.922828000	0.252035000	1.896617000
H	-7.314960000	1.363981000	-0.230155000
H	-5.902664000	3.235640000	-0.006024000
H	-5.760915000	2.777142000	1.700715000
H	-4.476004000	3.707140000	0.916388000
H	-1.382886000	-4.184806000	-4.823051000
H	-6.048045000	-2.474792000	-5.368457000
H	-2.315137000	-3.745975000	-7.067342000
H	-4.610278000	-2.898712000	-7.336452000

Physalin D (*E* = -1913.6729663 Hartree), solvent: Dimethylsulfoxide

C	-6.151202000	0.484687000	-0.602816000
C	-5.532681000	-0.864729000	-0.409236000
C	-4.012806000	-0.822391000	-0.553250000
C	-3.389954000	0.344622000	0.271008000
C	-3.976183000	1.609383000	-0.368107000
C	-5.444620000	1.613819000	-0.581963000
C	-3.390248000	-2.173382000	-0.210538000
C	-1.878422000	-2.131964000	-0.348990000

C	-1.245944000	-1.026174000	0.505302000
C	-1.844282000	0.351377000	0.136596000
O	-3.307976000	2.562163000	-0.701173000
C	0.300962000	-1.061110000	0.383561000
C	1.111775000	-0.778495000	1.667118000
C	2.361677000	-0.038560000	1.223965000
C	1.770023000	0.774421000	0.067221000
C	1.211130000	2.133448000	0.586408000
C	-0.229575000	2.405471000	0.172965000
C	-1.223099000	1.513919000	0.941199000
O	0.809023000	-1.122733000	2.770397000
O	0.778178000	-0.072061000	-0.513253000
O	0.665877000	-2.328240000	-0.085512000
C	3.530372000	-0.976054000	0.841420000
C	3.149124000	-2.136837000	-0.104758000
C	1.899835000	-2.926894000	0.279614000
C	4.565830000	-0.108293000	0.129033000
C	4.008010000	0.363103000	-1.194024000
C	2.735691000	1.214822000	-1.069777000
C	2.225690000	3.142757000	0.044888000
O	3.172684000	2.530591000	-0.678228000
O	2.250668000	4.308297000	0.297584000
C	3.119670000	-1.843962000	-1.598018000
O	3.727980000	-0.741828000	-2.058469000
C	2.043297000	1.351573000	-2.419319000
O	2.661781000	-2.617889000	-2.388858000
C	4.118413000	-1.543932000	2.133476000
O	-3.672807000	-0.638804000	-1.928331000
O	-3.789200000	-2.532736000	1.101160000
C	-3.838763000	0.334080000	1.749186000

O	1.323477000	2.224639000	1.996653000
H	-7.217578000	0.521909000	-0.789501000
H	-5.927410000	-1.561329000	-1.152151000
H	-5.813802000	-1.267223000	0.564680000
H	-5.894822000	2.578671000	-0.774590000
H	-3.794676000	-2.890406000	-0.932385000
H	-1.622669000	-1.981859000	-1.398315000
H	-1.487890000	-3.104609000	-0.059968000
H	-1.459066000	-1.242975000	1.556015000
H	-1.634283000	0.515035000	-0.920170000
H	2.709302000	0.595428000	2.030907000
H	-0.360085000	2.285312000	-0.900028000
H	-0.419817000	3.456243000	0.399493000
H	-2.009602000	2.168111000	1.296268000
H	-0.749433000	1.128423000	1.845781000
H	3.976003000	-2.852164000	-0.030670000
H	1.915573000	-3.145835000	1.348836000
H	1.904370000	-3.870804000	-0.255493000
H	4.827747000	0.753986000	0.743462000
H	5.477350000	-0.679065000	-0.053065000
H	4.737819000	0.960877000	-1.736318000
H	2.772921000	1.707352000	-3.145183000
H	1.236944000	2.079565000	-2.356573000
H	1.635611000	0.405678000	-2.756800000
H	4.466443000	-0.731953000	2.771119000
H	3.383003000	-2.117315000	2.696688000
H	4.965376000	-2.191936000	1.910734000
H	-4.296681000	-0.029790000	-2.334925000
H	-3.457835000	-3.415328000	1.286475000
H	-3.559721000	1.264003000	2.239369000

H	-4.916830000	0.233340000	1.840677000
H	-3.379882000	-0.491181000	2.283982000
H	1.073810000	3.120403000	2.252908000

Strychnobaillonine (Conformer 1, *E* = -1920.1949285 Hartree), solvent: Chloroform

O	1.191530000	2.833659000	1.742581000
O	-1.251845000	3.442388000	0.832065000
N	-2.377754000	1.612154000	0.111370000
N	-4.157948000	-2.420213000	-1.234413000
N	1.066865000	-0.642608000	0.383993000
N	5.066934000	-0.122728000	-1.545029000
C	-5.472225000	3.169472000	1.495634000
C	-6.375368000	2.224644000	1.026593000
C	-5.924911000	1.143377000	0.275447000
C	-4.574498000	1.024929000	0.005045000
C	-3.682582000	1.976478000	0.483318000
C	-4.110245000	3.065185000	1.232166000
C	-3.874568000	-0.053553000	-0.781609000
C	-2.385343000	0.432528000	-0.787766000
C	-1.247596000	2.324958000	0.338247000
C	0.016749000	1.566843000	-0.022748000
C	-0.175584000	0.111165000	0.463239000
C	-1.240228000	-0.565307000	-0.427452000
C	-4.485835000	-0.250965000	-2.196903000
C	-5.005201000	-1.706935000	-2.190562000
C	-4.073104000	-1.460208000	-0.131778000
C	-3.012000000	-1.791982000	0.895367000
C	-1.664245000	-1.922012000	0.187509000
C	0.167424000	-4.457810000	-0.108144000
C	-1.005426000	-4.074821000	-0.958233000

C	-1.781068000	-2.998533000	-0.859681000
C	-2.862647000	-2.723493000	-1.862070000
C	1.299550000	2.281270000	0.447755000
C	2.481225000	1.311349000	0.506931000
C	2.205386000	0.021228000	-0.265187000
C	1.592693000	-1.406416000	1.422403000
C	3.357335000	-1.003627000	-0.121267000
C	2.944078000	-1.688199000	1.161888000
C	0.982511000	-1.881922000	2.574877000
C	1.733032000	-2.681974000	3.437712000
C	3.055057000	-2.994704000	3.167830000
C	3.668499000	-2.485400000	2.019362000
C	3.851665000	1.930096000	0.196032000
C	4.902387000	0.929192000	0.677902000
C	4.758725000	-0.340940000	-0.131417000
C	4.676580000	-1.395205000	-2.159739000
C	3.457712000	-1.939886000	-1.351008000
C	4.353987000	1.009395000	-2.166444000
C	4.105686000	2.210243000	-1.267434000
C	4.113956000	3.429976000	-1.802594000
C	3.799955000	4.734260000	-1.134264000
H	-3.410344000	3.800590000	1.590012000
H	-2.983164000	-0.998726000	1.648293000
H	-5.055905000	-1.452274000	0.344447000
H	-6.036129000	-1.739124000	-1.835780000
H	-5.280795000	0.465149000	-2.390885000
H	-2.999802000	-3.576380000	-2.525512000
H	-6.624812000	0.405964000	-0.098659000
H	-7.429043000	2.330520000	1.242209000
H	-5.829673000	4.008680000	2.076074000

H	0.065345000	1.533754000	-1.117220000
H	-0.515925000	0.134813000	1.503284000
H	1.063229000	-4.546401000	-0.725598000
H	0.381973000	-3.745043000	0.683197000
H	-2.174246000	0.810517000	-1.789750000
H	-0.906157000	-2.214857000	0.904710000
H	-2.526388000	-1.882461000	-2.491687000
H	-1.229440000	-4.766905000	-1.765533000
H	-3.729423000	-0.108435000	-2.966723000
H	-4.967709000	-2.179660000	-3.170104000
H	-3.260116000	-2.724474000	1.401035000
H	-0.715032000	-0.776260000	-1.361003000
H	0.003658000	-5.435379000	0.347997000
H	1.505599000	3.075751000	-0.279650000
H	0.431469000	3.429235000	1.727416000
H	3.005736000	5.250062000	-1.676204000
H	3.476440000	4.620615000	-0.103114000
H	4.669165000	5.393884000	-1.149702000
H	4.366585000	3.508294000	-2.856836000
H	4.933255000	1.333796000	-3.031214000
H	3.387412000	0.675163000	-2.564401000
H	4.758503000	0.714591000	1.737523000
H	5.909479000	1.324066000	0.542779000
H	3.937754000	2.851096000	0.769137000
H	2.525340000	1.010996000	1.558408000
H	1.964453000	0.214305000	-1.308168000
H	-0.045345000	-1.646518000	2.813785000
H	1.266591000	-3.059961000	4.337324000
H	3.615111000	-3.618261000	3.849712000
H	4.707222000	-2.711663000	1.812173000

H	3.639364000	-2.965541000	-1.034849000
H	5.509587000	-2.093148000	-2.077491000
H	2.530525000	-1.933507000	-1.923513000
H	4.461859000	-1.257658000	-3.217364000
H	5.470517000	-1.090007000	0.229097000

Strychnobaillonine (Conformer 2, *E* = -1920.1922409 Hartree), solvent: Chloroform

O	1.238431000	2.711645000	1.809338000
O	-1.113245000	3.435284000	0.745298000
N	-2.306940000	1.611994000	0.125679000
N	-4.632503000	-2.395792000	-0.259315000
N	1.114274000	-0.744071000	0.323849000
N	5.199562000	-0.147222000	-1.448171000
C	-5.377306000	3.612946000	0.849077000
C	-6.307326000	2.678932000	0.414301000
C	-5.883704000	1.448102000	-0.079097000
C	-4.530194000	1.174008000	-0.139860000
C	-3.609363000	2.124292000	0.293431000
C	-4.011080000	3.354058000	0.796658000
C	-3.833178000	-0.092544000	-0.578328000
C	-2.366288000	0.391525000	-0.711053000
C	-1.151134000	2.288880000	0.323828000
C	0.093776000	1.483195000	-0.000680000
C	-0.118166000	0.025589000	0.460834000
C	-1.226640000	-0.612660000	-0.406778000
C	-4.367885000	-0.684721000	-1.895604000
C	-5.282571000	-1.820238000	-1.431870000
C	-4.081234000	-1.248031000	0.476250000
C	-2.814553000	-1.626571000	1.230016000
C	-1.717578000	-1.937396000	0.206877000

C	-0.032633000	-3.207093000	-2.042561000
C	-1.491970000	-3.454194000	-1.796542000
C	-2.233455000	-2.939139000	-0.819104000
C	-3.638720000	-3.429788000	-0.562027000
C	1.374797000	2.174575000	0.510692000
C	2.538476000	1.188448000	0.586574000
C	2.276186000	-0.055325000	-0.260522000
C	1.611998000	-1.592078000	1.310886000
C	3.418839000	-1.096502000	-0.155847000
C	2.967137000	-1.865797000	1.064056000
C	0.972614000	-2.140295000	2.415062000
C	1.700781000	-2.993274000	3.245153000
C	3.029147000	-3.291169000	2.989680000
C	3.669994000	-2.715726000	1.889150000
C	3.926659000	1.805723000	0.365125000
C	4.946803000	0.765355000	0.826765000
C	4.826416000	-0.449295000	-0.066628000
C	4.830912000	-1.378520000	-2.152063000
C	3.556063000	-1.940783000	-1.449454000
C	4.508267000	1.019854000	-2.027183000
C	4.236458000	2.168354000	-1.069268000
C	4.267116000	3.417096000	-1.531547000
C	3.929888000	4.680565000	-0.798907000
H	-3.288376000	4.080171000	1.126488000
H	-2.514783000	-0.799251000	1.875999000
H	-4.842665000	-0.893276000	1.172329000
H	-6.255630000	-1.424315000	-1.132642000
H	-4.870026000	0.065719000	-2.503948000
H	-3.594187000	-4.107303000	0.296243000
H	-6.608722000	0.716691000	-0.411291000

H	-7.362957000	2.906245000	0.460119000
H	-5.714909000	4.565506000	1.233271000
H	0.164380000	1.464199000	-1.094861000
H	-0.417859000	0.039336000	1.513868000
H	0.454672000	-2.727474000	-1.195291000
H	0.476710000	-4.150796000	-2.239335000
H	-2.249600000	0.732517000	-1.743504000
H	-0.862408000	-2.398407000	0.699758000
H	-3.996052000	-4.022210000	-1.402604000
H	-1.970789000	-4.160926000	-2.468232000
H	-3.539549000	-1.098888000	-2.472751000
H	-5.454395000	-2.579098000	-2.191894000
H	-3.013690000	-2.480791000	1.876760000
H	-0.744184000	-0.831232000	-1.358322000
H	0.124413000	-2.576641000	-2.921879000
H	1.610490000	2.975226000	-0.200295000
H	0.513856000	3.348247000	1.769475000
H	3.554033000	4.506008000	0.205790000
H	4.802943000	5.331717000	-0.730213000
H	3.166879000	5.236183000	-1.346216000
H	4.557568000	3.556010000	-2.569631000
H	5.113106000	1.389019000	-2.855650000
H	3.552201000	0.711871000	-2.469999000
H	4.755981000	0.486552000	1.863815000
H	5.962250000	1.155934000	0.759438000
H	3.999932000	2.690792000	0.993784000
H	2.531042000	0.838251000	1.623844000
H	2.062482000	0.199281000	-1.296452000
H	-0.058926000	-1.913195000	2.645920000
H	1.212170000	-3.424125000	4.108525000

H	3.572101000	-3.955809000	3.646151000
H	4.713274000	-2.931330000	1.694371000
H	3.683034000	-2.995146000	-1.210361000
H	5.647709000	-2.093399000	-2.054274000
H	2.663843000	-1.852144000	-2.068129000
H	4.683934000	-1.183568000	-3.212310000
H	5.514363000	-1.228096000	0.275803000

Equilibrium geometries of molecules from set 1 calculated at the **DFT(M06-2X)** level of theory with **6-311G(d,p)** basis set within the IEF-PCM model. Cartesian coordinates are given for standard orientation in form (atomic label, X, Y, Z). The X, Y, Z are given in Å.

12-28-oxaircinal (*E* = -1272.1382141 Hartree), solvent: Chloroform

O	1.581302	-0.804380	1.821438
O	-0.172109	4.723279	1.526055
N	1.768503	-0.502927	-0.465006
N	-2.189194	0.494091	-1.525731
C	0.067614	1.172777	-0.861504
C	0.390095	-0.138100	-0.108144
C	0.388171	-0.109872	1.449010
C	-0.575465	2.289495	0.000038
C	1.456308	1.618198	-1.386411
C	2.490348	0.769216	-0.632023
C	-0.864911	0.821017	-2.027261
C	2.179132	-1.357676	0.633813
C	-2.102276	2.179640	0.202990
C	0.087459	2.372177	1.346996
C	0.502002	1.282569	2.001316
C	-0.864485	-0.819891	1.994831
C	-2.827910	1.714245	-1.050873
C	3.669066	-1.503318	0.862270

C	3.799039	0.703527	-1.370587
C	-2.989452	-0.286043	-2.459218
C	-0.834157	-2.345760	1.805564
C	4.371477	-2.320596	-0.223102
C	0.215437	3.684858	2.006983
C	4.479589	-0.351574	-1.817430
C	4.123759	-1.808783	-1.654422
C	-4.302930	-0.785977	-1.843321
C	-2.192304	-2.956940	2.045862
C	-4.173978	-1.314622	-0.411492
C	-3.160628	-2.455720	-0.248480
C	-3.181212	-3.005590	1.152335
H	-0.292498	-0.924727	-0.434054
H	-0.399562	3.233298	-0.526690
H	1.636488	2.686318	-1.250703
H	1.536354	1.395194	-2.454924
H	2.679546	1.243014	0.346234
H	-0.900422	1.659232	-2.747625
H	-0.447199	-0.044543	-2.551168
H	1.734385	-2.347556	0.460138
H	-2.474075	3.155699	0.526691
H	-2.320874	1.471991	1.005200
H	0.948412	1.364713	2.990823
H	-1.728154	-0.424341	1.452558
H	-1.006264	-0.584835	3.054335
H	-3.873078	1.516881	-0.808945
H	-2.811782	2.497162	-1.830919
H	3.792807	-1.998671	1.828298
H	4.116527	-0.510891	0.955532
H	4.226700	1.689470	-1.546643

H	-2.380902	-1.147021	-2.753465
H	-3.213174	0.277377	-3.382288
H	-0.096718	-2.775668	2.485870
H	-0.502480	-2.589259	0.793662
H	4.040586	-3.362637	-0.164545
H	5.445451	-2.318706	-0.015647
H	0.701489	3.664270	3.001390
H	5.419101	-0.143867	-2.323774
H	3.077743	-1.967818	-1.914462
H	4.726559	-2.403633	-2.344073
H	-4.692263	-1.576503	-2.493231
H	-5.051811	0.011159	-1.859297
H	-2.376319	-3.362296	3.037557
H	-5.157856	-1.659501	-0.076485
H	-3.882668	-0.500205	0.260127
H	-2.167342	-2.085412	-0.512108
H	-3.409702	-3.255534	-0.957362
H	-4.124434	-3.455035	1.458766

Anabsinthin (Conformer 1, E = -1618.4548131 Hartree), solvent: Acetonitrile

O	-2.308383	1.214259	-2.209971
O	-2.556471	-1.947608	0.603602
O	2.462683	0.070832	3.091415
O	3.047738	-1.635697	-1.233127
O	-3.761462	-3.407803	1.776012
O	4.506745	-2.109733	-2.850454
C	-1.638874	0.178128	-0.239724
C	-1.967813	1.676234	0.045143
C	-0.486443	2.132250	-0.017785
C	-1.146264	0.540098	-1.679576

C	-0.528086	-0.132135	0.797411
C	0.147690	1.240251	1.063061
C	-2.789240	2.117417	-1.183344
C	-0.070790	1.610550	-1.414518
C	-2.804687	-0.813971	-0.267695
C	1.679945	1.032126	0.958118
C	0.607620	-1.032772	0.386686
C	-4.176556	-0.306035	0.173822
C	-4.308981	1.949869	-1.011759
C	1.780356	-0.389838	0.430704
C	-0.725280	-0.527969	-2.659617
C	-4.855555	0.529050	-0.899374
C	2.499264	1.242282	2.276206
C	-2.550422	3.572103	-1.594852
C	-4.875221	-1.619239	0.527917
C	3.094148	-1.018394	0.086304
C	0.380698	-2.477048	0.065185
C	3.988716	1.503007	1.993135
C	-3.721673	-2.449198	1.056211
C	4.288386	-0.074289	0.000282
C	4.801167	0.372490	1.360619
C	1.969721	2.442455	3.069577
C	-6.039024	-1.539270	1.501428
C	5.247188	-0.909594	-0.848463
C	4.282069	-1.624310	-1.777382
C	6.336002	-0.160558	-1.597841
H	-2.445681	1.889177	1.002667
H	-0.310940	3.196633	0.141775
H	-1.013725	-0.535469	1.690895
H	-0.151508	1.621361	2.038156

H	0.931931	1.184317	-1.431700
H	-0.122364	2.365679	-2.198851
H	-2.910472	-1.224138	-1.277024
H	2.089200	1.755889	0.241955
H	-4.063371	0.276004	1.098234
H	-4.797093	2.437003	-1.861422
H	-4.594840	2.520587	-0.121361
H	-1.487723	-1.297719	-2.793965
H	-0.549116	-0.057768	-3.630140
H	0.205216	-0.994753	-2.332195
H	-4.753867	0.013240	-1.859655
H	-5.926536	0.593093	-0.685580
H	-3.041797	3.758867	-2.551956
H	-2.987390	4.242835	-0.850234
H	-1.496506	3.819651	-1.698323
H	-5.196079	-2.107643	-0.403518
H	3.324026	-1.821505	0.798575
H	-0.321735	-2.588418	-0.765672
H	1.306009	-2.991089	-0.190710
H	-0.087419	-2.968672	0.922351
H	4.455414	1.752104	2.950326
H	4.057125	2.398761	1.366348
H	3.996229	0.801771	-0.594859
H	4.828793	-0.488629	2.036058
H	5.831449	0.724949	1.253798
H	1.016953	2.215287	3.548687
H	1.830468	3.312240	2.421164
H	2.679194	2.703776	3.856433
H	-5.726885	-1.059889	2.432078
H	-6.407522	-2.538317	1.737245

H	-6.859375	-0.961631	1.072560
H	1.543519	-0.198071	3.194231
H	5.692387	-1.687365	-0.211594
H	6.896108	-0.843657	-2.237484
H	7.032652	0.306221	-0.899763
H	5.900110	0.619129	-2.226609

Anabsinthin (Conformer 2, *E* = -1618.4553793 Hartree), solvent: Acetonitrile

O	-2.181375	2.410533	-0.817806
O	-2.590783	-1.799370	-0.869575
O	1.510282	-1.987407	2.845175
O	3.036022	-0.254783	-2.032582
O	-3.860393	-3.624879	-0.989737
O	4.464663	0.733309	-3.430724
C	-1.593037	0.298420	-0.052146
C	-1.877399	1.230899	1.163596
C	-0.380174	1.553727	1.414153
C	-1.052143	1.510821	-0.881656
C	-0.522940	-0.675820	0.507149
C	0.193923	0.144555	1.618747
C	-2.657489	2.411539	0.551766
C	0.049940	2.087302	0.027476
C	-2.789380	-0.368195	-0.735826
C	1.706698	-0.034702	1.383343
C	0.584083	-1.103166	-0.430105
C	-4.151196	-0.245935	-0.054145
C	-4.185395	2.230070	0.575886
C	1.774292	-0.681460	0.013801
C	-0.644155	1.361847	-2.327439
C	-4.782473	1.118388	-0.283586

C	2.385198	-0.918535	2.471369
C	-2.362809	3.756431	1.220208
C	-4.896484	-1.428918	-0.673049
C	3.089254	-0.828044	-0.696320
C	0.325591	-1.913130	-1.661481
C	3.697875	-1.570138	1.990635
C	-3.778288	-2.434893	-0.864859
C	4.231195	-0.062102	-0.029254
C	4.739652	-0.703337	1.267612
C	2.602221	-0.100846	3.735439
C	-6.067057	-1.993313	0.114358
C	5.210251	0.068587	-1.193259
C	4.251851	0.236725	-2.361329
C	6.238651	1.183206	-1.106948
H	-2.367122	0.765780	2.020671
H	-0.175203	2.228813	2.245637
H	-1.041891	-1.552089	0.906337
H	-0.088793	-0.220786	2.605946
H	1.040383	1.745023	-0.272321
H	0.038406	3.174203	-0.049848
H	-2.891728	0.021576	-1.753546
H	2.212905	0.936587	1.400862
H	-4.035245	-0.440372	1.020152
H	-4.639366	3.177702	0.270038
H	-4.470674	2.073017	1.621879
H	-0.461051	2.357423	-2.738783
H	0.279925	0.786078	-2.401544
H	-1.417007	0.885717	-2.933787
H	-4.682500	1.373359	-1.343658
H	-5.854066	1.063044	-0.070048

H	-2.789769	3.768793	2.226588
H	-1.299165	3.969019	1.299882
H	-2.830896	4.555598	0.641736
H	-5.223285	-1.145507	-1.683832
H	3.360676	-1.882994	-0.826804
H	-0.142911	-2.861179	-1.384087
H	-0.380445	-1.410517	-2.327527
H	1.245357	-2.109552	-2.212612
H	3.420129	-2.399436	1.330800
H	4.165314	-2.031045	2.865087
H	3.856359	0.946369	0.176909
H	5.589396	-1.351919	1.031527
H	5.122362	0.074140	1.933841
H	2.936853	-0.752536	4.544910
H	1.674307	0.387765	4.042338
H	3.358567	0.669382	3.568611
H	-6.478469	-2.869162	-0.388728
H	-6.858351	-1.248543	0.213206
H	-5.746056	-2.291933	1.114893
H	1.303134	-2.484905	2.044745
H	5.711391	-0.897587	-1.345887
H	6.926905	1.003052	-0.279639
H	5.747240	2.145505	-0.946040
H	6.815508	1.242987	-2.030721

Betulinic Acid (*E* = -1397.5744259 Hartree), solvent: Pyridine

C	4.187565	1.210538	0.362231
C	3.160217	0.295206	-0.343305
C	3.799515	-1.103624	-0.368891
C	5.256376	-0.758925	-0.735224

C	5.557028	0.486449	0.122444
C	1.718132	0.256151	0.160732
C	0.842275	-0.706471	-0.701637
C	1.550207	-2.078097	-0.883942
C	3.017193	-1.987078	-1.338643
C	1.071012	1.638496	0.252615
C	-0.366224	1.577229	0.788721
C	-1.250773	0.530592	0.087276
C	-0.553848	-0.867416	0.046283
C	-2.745129	0.540176	0.571806
C	-3.510091	-0.450339	-0.358623
C	-2.914374	-1.857787	-0.260818
C	-1.451077	-1.868139	-0.708784
C	-3.312215	1.957322	0.323062
C	-4.834877	2.029232	0.411090
C	-5.501895	1.047753	-0.533539
C	-5.065233	-0.415755	-0.290382
C	4.230488	2.638326	-0.138082
C	4.249901	3.665047	0.710954
C	4.308347	2.851313	-1.628468
C	3.855501	-1.749167	1.010234
O	3.745209	-1.193702	2.074327
O	4.119464	-3.066100	0.936315
C	0.667363	-0.136338	-2.129168
C	-0.343640	-1.423234	1.474824
C	-2.895756	0.227274	2.077745
C	-5.636523	-1.266677	-1.438406
C	-5.658052	-0.953738	1.018476
O	-6.906237	1.234634	-0.386245
H	3.977266	1.229483	1.432922

H	3.151161	0.597983	-1.396279
H	5.280779	-0.512080	-1.801166
H	5.953850	-1.581073	-0.563445
H	6.286013	1.141876	-0.357358
H	5.978138	0.190330	1.084947
H	1.761895	-0.150797	1.174645
H	0.991380	-2.671041	-1.613111
H	1.537681	-2.651879	0.046229
H	3.097683	-1.549934	-2.337613
H	3.445843	-2.989346	-1.391820
H	1.089160	2.124098	-0.729827
H	1.668056	2.273703	0.912011
H	-0.814211	2.567255	0.681097
H	-0.320850	1.381155	1.863741
H	-1.338992	0.850264	-0.956057
H	-3.279737	-0.098345	-1.377858
H	-3.464512	-2.547965	-0.903499
H	-3.013812	-2.249924	0.755055
H	-1.439451	-1.644284	-1.779346
H	-1.042086	-2.877102	-0.591730
H	-3.001966	2.294173	-0.674579
H	-2.885136	2.661049	1.042179
H	-5.173455	3.036351	0.153870
H	-5.187696	1.836653	1.428766
H	-5.205692	1.307327	-1.562329
H	4.332247	4.688743	0.361300
H	4.179617	3.508675	1.782578
H	3.339418	2.661674	-2.100688
H	4.602853	3.874310	-1.863938
H	5.027051	2.166392	-2.090313

H	4.192912	-3.398091	1.842449
H	0.159373	0.824571	-2.163750
H	1.634726	0.008806	-2.611407
H	0.109970	-0.829063	-2.761536
H	-1.264701	-1.858552	1.857888
H	0.405185	-2.215226	1.493719
H	-0.028262	-0.666654	2.192075
H	-2.003829	0.522614	2.630765
H	-3.727988	0.781744	2.512556
H	-3.074129	-0.827741	2.284126
H	-5.165140	-1.014167	-2.393079
H	-5.498936	-2.333862	-1.259017
H	-6.714654	-1.111536	-1.536187
H	-6.744450	-1.013050	0.927447
H	-5.281561	-1.959534	1.222507
H	-5.434885	-0.327439	1.880330
H	-7.352086	0.780881	-1.105929

Icajine (Conformer 1, *E* = -1187.7964741 Hartree), solvent: Chloroform

O	-0.131455	-2.870891	1.217254
O	2.083148	3.026801	0.703913
O	-2.550725	3.195672	-0.414761
N	-1.448084	1.200145	-0.496888
N	2.090347	-2.081394	-0.550351
C	-0.149144	0.523118	-0.317207
C	0.217175	-1.737461	0.963214
C	1.376318	-1.734980	-1.773296
C	-0.133082	-1.749690	-1.508154
C	-0.481943	-0.991728	-0.205209
C	-1.991673	-0.976893	-0.018837

C	-2.849177	-2.028746	0.259487
C	-4.222712	-1.786032	0.308791
C	-4.719747	-0.508151	0.072564
C	-3.869824	0.559907	-0.213288
C	-2.503816	0.302309	-0.249989
C	1.207739	-1.021212	1.869311
C	1.747901	0.303908	1.316102
C	0.507241	1.115098	0.926764
C	0.722629	2.639295	0.777380
C	2.838272	2.655342	-0.429133
C	3.164226	1.196714	-0.570361
C	2.737615	0.163054	0.154781
C	3.217437	-1.227164	-0.200384
C	-0.137109	3.214588	-0.368713
C	-1.510023	2.567839	-0.425914
C	2.338226	-3.501751	-0.402916
H	0.470642	0.714543	-1.194830
H	1.623908	-2.426484	-2.589363
H	1.693192	-0.741444	-2.096668
H	-0.491954	-2.776245	-1.392774
H	-0.676893	-1.304517	-2.346992
H	-2.445953	-3.016195	0.443657
H	-4.904363	-2.598541	0.528619
H	-5.788335	-0.332369	0.109928
H	-4.247217	1.554713	-0.394376
H	1.989210	-1.742567	2.114789
H	0.678835	-0.808857	2.805046
H	2.262647	0.824855	2.128512
H	-0.227486	0.989619	1.732482
H	0.392111	3.109205	1.704777

H	3.775746	3.210970	-0.338158
H	2.371078	3.010023	-1.358295
H	3.853553	0.995976	-1.389207
H	3.707560	-1.680836	0.667767
H	3.970558	-1.171192	-1.000602
H	-0.268051	4.289065	-0.259167
H	0.345705	3.024850	-1.331584
H	3.088971	-3.874433	-1.115186
H	1.404341	-4.047389	-0.552944
H	2.679437	-3.710842	0.614039

Icajine (Conformer 2, *E* = -1187.7978288 Hartree), solvent: Chloroform

O	-0.021195	-2.784882	1.311900
O	1.570530	2.928966	-0.581407
O	-2.883937	3.001790	-0.263457
N	-1.542089	1.175152	-0.356713
N	2.198384	-2.007466	-0.482787
C	-0.188490	0.580417	-0.274831
C	0.291455	-1.651333	1.018013
C	1.503668	-1.633148	-1.707463
C	-0.003464	-1.696655	-1.469174
C	-0.420572	-0.959084	-0.171671
C	-1.930144	-1.066200	-0.049272
C	-2.706893	-2.198262	0.136147
C	-4.096416	-2.074347	0.123964
C	-4.686106	-0.832317	-0.086710
C	-3.916587	0.314224	-0.280627
C	-2.532075	0.176422	-0.249180
C	1.258110	-0.872724	1.899767
C	1.798436	0.428931	1.288128

C	0.551038	1.234057	0.886233
C	0.746481	2.715947	0.549789
C	2.952323	2.700421	-0.349685
C	3.351725	1.258153	-0.506767
C	2.840477	0.238553	0.183713
C	3.320433	-1.162826	-0.111612
C	-0.599065	3.381495	0.249220
C	-1.774129	2.509557	-0.168541
C	2.443147	-3.429283	-0.353407
H	0.351900	0.815049	-1.195748
H	1.795325	-0.617543	-1.982725
H	1.785049	-2.284315	-2.546138
H	-0.328869	-2.736004	-1.367112
H	-0.547910	-1.267852	-2.315886
H	-2.230271	-3.156186	0.299739
H	-4.716152	-2.950194	0.272365
H	-5.766242	-0.746340	-0.103756
H	-4.369238	1.279080	-0.444716
H	0.707599	-0.617016	2.812235
H	2.042682	-1.569067	2.200297
H	2.299947	0.983720	2.092064
H	-0.118734	1.213968	1.757101
H	1.206904	3.216038	1.412659
H	3.219953	3.070533	0.651179
H	3.481974	3.311551	-1.080010
H	4.103474	1.043485	-1.261421
H	3.770606	-1.596063	0.788502
H	4.106503	-1.132867	-0.881611
H	-0.967507	3.903140	1.135420
H	-0.450928	4.142543	-0.519157

H	3.203085	-3.792278	-1.061340
H	1.512264	-3.974002	-0.522864
H	2.771228	-3.652154	0.664929

Iguesterin (*E* = -1238.8814329 Hartree), solvent: Chloroform

O	-6.529184	0.814632	-0.981052
O	-5.271269	2.643877	0.412472
C	1.665188	0.213889	0.435546
C	1.053030	-1.230067	0.557102
C	3.210406	0.148076	0.641706
C	3.975378	-0.896573	-0.229319
C	1.735753	-2.155058	-0.471300
C	1.016861	1.074121	1.538267
C	3.247572	-2.249840	-0.261129
C	-0.454191	-1.123101	0.309318
C	-1.278412	-0.079237	1.063530
C	-0.494618	1.226211	1.353627
C	3.911968	1.517336	0.536722
C	1.299889	0.852859	-0.919627
C	4.201229	-0.431279	-1.683964
C	1.282597	-1.874211	1.948698
C	5.361261	-1.117870	0.399302
C	4.385420	1.912003	-0.839951
C	-2.610012	0.244552	0.386759
C	4.516800	1.027444	-1.825690
C	-1.096302	-1.964196	-0.525667
C	-1.727652	-0.722088	2.418682
C	-3.249231	-0.772763	-0.441414
C	-2.496958	-1.836189	-0.831580
C	4.718406	3.367738	-1.010505

C	-3.275458	1.390788	0.668177
C	-4.622829	-0.601663	-0.899230
C	-4.625620	1.619092	0.192913
C	-5.263819	0.545652	-0.592106
C	-5.297247	-1.664176	-1.720551
H	3.343521	-0.177554	1.680504
H	1.322403	-3.164183	-0.387963
H	1.512631	-1.808516	-1.484837
H	1.256618	0.671632	2.524777
H	1.430877	2.083977	1.520395
H	3.681987	-2.853085	-1.066567
H	3.459672	-2.798816	0.660080
H	-0.671600	1.922592	0.531568
H	-0.915418	1.696207	2.246399
H	3.268523	2.313758	0.918911
H	4.784457	1.525577	1.202806
H	0.226147	0.806172	-1.113407
H	1.589916	1.907438	-0.921616
H	1.796047	0.379657	-1.761915
H	5.033405	-1.019452	-2.091435
H	3.345336	-0.690057	-2.317746
H	2.336360	-2.080736	2.125487
H	0.942750	-1.264823	2.778885
H	0.745186	-2.825230	1.990217
H	5.274171	-1.361197	1.462421
H	5.995362	-0.234122	0.295819
H	5.866669	-1.950864	-0.098089
H	4.869602	1.365611	-2.797468
H	-0.565538	-2.758795	-1.031208
H	-0.886384	-0.863371	3.091837

H	-2.209755	-1.686624	2.247519
H	-2.442124	-0.051262	2.898963
H	-2.938417	-2.598447	-1.464213
H	5.445579	3.689589	-0.257765
H	3.823973	3.984182	-0.869980
H	5.129539	3.573555	-1.999662
H	-2.852942	2.180740	1.275747
H	-5.275816	-2.629579	-1.209041
H	-4.802675	-1.788983	-2.687813
H	-6.335093	-1.392827	-1.902394
H	-6.717783	1.701060	-0.630462

Itoaic Acid (Conformer 1, *E* = -1623.2510555 Hartree), solvent:

C	-3.421339	-1.108362	-0.275518
C	-2.565129	0.161235	0.058522
C	-1.261309	-0.115350	0.904543
C	-0.422157	-1.140834	0.059368
C	-1.218120	-2.433226	-0.132602
C	-2.500160	-2.163850	-0.912995
C	-0.435941	1.202554	1.013693
C	1.054959	1.001851	1.318111
C	1.781751	0.041608	0.358401
C	1.065582	-1.352845	0.476649
C	3.302218	-0.071699	0.755379
C	4.098139	-1.288608	0.176561
C	3.243786	-2.560401	-0.058202
C	1.774733	-2.367946	-0.444064
C	4.134253	1.247730	0.662845
C	5.088181	1.528060	-0.534842
C	5.777600	0.240066	-1.005306

C	4.803593	-0.924206	-1.146073
C	-3.389703	1.339147	0.602585
C	-4.453628	1.922262	-0.340400
C	-5.719239	1.084335	-0.301256
O	-5.675567	-0.166394	-0.741532
C	-4.487412	-0.736862	-1.347965
O	-4.774092	3.233028	0.054651
O	-6.740637	1.521837	0.167410
C	-5.043283	-1.893786	-2.166216
C	-4.150737	-1.721536	0.930472
C	-1.605640	-0.583448	2.336851
O	-0.948391	2.122035	1.973966
C	1.163472	-1.932370	1.903906
C	5.168286	-1.667283	1.219163
C	6.152520	2.516277	-0.037285
C	4.404006	2.182476	-1.744895
C	1.686356	0.619827	-1.057606
O	1.530965	1.956640	-1.051302
O	1.771576	0.027999	-2.104787
H	-2.180830	0.498651	-0.916610
H	-0.365983	-0.700201	-0.945933
H	-1.439922	-2.916251	0.822876
H	-0.636936	-3.152008	-0.709946
H	-3.052354	-3.098837	-1.038463
H	-2.214000	-1.825284	-1.917311
H	-0.526905	1.729845	0.060707
H	1.167749	0.622790	2.338106
H	1.502363	1.994464	1.308393
H	3.227792	-0.256032	1.832100
H	3.280125	-3.181561	0.839905

H	3.734420	-3.154550	-0.835624
H	1.296936	-3.344351	-0.338496
H	1.675184	-2.066865	-1.484977
H	4.767269	1.253715	1.555051
H	3.474178	2.112176	0.774994
H	6.575551	-0.030699	-0.306779
H	6.269264	0.434146	-1.964983
H	5.352043	-1.809456	-1.484714
H	4.054168	-0.711430	-1.913876
H	-2.728445	2.177088	0.805103
H	-3.870639	1.105377	1.557709
H	-4.065367	1.956496	-1.364538
H	-4.061881	-0.005208	-2.041702
H	-5.676891	3.200320	0.398952
H	-4.286866	-2.310205	-2.829034
H	-5.429725	-2.687802	-1.524955
H	-5.865599	-1.519599	-2.776257
H	-4.666482	-0.973513	1.534396
H	-4.907363	-2.429746	0.585135
H	-3.468347	-2.268227	1.578340
H	-2.571873	-0.193999	2.657384
H	-0.875143	-0.230833	3.067704
H	-1.635250	-1.666329	2.438625
H	-0.767906	1.779195	2.853838
H	0.643742	-2.894681	1.924534
H	0.723171	-1.309710	2.673386
H	2.193808	-2.122681	2.198768
H	5.803186	-0.826281	1.502630
H	4.693457	-2.042396	2.130140
H	5.814707	-2.458870	0.826885

H	6.866548	2.742860	-0.834347
H	5.693713	3.456554	0.284186
H	6.707842	2.100727	0.808216
H	5.173157	2.512633	-2.448948
H	3.754353	1.490980	-2.282875
H	3.823354	3.057318	-1.443308
H	1.561978	2.252218	-1.972581

Itoaic Acid (Conformer 2, *E* = -1623.2505042 Hartree), solvent:

C	-3.493245	-1.103727	0.145716
C	-2.637627	0.205458	0.014587
C	-1.242549	0.170095	0.756239
C	-0.490239	-1.065914	0.150009
C	-1.267478	-2.347655	0.454283
C	-2.621049	-2.314249	-0.244513
C	-0.421075	1.443384	0.393482
C	1.082745	1.314606	0.661090
C	1.742999	0.128093	-0.062257
C	1.041431	-1.185767	0.413103
C	3.281499	0.062559	0.265267
C	3.984407	-1.280429	-0.187405
C	3.028576	-2.192925	-0.972579
C	1.629338	-2.403151	-0.364986
C	4.055518	1.303743	-0.217629
C	5.562189	1.287498	0.098909
C	6.196417	-0.037650	-0.416669
C	5.175117	-0.941423	-1.105932
C	-3.432304	1.490013	0.295214
C	-4.604722	1.790183	-0.651740
C	-5.836546	0.996980	-0.252534

O	-5.806884	-0.326144	-0.340617
C	-4.667927	-1.048313	-0.876259
O	-4.921016	3.159384	-0.599345
O	-6.815927	1.548096	0.184825
C	-5.271933	-2.388218	-1.271994
C	-4.088772	-1.332156	1.544976
C	-1.416095	0.146810	2.291507
O	-0.868203	2.627603	1.045879
C	1.289692	-1.438278	1.915125
C	4.538370	-2.082566	1.012060
C	5.803206	1.458291	1.603313
C	6.202238	2.477538	-0.625583
C	1.501888	0.372828	-1.566460
O	1.708231	1.657977	-1.914640
O	1.132879	-0.421920	-2.392430
H	-2.368914	0.252932	-1.051935
H	-0.571037	-0.937457	-0.934504
H	-1.397838	-2.498404	1.528643
H	-0.717240	-3.219168	0.093668
H	-3.169754	-3.235436	-0.031117
H	-2.441522	-2.291598	-1.327045
H	-0.570830	1.648641	-0.673394
H	1.259588	1.218856	1.736091
H	1.528516	2.258719	0.353468
H	3.307058	0.093474	1.358908
H	3.514096	-3.168653	-1.084499
H	2.917247	-1.797914	-1.981880
H	1.647049	-3.262010	0.312706
H	0.961967	-2.669917	-1.186524
H	3.623439	2.204698	0.227477

H	3.943739	1.420742	-1.296736
H	6.652244	-0.581092	0.417107
H	7.010795	0.186932	-1.111423
H	5.650555	-1.876321	-1.420060
H	4.808701	-0.461268	-2.019970
H	-2.775106	2.347350	0.182325
H	-3.804509	1.533650	1.323595
H	-4.324651	1.540684	-1.681454
H	-4.330653	-0.545009	-1.787827
H	-5.781052	3.222331	-0.162207
H	-4.574637	-2.978538	-1.863435
H	-5.570918	-2.965006	-0.395159
H	-6.160589	-2.199190	-1.874414
H	-4.546123	-0.432386	1.959091
H	-4.869933	-2.094262	1.496148
H	-3.338355	-1.683405	2.250602
H	-2.330316	0.660710	2.590579
H	-0.593882	0.651168	2.804191
H	-1.453796	-0.859771	2.701767
H	-0.657985	2.557698	1.981711
H	0.733095	-2.328014	2.219264
H	0.974317	-0.622318	2.558715
H	2.335963	-1.633635	2.128845
H	4.945903	-1.445044	1.799127
H	3.778964	-2.724789	1.459229
H	5.342735	-2.741255	0.671191
H	5.344560	0.658020	2.188388
H	6.875644	1.452728	1.818520
H	5.392536	2.409839	1.954917
H	6.081925	2.380981	-1.708670

H	5.742470	3.420231	-0.313600
H	7.272716	2.531010	-0.406976
H	1.526759	1.726570	-2.862875

Matopensine (*E* = -1767.2446725 Hartree), solvent: Chloroform

O	-0.000020	-0.000007	2.600943
N	-1.354389	-0.866229	0.951971
N	-3.775696	2.234157	-1.064070
C	-2.073176	0.388688	1.237757
C	-3.334414	0.291767	0.342774
C	-1.099386	1.560447	1.042077
C	-0.218787	-1.155698	1.806731
C	-3.092937	0.930806	-1.067849
C	-0.921862	1.981260	-0.452809
C	-1.617645	1.005561	-1.422297
C	-4.562287	1.062319	0.875382
C	-3.500059	-1.211742	0.258350
C	-2.301944	-1.832342	0.640626
C	-5.004048	1.936341	-0.319758
C	-1.574501	3.317400	-0.739119
C	-3.043122	3.337318	-0.404156
C	-4.580418	-1.975387	-0.135100
C	-2.177575	-3.215720	0.665182
C	-1.003764	4.355362	-1.353985
C	-4.470767	-3.372693	-0.123963
C	-3.285377	-3.976189	0.276433
C	0.401402	4.481741	-1.864660
H	-2.404587	0.379413	2.285903
H	-1.501974	2.412630	1.594911
H	-0.479127	-1.943323	2.522660

H	-3.620437	0.311277	-1.801242
H	0.137634	2.018200	-0.694513
H	-1.498175	1.383049	-2.440848
H	-1.182926	0.005455	-1.372527
H	-4.277570	1.684680	1.728096
H	-5.350935	0.390661	1.216848
H	-5.677555	1.370897	-0.971241
H	-5.511615	2.856170	-0.024314
H	-3.490696	4.277206	-0.735427
H	-3.167149	3.294943	0.689797
H	-5.509729	-1.500604	-0.435395
H	-1.247764	-3.695296	0.955586
H	-1.638510	5.218504	-1.548854
H	-5.314657	-3.983047	-0.420905
H	-3.211918	-5.057677	0.287491
H	0.380917	4.771641	-2.919045
H	0.945465	5.267757	-1.331834
H	0.976509	3.561573	-1.776645
N	1.354375	0.866223	0.951995
N	3.775710	-2.234152	-1.064029
C	2.073158	-0.388695	1.237785
C	3.334410	-0.291770	0.342821
C	1.099371	-1.560452	1.042085
C	0.218761	1.155688	1.806740
C	3.092952	-0.930800	-1.067809
C	0.921869	-1.981257	-0.452805
C	1.617665	-1.005553	-1.422279
C	4.562274	-1.062326	0.875442
C	3.500056	1.211739	0.258411
C	2.301937	1.832338	0.640674

C	5.004052	-1.936340	-0.319697
C	1.574510	-3.317395	-0.739114
C	3.043128	-3.337317	-0.404132
C	4.580423	1.975387	-0.135015
C	2.177570	3.215715	0.665242
C	1.003779	-4.355353	-1.353993
C	4.470774	3.372693	-0.123866
C	3.285379	3.976187	0.276517
C	-0.401384	-4.481727	-1.864678
H	2.404554	-0.379425	2.285936
H	1.501950	-2.412639	1.594920
H	0.479091	1.943310	2.522678
H	3.620462	-0.311267	-1.801191
H	-0.137624	-2.018195	-0.694524
H	1.498210	-1.383037	-2.440833
H	1.182946	-0.005447	-1.372511
H	4.277545	-1.684693	1.728147
H	5.350917	-0.390670	1.216924
H	5.677569	-1.370893	-0.971166
H	5.511614	-2.856171	-0.024251
H	3.490705	-4.277202	-0.735402
H	3.167140	-3.294948	0.689823
H	5.509738	1.500605	-0.435299
H	1.247756	3.695291	0.955637
H	1.638526	-5.218494	-1.548862
H	5.314670	3.983049	-0.420790
H	3.211921	5.057675	0.287584
H	-0.380892	-4.771631	-2.919062
H	-0.945456	-5.267737	-1.331853
H	-0.976487	-3.561554	-1.776672

Naucleidinal (*E* = -1109.2106341 Hartree), solvent: Chloroform

O	-3.471126	1.448317	1.311899
O	-1.229235	-2.001351	1.052350
O	-6.769011	-0.149664	-1.598499
N	-2.991368	-3.225474	0.320118
N	-5.081961	-2.937580	-2.695446
C	-4.334057	-3.299869	-0.268024
C	-4.559261	-0.841208	-0.091957
C	-5.233059	-2.172590	0.212652
C	-5.396989	0.367247	0.301095
C	-4.143678	-3.324822	-1.761641
C	-3.247466	-0.822035	0.643469
C	-2.121140	-4.317591	-0.120917
C	-2.991212	-3.723320	-2.375857
C	-2.398301	-2.042142	0.693669
C	-1.773738	-4.164263	-1.613972
C	-4.502193	1.620229	0.328089
C	-3.205685	-3.560883	-3.788688
C	-2.830705	0.264961	1.302275
C	-4.518424	-3.051983	-3.948864
C	-6.578432	0.540013	-0.628885
C	-5.215934	2.907443	0.681909
C	-2.417824	-3.784410	-4.927031
C	-5.050338	-2.752998	-5.204279
C	-2.943417	-3.491253	-6.173242
C	-4.246107	-2.977052	-6.309014
H	-4.767515	-4.252375	0.057605
H	-4.381535	-0.787247	-1.172435
H	-6.208106	-2.253747	-0.271623

H	-5.385923	-2.272052	1.291308
H	-5.802637	0.248992	1.316872
H	-2.662557	-5.252549	0.043621
H	-1.226220	-4.315703	0.495577
H	-1.389818	-5.115660	-1.993408
H	-0.971737	-3.427035	-1.729858
H	-4.004537	1.708382	-0.645693
H	-5.918610	-2.409486	-2.488703
H	-1.928476	0.267957	1.901278
H	-7.293740	1.341027	-0.370160
H	-5.887166	3.216608	-0.120412
H	-5.791612	2.787053	1.602118
H	-4.478897	3.696490	0.832136
H	-1.412143	-4.178514	-4.829223
H	-6.054378	-2.358444	-5.307877
H	-2.345678	-3.657977	-7.061425
H	-4.628253	-2.753941	-7.298009

Physalin D (*E* = -1913.5311175 Hartree), solvent: Dimethylsulfoxide

C	-6.151680	0.494472	-0.616384
C	-5.538840	-0.857303	-0.401620
C	-4.016434	-0.821516	-0.546886
C	-3.390638	0.350292	0.271429
C	-3.966740	1.614599	-0.382347
C	-5.436707	1.623098	-0.606638
C	-3.397353	-2.172138	-0.196081
C	-1.884297	-2.134486	-0.339311
C	-1.246469	-1.028138	0.514105
C	-1.842231	0.351232	0.141520
O	-3.292573	2.565517	-0.718756

C	0.300604	-1.067377	0.386739
C	1.118685	-0.788466	1.669377
C	2.369512	-0.047464	1.224463
C	1.774367	0.769481	0.070589
C	1.215372	2.124311	0.596516
C	-0.223897	2.404517	0.177454
C	-1.224397	1.518695	0.945080
O	0.816458	-1.138857	2.773741
O	0.779892	-0.076771	-0.515736
O	0.657816	-2.336427	-0.091478
C	3.535533	-0.984432	0.831812
C	3.144317	-2.135254	-0.125728
C	1.900246	-2.934884	0.262352
C	4.573541	-0.112745	0.122404
C	4.013313	0.369032	-1.198279
C	2.739060	1.218109	-1.066327
C	2.233025	3.139021	0.068935
O	3.183320	2.532169	-0.660382
O	2.256491	4.304555	0.332225
C	3.099135	-1.827482	-1.616983
O	3.731012	-0.731897	-2.074904
C	2.043748	1.368871	-2.414115
O	2.617361	-2.586652	-2.410767
C	4.126217	-1.566989	2.118710
O	-3.673358	-0.648350	-1.926003
O	-3.794457	-2.519448	1.123029
C	-3.849064	0.346502	1.748555
O	1.322425	2.198597	2.011810
H	-7.219078	0.535687	-0.810033
H	-5.937832	-1.563664	-1.136788

H	-5.818544	-1.245743	0.581551
H	-5.882051	2.589415	-0.814980
H	-3.806404	-2.895660	-0.912531
H	-1.634408	-1.981668	-1.392302
H	-1.489108	-3.109014	-0.052971
H	-1.457545	-1.244761	1.568118
H	-1.628208	0.510897	-0.918156
H	2.716064	0.587621	2.035223
H	-0.353301	2.282319	-0.898231
H	-0.410733	3.459213	0.401664
H	-2.014715	2.179297	1.287674
H	-0.757236	1.138900	1.858624
H	3.975971	-2.851199	-0.066835
H	1.920404	-3.164483	1.331836
H	1.902978	-3.873919	-0.285312
H	4.838381	0.748247	0.742246
H	5.486799	-0.683943	-0.065681
H	4.743248	0.970462	-1.740636
H	2.775899	1.721018	-3.143115
H	1.243364	2.106833	-2.347296
H	1.624934	0.425466	-2.753679
H	4.477906	-0.759730	2.764800
H	3.389696	-2.145744	2.680208
H	4.973736	-2.215998	1.887595
H	-4.295944	-0.032848	-2.327476
H	-3.448166	-3.396466	1.312790
H	-3.519084	1.253000	2.256118
H	-4.935057	0.308757	1.830162
H	-3.445790	-0.516595	2.273312
H	1.041159	3.083474	2.275834

Strychnobaillonine (Conformer 1, *E* = -1919.9126963 Hartree), solvent: Chloroform

O	1.199103	2.854030	1.695165
O	-1.237825	3.458152	0.780143
N	-2.376025	1.615580	0.097866
N	-4.194802	-2.418946	-1.213251
N	1.063095	-0.656183	0.360593
N	5.104907	-0.141571	-1.515161
C	-5.455119	3.196146	1.509999
C	-6.369271	2.252272	1.049864
C	-5.930791	1.165179	0.294615
C	-4.580201	1.040439	0.011206
C	-3.677932	1.990919	0.479732
C	-4.093202	3.085183	1.233000
C	-3.891000	-0.047197	-0.776938
C	-2.396923	0.431005	-0.799242
C	-1.240225	2.330746	0.302980
C	0.020119	1.561478	-0.056900
C	-0.177667	0.109955	0.442514
C	-1.249854	-0.569873	-0.440859
C	-4.518728	-0.247160	-2.186783
C	-5.047259	-1.702202	-2.166573
C	-4.091562	-1.450292	-0.115471
C	-3.019671	-1.784695	0.903516
C	-1.677700	-1.923361	0.182456
C	0.159428	-4.456185	-0.144191
C	-1.031513	-4.079441	-0.976059
C	-1.809270	-3.001566	-0.864576
C	-2.903504	-2.724273	-1.855976
C	1.306144	2.279667	0.406114

C	2.479640	1.302425	0.491774
C	2.213763	0.007165	-0.279391
C	1.581647	-1.411622	1.414546
C	3.365982	-1.017569	-0.113894
C	2.937845	-1.694671	1.170039
C	0.960478	-1.876831	2.569488
C	1.704511	-2.669819	3.448886
C	3.032260	-2.985113	3.193235
C	3.656800	-2.485935	2.043256
C	3.858251	1.919336	0.205940
C	4.901559	0.918195	0.707699
C	4.770117	-0.354840	-0.104055
C	4.715889	-1.416712	-2.133090
C	3.483471	-1.959525	-1.339810
C	4.396867	0.990967	-2.149949
C	4.138663	2.195516	-1.255818
C	4.165054	3.418403	-1.792673
C	3.845856	4.726005	-1.126787
H	-3.383977	3.819026	1.582779
H	-2.979319	-0.989374	1.657821
H	-5.070936	-1.435338	0.374035
H	-6.077804	-1.728115	-1.802027
H	-5.313454	0.474981	-2.374794
H	-3.049169	-3.579569	-2.518966
H	-6.638960	0.429004	-0.073158
H	-7.422454	2.364316	1.275892
H	-5.803011	4.039629	2.094426
H	0.067332	1.518818	-1.154294
H	-0.511123	0.144008	1.487251
H	1.054176	-4.505889	-0.772676

H	0.363552	-3.755817	0.664150
H	-2.192757	0.806394	-1.806870
H	-0.912768	-2.221121	0.894626
H	-2.570660	-1.881341	-2.489944
H	-1.265563	-4.772349	-1.783161
H	-3.768907	-0.111549	-2.968346
H	-5.021996	-2.182709	-3.145965
H	-3.267979	-2.718140	1.412765
H	-0.727918	-0.788813	-1.377591
H	0.023473	-5.450534	0.290277
H	1.519047	3.061679	-0.336884
H	0.454850	3.469196	1.658008
H	3.085199	5.262722	-1.700729
H	3.472330	4.611249	-0.109751
H	4.729676	5.370179	-1.097362
H	4.439940	3.495927	-2.843910
H	4.989681	1.313729	-3.009645
H	3.432698	0.655331	-2.560594
H	4.738882	0.703099	1.767412
H	5.913061	1.313961	0.588471
H	3.932974	2.843397	0.780612
H	2.499171	1.007875	1.548995
H	1.983578	0.191472	-1.329374
H	-0.071640	-1.638923	2.798883
H	1.229308	-3.039457	4.350112
H	3.588075	-3.602764	3.887443
H	4.699996	-2.713218	1.847694
H	3.660368	-2.986631	-1.015795
H	5.548885	-2.117846	-2.041036
H	2.562761	-1.956182	-1.927747

H	4.515518	-1.278278	-3.196430
H	5.477715	-1.104345	0.271672

Strychnobaillonine (Conformer 2, *E* = -1919.9138709 Hartree), solvent: Chloroform

O	1.244947	2.711875	1.788440
O	-1.098658	3.442572	0.719597
N	-2.304051	1.615236	0.118556
N	-4.663470	-2.379737	-0.220504
N	1.111481	-0.760079	0.296540
N	5.222465	-0.149035	-1.441197
C	-5.363686	3.649371	0.832494
C	-6.302963	2.717616	0.402270
C	-5.889022	1.477701	-0.085261
C	-4.534286	1.192339	-0.144253
C	-3.604998	2.139992	0.284720
C	-3.996642	3.378880	0.781743
C	-3.843845	-0.084003	-0.573458
C	-2.374006	0.391693	-0.718258
C	-1.142985	2.291131	0.305002
C	0.097895	1.477216	-0.021587
C	-0.118661	0.020942	0.445113
C	-1.235507	-0.616780	-0.415877
C	-4.389825	-0.687887	-1.883087
C	-5.315192	-1.809184	-1.398270
C	-4.092156	-1.226105	0.497264
C	-2.817876	-1.607743	1.243086
C	-1.731496	-1.936243	0.209941
C	-0.075662	-3.233759	-2.050650
C	-1.536841	-3.469993	-1.788589
C	-2.267578	-2.940441	-0.805614

C	-3.674389	-3.423078	-0.526240
C	1.381511	2.167849	0.489024
C	2.537907	1.174435	0.577801
C	2.281603	-0.066656	-0.279107
C	1.608709	-1.606919	1.291303
C	3.424471	-1.109544	-0.169918
C	2.965873	-1.883175	1.046975
C	0.966174	-2.152107	2.398799
C	1.691456	-3.010579	3.230583
C	3.021949	-3.314327	2.974585
C	3.667108	-2.738810	1.873213
C	3.931436	1.790744	0.382840
C	4.946690	0.744270	0.845617
C	4.833549	-0.462641	-0.063232
C	4.849903	-1.373887	-2.161058
C	3.571528	-1.946942	-1.467583
C	4.537080	1.028909	-2.014765
C	4.263846	2.169000	-1.044440
C	4.321176	3.428029	-1.485979
C	3.990888	4.683580	-0.730115
H	-3.265489	4.102078	1.107028
H	-2.506005	-0.774625	1.880471
H	-4.844351	-0.858099	1.200605
H	-6.285269	-1.400698	-1.096008
H	-4.888162	0.061036	-2.501129
H	-3.624263	-4.093769	0.340298
H	-6.621324	0.749809	-0.416684
H	-7.359004	2.954346	0.445991
H	-5.695125	4.608922	1.211224
H	0.166268	1.454777	-1.118944

H	-0.408433	0.039196	1.503844
H	0.420998	-2.726242	-1.221676
H	0.430087	-4.188216	-2.218503
H	-2.261675	0.732849	-1.754185
H	-0.874437	-2.405302	0.698366
H	-4.044906	-4.024485	-1.358388
H	-2.026806	-4.181987	-2.450764
H	-3.566669	-1.122134	-2.458603
H	-5.500845	-2.577369	-2.149674
H	-3.019093	-2.456395	1.901046
H	-0.758020	-0.841844	-1.371649
H	0.076925	-2.634275	-2.955472
H	1.623105	2.964889	-0.228401
H	0.533731	3.363437	1.734726
H	3.514025	4.493592	0.231302
H	4.892269	5.278395	-0.553537
H	3.313573	5.306994	-1.319920
H	4.635198	3.582499	-2.517470
H	5.152544	1.406032	-2.835433
H	3.580258	0.729461	-2.469711
H	4.744739	0.453582	1.880298
H	5.965336	1.135718	0.791570
H	3.995839	2.670218	1.024926
H	2.511692	0.819917	1.616543
H	2.072722	0.189521	-1.318728
H	-0.066892	-1.920589	2.630074
H	1.199932	-3.440832	4.095384
H	3.563616	-3.982971	3.631801
H	4.712368	-2.957334	1.679150
H	3.703953	-3.004276	-1.231620

H	5.668641	-2.092060	-2.072381
H	2.679296	-1.861057	-2.091993
H	4.705085	-1.164210	-3.221780
H	5.520480	-1.246136	0.279744

Equilibrium geometries of molecules from set **1** calculated at the **DFT(M06-2X)** level of theory with **cc-pVDZ** basis set within the IEF-PCM model. Cartesian coordinates are given for standard orientation in form (atomic label, X, Y, Z). The X, Y, Z are given in Å.

12-28-oxaircinal A (*E* = -1271.9208811 Hartree), solvent: Chloroform

O	1.588344000	-0.787877000	1.832217000
O	-0.202501000	4.744429000	1.478362000
N	1.766595000	-0.505059000	-0.459400000
N	-2.178533000	0.471956000	-1.528408000
C	0.070263000	1.172532000	-0.861539000
C	0.392340000	-0.131856000	-0.098318000
C	0.397311000	-0.090772000	1.458753000
C	-0.582649000	2.292047000	-0.011192000
C	1.460636000	1.619259000	-1.379632000
C	2.492217000	0.764974000	-0.627074000
C	-0.856028000	0.807470000	-2.028289000
C	2.174710000	-1.353477000	0.646182000
C	-2.108623000	2.174402000	0.184934000
C	0.081995000	2.392524000	1.334684000
C	0.513180000	1.308369000	1.997690000
C	-0.856914000	-0.791245000	2.014132000
C	-2.826082000	1.692021000	-1.066886000
C	3.664496000	-1.515481000	0.866533000
C	3.801474000	0.694247000	-1.365093000
C	-2.967995000	-0.314660000	-2.464894000
C	-0.836836000	-2.316365000	1.827377000

C	4.354197000	-2.336023000	-0.223707000
C	0.201847000	3.716140000	1.976657000
C	4.476289000	-0.368454000	-1.818314000
C	4.107992000	-1.822613000	-1.653725000
C	-4.284096000	-0.817296000	-1.859415000
C	-2.193111000	-2.926930000	2.077522000
C	-4.171970000	-1.336680000	-0.423685000
C	-3.156639000	-2.469822000	-0.232171000
C	-3.182604000	-2.994906000	1.177943000
H	-0.299858000	-0.923269000	-0.414499000
H	-0.407686000	3.238348000	-0.547193000
H	1.641240000	2.693476000	-1.236777000
H	1.544262000	1.401561000	-2.455864000
H	2.686657000	1.241289000	0.357302000
H	-0.895472000	1.647506000	-2.757514000
H	-0.429669000	-0.063398000	-2.550945000
H	1.718391000	-2.347690000	0.478078000
H	-2.492452000	3.156048000	0.500926000
H	-2.326813000	1.467886000	0.998662000
H	0.971508000	1.404554000	2.988304000
H	-1.725150000	-0.386071000	1.470854000
H	-0.993033000	-0.548398000	3.079895000
H	-3.878245000	1.488948000	-0.827384000
H	-2.811992000	2.473665000	-1.858757000
H	3.787740000	-2.018069000	1.836923000
H	4.125235000	-0.521269000	0.963847000
H	4.237318000	1.684242000	-1.542020000
H	-2.346064000	-1.177728000	-2.752631000
H	-3.186298000	0.244944000	-3.400658000
H	-0.086000000	-2.751852000	2.501024000

H	-0.516451000	-2.558949000	0.803404000
H	4.012813000	-3.382583000	-0.164980000
H	5.435862000	-2.346570000	-0.016714000
H	0.698769000	3.714730000	2.975232000
H	5.423276000	-0.162248000	-2.327181000
H	3.051821000	-1.971024000	-1.910467000
H	4.705255000	-2.428163000	-2.350082000
H	-4.667553000	-1.616161000	-2.515069000
H	-5.042215000	-0.018767000	-1.887196000
H	-2.384084000	-3.315951000	3.082522000
H	-5.166152000	-1.684472000	-0.097643000
H	-3.895663000	-0.508933000	0.250814000
H	-2.154837000	-2.098828000	-0.494735000
H	-3.398131000	-3.288449000	-0.933469000
H	-4.131909000	-3.443479000	1.493186000

Anabsinthin (Conformer 1, *E* = -1618.1725207 Hartree), solvent: Acetonitrile

O	-2.306290000	1.192679000	-2.218831000
O	-2.554658000	-1.937236000	0.621198000
O	2.538634000	0.091681000	3.058738000
O	3.040351000	-1.694653000	-1.166075000
O	-3.764662000	-3.388805000	1.808710000
O	4.480076000	-2.211985000	-2.793667000
C	-1.639304000	0.183142000	-0.234272000
C	-1.968541000	1.682847000	0.032228000
C	-0.487452000	2.137710000	-0.031183000
C	-1.143992000	0.529013000	-1.677343000
C	-0.532434000	-0.116286000	0.810907000
C	0.146691000	1.257165000	1.060099000
C	-2.786524000	2.110671000	-1.204505000
C	-0.068540000	1.602049000	-1.420578000

C	-2.805122000	-0.811092000	-0.256721000
C	1.677520000	1.046401000	0.953081000
C	0.600725000	-1.029362000	0.417901000
C	-4.180550000	-0.301169000	0.172930000
C	-4.306652000	1.946735000	-1.032491000
C	1.778869000	-0.383625000	0.448379000
C	-0.719634000	-0.554771000	-2.639725000
C	-4.851760000	0.527263000	-0.910819000
C	2.516379000	1.271042000	2.256592000
C	-2.543765000	3.559398000	-1.633792000
C	-4.878222000	-1.612830000	0.534638000
C	3.097933000	-1.012716000	0.118211000
C	0.371353000	-2.479069000	0.123439000
C	3.989069000	1.575560000	1.931083000
C	-3.724621000	-2.435186000	1.076341000
C	4.280937000	-0.057575000	-0.024922000
C	4.814113000	0.451394000	1.305826000
C	1.970833000	2.445536000	3.074277000
C	-6.049445000	-1.537135000	1.498503000
C	5.236718000	-0.914797000	-0.854953000
C	4.266603000	-1.683335000	-1.734419000
C	6.305355000	-0.192796000	-1.656506000
H	-2.451339000	1.906790000	0.992634000
H	-0.313188000	3.210669000	0.119802000
H	-1.029313000	-0.509087000	1.710822000
H	-0.149967000	1.655357000	2.036596000
H	0.940258000	1.171111000	-1.427294000
H	-0.117129000	2.354146000	-2.217538000
H	-2.907376000	-1.227883000	-1.271827000
H	2.083530000	1.761713000	0.216802000

H	-4.070568000	0.292713000	1.098063000
H	-4.797948000	2.431276000	-1.890807000
H	-4.594186000	2.529426000	-0.141676000
H	-1.486846000	-1.332260000	-2.761932000
H	-0.542124000	-0.100155000	-3.625497000
H	0.217019000	-1.018252000	-2.301349000
H	-4.738854000	0.001351000	-1.872659000
H	-5.931497000	0.590626000	-0.706191000
H	-3.036444000	3.737820000	-2.600090000
H	-2.983560000	4.244847000	-0.893838000
H	-1.481635000	3.805499000	-1.738956000
H	-5.188954000	-2.113130000	-0.401941000
H	3.346957000	-1.777402000	0.875949000
H	-0.331882000	-2.605667000	-0.714261000
H	1.302114000	-3.004342000	-0.119142000
H	-0.109318000	-2.956010000	0.991012000
H	4.477130000	1.864279000	2.874630000
H	4.013536000	2.463430000	1.278283000
H	3.963790000	0.794524000	-0.653638000
H	4.881633000	-0.392810000	2.010721000
H	5.838300000	0.827728000	1.158515000
H	1.017917000	2.189435000	3.555751000
H	1.811156000	3.330719000	2.439751000
H	2.684388000	2.706150000	3.868086000
H	-5.748622000	-1.039963000	2.432350000
H	-6.405566000	-2.546097000	1.745286000
H	-6.881489000	-0.972731000	1.056351000
H	1.624869000	-0.214050000	3.159340000
H	5.702429000	-1.664915000	-0.188383000
H	6.856905000	-0.906150000	-2.283092000

H	7.019229000	0.310453000	-0.990607000
H	5.849802000	0.562301000	-2.313844000

Anabsinthin (Conformer 2, *E* = -1618.1729453 Hartree), solvent: Acetonitrile

O	-2.184590000	2.364790000	-0.931403000
O	-2.590178000	-1.843835000	-0.770986000
O	1.525040000	-1.835057000	2.949565000
O	3.032895000	-0.378048000	-2.019690000
O	-3.867874000	-3.673784000	-0.792029000
O	4.456433000	0.539781000	-3.477584000
C	-1.592461000	0.292518000	-0.061957000
C	-1.876831000	1.284120000	1.106115000
C	-0.379929000	1.617583000	1.340195000
C	-1.054298000	1.465785000	-0.949768000
C	-0.521728000	-0.653025000	0.544000000
C	0.196937000	0.220909000	1.612403000
C	-2.656162000	2.434972000	0.437475000
C	0.049426000	2.084467000	-0.069954000
C	-2.788573000	-0.408741000	-0.713848000
C	1.709958000	0.033597000	1.383819000
C	0.582423000	-1.129137000	-0.372294000
C	-4.154951000	-0.245478000	-0.048606000
C	-4.183853000	2.255186000	0.472394000
C	1.778327000	-0.685936000	0.051213000
C	-0.649162000	1.245274000	-2.388185000
C	-4.777659000	1.110129000	-0.342170000
C	2.392292000	-0.784782000	2.518404000
C	-2.359379000	3.811009000	1.037856000
C	-4.900577000	-1.456207000	-0.610989000
C	3.093635000	-0.867179000	-0.652882000

C	0.318396000	-1.994039000	-1.564292000
C	3.710503000	-1.451094000	2.073212000
C	-3.783711000	-2.475006000	-0.736026000
C	4.233924000	-0.059832000	-0.031210000
C	4.745468000	-0.621599000	1.300829000
C	2.604857000	0.103342000	3.735524000
C	-6.084110000	-1.977329000	0.185366000
C	5.209887000	0.009229000	-1.202980000
C	4.247788000	0.103832000	-2.376972000
C	6.236847000	1.127512000	-1.190829000
H	-2.369145000	0.857499000	1.990063000
H	-0.175296000	2.336027000	2.144196000
H	-1.047581000	-1.512371000	0.986021000
H	-0.082371000	-0.098622000	2.623539000
H	1.046219000	1.724456000	-0.354637000
H	0.037078000	3.173766000	-0.198496000
H	-2.884744000	-0.068647000	-1.757789000
H	2.217896000	1.010858000	1.344310000
H	-4.043677000	-0.390004000	1.041021000
H	-4.642771000	3.195367000	0.128384000
H	-4.469631000	2.140799000	1.531163000
H	-0.466372000	2.225293000	-2.853089000
H	0.280386000	0.662555000	-2.439135000
H	-1.430047000	0.737918000	-2.972198000
H	-4.664291000	1.320716000	-1.417981000
H	-5.858423000	1.068016000	-0.137259000
H	-2.790625000	3.876117000	2.048109000
H	-1.288153000	4.028157000	1.108901000
H	-2.827996000	4.586494000	0.415488000
H	-5.212082000	-1.224908000	-1.647078000

H	3.370549000	-1.934276000	-0.716203000
H	-0.132874000	-2.943960000	-1.239701000
H	-0.415572000	-1.527896000	-2.238530000
H	1.236197000	-2.197461000	-2.130128000
H	3.434823000	-2.323104000	1.456425000
H	4.184571000	-1.866244000	2.975625000
H	3.849326000	0.962335000	0.117200000
H	5.605290000	-1.281445000	1.101782000
H	5.128518000	0.200756000	1.923969000
H	2.934643000	-0.505344000	4.589153000
H	1.670947000	0.615570000	4.010620000
H	3.368709000	0.865799000	3.526960000
H	-6.475254000	-2.896245000	-0.271087000
H	-6.890917000	-1.232707000	0.215739000
H	-5.782775000	-2.206833000	1.218001000
H	1.325967000	-2.374549000	2.167978000
H	5.714680000	-0.969871000	-1.303282000
H	6.942019000	0.994106000	-0.359193000
H	5.742481000	2.103297000	-1.075174000
H	6.802800000	1.137340000	-2.131744000

Betulinic Acid (*E* = -1397.3091243 Hartree), Solvent: Pyridine

C	4.196388000	1.199897000	0.348716000
C	3.160446000	0.290345000	-0.349719000
C	3.792738000	-1.111364000	-0.375868000
C	5.248130000	-0.775957000	-0.753138000
C	5.561761000	0.478609000	0.084597000
C	1.721002000	0.255849000	0.160681000
C	0.839332000	-0.703640000	-0.698804000
C	1.537779000	-2.077638000	-0.888243000

C	3.004810000	-1.996328000	-1.339397000
C	1.076636000	1.638970000	0.260590000
C	-0.365548000	1.582746000	0.780514000
C	-1.248159000	0.532530000	0.083635000
C	-0.550574000	-0.864577000	0.057549000
C	-2.741169000	0.547471000	0.564473000
C	-3.505786000	-0.453630000	-0.353137000
C	-2.910399000	-1.858049000	-0.232995000
C	-1.448330000	-1.878679000	-0.679141000
C	-3.308431000	1.960082000	0.301566000
C	-4.829286000	2.031179000	0.394372000
C	-5.500453000	1.044524000	-0.542344000
C	-5.060090000	-0.416875000	-0.290774000
C	4.230075000	2.635353000	-0.131531000
C	4.271645000	3.651741000	0.737091000
C	4.273670000	2.871919000	-1.618903000
C	3.850697000	-1.751043000	1.007120000
O	3.774542000	-1.181343000	2.072193000
O	4.070603000	-3.076477000	0.938340000
C	0.659371000	-0.128151000	-2.121586000
C	-0.329133000	-1.401822000	1.490806000
C	-2.890257000	0.249092000	2.071660000
C	-5.626509000	-1.273079000	-1.436344000
C	-5.658513000	-0.946783000	1.018777000
O	-6.900308000	1.230731000	-0.389103000
H	3.999939000	1.203398000	1.428823000
H	3.143084000	0.592792000	-1.410129000
H	5.266731000	-0.543517000	-1.829551000
H	5.946356000	-1.605800000	-0.575288000
H	6.280567000	1.136644000	-0.423654000

H	6.011306000	0.193802000	1.045841000
H	1.767242000	-0.158985000	1.178628000
H	0.970822000	-2.666640000	-1.625207000
H	1.517830000	-2.658966000	0.046082000
H	3.089251000	-1.560195000	-2.346446000
H	3.432360000	-3.006616000	-1.388509000
H	1.107866000	2.136337000	-0.723489000
H	1.673287000	2.271705000	0.934059000
H	-0.814144000	2.579487000	0.666998000
H	-0.330254000	1.388892000	1.863839000
H	-1.331444000	0.840631000	-0.971035000
H	-3.270827000	-0.111548000	-1.381823000
H	-3.468229000	-2.565541000	-0.862051000
H	-3.008364000	-2.229952000	0.798807000
H	-1.434707000	-1.675413000	-1.760741000
H	-1.038324000	-2.892078000	-0.542196000
H	-2.997522000	2.284080000	-0.708039000
H	-2.874401000	2.678200000	1.012998000
H	-5.175381000	3.043895000	0.139390000
H	-5.176547000	1.835180000	1.421222000
H	-5.198510000	1.297340000	-1.579849000
H	4.348375000	4.687360000	0.398064000
H	4.225103000	3.473417000	1.814162000
H	3.293012000	2.669624000	-2.078581000
H	4.547705000	3.909680000	-1.848566000
H	4.997568000	2.200729000	-2.109234000
H	4.145929000	-3.396457000	1.854738000
H	0.248043000	0.886668000	-2.144626000
H	1.622676000	-0.088102000	-2.647524000
H	0.004367000	-0.767386000	-2.728882000

H	-1.233528000	-1.895684000	1.865729000
H	0.473655000	-2.149969000	1.524639000
H	-0.067580000	-0.618907000	2.212619000
H	-2.018495000	0.610760000	2.632811000
H	-3.765190000	0.762081000	2.491731000
H	-3.006820000	-0.818630000	2.294360000
H	-5.145888000	-1.026179000	-2.396211000
H	-5.494228000	-2.347538000	-1.249423000
H	-6.710807000	-1.112468000	-1.545071000
H	-6.751744000	-1.008898000	0.922246000
H	-5.278601000	-1.956068000	1.238245000
H	-5.442908000	-0.306849000	1.882587000
H	-7.348384000	0.706253000	-1.065278000

Icajine (Conformer 1, *E* = -1187.6111874 Hartree), solvent: Chloroform

O	-0.142283000	-2.888550000	1.206513000
O	2.085808000	3.015865000	0.745730000
O	-2.525349000	3.208004000	-0.456535000
N	-1.440264000	1.197110000	-0.515231000
N	2.089603000	-2.086643000	-0.563954000
C	-0.150398000	0.514160000	-0.315797000
C	0.204873000	-1.748751000	0.961547000
C	1.365414000	-1.731973000	-1.777864000
C	-0.142241000	-1.754798000	-1.507320000
C	-0.490492000	-0.997454000	-0.203972000
C	-2.001177000	-0.972649000	-0.015230000
C	-2.869047000	-2.015518000	0.281344000
C	-4.243494000	-1.757821000	0.335861000
C	-4.730990000	-0.474030000	0.089238000

C	-3.870536000	0.585931000	-0.212680000
C	-2.504179000	0.312775000	-0.255608000
C	1.197334000	-1.035022000	1.868695000
C	1.741163000	0.290589000	1.319831000
C	0.503294000	1.106267000	0.929772000
C	0.723111000	2.631155000	0.776910000
C	2.860313000	2.662836000	-0.378843000
C	3.173030000	1.203935000	-0.549713000
C	2.734879000	0.157217000	0.160685000
C	3.212308000	-1.230463000	-0.210344000
C	-0.104920000	3.196605000	-0.396228000
C	-1.487075000	2.568952000	-0.456216000
C	2.345283000	-3.505807000	-0.434094000
H	0.485182000	0.697590000	-1.191053000
H	1.613024000	-2.415515000	-2.610278000
H	1.679175000	-0.726971000	-2.093699000
H	-0.496719000	-2.789950000	-1.387073000
H	-0.695154000	-1.312135000	-2.350582000
H	-2.470112000	-3.010093000	0.477277000
H	-4.936139000	-2.566498000	0.570400000
H	-5.804712000	-0.286497000	0.132412000
H	-4.237444000	1.590752000	-0.401790000
H	1.980464000	-1.762099000	2.121216000
H	0.662218000	-0.819946000	2.808458000
H	2.255783000	0.813344000	2.139818000
H	-0.235660000	0.980137000	1.739848000
H	0.363510000	3.110900000	1.696114000
H	3.808181000	3.209012000	-0.254522000
H	2.417142000	3.050731000	-1.315360000
H	3.869448000	1.006825000	-1.372934000

H	3.705585000	-1.694393000	0.659535000
H	3.973002000	-1.162542000	-1.012820000
H	-0.220417000	4.282395000	-0.317575000
H	0.392217000	2.968682000	-1.351624000
H	3.086245000	-3.876354000	-1.169385000
H	1.403129000	-4.056611000	-0.567544000
H	2.712547000	-3.723404000	0.579946000

Icajine (Conformer 2, *E* = -1187.6116292 Hartree), solvent: Chloroform

O	-0.031396000	-2.790147000	1.303100000
O	1.591497000	2.919439000	-0.590867000
O	-2.891142000	3.004352000	-0.221787000
N	-1.544418000	1.178764000	-0.365736000
N	2.202133000	-2.014124000	-0.478826000
C	-0.193176000	0.583012000	-0.281608000
C	0.279978000	-1.650438000	1.016544000
C	1.509929000	-1.629073000	-1.700785000
C	0.002304000	-1.696373000	-1.470088000
C	-0.424009000	-0.956761000	-0.177413000
C	-1.934658000	-1.064446000	-0.057041000
C	-2.714698000	-2.198222000	0.131680000
C	-4.107643000	-2.071616000	0.121978000
C	-4.697490000	-0.824888000	-0.085715000
C	-3.924931000	0.324172000	-0.278655000
C	-2.536675000	0.182564000	-0.251985000
C	1.240223000	-0.870052000	1.903543000
C	1.789538000	0.426677000	1.290545000
C	0.549920000	1.238097000	0.877328000
C	0.755237000	2.717308000	0.533397000
C	2.968136000	2.693861000	-0.337091000

C	3.371984000	1.251547000	-0.486680000
C	2.844865000	0.229789000	0.199315000
C	3.321233000	-1.173921000	-0.092781000
C	-0.583701000	3.387881000	0.208587000
C	-1.774046000	2.514617000	-0.160382000
C	2.445053000	-3.435906000	-0.363074000
H	0.353325000	0.819682000	-1.206626000
H	1.803201000	-0.603130000	-1.964518000
H	1.796513000	-2.272717000	-2.553041000
H	-0.321269000	-2.743382000	-1.362750000
H	-0.543640000	-1.272050000	-2.327129000
H	-2.234214000	-3.161669000	0.296697000
H	-4.732637000	-2.952402000	0.271621000
H	-5.784713000	-0.736596000	-0.099971000
H	-4.376123000	1.298190000	-0.437710000
H	0.676536000	-0.605609000	2.813709000
H	2.024068000	-1.570327000	2.220187000
H	2.287394000	0.987474000	2.101507000
H	-0.127109000	1.225586000	1.750474000
H	1.211195000	3.224454000	1.403734000
H	3.221386000	3.066319000	0.674949000
H	3.514002000	3.310022000	-1.062142000
H	4.139309000	1.028381000	-1.233544000
H	3.757685000	-1.615757000	0.818227000
H	4.126326000	-1.142872000	-0.853595000
H	-0.942296000	3.974525000	1.065307000
H	-0.420925000	4.103138000	-0.609675000
H	3.199859000	-3.801365000	-1.087012000
H	1.503633000	-3.980253000	-0.523990000
H	2.788589000	-3.668262000	0.655930000

Iguesterin (*E* = -1238.670903 Hartree), solvent: Chloroform

O	-6.527328000	0.829643000	-0.979077000
O	-5.267134000	2.652532000	0.412645000
C	1.666853000	0.209223000	0.441437000
C	1.057122000	-1.238315000	0.557610000
C	3.211837000	0.147559000	0.640403000
C	3.976284000	-0.891188000	-0.237705000
C	1.741549000	-2.155229000	-0.475671000
C	1.018900000	1.057232000	1.553349000
C	3.252904000	-2.246488000	-0.265925000
C	-0.450646000	-1.131747000	0.312537000
C	-1.276036000	-0.089604000	1.069949000
C	-0.491071000	1.212999000	1.367259000
C	3.907557000	1.519876000	0.540728000
C	1.292310000	0.850872000	-0.908427000
C	4.189779000	-0.420426000	-1.692068000
C	1.292013000	-1.886183000	1.944432000
C	5.367869000	-1.105003000	0.378555000
C	4.372730000	1.924117000	-0.836535000
C	-2.606491000	0.238838000	0.390403000
C	4.503040000	1.039115000	-1.830070000
C	-1.094309000	-1.973789000	-0.528305000
C	-1.732001000	-0.737196000	2.418608000
C	-3.248906000	-0.774806000	-0.443216000
C	-2.496746000	-1.843590000	-0.835708000
C	4.702585000	3.380316000	-1.003974000
C	-3.270255000	1.390851000	0.673346000
C	-4.622679000	-0.600170000	-0.904180000
C	-4.621612000	1.623145000	0.195304000

C	-5.264097000	0.551306000	-0.592436000
C	-5.295902000	-1.659137000	-1.730814000
H	3.347659000	-0.187726000	1.682281000
H	1.325605000	-3.171368000	-0.402090000
H	1.516347000	-1.798843000	-1.492865000
H	1.257381000	0.634614000	2.539709000
H	1.440452000	2.071924000	1.552721000
H	3.692089000	-2.857225000	-1.072238000
H	3.466387000	-2.792181000	0.665357000
H	-0.666642000	1.915801000	0.541095000
H	-0.916590000	1.685366000	2.264840000
H	3.258758000	2.316481000	0.934743000
H	4.788845000	1.529176000	1.206854000
H	0.210125000	0.800329000	-1.096773000
H	1.582385000	1.913169000	-0.910506000
H	1.787897000	0.376559000	-1.760393000
H	5.022325000	-1.011091000	-2.113660000
H	3.323193000	-0.677224000	-2.325207000
H	2.358321000	-2.054740000	2.135399000
H	0.914204000	-1.294213000	2.780873000
H	0.787269000	-2.863849000	1.970460000
H	5.293100000	-1.346055000	1.450762000
H	6.000802000	-0.212444000	0.264814000
H	5.876435000	-1.942638000	-0.123769000
H	4.852377000	1.386385000	-2.807910000
H	-0.558287000	-2.772086000	-1.037201000
H	-0.887483000	-0.896471000	3.096380000
H	-2.228804000	-1.700946000	2.237158000
H	-2.443944000	-0.057920000	2.907967000
H	-2.943344000	-2.607373000	-1.474394000

H	5.442846000	3.700958000	-0.252764000
H	3.805027000	4.001117000	-0.846600000
H	5.104103000	3.594472000	-2.003398000
H	-2.843761000	2.184508000	1.285889000
H	-5.295853000	-2.629032000	-1.211550000
H	-4.781161000	-1.800395000	-2.693536000
H	-6.333547000	-1.373988000	-1.935350000
H	-6.694291000	1.724079000	-0.618937000

Itoaic Acid (Conformer 1, *E* = -1622.9430543 Hartree), solvent: Chloroform

C	-3.417557000	-1.107918000	-0.275922000
C	-2.566016000	0.162200000	0.062424000
C	-1.258276000	-0.110935000	0.901912000
C	-0.419841000	-1.140136000	0.063445000
C	-1.216966000	-2.431634000	-0.126093000
C	-2.494146000	-2.162098000	-0.911552000
C	-0.432991000	1.207626000	1.005210000
C	1.057076000	1.008094000	1.310424000
C	1.784480000	0.043405000	0.358074000
C	1.067643000	-1.349307000	0.481962000
C	3.303131000	-0.070659000	0.757398000
C	4.098456000	-1.288977000	0.180554000
C	3.243254000	-2.560706000	-0.047787000
C	1.775996000	-2.368054000	-0.434555000
C	4.136033000	1.246050000	0.663764000
C	5.083625000	1.528056000	-0.538289000
C	5.773783000	0.238585000	-1.004999000
C	4.798400000	-0.923703000	-1.144769000
C	-3.397621000	1.329210000	0.613946000
C	-4.459103000	1.919152000	-0.326650000

C	-5.723350000	1.077346000	-0.305589000
O	-5.677565000	-0.177173000	-0.744458000
C	-4.483372000	-0.734008000	-1.348400000
O	-4.788492000	3.221705000	0.080760000
O	-6.756229000	1.515696000	0.147810000
C	-5.025186000	-1.889459000	-2.177290000
C	-4.146312000	-1.723282000	0.928857000
C	-1.602973000	-0.571682000	2.335680000
O	-0.943543000	2.133255000	1.957012000
C	1.167589000	-1.921131000	1.911451000
C	5.169007000	-1.665214000	1.222085000
C	6.145275000	2.519732000	-0.045162000
C	4.397106000	2.178435000	-1.748485000
C	1.690299000	0.616425000	-1.061399000
O	1.541574000	1.954954000	-1.058406000
O	1.772330000	0.020032000	-2.112106000
H	-2.183689000	0.506410000	-0.918792000
H	-0.361631000	-0.697327000	-0.948109000
H	-1.448328000	-2.907121000	0.839931000
H	-0.630620000	-3.162963000	-0.695355000
H	-3.050474000	-3.101737000	-1.043781000
H	-2.203474000	-1.817391000	-1.919991000
H	-0.521537000	1.730644000	0.041546000
H	1.166260000	0.630638000	2.338815000
H	1.508808000	2.006101000	1.298759000
H	3.225284000	-0.256522000	1.840317000
H	3.281352000	-3.178129000	0.861269000
H	3.737818000	-3.165610000	-0.824389000
H	1.290915000	-3.348810000	-0.329181000
H	1.676284000	-2.064471000	-1.481773000

H	4.782494000	1.247087000	1.555324000
H	3.475252000	2.117522000	0.790068000
H	6.572250000	-0.033035000	-0.296347000
H	6.274186000	0.431671000	-1.968294000
H	5.343041000	-1.815555000	-1.494876000
H	4.038472000	-0.701398000	-1.909632000
H	-2.737864000	2.171503000	0.837417000
H	-3.888218000	1.075759000	1.568203000
H	-4.062109000	1.959157000	-1.355888000
H	-4.054301000	0.010929000	-2.037499000
H	-5.707744000	3.169451000	0.396258000
H	-4.258170000	-2.297054000	-2.844812000
H	-5.405875000	-2.697253000	-1.536600000
H	-5.856497000	-1.517687000	-2.789602000
H	-4.646489000	-0.968213000	1.550750000
H	-4.922320000	-2.420626000	0.580534000
H	-3.460376000	-2.290207000	1.568292000
H	-2.570914000	-0.167856000	2.659575000
H	-0.859307000	-0.226401000	3.068691000
H	-1.647598000	-1.661901000	2.437087000
H	-0.799196000	1.763162000	2.838726000
H	0.633455000	-2.883669000	1.942996000
H	0.738671000	-1.283860000	2.686388000
H	2.205280000	-2.123138000	2.201589000
H	5.812406000	-0.819215000	1.499702000
H	4.690158000	-2.033076000	2.142853000
H	5.814054000	-2.469147000	0.832250000
H	6.860968000	2.750920000	-0.849377000
H	5.680089000	3.464501000	0.278825000
H	6.708469000	2.106202000	0.805742000

H	5.169633000	2.503730000	-2.462804000
H	3.736937000	1.482736000	-2.284135000
H	3.817883000	3.063605000	-1.447517000
H	1.567058000	2.237435000	-1.989527000

Itoaic Acid (Conformer 2, *E* = -1622.9423197 Hartree), solvent: Chloroform

C	-3.488958000	-1.105289000	0.149714000
C	-2.639232000	0.205760000	0.017641000
C	-1.241393000	0.175566000	0.750073000
C	-0.487848000	-1.061017000	0.148633000
C	-1.263341000	-2.343476000	0.452469000
C	-2.616141000	-2.312081000	-0.245378000
C	-0.420760000	1.447892000	0.381859000
C	1.082463000	1.319994000	0.652325000
C	1.743960000	0.132515000	-0.066933000
C	1.042803000	-1.180007000	0.411857000
C	3.280334000	0.064771000	0.261337000
C	3.983112000	-1.278502000	-0.186732000
C	3.027476000	-2.193078000	-0.969514000
C	1.630428000	-2.401078000	-0.359002000
C	4.058501000	1.302300000	-0.220766000
C	5.563218000	1.285486000	0.104533000
C	6.195725000	-0.038541000	-0.415324000
C	5.173301000	-0.938949000	-1.105096000
C	-3.441390000	1.483685000	0.304060000
C	-4.610554000	1.786812000	-0.644654000
C	-5.843421000	0.990599000	-0.253497000
O	-5.809984000	-0.336950000	-0.322418000
C	-4.669495000	-1.048422000	-0.865379000
O	-4.930081000	3.152876000	-0.587571000

O	-6.836149000	1.543560000	0.163036000
C	-5.264729000	-2.389907000	-1.267799000
C	-4.074391000	-1.336947000	1.551612000
C	-1.408953000	0.157728000	2.285532000
O	-0.868601000	2.635149000	1.023358000
C	1.292134000	-1.424617000	1.914271000
C	4.538990000	-2.076646000	1.012934000
C	5.796400000	1.446964000	1.610639000
C	6.207257000	2.477273000	-0.611850000
C	1.505748000	0.371627000	-1.571975000
O	1.704680000	1.658125000	-1.921965000
O	1.144780000	-0.431492000	-2.400718000
H	-2.372661000	0.255468000	-1.056338000
H	-0.568795000	-0.930406000	-0.942329000
H	-1.396614000	-2.488842000	1.535015000
H	-0.707601000	-3.220885000	0.094514000
H	-3.166850000	-3.241036000	-0.036363000
H	-2.436884000	-2.281977000	-1.334805000
H	-0.565855000	1.645050000	-0.694299000
H	1.255664000	1.223754000	1.735293000
H	1.532299000	2.269897000	0.344620000
H	3.304052000	0.096412000	1.361791000
H	3.516149000	-3.174727000	-1.083634000
H	2.914142000	-1.794029000	-1.984397000
H	1.649556000	-3.260626000	0.329499000
H	0.956648000	-2.677448000	-1.181569000
H	3.619334000	2.211421000	0.218322000
H	3.953610000	1.412626000	-1.308245000
H	6.654266000	-0.588774000	0.421473000
H	7.015392000	0.187437000	-1.114409000

H	5.649496000	-1.879918000	-1.425131000
H	4.803557000	-0.453932000	-2.023471000
H	-2.784268000	2.351398000	0.206797000
H	-3.822596000	1.513182000	1.338164000
H	-4.323244000	1.532160000	-1.679577000
H	-4.333371000	-0.534222000	-1.780001000
H	-5.812466000	3.195112000	-0.179082000
H	-4.560993000	-2.975974000	-1.868765000
H	-5.556807000	-2.977939000	-0.386425000
H	-6.163909000	-2.204219000	-1.868741000
H	-4.524198000	-0.429371000	1.976862000
H	-4.865951000	-2.099327000	1.506009000
H	-3.312820000	-1.697630000	2.252101000
H	-2.327472000	0.676705000	2.590684000
H	-0.575292000	0.663927000	2.794950000
H	-1.448393000	-0.854596000	2.702485000
H	-0.701918000	2.538416000	1.971418000
H	0.707730000	-2.300162000	2.233050000
H	1.004203000	-0.586508000	2.555811000
H	2.340963000	-1.649838000	2.125727000
H	4.951170000	-1.432019000	1.802752000
H	3.775855000	-2.721610000	1.467845000
H	5.347548000	-2.741348000	0.669560000
H	5.338273000	0.632599000	2.191297000
H	6.874886000	1.447538000	1.833526000
H	5.373923000	2.398524000	1.971224000
H	6.086644000	2.387200000	-1.702907000
H	5.746440000	3.426198000	-0.294604000
H	7.284925000	2.528028000	-0.391171000
H	1.527623000	1.711680000	-2.877820000

Matopensine (*E* = -1766.9826817 Hartree), solvent: Chloroform

O	0.000029000	0.000024000	2.586211000
N	1.340638000	0.878493000	0.927267000
N	3.816775000	-2.202065000	-1.057765000
C	2.076600000	-0.363783000	1.224651000
C	3.341821000	-0.256550000	0.335153000
C	1.119438000	-1.549965000	1.033225000
C	0.205837000	1.156378000	1.789441000
C	3.114325000	-0.909758000	-1.072240000
C	0.955686000	-1.987024000	-0.457880000
C	1.641514000	-1.010796000	-1.433620000
C	4.579325000	-1.007485000	0.875920000
C	3.490741000	1.249861000	0.246962000
C	2.281477000	1.857929000	0.625731000
C	5.036319000	-1.880315000	-0.312804000
C	1.632768000	-3.315931000	-0.724930000
C	3.099710000	-3.304139000	-0.383643000
C	4.568113000	2.029242000	-0.137772000
C	2.143304000	3.243292000	0.659985000
C	1.080293000	-4.377793000	-1.327143000
C	4.444093000	3.428439000	-0.119167000
C	3.248041000	4.019278000	0.281288000
C	-0.320858000	-4.535695000	-1.837321000
H	2.404719000	-0.343453000	2.280769000
H	1.532031000	-2.396383000	1.599617000
H	0.465366000	1.950037000	2.509072000
H	3.638291000	-0.283000000	-1.811973000
H	-0.108438000	-2.047180000	-0.706868000
H	1.533910000	-1.404282000	-2.454770000

H	1.189306000	-0.010532000	-1.397579000
H	4.298504000	-1.632710000	1.737372000
H	5.361909000	-0.318588000	1.219981000
H	5.706260000	-1.303614000	-0.969981000
H	5.562775000	-2.795843000	-0.010774000
H	3.567813000	-4.249405000	-0.694757000
H	3.217113000	-3.241703000	0.717942000
H	5.510364000	1.563513000	-0.436885000
H	1.202703000	3.714537000	0.954815000
H	1.737983000	-5.237053000	-1.503728000
H	5.288693000	4.052760000	-0.410854000
H	3.163631000	5.107003000	0.301101000
H	-0.296126000	-4.836876000	-2.896247000
H	-0.854199000	-5.333973000	-1.296164000
H	-0.917081000	-3.619071000	-1.755974000
N	-1.340601000	-0.878458000	0.927301000
N	-3.816833000	2.202051000	-1.057682000
C	-2.076577000	0.363805000	1.224686000
C	-3.341817000	0.256541000	0.335212000
C	-1.119425000	1.549994000	1.033223000
C	-0.205797000	-1.156339000	1.789463000
C	-3.114368000	0.909754000	-1.072182000
C	-0.955721000	1.987044000	-0.457894000
C	-1.641569000	1.010807000	-1.433610000
C	-4.579326000	1.007444000	0.876009000
C	-3.490698000	-1.249874000	0.247002000
C	-2.281409000	-1.857913000	0.625735000
C	-5.036357000	1.880282000	-0.312694000
C	-1.632836000	3.315935000	-0.724948000
C	-3.099758000	3.304132000	-0.383577000

C	-4.568042000	-2.029277000	-0.137768000
C	-2.143176000	-3.243271000	0.659902000
C	-1.080430000	4.377779000	-1.327257000
C	-4.443964000	-3.428471000	-0.119240000
C	-3.247881000	-4.019282000	0.281166000
C	0.320668000	4.535695000	-1.837574000
H	-2.404679000	0.343484000	2.280808000
H	-1.532002000	2.396414000	1.599624000
H	-0.465312000	-1.949997000	2.509103000
H	-3.638348000	0.282994000	-1.811903000
H	0.108395000	2.047208000	-0.706914000
H	-1.534006000	1.404295000	-2.454764000
H	-1.189346000	0.010550000	-1.397586000
H	-4.298497000	1.632664000	1.737462000
H	-5.361890000	0.318530000	1.220080000
H	-5.706305000	1.303577000	-0.969862000
H	-5.562819000	2.795800000	-0.010646000
H	-3.567884000	4.249394000	-0.694670000
H	-3.217114000	3.241688000	0.718011000
H	-5.510312000	-1.563567000	-0.436851000
H	-1.202546000	-3.714493000	0.954681000
H	-1.738152000	5.237019000	-1.503818000
H	-5.288538000	-4.052812000	-0.410960000
H	-3.163415000	-5.107005000	0.300900000
H	0.295812000	4.836780000	-2.896525000
H	0.854029000	5.334045000	-1.296548000
H	0.916939000	3.619103000	-1.756208000

Naucleidinal (*E* = -1109.0425606 Hartree), solvent: Chloroform

O	-3.464341000	1.456619000	1.300643000

O	-1.223556000	-2.000149000	1.042207000
O	-6.788736000	-0.172813000	-1.580769000
N	-2.994083000	-3.230720000	0.326111000
N	-5.075102000	-2.922539000	-2.697093000
C	-4.333882000	-3.299154000	-0.267496000
C	-4.567646000	-0.841856000	-0.086812000
C	-5.235381000	-2.175789000	0.218154000
C	-5.401863000	0.369015000	0.305644000
C	-4.142187000	-3.318994000	-1.761654000
C	-3.248012000	-0.821679000	0.634267000
C	-2.123458000	-4.320688000	-0.115179000
C	-2.987815000	-3.729408000	-2.375243000
C	-2.397004000	-2.040941000	0.686161000
C	-1.774509000	-4.173402000	-1.607755000
C	-4.507277000	1.622664000	0.328627000
C	-3.200431000	-3.564657000	-3.789918000
C	-2.823667000	0.272468000	1.286654000
C	-4.511773000	-3.042005000	-3.950538000
C	-6.587627000	0.535502000	-0.621199000
C	-5.218129000	2.909602000	0.690461000
C	-2.413445000	-3.796360000	-4.931681000
C	-5.043214000	-2.739254000	-5.209688000
C	-2.938245000	-3.499306000	-6.181080000
C	-4.240017000	-2.973205000	-6.317559000
H	-4.771928000	-4.258578000	0.054195000
H	-4.400367000	-0.787937000	-1.175684000
H	-6.217011000	-2.260754000	-0.268166000
H	-5.387807000	-2.279763000	1.303334000
H	-5.809910000	0.253758000	1.328037000
H	-2.666232000	-5.261881000	0.055483000

H	-1.222159000	-4.313783000	0.504175000
H	-1.386244000	-5.131851000	-1.985595000
H	-0.965048000	-3.433700000	-1.724625000
H	-4.018458000	1.713815000	-0.657488000
H	-5.912961000	-2.389001000	-2.488605000
H	-1.908120000	0.276469000	1.878528000
H	-7.300505000	1.352200000	-0.370437000
H	-5.908190000	3.217537000	-0.105778000
H	-5.781380000	2.789797000	1.627112000
H	-4.475227000	3.705754000	0.827411000
H	-1.404508000	-4.200917000	-4.832849000
H	-6.050632000	-2.334258000	-5.313460000
H	-2.337882000	-3.672829000	-7.074936000
H	-4.624229000	-2.747531000	-7.313018000

Physalin D (*E* = -1913.2048605 Hartree), solvent: Dimethylsulfoxide

C	-6.153381000	0.491558000	-0.619035000
C	-5.536781000	-0.858676000	-0.404644000
C	-4.014099000	-0.820700000	-0.547315000
C	-3.390745000	0.350752000	0.274535000
C	-3.966476000	1.616598000	-0.379200000
C	-5.436855000	1.625826000	-0.607113000
C	-3.395792000	-2.171805000	-0.191367000
C	-1.883325000	-2.133780000	-0.337489000
C	-1.244836000	-1.025435000	0.511324000
C	-1.843070000	0.353645000	0.142176000
O	-3.288267000	2.570922000	-0.712861000
C	0.301723000	-1.064903000	0.385255000
C	1.117576000	-0.784486000	1.667384000
C	2.369756000	-0.044154000	1.223103000

C	1.775263000	0.769696000	0.066137000
C	1.216094000	2.124677000	0.590556000
C	-0.223843000	2.405804000	0.171616000
C	-1.221784000	1.520557000	0.941869000
O	0.810672000	-1.134646000	2.775911000
O	0.780374000	-0.077061000	-0.521684000
O	0.656297000	-2.337157000	-0.091434000
C	3.535553000	-0.983943000	0.834542000
C	3.141294000	-2.138261000	-0.117446000
C	1.895080000	-2.933885000	0.272149000
C	4.572790000	-0.118625000	0.116436000
C	4.009950000	0.358110000	-1.205517000
C	2.739426000	1.215286000	-1.072831000
C	2.240566000	3.134768000	0.066533000
O	3.188359000	2.529360000	-0.672120000
O	2.276078000	4.301355000	0.344948000
C	3.090205000	-1.835082000	-1.610268000
O	3.722602000	-0.741410000	-2.080717000
C	2.038707000	1.361581000	-2.418807000
O	2.597772000	-2.596808000	-2.401190000
C	4.123935000	-1.559173000	2.124933000
O	-3.667123000	-0.646672000	-1.923748000
O	-3.794729000	-2.511120000	1.127396000
C	-3.847965000	0.347118000	1.750334000
O	1.321451000	2.203868000	2.006139000
H	-7.227640000	0.534055000	-0.816199000
H	-5.937017000	-1.570147000	-1.144330000
H	-5.817925000	-1.251948000	0.583967000
H	-5.885290000	2.597587000	-0.820838000
H	-3.806556000	-2.899868000	-0.914143000

H	-1.634597000	-1.982762000	-1.398063000
H	-1.483975000	-3.113346000	-0.048401000
H	-1.456939000	-1.243200000	1.572308000
H	-1.635322000	0.514219000	-0.925593000
H	2.716614000	0.595915000	2.037413000
H	-0.354953000	2.282968000	-0.911289000
H	-0.407305000	3.467666000	0.397649000
H	-2.018078000	2.182434000	1.292271000
H	-0.744572000	1.140132000	1.858744000
H	3.977110000	-2.859132000	-0.058129000
H	1.914687000	-3.159461000	1.350011000
H	1.898918000	-3.881753000	-0.274519000
H	4.839828000	0.752707000	0.732465000
H	5.490815000	-0.694967000	-0.070857000
H	4.745632000	0.959958000	-1.753019000
H	2.769990000	1.715611000	-3.158443000
H	1.232254000	2.103798000	-2.347534000
H	1.614793000	0.410797000	-2.754463000
H	4.493002000	-0.743735000	2.763320000
H	3.373146000	-2.119532000	2.700360000
H	4.965054000	-2.230109000	1.899181000
H	-4.266526000	0.011328000	-2.305853000
H	-3.434736000	-3.387911000	1.321385000
H	-3.524429000	1.264934000	2.258531000
H	-4.941378000	0.300398000	1.833212000
H	-3.434403000	-0.517369000	2.280559000
H	1.098104000	3.117276000	2.248086000

Strychnobaillonine (Conformer 1, *E* = -1919.6242756 Hartree), solvent: Chloroform

O	1.200105000	2.841393000	1.714747000

O	-1.241769000	3.446965000	0.828845000
N	-2.378732000	1.614175000	0.105071000
N	-4.188896000	-2.421767000	-1.222191000
N	1.064405000	-0.656687000	0.359419000
N	5.099162000	-0.132269000	-1.529564000
C	-5.468011000	3.210004000	1.493354000
C	-6.382964000	2.262641000	1.031628000
C	-5.940950000	1.168560000	0.281833000
C	-4.585348000	1.039166000	0.005559000
C	-3.682491000	1.991788000	0.479577000
C	-4.101195000	3.094677000	1.225193000
C	-3.889275000	-0.048023000	-0.780468000
C	-2.395776000	0.432901000	-0.793939000
C	-1.240344000	2.325327000	0.326826000
C	0.020941000	1.563598000	-0.044572000
C	-0.175155000	0.108488000	0.446654000
C	-1.251242000	-0.569068000	-0.434200000
C	-4.506305000	-0.249217000	-2.194312000
C	-5.034822000	-1.703006000	-2.177640000
C	-4.090943000	-1.453917000	-0.123556000
C	-3.025046000	-1.788158000	0.902295000
C	-1.679267000	-1.923375000	0.187838000
C	0.145443000	-4.474474000	-0.115186000
C	-1.031209000	-4.089579000	-0.961910000
C	-1.806803000	-3.002716000	-0.859855000
C	-2.895149000	-2.723836000	-1.857763000
C	1.307050000	2.280299000	0.422936000
C	2.482869000	1.303480000	0.494977000
C	2.212730000	0.010136000	-0.277492000
C	1.584952000	-1.415115000	1.410938000

C	3.366271000	-1.015869000	-0.123576000
C	2.943268000	-1.699334000	1.160969000
C	0.964538000	-1.882437000	2.569784000
C	1.711892000	-2.679789000	3.447585000
C	3.042278000	-2.996653000	3.186942000
C	3.665975000	-2.494776000	2.033583000
C	3.859777000	1.920866000	0.203905000
C	4.904850000	0.918301000	0.698486000
C	4.769838000	-0.351338000	-0.118234000
C	4.709375000	-1.404963000	-2.148109000
C	3.479156000	-1.950600000	-1.355255000
C	4.385360000	0.998970000	-2.155736000
C	4.137464000	2.202761000	-1.258103000
C	4.169888000	3.432837000	-1.791283000
C	3.859194000	4.737549000	-1.117780000
H	-3.386182000	3.832823000	1.575111000
H	-2.992315000	-0.987182000	1.660487000
H	-5.079500000	-1.439352000	0.362344000
H	-6.073444000	-1.728251000	-1.814172000
H	-5.303127000	0.478661000	-2.393518000
H	-3.035301000	-3.582277000	-2.529904000
H	-6.653578000	0.427977000	-0.088601000
H	-7.444377000	2.377230000	1.252136000
H	-5.821151000	4.063235000	2.073696000
H	0.061196000	1.527431000	-1.148146000
H	-0.507063000	0.138907000	1.499090000
H	1.053419000	-4.540835000	-0.736107000
H	0.349776000	-3.769757000	0.700070000
H	-2.186967000	0.812214000	-1.806380000
H	-0.909944000	-2.222217000	0.905210000

H	-2.555760000	-1.874857000	-2.493683000
H	-1.266107000	-4.785406000	-1.776218000
H	-3.742620000	-0.112519000	-2.973035000
H	-5.008429000	-2.186865000	-3.163133000
H	-3.276485000	-2.727949000	1.413003000
H	-0.725353000	-0.790197000	-1.375844000
H	-0.007037000	-5.473161000	0.323037000
H	1.521368000	3.069018000	-0.323877000
H	0.432270000	3.437211000	1.679498000
H	3.087177000	5.281230000	-1.684391000
H	3.496118000	4.618185000	-0.089297000
H	4.748195000	5.387744000	-1.097600000
H	4.445118000	3.510869000	-2.850074000
H	4.969921000	1.325359000	-3.029212000
H	3.409403000	0.664292000	-2.560444000
H	4.744654000	0.697016000	1.764080000
H	5.922449000	1.315758000	0.575313000
H	3.935590000	2.849604000	0.783488000
H	2.509065000	1.004258000	1.557066000
H	1.977828000	0.202447000	-1.331593000
H	-0.073042000	-1.639983000	2.802851000
H	1.235110000	-3.052588000	4.355254000
H	3.602576000	-3.620980000	3.882772000
H	4.715286000	-2.723085000	1.833227000
H	3.656529000	-2.986776000	-1.036301000
H	5.548672000	-2.109877000	-2.056613000
H	2.549265000	-1.942518000	-1.942364000
H	4.509660000	-1.265285000	-3.218883000
H	5.483993000	-1.107193000	0.252435000

Strychnobaillonine (Conformer 2, *E* = -1919.6253359 Hartree), solvent: Chloroform

O	1.255072000	2.746147000	1.754173000
O	-1.107106000	3.444723000	0.729365000
N	-2.309573000	1.613882000	0.117562000
N	-4.655943000	-2.392255000	-0.234687000
N	1.109173000	-0.757296000	0.295730000
N	5.239804000	-0.165095000	-1.416762000
C	-5.375034000	3.642327000	0.854700000
C	-6.316581000	2.706669000	0.425565000
C	-5.900714000	1.466532000	-0.069483000
C	-4.541919000	1.184360000	-0.137704000
C	-3.610832000	2.135905000	0.291122000
C	-4.003968000	3.375446000	0.795526000
C	-3.847775000	-0.088591000	-0.576526000
C	-2.378186000	0.393801000	-0.720826000
C	-1.146098000	2.292415000	0.303956000
C	0.096611000	1.483918000	-0.031792000
C	-0.118379000	0.027483000	0.438531000
C	-1.236206000	-0.611516000	-0.420061000
C	-4.392142000	-0.688317000	-1.888993000
C	-5.306548000	-1.820336000	-1.411347000
C	-4.090366000	-1.239004000	0.487542000
C	-2.815247000	-1.617387000	1.232473000
C	-1.727038000	-1.935305000	0.199322000
C	-0.067764000	-3.212717000	-2.085974000
C	-1.524819000	-3.457956000	-1.816487000
C	-2.257258000	-2.937211000	-0.822610000
C	-3.661290000	-3.426695000	-0.543012000
C	1.384161000	2.179248000	0.467043000
C	2.536452000	1.182053000	0.569236000

C	2.282852000	-0.068353000	-0.274507000
C	1.599952000	-1.595821000	1.303054000
C	3.426655000	-1.110951000	-0.152954000
C	2.961000000	-1.876355000	1.068356000
C	0.946855000	-2.132146000	2.413142000
C	1.667638000	-2.984598000	3.260284000
C	3.003353000	-3.291862000	3.015731000
C	3.658013000	-2.726577000	1.910504000
C	3.932410000	1.793764000	0.374490000
C	4.942665000	0.751869000	0.857546000
C	4.835978000	-0.463758000	-0.040690000
C	4.876499000	-1.398747000	-2.122454000
C	3.583407000	-1.955206000	-1.446329000
C	4.550685000	0.999148000	-2.008342000
C	4.273808000	2.153424000	-1.056628000
C	4.329607000	3.411393000	-1.517748000
C	3.988181000	4.674973000	-0.782830000
H	-3.266066000	4.102678000	1.119572000
H	-2.504226000	-0.778617000	1.875035000
H	-4.848770000	-0.874642000	1.197279000
H	-6.287811000	-1.418019000	-1.108857000
H	-4.900901000	0.065652000	-2.505460000
H	-3.604554000	-4.102898000	0.327970000
H	-6.637135000	0.731766000	-0.399716000
H	-7.380034000	2.941152000	0.476877000
H	-5.708656000	4.606051000	1.241305000
H	0.154817000	1.458478000	-1.135465000
H	-0.411148000	0.048975000	1.503374000
H	0.425923000	-2.667076000	-1.269788000
H	0.455130000	-4.171501000	-2.224754000

H	-2.268321000	0.736488000	-1.763350000
H	-0.861237000	-2.403215000	0.688870000
H	-4.033812000	-4.034038000	-1.379263000
H	-2.021999000	-4.171188000	-2.484332000
H	-3.560308000	-1.111306000	-2.473530000
H	-5.487089000	-2.592565000	-2.170406000
H	-3.012913000	-2.472749000	1.893867000
H	-0.754234000	-0.835262000	-1.380940000
H	0.077585000	-2.639888000	-3.017918000
H	1.632839000	2.961464000	-0.275501000
H	0.521680000	3.380807000	1.687414000
H	3.548390000	4.494400000	0.206253000
H	4.881045000	5.307564000	-0.655040000
H	3.266422000	5.267193000	-1.366493000
H	4.649820000	3.549161000	-2.557546000
H	5.164991000	1.368022000	-2.843563000
H	3.587510000	0.689202000	-2.461457000
H	4.728775000	0.469040000	1.898904000
H	5.968617000	1.143496000	0.807950000
H	3.991907000	2.688153000	1.007150000
H	2.502269000	0.838406000	1.618013000
H	2.078369000	0.183079000	-1.322851000
H	-0.095256000	-1.897866000	2.633580000
H	1.165748000	-3.408764000	4.131098000
H	3.543207000	-3.959238000	3.687375000
H	4.711103000	-2.948850000	1.724249000
H	3.698170000	-3.020788000	-1.205336000
H	5.696436000	-2.122063000	-2.003864000
H	2.695617000	-1.857957000	-2.087854000
H	4.756114000	-1.206148000	-3.196849000

H	5.522301000	-1.250128000	0.317854000

Equilibrium geometries of molecules from set **1** calculated at the **DFT(M06-2X)** level of theory with **cc-pVTZ** basis set within the IEF-PCM model. Cartesian coordinates are given for standard orientation in form (atomic label, X, Y, Z). The X, Y, Z are given in Å.

12-28-oxaircinal (*E* = -1272.2607587 Hartree), solvent: Chloroform

O	1.591998000	-0.779056000	1.829797000
O	-0.171389000	4.733829000	1.495782000
N	1.765784000	-0.504352000	-0.454900000
N	-2.182251000	0.481449000	-1.535381000
C	0.068078000	1.165624000	-0.860141000
C	0.391049000	-0.135282000	-0.096577000
C	0.395618000	-0.093362000	1.457736000
C	-0.581489000	2.284976000	-0.012821000
C	1.454248000	1.610710000	-1.384973000
C	2.489475000	0.760934000	-0.638437000
C	-0.856372000	0.804217000	-2.025932000
C	2.180954000	-1.348577000	0.646990000
C	-2.105956000	2.170897000	0.183304000
C	0.077231000	2.382137000	1.332329000
C	0.500469000	1.303294000	1.993193000
C	-0.848613000	-0.799016000	2.021644000
C	-2.824335000	1.699340000	-1.068949000
C	3.669131000	-1.497682000	0.867366000
C	3.788471000	0.685025000	-1.385826000
C	-2.971784000	-0.312756000	-2.461590000
C	-0.840773000	-2.321040000	1.818155000
C	4.365137000	-2.322222000	-0.212545000
C	0.206550000	3.694401000	1.983400000
C	4.463106000	-0.372626000	-1.825733000

C	4.112666000	-1.826062000	-1.645120000
C	-4.285746000	-0.809974000	-1.852637000
C	-2.200319000	-2.916905000	2.067987000
C	-4.170192000	-1.324774000	-0.418098000
C	-3.163751000	-2.464597000	-0.233020000
C	-3.185878000	-2.981664000	1.176478000
H	-0.293211000	-0.921850000	-0.410555000
H	-0.408449000	3.221996000	-0.547840000
H	1.634560000	2.676243000	-1.247856000
H	1.529470000	1.393372000	-2.452691000
H	2.691164000	1.238470000	0.333286000
H	-0.886528000	1.636206000	-2.750541000
H	-0.436884000	-0.062222000	-2.542451000
H	1.732513000	-2.336817000	0.486728000
H	-2.482610000	3.143726000	0.503258000
H	-2.324908000	1.464994000	0.983808000
H	0.946309000	1.400294000	2.979194000
H	-1.719304000	-0.387507000	1.507783000
H	-0.961510000	-0.572177000	3.084074000
H	-3.868158000	1.501768000	-0.831917000
H	-2.806749000	2.477602000	-1.850446000
H	3.797727000	-1.985890000	1.833880000
H	4.119245000	-0.508130000	0.952020000
H	4.213689000	1.666365000	-1.578460000
H	-2.357678000	-1.171713000	-2.741444000
H	-3.190688000	0.236383000	-3.391642000
H	-0.102874000	-2.767593000	2.483718000
H	-0.526459000	-2.557771000	0.802121000
H	4.036627000	-3.361802000	-0.143354000
H	5.437605000	-2.317322000	-0.009629000

H	0.685945000	3.678404000	2.978415000
H	5.395604000	-0.169620000	-2.342140000
H	3.068678000	-1.993008000	-1.899567000
H	4.713314000	-2.425139000	-2.329213000
H	-4.666991000	-1.605371000	-2.497572000
H	-5.034479000	-0.016353000	-1.881726000
H	-2.389280000	-3.294838000	3.067161000
H	-5.155490000	-1.663329000	-0.088644000
H	-3.884338000	-0.506075000	0.246838000
H	-2.169842000	-2.107673000	-0.503260000
H	-3.417371000	-3.275212000	-0.924066000
H	-4.130090000	-3.416369000	1.492951000

Anabsinthin (Conformer 1, *E* = -1618.6140739 Hartree), solvent: Acetonitrile

O	-2.325517000	1.148984000	-2.244626000
O	-2.552687000	-1.947427000	0.637366000
O	2.390064000	0.245306000	3.118700000
O	3.066012000	-1.654453000	-1.204136000
O	-3.742002000	-3.381638000	1.851867000
O	4.546567000	-2.172442000	-2.784450000
C	-1.641909000	0.162071000	-0.257362000
C	-1.970932000	1.663002000	-0.008850000
C	-0.494697000	2.119788000	-0.093622000
C	-1.162167000	0.486934000	-1.706486000
C	-0.523242000	-0.116877000	0.778863000
C	0.146738000	1.261797000	1.005160000
C	-2.801130000	2.074133000	-1.237301000
C	-0.085390000	1.559063000	-1.473774000

C	-2.807792000	-0.827798000	-0.249306000
C	1.677065000	1.060113000	0.905035000
C	0.615757000	-1.022828000	0.401456000
C	-4.170250000	-0.308962000	0.199548000
C	-4.316207000	1.911338000	-1.048300000
C	1.784159000	-0.377405000	0.429362000
C	-0.751560000	-0.607603000	-2.656346000
C	-4.863369000	0.498936000	-0.881542000
C	2.480292000	1.344833000	2.213316000
C	-2.565464000	3.517169000	-1.679647000
C	-4.866407000	-1.609875000	0.592883000
C	3.095343000	-1.018093000	0.107092000
C	0.400171000	-2.475658000	0.129726000
C	3.979216000	1.548505000	1.950095000
C	-3.710317000	-2.433240000	1.116187000
C	4.297842000	-0.091161000	0.025143000
C	4.780512000	0.380452000	1.383968000
C	1.973091000	2.619499000	2.892955000
C	-6.014881000	-1.501119000	1.576914000
C	5.262456000	-0.951703000	-0.787105000
C	4.308162000	-1.665847000	-1.722712000
C	6.378610000	-0.230084000	-1.515992000
H	-2.439457000	1.898814000	0.945239000
H	-0.321464000	3.186442000	0.032884000
H	-1.003970000	-0.493253000	1.684176000
H	-0.149122000	1.665309000	1.969576000
H	0.913207000	1.128550000	-1.477348000
H	-0.131851000	2.291032000	-2.276750000
H	-2.927056000	-1.254529000	-1.247937000
H	2.081656000	1.757441000	0.163479000

H	-4.043542000	0.294147000	1.105272000
H	-4.810844000	2.368985000	-1.907495000
H	-4.592408000	2.511696000	-0.177450000
H	-1.521438000	-1.369982000	-2.770795000
H	-0.564417000	-0.169119000	-3.637326000
H	0.169281000	-1.077373000	-2.313672000
H	-4.783283000	-0.045812000	-1.825416000
H	-5.927823000	0.574066000	-0.651131000
H	-3.081648000	3.694330000	-2.622985000
H	-2.975497000	4.200828000	-0.934637000
H	-1.514638000	3.753298000	-1.815191000
H	-5.201345000	-2.115801000	-0.321367000
H	3.309744000	-1.812626000	0.830685000
H	-0.296176000	-2.625086000	-0.697517000
H	1.328117000	-2.991969000	-0.102052000
H	-0.063987000	-2.940448000	1.001432000
H	4.428447000	1.832381000	2.903251000
H	4.081692000	2.410733000	1.285991000
H	4.027295000	0.773532000	-0.592134000
H	4.767549000	-0.459142000	2.083316000
H	5.820064000	0.703755000	1.300549000
H	1.005753000	2.465078000	3.366477000
H	1.875721000	3.432606000	2.171439000
H	2.674008000	2.925760000	3.668391000
H	-5.686028000	-1.013217000	2.494721000
H	-6.396763000	-2.488094000	1.831517000
H	-6.829002000	-0.917561000	1.149621000
H	1.460461000	0.051256000	3.280087000
H	5.678705000	-1.725915000	-0.130636000
H	6.958157000	-0.928573000	-2.116815000

H	7.049686000	0.251008000	-0.805973000
H	5.970530000	0.534541000	-2.177394000

Anabsinthin (Conformer 2, *E* = -1618.6140775 Hartree), solvent: Acetonitrile

O	-2.186210000	2.315819000	-1.060309000
O	-2.599149000	-1.880236000	-0.696389000
O	1.521176000	-1.671824000	3.044042000
O	3.055685000	-0.499728000	-1.991636000
O	-3.866454000	-3.706354000	-0.627193000
O	4.488997000	0.310195000	-3.491177000
C	-1.594618000	0.289231000	-0.095307000
C	-1.877474000	1.336913000	1.020318000
C	-0.383863000	1.683978000	1.234074000
C	-1.060034000	1.413560000	-1.040537000
C	-0.520380000	-0.619529000	0.555685000
C	0.190079000	0.306752000	1.581243000
C	-2.659116000	2.449300000	0.301619000
C	0.043950000	2.072638000	-0.197284000
C	-2.793369000	-0.442435000	-0.700816000
C	1.701434000	0.107126000	1.373437000
C	0.591043000	-1.138322000	-0.323643000
C	-4.148312000	-0.252958000	-0.026075000
C	-4.183496000	2.270759000	0.350199000
C	1.776893000	-0.678627000	0.082745000
C	-0.661244000	1.121316000	-2.463769000
C	-4.784970000	1.077445000	-0.382351000
C	2.381557000	-0.639354000	2.554359000
C	-2.363788000	3.848924000	0.837964000
C	-4.898371000	-1.490452000	-0.513595000
C	3.096809000	-0.900227000	-0.594125000

C	0.336695000	-2.069111000	-1.463025000
C	3.705392000	-1.317336000	2.159788000
C	-3.784086000	-2.508472000	-0.617170000
C	4.226322000	-0.051318000	-0.019002000
C	4.732600000	-0.521890000	1.346782000
C	2.578858000	0.318924000	3.716068000
C	-6.059888000	-1.964683000	0.338205000
C	5.213705000	-0.056551000	-1.180575000
C	4.268611000	-0.045001000	-2.366816000
C	6.234235000	1.063721000	-1.217176000
H	-2.362820000	0.959280000	1.918753000
H	-0.179205000	2.439583000	1.989730000
H	-1.036488000	-1.451092000	1.039324000
H	-0.096593000	0.045754000	2.597517000
H	1.030572000	1.697880000	-0.460569000
H	0.038645000	3.144366000	-0.382276000
H	-2.900824000	-0.155390000	-1.749562000
H	2.203472000	1.074245000	1.287184000
H	-4.023580000	-0.338897000	1.058903000
H	-4.639188000	3.179550000	-0.048321000
H	-4.461838000	2.224013000	1.406336000
H	-0.459456000	2.066242000	-2.969802000
H	0.248952000	0.523864000	-2.487153000
H	-1.443894000	0.604669000	-3.018510000
H	-4.702075000	1.222081000	-1.462302000
H	-5.851367000	1.042324000	-0.151186000
H	-2.772649000	3.949655000	1.844536000
H	-1.302424000	4.072874000	0.879071000
H	-2.846467000	4.589183000	0.200163000
H	-5.235114000	-1.312513000	-1.542470000

H	3.374876000	-1.959122000	-0.589389000
H	-0.112682000	-2.990220000	-1.087569000
H	-0.381891000	-1.648170000	-2.167689000
H	1.251843000	-2.308969000	-1.999977000
H	3.449808000	-2.222149000	1.601496000
H	4.175620000	-1.662277000	3.082169000
H	3.843743000	0.969741000	0.059474000
H	5.596278000	-1.173743000	1.196912000
H	5.092506000	0.335697000	1.916893000
H	2.921642000	-0.227728000	4.594148000
H	1.644010000	0.823656000	3.962161000
H	3.321271000	1.075445000	3.462026000
H	-6.482042000	-2.883112000	-0.065572000
H	-6.844253000	-1.209821000	0.367841000
H	-5.729490000	-2.158587000	1.358912000
H	1.336001000	-2.276958000	2.316045000
H	5.720507000	-1.028843000	-1.209259000
H	6.900046000	0.996132000	-0.357793000
H	5.737005000	2.033720000	-1.192528000
H	6.834132000	1.008745000	-2.123693000

Betulinic Acid (*E* = -1397.7051133 Hartree), solvent: Pyridine

C	4.181512000	1.209869000	0.350566000
C	3.152865000	0.294539000	-0.345664000
C	3.794380000	-1.100562000	-0.372389000
C	5.242256000	-0.752652000	-0.759656000
C	5.550235000	0.501376000	0.077265000
C	1.715097000	0.255153000	0.161092000
C	0.839994000	-0.708906000	-0.694710000
C	1.545331000	-2.078040000	-0.872960000

C	3.008057000	-1.988445000	-1.331089000
C	1.067258000	1.633851000	0.251769000
C	-0.368261000	1.572933000	0.783412000
C	-1.248798000	0.525115000	0.086448000
C	-0.551976000	-0.869357000	0.054562000
C	-2.740325000	0.538560000	0.566954000
C	-3.504467000	-0.454500000	-0.354625000
C	-2.909072000	-1.857303000	-0.245841000
C	-1.448247000	-1.873567000	-0.691089000
C	-3.303673000	1.951028000	0.310410000
C	-4.823131000	2.024831000	0.397333000
C	-5.491285000	1.039988000	-0.538740000
C	-5.057265000	-0.419215000	-0.286115000
C	4.205632000	2.642672000	-0.126045000
C	4.245419000	3.653317000	0.736627000
C	4.239958000	2.879402000	-1.610672000
C	3.869053000	-1.745693000	1.003235000
O	3.746909000	-1.196190000	2.069258000
O	4.164653000	-3.055560000	0.927928000
C	0.663952000	-0.145142000	-2.120965000
C	-0.338717000	-1.416280000	1.482710000
C	-2.892699000	0.235668000	2.071313000
C	-5.625943000	-1.276901000	-1.426346000
C	-5.647132000	-0.950430000	1.024208000
O	-6.894638000	1.233409000	-0.396686000
H	3.991878000	1.211374000	1.422776000
H	3.138388000	0.594551000	-1.396925000
H	5.249560000	-0.517967000	-1.826108000
H	5.944860000	-1.568021000	-0.591800000
H	6.252781000	1.160620000	-0.430672000

H	6.004606000	0.222526000	1.027093000
H	1.759263000	-0.148866000	1.173572000
H	0.985412000	-2.672553000	-1.596564000
H	1.533620000	-2.645837000	0.058402000
H	3.083790000	-1.555823000	-2.329958000
H	3.437038000	-2.987949000	-1.384248000
H	1.086605000	2.118378000	-0.728450000
H	1.660629000	2.268763000	0.910688000
H	-0.815967000	2.560086000	0.674645000
H	-0.324566000	1.378664000	1.856333000
H	-1.332861000	0.836613000	-0.957120000
H	-3.275532000	-0.109469000	-1.374042000
H	-3.458906000	-2.552517000	-0.879009000
H	-3.007676000	-2.237908000	0.771854000
H	-1.435474000	-1.661106000	-1.761259000
H	-1.042970000	-2.880239000	-0.564617000
H	-2.991509000	2.280005000	-0.686711000
H	-2.877267000	2.656944000	1.023848000
H	-5.159901000	3.029575000	0.138004000
H	-5.172063000	1.837347000	1.414725000
H	-5.194336000	1.289523000	-1.567199000
H	4.315043000	4.680840000	0.401849000
H	4.204079000	3.480462000	1.805215000
H	3.262525000	2.684405000	-2.057775000
H	4.514715000	3.907439000	-1.837904000
H	4.950894000	2.211095000	-2.102762000
H	4.250577000	-3.388964000	1.832736000
H	0.193539000	0.832062000	-2.155914000
H	1.626992000	-0.042458000	-2.616902000
H	0.073169000	-0.819975000	-2.737977000

H	-1.250263000	-1.871195000	1.860046000
H	0.426050000	-2.189559000	1.506741000
H	-0.043897000	-0.652699000	2.197776000
H	-2.016551000	0.565445000	2.626158000
H	-3.743841000	0.766435000	2.492714000
H	-3.038316000	-0.820014000	2.286345000
H	-5.157790000	-1.028297000	-2.381093000
H	-5.483084000	-2.340200000	-1.240886000
H	-6.702849000	-1.128275000	-1.523974000
H	-6.728002000	-1.048394000	0.924668000
H	-5.242635000	-1.937518000	1.252105000
H	-5.455361000	-0.302763000	1.875223000
H	-7.349818000	0.754294000	-1.093645000

Icajine (Conformer 1, *E* = -1187.9146277 Hartree), solvent: Chloroform

O	-0.153216000	-2.873798000	1.220485000
O	2.098559000	3.007937000	0.722157000
O	-2.507036000	3.216959000	-0.445807000
N	-1.438310000	1.204864000	-0.487267000
N	2.075960000	-2.090484000	-0.567694000
C	-0.149943000	0.516054000	-0.303851000
C	0.199622000	-1.741906000	0.965115000
C	1.349845000	-1.732035000	-1.775866000
C	-0.154212000	-1.747148000	-1.499710000
C	-0.496268000	-0.991903000	-0.195509000
C	-2.004372000	-0.962678000	-0.011667000
C	-2.872860000	-2.001987000	0.263485000
C	-4.240789000	-1.744040000	0.312589000
C	-4.722357000	-0.463328000	0.078297000

C	-3.861566000	0.592384000	-0.206351000
C	-2.501816000	0.319836000	-0.242142000
C	1.199718000	-1.036604000	1.863336000
C	1.747786000	0.284614000	1.315395000
C	0.514065000	1.104442000	0.933879000
C	0.737597000	2.625330000	0.773830000
C	2.868507000	2.639615000	-0.399157000
C	3.173303000	1.181185000	-0.554893000
C	2.734297000	0.146495000	0.155052000
C	3.201546000	-1.242084000	-0.212921000
C	-0.095560000	3.190692000	-0.392644000
C	-1.476608000	2.569090000	-0.443932000
C	2.319057000	-3.508999000	-0.427082000
H	0.472668000	0.697325000	-1.178019000
H	1.587094000	-2.413424000	-2.600491000
H	1.664670000	-0.738153000	-2.092718000
H	-0.510863000	-2.772108000	-1.384698000
H	-0.702294000	-1.301830000	-2.332504000
H	-2.485903000	-2.994328000	0.444279000
H	-4.930322000	-2.547683000	0.530602000
H	-5.786986000	-0.276138000	0.115662000
H	-4.227671000	1.588947000	-0.389057000
H	1.974706000	-1.762718000	2.105107000
H	0.677710000	-0.823219000	2.800354000
H	2.265024000	0.795142000	2.129486000
H	-0.213406000	0.986263000	1.743775000
H	0.389280000	3.105216000	1.686933000
H	3.811106000	3.179105000	-0.286668000
H	2.423653000	3.013993000	-1.328786000
H	3.859806000	0.981227000	-1.373279000

H	3.691077000	-1.704039000	0.648566000
H	3.952980000	-1.182146000	-1.011370000
H	-0.203400000	4.267786000	-0.309933000
H	0.392392000	2.966436000	-1.343160000
H	3.078419000	-3.876945000	-1.129539000
H	1.390528000	-4.053643000	-0.596651000
H	2.645765000	-3.726656000	0.590629000

Icajine (Conformer 2, *E* = -1187.9157106 Hartree), solvent: Chloroform

O	-0.202880000	-2.759511000	1.288980000
O	1.916820000	2.854698000	-0.542302000
O	-2.715661000	3.153132000	-0.027142000
N	-1.521015000	1.250108000	-0.361138000
N	2.063767000	-2.117510000	-0.476575000
C	-0.197634000	0.589703000	-0.338773000
C	0.164330000	-1.643709000	0.988843000
C	1.410733000	-1.690529000	-1.702749000
C	-0.099630000	-1.698481000	-1.495119000
C	-0.501790000	-0.933715000	-0.214582000
C	-2.011757000	-0.968252000	-0.077836000
C	-2.841727000	-2.062158000	0.084233000
C	-4.219090000	-1.865050000	0.123355000
C	-4.746787000	-0.587690000	-0.008165000
C	-3.924239000	0.520954000	-0.178770000
C	-2.552284000	0.307049000	-0.207822000
C	1.136208000	-0.897285000	1.882800000
C	1.774033000	0.351107000	1.265368000
C	0.602471000	1.224947000	0.791689000
C	0.900258000	2.683543000	0.427098000
C	3.225626000	2.513222000	-0.116870000

C	3.530790000	1.062698000	-0.350064000
C	2.864960000	0.072288000	0.234267000
C	3.229388000	-1.356689000	-0.074171000
C	-0.341126000	3.366290000	-0.173526000
C	-1.641414000	2.592913000	-0.161862000
C	2.214487000	-3.550301000	-0.354722000
H	0.313401000	0.788382000	-1.283574000
H	1.750041000	-0.683536000	-1.947136000
H	1.682439000	-2.331451000	-2.549781000
H	-0.462562000	-2.722446000	-1.391313000
H	-0.611381000	-1.257267000	-2.352530000
H	-2.418529000	-3.050398000	0.189634000
H	-4.879196000	-2.711377000	0.253291000
H	-5.818637000	-0.445070000	0.019647000
H	-4.328302000	1.513412000	-0.281580000
H	0.567156000	-0.590414000	2.765304000
H	1.869232000	-1.624626000	2.228680000
H	2.263180000	0.898275000	2.079054000
H	-0.076085000	1.285382000	1.650926000
H	1.204205000	3.204772000	1.341473000
H	3.348387000	2.773928000	0.942151000
H	3.904380000	3.139629000	-0.691547000
H	4.315501000	0.824576000	-1.059001000
H	3.626964000	-1.832054000	0.827184000
H	4.032234000	-1.377049000	-0.823869000
H	-0.541322000	4.326514000	0.292544000
H	-0.111265000	3.560700000	-1.223915000
H	2.972157000	-3.951067000	-1.041011000
H	1.261754000	-4.036432000	-0.561029000
H	2.495899000	-3.802264000	0.668370000

Iguesterin (*E* = -1239.0042232 Hartree), solvent: Chloroform

O	-6.512213000	0.826655000	-0.982623000
O	-5.248717000	2.649135000	0.407347000
C	1.660071000	0.208404000	0.442025000
C	1.049428000	-1.235411000	0.551771000
C	3.202079000	0.145671000	0.639823000
C	3.964627000	-0.890917000	-0.236409000
C	1.731942000	-2.148747000	-0.482046000
C	1.014764000	1.052403000	1.554272000
C	3.240820000	-2.242360000	-0.271028000
C	-0.454855000	-1.127464000	0.309550000
C	-1.276094000	-0.086555000	1.064189000
C	-0.492310000	1.211819000	1.367592000
C	3.895844000	1.516535000	0.536203000
C	1.290485000	0.856476000	-0.903705000
C	4.184307000	-0.420173000	-1.686759000
C	1.284655000	-1.889218000	1.934321000
C	5.350968000	-1.109126000	0.384909000
C	4.374207000	1.913268000	-0.834875000
C	-2.604180000	0.240760000	0.389707000
C	4.504810000	1.034554000	-1.821513000
C	-1.097173000	-1.962444000	-0.526231000
C	-1.729961000	-0.733320000	2.414311000
C	-3.243177000	-0.769343000	-0.439983000
C	-2.494767000	-1.831588000	-0.830598000
C	4.713092000	3.365199000	-0.996780000
C	-3.264245000	1.386566000	0.669260000
C	-4.612315000	-0.595146000	-0.899147000
C	-4.608531000	1.618484000	0.190966000

C	-5.248602000	0.551057000	-0.591971000
C	-5.282651000	-1.655872000	-1.721027000
H	3.338852000	-0.184040000	1.673806000
H	1.319911000	-3.156497000	-0.409455000
H	1.510312000	-1.794319000	-1.490539000
H	1.249297000	0.630139000	2.531295000
H	1.433965000	2.057625000	1.556119000
H	3.676598000	-2.842902000	-1.074482000
H	3.450729000	-2.790038000	0.648709000
H	-0.663987000	1.914418000	0.552805000
H	-0.913840000	1.674474000	2.260800000
H	3.246666000	2.308470000	0.911886000
H	4.762378000	1.532382000	1.206246000
H	0.218767000	0.807606000	-1.095420000
H	1.576249000	1.909879000	-0.897184000
H	1.785794000	0.391642000	-1.748054000
H	5.009348000	-1.009262000	-2.100974000
H	3.325665000	-0.670665000	-2.316049000
H	2.342325000	-2.043080000	2.127777000
H	0.897590000	-1.314066000	2.764608000
H	0.796940000	-2.865174000	1.949068000
H	5.269318000	-1.355572000	1.445491000
H	5.979438000	-0.223849000	0.282739000
H	5.857047000	-1.936690000	-0.115980000
H	4.860119000	1.375368000	-2.789037000
H	-0.567381000	-2.753581000	-1.033501000
H	-0.892024000	-0.877774000	3.087113000
H	-2.212044000	-1.694526000	2.238587000
H	-2.442128000	-0.063650000	2.894594000
H	-2.938215000	-2.589284000	-1.463495000

H	5.446168000	3.676170000	-0.248181000
H	3.825768000	3.985012000	-0.843325000
H	5.116581000	3.576746000	-1.985541000
H	-2.839573000	2.172439000	1.277127000
H	-5.270294000	-2.617407000	-1.206354000
H	-4.776605000	-1.789349000	-2.678714000
H	-6.315234000	-1.383285000	-1.917674000
H	-6.696494000	1.715245000	-0.633569000

Itoaic Acid (Conformer 1, *E* = -1623.406396 Hartree), solvent: Chloroform

C	-3.416553000	-1.105282000	-0.271470000
C	-2.564134000	0.164219000	0.055322000
C	-1.258749000	-0.105869000	0.898212000
C	-0.421323000	-1.133110000	0.059994000
C	-1.215585000	-2.424240000	-0.123919000
C	-2.495723000	-2.160894000	-0.902454000
C	-0.436235000	1.211216000	1.000958000
C	1.053876000	1.014693000	1.296363000
C	1.778920000	0.048536000	0.346573000
C	1.063919000	-1.341585000	0.477285000
C	3.294849000	-0.065941000	0.750416000
C	4.090379000	-1.285663000	0.185216000
C	3.236463000	-2.555177000	-0.049417000
C	1.768759000	-2.366211000	-0.430657000
C	4.129896000	1.247533000	0.658108000
C	5.088619000	1.520446000	-0.532801000
C	5.780934000	0.231321000	-0.985354000
C	4.808781000	-0.930099000	-1.129675000
C	-3.387058000	1.338892000	0.597948000
C	-4.460010000	1.913342000	-0.336049000

C	-5.718203000	1.071845000	-0.295720000
O	-5.670800000	-0.170926000	-0.748045000
C	-4.479913000	-0.740740000	-1.345318000
O	-4.786551000	3.220324000	0.063209000
O	-6.743502000	1.502316000	0.173635000
C	-5.026533000	-1.901466000	-2.159420000
C	-4.142226000	-1.712874000	0.936068000
C	-1.601067000	-0.569180000	2.329080000
O	-0.941805000	2.132082000	1.962742000
C	1.165995000	-1.907945000	1.906863000
C	5.147812000	-1.660577000	1.238290000
C	6.144650000	2.512917000	-0.036344000
C	4.411594000	2.164475000	-1.748106000
C	1.687927000	0.611219000	-1.073601000
O	1.513845000	1.944565000	-1.087451000
O	1.791384000	0.008648000	-2.112952000
H	-2.184180000	0.497463000	-0.920111000
H	-0.365938000	-0.697290000	-0.945216000
H	-1.436247000	-2.898611000	0.833719000
H	-0.635131000	-3.146004000	-0.693536000
H	-3.046954000	-3.094319000	-1.022972000
H	-2.212156000	-1.827443000	-1.906624000
H	-0.533504000	1.734927000	0.049306000
H	1.169796000	0.646575000	2.317235000
H	1.501550000	2.004396000	1.278180000
H	3.212178000	-0.245354000	1.824694000
H	3.276772000	-3.173730000	0.847120000
H	3.725473000	-3.148245000	-0.825315000
H	1.289908000	-3.337773000	-0.311590000
H	1.664689000	-2.080953000	-1.472914000

H	4.759202000	1.252927000	1.550162000
H	3.475094000	2.113636000	0.767103000
H	6.565770000	-0.035086000	-0.274381000
H	6.285483000	0.418814000	-1.936896000
H	5.356313000	-1.816775000	-1.458165000
H	4.068928000	-0.718811000	-1.903533000
H	-2.727111000	2.177569000	0.787324000
H	-3.856487000	1.108339000	1.556999000
H	-4.078307000	1.947306000	-1.360896000
H	-4.053470000	-0.011221000	-2.036920000
H	-5.703736000	3.194071000	0.369713000
H	-4.265796000	-2.318055000	-2.813205000
H	-5.411411000	-2.691663000	-1.516081000
H	-5.845439000	-1.536028000	-2.775657000
H	-4.637358000	-0.961414000	1.549370000
H	-4.910768000	-2.407211000	0.595957000
H	-3.460970000	-2.270135000	1.571903000
H	-2.560739000	-0.170456000	2.650714000
H	-0.864896000	-0.225948000	3.055106000
H	-1.642208000	-1.649695000	2.427489000
H	-0.770390000	1.789519000	2.844444000
H	0.633926000	-2.860368000	1.941488000
H	0.744833000	-1.273485000	2.673900000
H	2.194605000	-2.109761000	2.191793000
H	5.782541000	-0.821498000	1.518259000
H	4.662996000	-2.023824000	2.146119000
H	5.790914000	-2.457797000	0.858590000
H	6.858709000	2.739912000	-0.830213000
H	5.680489000	3.450054000	0.279076000
H	6.697528000	2.104747000	0.811492000

H	5.181052000	2.478175000	-2.456141000
H	3.753831000	1.475740000	-2.275107000
H	3.841824000	3.047281000	-1.456780000
H	1.547851000	2.228607000	-2.012470000

Itoaic Acid (Conformer 2, *E* = -1623.4065431 Hartree), solvent: Chloroform

C	-3.485167000	-1.102792000	0.154318000
C	-2.634224000	0.203529000	0.011922000
C	-1.240278000	0.175324000	0.750400000
C	-0.488694000	-1.058266000	0.144718000
C	-1.260470000	-2.338696000	0.455239000
C	-2.615176000	-2.310937000	-0.234526000
C	-0.421496000	1.447234000	0.389150000
C	1.080172000	1.316757000	0.653753000
C	1.739153000	0.134956000	-0.073352000
C	1.040065000	-1.177389000	0.400002000
C	3.274523000	0.063855000	0.256279000
C	3.972507000	-1.277504000	-0.197422000
C	3.017548000	-2.177494000	-0.991239000
C	1.624233000	-2.391478000	-0.379879000
C	4.058044000	1.298410000	-0.216260000
C	5.557946000	1.273852000	0.116698000
C	6.187989000	-0.049532000	-0.399558000
C	5.169359000	-0.939777000	-1.103175000
C	-3.426965000	1.488143000	0.282785000
C	-4.605705000	1.777926000	-0.655027000
C	-5.831586000	0.989668000	-0.245258000
O	-5.799742000	-0.330536000	-0.329485000
C	-4.662192000	-1.054734000	-0.860703000
O	-4.921475000	3.146474000	-0.609971000

O	-6.813660000	1.541129000	0.188803000
C	-5.262359000	-2.396650000	-1.245498000
C	-4.069192000	-1.324913000	1.555830000
C	-1.408977000	0.151130000	2.283217000
O	-0.865453000	2.629441000	1.046110000
C	1.292084000	-1.430762000	1.898618000
C	4.515603000	-2.090106000	0.996217000
C	5.783401000	1.433832000	1.621456000
C	6.209996000	2.462177000	-0.593094000
C	1.498711000	0.387629000	-1.573019000
O	1.724664000	1.668680000	-1.920858000
O	1.113501000	-0.399146000	-2.399463000
H	-2.366632000	0.243123000	-1.052604000
H	-0.574799000	-0.931595000	-0.936998000
H	-1.385460000	-2.483580000	1.528455000
H	-0.710279000	-3.208204000	0.097347000
H	-3.160675000	-3.229809000	-0.015736000
H	-2.442424000	-2.291422000	-1.315874000
H	-0.572917000	1.652641000	-0.675401000
H	1.255114000	1.214353000	1.726146000
H	1.528259000	2.259495000	0.354031000
H	3.295549000	0.090950000	1.347389000
H	3.500561000	-3.150314000	-1.115811000
H	2.904783000	-1.770529000	-1.993290000
H	1.648191000	-3.248014000	0.296610000
H	0.953329000	-2.660875000	-1.194624000
H	3.625882000	2.200470000	0.220861000
H	3.961475000	1.413025000	-1.294474000
H	6.628545000	-0.600309000	0.434128000
H	7.009999000	0.174038000	-1.081796000

H	5.640024000	-1.873910000	-1.418939000
H	4.814101000	-0.452918000	-2.015225000
H	-2.771752000	2.341158000	0.150973000
H	-3.786764000	1.545334000	1.312655000
H	-4.333089000	1.516720000	-1.681922000
H	-4.327161000	-0.557776000	-1.773459000
H	-5.802386000	3.213523000	-0.216016000
H	-4.563836000	-2.989302000	-1.828936000
H	-5.560130000	-2.965232000	-0.365438000
H	-6.148841000	-2.217794000	-1.850515000
H	-4.507631000	-0.420650000	1.975290000
H	-4.858412000	-2.075855000	1.514773000
H	-3.315647000	-1.684817000	2.249753000
H	-2.319630000	0.664860000	2.585169000
H	-0.584967000	0.653181000	2.790065000
H	-1.446650000	-0.853296000	2.692739000
H	-0.637809000	2.569918000	1.978511000
H	0.727269000	-2.311593000	2.204409000
H	0.988905000	-0.611881000	2.540668000
H	2.334305000	-1.637320000	2.109328000
H	4.913928000	-1.461287000	1.792008000
H	3.755078000	-2.736707000	1.428714000
H	5.322368000	-2.741697000	0.654609000
H	5.323811000	0.628382000	2.194385000
H	6.851626000	1.430504000	1.846270000
H	5.366411000	2.379179000	1.975490000
H	6.098342000	2.373452000	-1.675600000
H	5.753941000	3.403742000	-0.279988000
H	7.276722000	2.507653000	-0.365727000
H	1.543553000	1.742299000	-2.869043000

Matopensine (E = -1767.4149876 Hartree), solvent: Chloroform

O	-0.000019000	-0.000002000	2.585437000
N	-1.327452000	-0.896851000	0.935433000
N	-3.841434000	2.155269000	-1.041332000
C	-2.078421000	0.335432000	1.223669000
C	-3.335788000	0.210300000	0.329554000
C	-1.140815000	1.534114000	1.039413000
C	-0.189669000	-1.158708000	1.792057000
C	-3.119324000	0.876379000	-1.069518000
C	-0.984155000	1.985327000	-0.446176000
C	-1.651268000	1.003944000	-1.426223000
C	-4.586636000	0.931356000	0.873520000
C	-3.456240000	-1.293687000	0.230360000
C	-2.246911000	-1.883187000	0.616568000
C	-5.057692000	1.807042000	-0.304427000
C	-1.681694000	3.301661000	-0.705104000
C	-3.144249000	3.265553000	-0.362463000
C	-4.512257000	-2.081573000	-0.169142000
C	-2.090303000	-3.260040000	0.641602000
C	-1.151602000	4.368141000	-1.300299000
C	-4.368598000	-3.473068000	-0.160603000
C	-3.173928000	-4.045969000	0.246432000
C	0.241685000	4.552050000	-1.815816000
H	-2.411480000	0.311826000	2.268613000
H	-1.562338000	2.363910000	1.606340000
H	-0.435869000	-1.952302000	2.503302000
H	-3.629302000	0.253110000	-1.808422000
H	0.070001000	2.063119000	-0.691614000
H	-1.545748000	1.396510000	-2.438267000

H	-1.185714000	0.019260000	-1.393445000
H	-4.323026000	1.547278000	1.734762000
H	-5.350495000	0.229801000	1.203641000
H	-5.711307000	1.230595000	-0.962500000
H	-5.593058000	2.703192000	0.005736000
H	-3.625317000	4.195249000	-0.666468000
H	-3.261740000	3.193021000	0.728065000
H	-5.450656000	-1.631094000	-0.471672000
H	-1.153121000	-3.716267000	0.937723000
H	-1.815492000	5.211484000	-1.470820000
H	-5.193327000	-4.102603000	-0.463737000
H	-3.073949000	-5.123291000	0.256998000
H	0.205338000	4.876079000	-2.857475000
H	0.768043000	5.332799000	-1.262090000
H	0.841053000	3.647249000	-1.762962000
N	1.327444000	0.896849000	0.935458000
N	3.841440000	-2.155265000	-1.041289000
C	2.078404000	-0.335437000	1.223705000
C	3.335786000	-0.210307000	0.329609000
C	1.140799000	-1.534116000	1.039430000
C	0.189643000	1.158705000	1.792060000
C	3.119335000	-0.876372000	-1.069471000
C	0.984156000	-1.985317000	-0.446163000
C	1.651284000	-1.003928000	-1.426193000
C	4.586621000	-0.931377000	0.873585000
C	3.456248000	1.293680000	0.230433000
C	2.246915000	1.883183000	0.616623000
C	5.057690000	-1.807051000	-0.304365000
C	1.681691000	-3.301652000	-0.705092000
C	3.144244000	-3.265554000	-0.362438000

C	4.512278000	2.081564000	-0.169039000
C	2.090316000	3.260037000	0.641671000
C	1.151595000	-4.368127000	-1.300292000
C	4.368629000	3.473060000	-0.160485000
C	3.173955000	4.045963000	0.246534000
C	-0.241693000	-4.552027000	-1.815810000
H	2.411447000	-0.311835000	2.268655000
H	1.562311000	-2.363917000	1.606358000
H	0.435831000	1.952298000	2.503311000
H	3.629325000	-0.253098000	-1.808363000
H	-0.069998000	-2.063101000	-0.691612000
H	1.545773000	-1.396483000	-2.438243000
H	1.185733000	-0.019242000	-1.393412000
H	4.322995000	-1.547307000	1.734817000
H	5.350479000	-0.229830000	1.203726000
H	5.711315000	-1.230599000	-0.962424000
H	5.593049000	-2.703206000	0.005794000
H	3.625310000	-4.195249000	-0.666449000
H	3.261725000	-3.193033000	0.728091000
H	5.450680000	1.631082000	-0.471556000
H	1.153132000	3.716267000	0.937781000
H	1.815480000	-5.211474000	-1.470814000
H	5.193368000	4.102593000	-0.463596000
H	3.073983000	5.123286000	0.257112000
H	-0.205349000	-4.876041000	-2.857473000
H	-0.768052000	-5.332783000	-1.262094000
H	-0.841060000	-3.647226000	-1.762941000

Naucleidinal (*E* = -1109.3269968 Hartree), solvent: Chloroform

O	-3.461672000	1.452919000	1.354870000

O	-1.246228000	-1.998063000	1.061177000
O	-6.775539000	-0.010677000	-1.577507000
N	-3.002257000	-3.195749000	0.285600000
N	-5.073318000	-2.957589000	-2.747019000
C	-4.338053000	-3.263193000	-0.312812000
C	-4.558053000	-0.801101000	-0.096254000
C	-5.237146000	-2.136234000	0.161472000
C	-5.391634000	0.393579000	0.338105000
C	-4.136376000	-3.306536000	-1.801597000
C	-3.251892000	-0.803473000	0.645625000
C	-2.133906000	-4.290433000	-0.145205000
C	-2.979421000	-3.704729000	-2.401783000
C	-2.412525000	-2.026286000	0.686444000
C	-1.767890000	-4.129807000	-1.628304000
C	-4.501855000	1.646535000	0.388689000
C	-3.191150000	-3.583684000	-3.815794000
C	-2.832489000	0.265948000	1.325353000
C	-4.509051000	-3.102650000	-3.993920000
C	-6.585187000	0.604847000	-0.559816000
C	-5.216979000	2.919314000	0.779389000
C	-2.398958000	-3.828733000	-4.942899000
C	-5.042100000	-2.853506000	-5.256243000
C	-2.925487000	-3.585224000	-6.195895000
C	-4.234357000	-3.099997000	-6.349826000
H	-4.778585000	-4.209794000	0.013997000
H	-4.379259000	-0.712805000	-1.172462000
H	-6.199026000	-2.201486000	-0.346203000
H	-5.418731000	-2.255739000	1.231652000
H	-5.781627000	0.246732000	1.353704000
H	-2.682785000	-5.220796000	0.005675000

H	-1.249472000	-4.298950000	0.481961000
H	-1.368667000	-5.073499000	-2.004680000
H	-0.973103000	-3.386054000	-1.730483000
H	-4.018179000	1.761238000	-0.586761000
H	-5.944925000	-2.489414000	-2.558238000
H	-1.935033000	0.249715000	1.927488000
H	-7.310245000	1.367546000	-0.231120000
H	-5.901414000	3.237733000	-0.004959000
H	-5.778316000	2.775543000	1.702709000
H	-4.486417000	3.710534000	0.935016000
H	-1.388236000	-4.200111000	-4.831371000
H	-6.050720000	-2.480931000	-5.374836000
H	-2.323959000	-3.768407000	-7.075765000
H	-4.617976000	-2.917035000	-7.344272000

Physalin D (*E* = -1913.7223666 Hartree), solvent: Dimethylsulfoxide

C	-6.157708000	0.473881000	-0.583927000
C	-5.533637000	-0.872318000	-0.394225000
C	-4.016219000	-0.825393000	-0.551465000
C	-3.391078000	0.343655000	0.266731000
C	-3.985152000	1.604270000	-0.369604000
C	-5.453882000	1.605482000	-0.570261000
C	-3.386371000	-2.173826000	-0.217581000
C	-1.876463000	-2.125816000	-0.362346000
C	-1.246253000	-1.022967000	0.495695000
C	-1.846142000	0.353544000	0.127389000
O	-3.320884000	2.560755000	-0.711374000
C	0.299770000	-1.057975000	0.380624000
C	1.107471000	-0.771631000	1.663468000
C	2.358394000	-0.034461000	1.222975000

C	1.771264000	0.777500000	0.064185000
C	1.209296000	2.135421000	0.580423000
C	-0.229430000	2.405893000	0.162822000
C	-1.226183000	1.517388000	0.929221000
O	0.799959000	-1.112483000	2.769513000
O	0.780294000	-0.070958000	-0.519537000
O	0.668225000	-2.328054000	-0.083658000
C	3.528882000	-0.971713000	0.846532000
C	3.153143000	-2.136881000	-0.094896000
C	1.903375000	-2.924834000	0.289157000
C	4.564628000	-0.105752000	0.134193000
C	4.009575000	0.363487000	-1.189291000
C	2.738754000	1.215845000	-1.069380000
C	2.224467000	3.145393000	0.043831000
O	3.177642000	2.533155000	-0.673029000
O	2.246870000	4.315031000	0.291716000
C	3.134320000	-1.854565000	-1.588709000
O	3.728353000	-0.745985000	-2.053204000
C	2.050823000	1.357749000	-2.419471000
O	2.693494000	-2.642552000	-2.380497000
C	4.115616000	-1.534097000	2.141040000
O	-3.689692000	-0.639085000	-1.932725000
O	-3.777881000	-2.542225000	1.096940000
C	-3.832922000	0.333389000	1.746491000
O	1.319232000	2.225490000	1.993457000
H	-7.226020000	0.508460000	-0.761007000
H	-5.934347000	-1.571674000	-1.131938000
H	-5.804791000	-1.274010000	0.583506000
H	-5.909641000	2.568200000	-0.760641000
H	-3.791362000	-2.890228000	-0.939919000

H	-1.626299000	-1.967522000	-1.412335000
H	-1.480188000	-3.099140000	-0.082395000
H	-1.463180000	-1.242135000	1.545576000
H	-1.639351000	0.517108000	-0.930659000
H	2.704012000	0.600912000	2.030257000
H	-0.355779000	2.283252000	-0.911008000
H	-0.421099000	3.457719000	0.385020000
H	-2.013733000	2.174066000	1.278852000
H	-0.757004000	1.134854000	1.838020000
H	3.979452000	-2.852544000	-0.010787000
H	1.915291000	-3.137968000	1.359959000
H	1.909117000	-3.872173000	-0.240338000
H	4.826703000	0.757882000	0.747516000
H	5.477212000	-0.676621000	-0.044559000
H	4.741193000	0.956650000	-1.734387000
H	2.783130000	1.710224000	-3.144968000
H	1.247847000	2.090522000	-2.358767000
H	1.637292000	0.414589000	-2.759008000
H	4.456741000	-0.719396000	2.779747000
H	3.382667000	-2.112749000	2.702900000
H	4.967999000	-2.177065000	1.921772000
H	-4.320361000	-0.029008000	-2.329680000
H	-3.417169000	-3.414079000	1.283662000
H	-3.542454000	1.259112000	2.239133000
H	-4.912015000	0.243421000	1.843116000
H	-3.380551000	-0.498824000	2.276918000
H	1.031772000	3.109442000	2.253901000

Strychnobaillonine (Conformer 1, *E* = -1920.1002471 Hartree), solvent: Chloroform

O	1.188172000	2.831905000	1.743395000

O	-1.248499000	3.446404000	0.830451000
N	-2.375664000	1.613957000	0.111818000
N	-4.158184000	-2.415876000	-1.241398000
N	1.065475000	-0.643040000	0.385693000
N	5.069507000	-0.132793000	-1.541471000
C	-5.470233000	3.167436000	1.502920000
C	-6.372920000	2.221699000	1.033933000
C	-5.922427000	1.141888000	0.280721000
C	-4.572178000	1.025594000	0.008154000
C	-3.681014000	1.977484000	0.486047000
C	-4.108441000	3.064713000	1.237173000
C	-3.873422000	-0.051081000	-0.780993000
C	-2.384563000	0.433623000	-0.788079000
C	-1.245610000	2.325108000	0.336652000
C	0.017102000	1.566370000	-0.023859000
C	-0.176987000	0.111565000	0.462708000
C	-1.239765000	-0.563850000	-0.429471000
C	-4.486286000	-0.243248000	-2.195853000
C	-5.005891000	-1.697870000	-2.194592000
C	-4.071925000	-1.458530000	-0.135107000
C	-3.010338000	-1.793357000	0.889843000
C	-1.663332000	-1.921731000	0.181197000
C	0.149356000	-4.471029000	-0.113605000
C	-1.016435000	-4.077668000	-0.966849000
C	-1.783249000	-2.994648000	-0.868718000
C	-2.861625000	-2.712164000	-1.870883000
C	1.299486000	2.279642000	0.445495000
C	2.480860000	1.311315000	0.503956000
C	2.205099000	0.019969000	-0.263743000
C	1.590975000	-1.402580000	1.427946000

C	3.356676000	-1.003485000	-0.114776000
C	2.943353000	-1.681532000	1.171433000
C	0.979083000	-1.875565000	2.580437000
C	1.730245000	-2.667765000	3.449978000
C	3.054694000	-2.975967000	3.185226000
C	3.668810000	-2.471296000	2.034996000
C	3.850206000	1.928880000	0.187603000
C	4.900924000	0.931690000	0.675355000
C	4.757801000	-0.342515000	-0.126336000
C	4.678003000	-1.409556000	-2.147954000
C	3.455968000	-1.945055000	-1.340000000
C	4.351887000	0.993880000	-2.168643000
C	4.106122000	2.199501000	-1.277016000
C	4.123931000	3.416907000	-1.818458000
C	3.817322000	4.725928000	-1.157769000
H	-3.409285000	3.801205000	1.594863000
H	-2.981049000	-1.002269000	1.645843000
H	-5.054738000	-1.452114000	0.341900000
H	-6.037152000	-1.731811000	-1.839370000
H	-5.281604000	0.474118000	-2.386630000
H	-2.996456000	-3.559365000	-2.542747000
H	-6.622554000	0.404197000	-0.093376000
H	-7.426659000	2.326235000	1.251420000
H	-5.827990000	4.005686000	2.085089000
H	0.064960000	1.531828000	-1.119037000
H	-0.518315000	0.135026000	1.502891000
H	1.041788000	-4.591014000	-0.731509000
H	0.381212000	-3.748001000	0.664591000
H	-2.173981000	0.812747000	-1.790156000
H	-0.903688000	-2.216985000	0.897011000

H	-2.525889000	-1.864958000	-2.493401000
H	-1.245470000	-4.767482000	-1.775079000
H	-3.730967000	-0.096941000	-2.966920000
H	-4.969414000	-2.168023000	-3.175975000
H	-3.258136000	-2.727083000	1.394135000
H	-0.713545000	-0.772260000	-1.363785000
H	-0.033744000	-5.436904000	0.361330000
H	1.505868000	3.075806000	-0.280498000
H	0.423047000	3.423437000	1.723334000
H	3.029777000	5.246837000	-1.705712000
H	3.488176000	4.619713000	-0.126959000
H	4.692043000	5.379306000	-1.172222000
H	4.380963000	3.487990000	-2.872451000
H	4.926840000	1.313492000	-3.038568000
H	3.384401000	0.654441000	-2.561584000
H	4.757120000	0.723241000	1.736739000
H	5.908303000	1.326286000	0.538763000
H	3.936451000	2.854196000	0.754208000
H	2.528190000	1.013265000	1.556504000
H	1.964395000	0.209178000	-1.307904000
H	-0.051093000	-1.643857000	2.814820000
H	1.262839000	-3.043327000	4.350418000
H	3.615633000	-3.593422000	3.872368000
H	4.709185000	-2.695030000	1.831489000
H	3.631496000	-2.971123000	-1.020053000
H	5.509890000	-2.108619000	-2.058002000
H	2.529789000	-1.936875000	-1.915168000
H	4.467671000	-1.279063000	-3.207950000
H	5.468805000	-1.090129000	0.239661000

Strychnobaillonine (Conformer 2, *E* = -1920.100348 Hartree), solvent: Chloroform

O	1.233537000	2.712588000	1.805443000
O	-1.108593000	3.441185000	0.734558000
N	-2.304551000	1.615963000	0.119392000
N	-4.634991000	-2.392901000	-0.250999000
N	1.112106000	-0.744915000	0.320425000
N	5.207326000	-0.154949000	-1.437309000
C	-5.374737000	3.616612000	0.850211000
C	-6.305326000	2.680144000	0.421008000
C	-5.882255000	1.448720000	-0.071284000
C	-4.528572000	1.176112000	-0.136478000
C	-3.607448000	2.128313000	0.290755000
C	-4.008392000	3.358775000	0.792993000
C	-3.833273000	-0.090725000	-0.574998000
C	-2.367345000	0.392465000	-0.714354000
C	-1.148320000	2.290380000	0.315024000
C	0.095057000	1.483285000	-0.006909000
C	-0.119583000	0.027207000	0.455858000
C	-1.227289000	-0.610152000	-0.411758000
C	-4.373842000	-0.683078000	-1.889334000
C	-5.288142000	-1.816159000	-1.422241000
C	-4.077780000	-1.244482000	0.481070000
C	-2.808832000	-1.625622000	1.228160000
C	-1.716783000	-1.935120000	0.200288000
C	-0.047284000	-3.199945000	-2.065657000
C	-1.503807000	-3.446745000	-1.809102000
C	-2.238293000	-2.933654000	-0.824873000
C	-3.640774000	-3.425595000	-0.560289000
C	1.375525000	2.172736000	0.505344000
C	2.537279000	1.186742000	0.585239000

C	2.276905000	-0.056878000	-0.260664000
C	1.607136000	-1.592033000	1.310357000
C	3.418269000	-1.098077000	-0.151092000
C	2.962553000	-1.865905000	1.067796000
C	0.963926000	-2.140000000	2.412240000
C	1.688668000	-2.993185000	3.245047000
C	3.018008000	-3.291604000	2.993366000
C	3.662432000	-2.716543000	1.894697000
C	3.926025000	1.802459000	0.365986000
C	4.943078000	0.762941000	0.834360000
C	4.825627000	-0.453158000	-0.056236000
C	4.839146000	-1.388443000	-2.138817000
C	3.558774000	-1.943357000	-1.443036000
C	4.514076000	1.009205000	-2.021121000
C	4.243257000	2.160128000	-1.067577000
C	4.286442000	3.408340000	-1.531738000
C	3.955302000	4.674789000	-0.803094000
H	-3.285568000	4.087074000	1.118315000
H	-2.505266000	-0.800440000	1.875984000
H	-4.835579000	-0.888493000	1.180935000
H	-6.260518000	-1.419352000	-1.120072000
H	-4.877590000	0.067718000	-2.496763000
H	-3.591264000	-4.104395000	0.297359000
H	-6.607923000	0.715626000	-0.399385000
H	-7.361312000	2.906537000	0.470490000
H	-5.712115000	4.569873000	1.233687000
H	0.166562000	1.462350000	-1.101701000
H	-0.419453000	0.041953000	1.509351000
H	0.446084000	-2.717642000	-1.222614000
H	0.461548000	-4.144463000	-2.263386000

H	-2.254230000	0.731536000	-1.748182000
H	-0.859123000	-2.397671000	0.688782000
H	-4.002881000	-4.018172000	-1.399441000
H	-1.988548000	-4.151725000	-2.478958000
H	-3.548438000	-1.098377000	-2.470922000
H	-5.463914000	-2.575957000	-2.181175000
H	-3.006555000	-2.481281000	1.874232000
H	-0.744885000	-0.827638000	-1.364226000
H	0.104222000	-2.572159000	-2.948625000
H	1.614951000	2.973089000	-0.205280000
H	0.504967000	3.346432000	1.757492000
H	3.569349000	4.504568000	0.199172000
H	4.834131000	5.318414000	-0.727479000
H	3.202251000	5.238525000	-1.357028000
H	4.585002000	3.543084000	-2.568370000
H	5.117265000	1.375831000	-2.852454000
H	3.557848000	0.697732000	-2.462685000
H	4.747747000	0.486772000	1.871822000
H	5.959466000	1.152720000	0.771202000
H	3.997873000	2.689998000	0.991774000
H	2.527692000	0.837182000	1.623299000
H	2.065887000	0.196249000	-1.298003000
H	-0.068987000	-1.913514000	2.639235000
H	1.197139000	-3.423963000	4.107137000
H	3.558602000	-3.956880000	3.651616000
H	4.706461000	-2.933106000	1.702940000
H	3.678600000	-2.998943000	-1.203470000
H	5.653786000	-2.105357000	-2.033084000
H	2.670059000	-1.850580000	-2.067194000
H	4.700388000	-1.196838000	-3.201372000

H	5.511047000	-1.232250000	0.291597000

Equilibrium geometries of molecules from set **1** calculated at the **DFT(M06-2X)** level of theory with **cc-pVQZ** basis set within the IEF-PCM model. Cartesian coordinates are given for standard orientation in form (atomic label, X, Y, Z). The X, Y, Z are given in Å.

12-28-oxaircinal (*E* = -1272.3443898 Hartree), solvent: Chloroform

O	1.590597000	-0.779208000	1.826498000
O	-0.137032000	4.735176000	1.501053000
N	1.761575000	-0.505500000	-0.456194000
N	-2.189306000	0.492085000	-1.533428000
C	0.065162000	1.165963000	-0.860672000
C	0.388044000	-0.135704000	-0.097615000
C	0.393252000	-0.095937000	1.457106000
C	-0.578176000	2.286464000	-0.009488000
C	1.450084000	1.607155000	-1.390559000
C	2.485552000	0.757226000	-0.645856000
C	-0.863847000	0.808805000	-2.023687000
C	2.178747000	-1.348533000	0.644918000
C	-2.101295000	2.172161000	0.192581000
C	0.084197000	2.380778000	1.333943000
C	0.499625000	1.299615000	1.995383000
C	-0.847849000	-0.807514000	2.019306000
C	-2.825434000	1.707701000	-1.058183000
C	3.666999000	-1.494640000	0.863965000
C	3.783468000	0.678736000	-1.394924000
C	-2.985128000	-0.297963000	-2.455389000
C	-0.837755000	-2.327683000	1.807304000
C	4.362795000	-2.323555000	-0.211381000
C	0.226007000	3.691337000	1.986811000
C	4.459439000	-0.379399000	-1.830843000

C	4.112395000	-1.832600000	-1.645375000
C	-4.294920000	-0.794215000	-1.838457000
C	-2.195080000	-2.927654000	2.056480000
C	-4.167695000	-1.316007000	-0.408288000
C	-3.168845000	-2.464160000	-0.237741000
C	-3.183561000	-2.989184000	1.168499000
H	-0.297497000	-0.921091000	-0.411213000
H	-0.405535000	3.223085000	-0.544055000
H	1.632558000	2.671838000	-1.255896000
H	1.521478000	1.388959000	-2.457637000
H	2.690693000	1.237731000	0.323310000
H	-0.890341000	1.640199000	-2.748563000
H	-0.448916000	-0.058696000	-2.540804000
H	1.731279000	-2.336943000	0.486219000
H	-2.478008000	3.141342000	0.520936000
H	-2.315441000	1.461347000	0.989318000
H	0.944965000	1.395723000	2.980960000
H	-1.720401000	-0.397341000	1.509234000
H	-0.960685000	-0.586565000	3.082256000
H	-3.868063000	1.510944000	-0.818270000
H	-2.810207000	2.489660000	-1.835546000
H	3.798303000	-1.975830000	1.832722000
H	4.115478000	-0.504429000	0.941574000
H	4.208745000	1.658818000	-1.590188000
H	-2.375156000	-1.156570000	-2.742218000
H	-3.209801000	0.253064000	-3.382348000
H	-0.098764000	-2.777530000	2.468200000
H	-0.524296000	-2.557121000	0.790012000
H	4.033625000	-3.361954000	-0.138690000
H	5.434217000	-2.319907000	-0.007179000

H	0.702418000	3.667355000	2.982459000
H	5.391133000	-0.176699000	-2.347164000
H	3.070285000	-2.003182000	-1.901903000
H	4.715021000	-2.432581000	-2.325672000
H	-4.684756000	-1.583289000	-2.484678000
H	-5.040624000	0.001576000	-1.856635000
H	-2.379797000	-3.312311000	3.053132000
H	-5.151046000	-1.647506000	-0.068531000
H	-3.868195000	-0.502236000	0.255783000
H	-2.174757000	-2.115396000	-0.516023000
H	-3.434389000	-3.268762000	-0.930148000
H	-4.125011000	-3.426714000	1.487172000

Anabsinthin (Conformer 1, *E* = -1618.7181575 Hartree), solvent: Acetonitrile

O	-2.328706000	1.156332000	-2.239895000
O	-2.558685000	-1.948749000	0.638142000
O	2.366268000	0.232010000	3.123776000
O	3.081189000	-1.637477000	-1.222168000
O	-3.748557000	-3.381913000	1.849291000
O	4.571229000	-2.143899000	-2.793897000
C	-1.643483000	0.157646000	-0.258728000
C	-1.971154000	1.658590000	-0.003424000
C	-0.495277000	2.114203000	-0.088498000
C	-1.166331000	0.489988000	-1.707789000
C	-0.520460000	-0.126385000	0.770954000
C	0.146852000	1.251784000	1.005154000
C	-2.803246000	2.075644000	-1.228555000
C	-0.089639000	1.562194000	-1.472520000

C	-2.809908000	-0.831434000	-0.250389000
C	1.677311000	1.053044000	0.906928000
C	0.620151000	-1.025518000	0.381697000
C	-4.172821000	-0.311318000	0.195833000
C	-4.317959000	1.911854000	-1.041104000
C	1.787555000	-0.379825000	0.418397000
C	-0.759769000	-0.599850000	-2.664049000
C	-4.864619000	0.499021000	-0.882776000
C	2.472743000	1.331326000	2.221347000
C	-2.567693000	3.520693000	-1.663732000
C	-4.871239000	-1.610563000	0.589130000
C	3.100534000	-1.017182000	0.095000000
C	0.405715000	-2.474748000	0.092679000
C	3.974542000	1.528252000	1.972002000
C	-3.715467000	-2.434217000	1.114245000
C	4.304358000	-0.090614000	0.033828000
C	4.776402000	0.363688000	1.401376000
C	1.967625000	2.608127000	2.898454000
C	-6.020698000	-1.496762000	1.570628000
C	5.274289000	-0.942002000	-0.781113000
C	4.325486000	-1.646139000	-1.730743000
C	6.395665000	-0.211569000	-1.492113000
H	-2.437830000	1.890225000	0.951742000
H	-0.321852000	3.179602000	0.043030000
H	-0.995506000	-0.512116000	1.674470000
H	-0.151071000	1.649554000	1.970625000
H	0.910466000	1.136956000	-1.484939000
H	-0.142443000	2.299352000	-2.269613000
H	-2.928250000	-1.259370000	-1.248171000
H	2.083255000	1.755635000	0.171771000

H	-4.046544000	0.289432000	1.102907000
H	-4.811706000	2.374989000	-1.896868000
H	-4.594995000	2.506270000	-0.167495000
H	-1.530872000	-1.359102000	-2.782897000
H	-0.571956000	-0.157498000	-3.642202000
H	0.159145000	-1.074307000	-2.325614000
H	-4.782992000	-0.041647000	-1.828001000
H	-5.928816000	0.571914000	-0.654298000
H	-3.086326000	3.703993000	-2.603533000
H	-2.973419000	4.201077000	-0.914879000
H	-1.518166000	3.756400000	-1.802556000
H	-5.205911000	-2.117508000	-0.323919000
H	3.309309000	-1.820015000	0.810329000
H	-0.313638000	-2.613865000	-0.714951000
H	1.327908000	-2.982090000	-0.174608000
H	-0.028170000	-2.956798000	0.969595000
H	4.417483000	1.804033000	2.929706000
H	4.085393000	2.394771000	1.316117000
H	4.039596000	0.781435000	-0.574526000
H	4.756281000	-0.483046000	2.090708000
H	5.816918000	0.684224000	1.330980000
H	0.995517000	2.460312000	3.361918000
H	1.882800000	3.422060000	2.177722000
H	2.662490000	2.909250000	3.680125000
H	-5.691642000	-1.013881000	2.490020000
H	-6.412039000	-2.479968000	1.821641000
H	-6.828483000	-0.906343000	1.142706000
H	1.435940000	0.030045000	3.263031000
H	5.685401000	-1.723096000	-0.130596000
H	6.990759000	-0.902583000	-2.084522000

H	7.051733000	0.272769000	-0.771614000
H	5.993545000	0.550914000	-2.158027000

Anabsinthin (Conformer 2, *E* = -1618.7180442 Hartree), solvent: Acetonitrile

O	-2.159775000	2.328580000	-1.029579000
O	-2.611601000	-1.869802000	-0.713079000
O	1.547883000	-1.734720000	3.019734000
O	3.045043000	-0.509557000	-1.997873000
O	-3.891123000	-3.685292000	-0.656170000
O	4.453945000	0.325006000	-3.502770000
C	-1.589869000	0.284515000	-0.088478000
C	-1.870446000	1.320047000	1.039069000
C	-0.375930000	1.653182000	1.264444000
C	-1.042237000	1.417310000	-1.016510000
C	-0.522436000	-0.639728000	0.554098000
C	0.190849000	0.268270000	1.592960000
C	-2.640218000	2.448075000	0.330384000
C	0.061246000	2.057073000	-0.159331000
C	-2.791678000	-0.431052000	-0.706506000
C	1.701196000	0.071804000	1.379615000
C	0.587934000	-1.150430000	-0.331325000
C	-4.146923000	-0.235832000	-0.035505000
C	-4.166029000	2.282934000	0.365565000
C	1.774717000	-0.698614000	0.079709000
C	-0.639605000	1.137530000	-2.440656000
C	-4.773621000	1.101795000	-0.380435000
C	2.391280000	-0.681004000	2.549514000
C	-2.335578000	3.837909000	0.886185000
C	-4.906459000	-1.462929000	-0.532950000
C	3.095037000	-0.905958000	-0.601246000

C	0.329476000	-2.066136000	-1.481618000
C	3.729393000	-1.326249000	2.145862000
C	-3.799448000	-2.489046000	-0.639218000
C	4.212946000	-0.038582000	-0.029729000
C	4.733011000	-0.500983000	1.333925000
C	2.568927000	0.261223000	3.727039000
C	-6.075259000	-1.928220000	0.312711000
C	5.195347000	-0.021536000	-1.194800000
C	4.244833000	-0.031497000	-2.377710000
C	6.189473000	1.121657000	-1.234710000
H	-2.363877000	0.935088000	1.929313000
H	-0.169496000	2.396918000	2.030469000
H	-1.043828000	-1.475129000	1.023640000
H	-0.094728000	-0.007500000	2.605283000
H	1.045871000	1.679417000	-0.422457000
H	0.064659000	3.130323000	-0.330038000
H	-2.891157000	-0.133735000	-1.752848000
H	2.200217000	1.040898000	1.300899000
H	-4.024507000	-0.331577000	1.048438000
H	-4.609251000	3.199328000	-0.027626000
H	-4.452716000	2.230650000	1.418395000
H	-0.419019000	2.083639000	-2.934531000
H	0.259535000	0.524992000	-2.465953000
H	-1.427277000	0.640870000	-3.004946000
H	-4.688465000	1.255972000	-1.458344000
H	-5.840063000	1.071508000	-0.152402000
H	-2.749432000	3.931110000	1.890491000
H	-1.273290000	4.050883000	0.936716000
H	-2.806490000	4.590465000	0.255412000
H	-5.237886000	-1.276601000	-1.561530000

H	3.387803000	-1.960144000	-0.594162000
H	-0.128719000	-2.986598000	-1.117960000
H	-0.381020000	-1.631526000	-2.185028000
H	1.242877000	-2.310436000	-2.017257000
H	3.493105000	-2.232798000	1.583704000
H	4.211659000	-1.666338000	3.063046000
H	3.810696000	0.974056000	0.052559000
H	5.614106000	-1.127098000	1.180789000
H	5.069621000	0.363311000	1.906747000
H	2.919276000	-0.292296000	4.596790000
H	1.626132000	0.745533000	3.979938000
H	3.297794000	1.034501000	3.488675000
H	-6.510980000	-2.835972000	-0.098280000
H	-6.847800000	-1.162701000	0.347625000
H	-5.750682000	-2.135030000	1.331869000
H	1.370521000	-2.330902000	2.283984000
H	5.724092000	-0.981227000	-1.227338000
H	6.861307000	1.068500000	-0.380116000
H	5.671026000	2.079368000	-1.204895000
H	6.785835000	1.083378000	-2.143315000

Betulinic Acid (*E* = -1397.8002624 Hartree), solvent: Pyridine

C	4.178567000	1.212919000	0.362132000
C	3.151707000	0.295542000	-0.334266000
C	3.793772000	-1.099191000	-0.367767000
C	5.243126000	-0.746243000	-0.744835000
C	5.545388000	0.494129000	0.112346000
C	1.713620000	0.253868000	0.170322000
C	0.839684000	-0.699967000	-0.697449000
C	1.545129000	-2.067338000	-0.886251000

C	3.009403000	-1.976527000	-1.336758000
C	1.067721000	1.632200000	0.273582000
C	-0.369095000	1.568060000	0.799686000
C	-1.249540000	0.529147000	0.090175000
C	-0.554412000	-0.865106000	0.045606000
C	-2.741858000	0.537826000	0.568809000
C	-3.504405000	-0.449260000	-0.360305000
C	-2.907860000	-1.852561000	-0.266348000
C	-1.447948000	-1.861753000	-0.712027000
C	-3.307770000	1.951283000	0.320157000
C	-4.826533000	2.022653000	0.408178000
C	-5.493568000	1.042322000	-0.532905000
C	-5.057360000	-0.417944000	-0.289169000
C	4.213691000	2.637865000	-0.136696000
C	4.230977000	3.662360000	0.709495000
C	4.285230000	2.850638000	-1.623333000
C	3.863569000	-1.754237000	1.004056000
O	3.737920000	-1.209518000	2.070331000
O	4.159076000	-3.062515000	0.922516000
C	0.666305000	-0.120681000	-2.117162000
C	-0.346919000	-1.424857000	1.469302000
C	-2.895426000	0.222288000	2.069592000
C	-5.623328000	-1.270038000	-1.434131000
C	-5.641375000	-0.958787000	1.019128000
O	-6.896146000	1.234244000	-0.391840000
H	3.977464000	1.233639000	1.431616000
H	3.136760000	0.597933000	-1.384379000
H	5.254639000	-0.495393000	-1.806865000
H	5.946352000	-1.561953000	-0.586585000
H	6.267694000	1.150969000	-0.368581000

H	5.973163000	0.197527000	1.068339000
H	1.756851000	-0.161035000	1.178050000
H	0.988106000	-2.654860000	-1.616619000
H	1.527009000	-2.642815000	0.039388000
H	3.089883000	-1.534698000	-2.330280000
H	3.435669000	-2.975977000	-1.399254000
H	1.091134000	2.126407000	-0.701035000
H	1.661032000	2.259455000	0.938812000
H	-0.815611000	2.556074000	0.700311000
H	-0.330052000	1.362709000	1.870023000
H	-1.333855000	0.851790000	-0.949700000
H	-3.277742000	-0.094917000	-1.376541000
H	-3.457595000	-2.541808000	-0.904908000
H	-3.004702000	-2.243473000	0.746966000
H	-1.434067000	-1.636512000	-1.779090000
H	-1.039566000	-2.867933000	-0.598225000
H	-2.997230000	2.286026000	-0.674860000
H	-2.881623000	2.654419000	1.035530000
H	-5.163607000	3.028201000	0.155173000
H	-5.174708000	1.830178000	1.424316000
H	-5.197145000	1.297685000	-1.559441000
H	4.306924000	4.683317000	0.359309000
H	4.163540000	3.507693000	1.778635000
H	3.321254000	2.646350000	-2.092688000
H	4.563063000	3.874773000	-1.860025000
H	5.009844000	2.177402000	-2.086042000
H	4.240811000	-3.403706000	1.823375000
H	0.185901000	0.851224000	-2.139962000
H	1.629636000	-0.000031000	-2.607001000
H	0.084061000	-0.792020000	-2.744624000

H	-1.262348000	-1.873049000	1.842075000
H	0.410701000	-2.204319000	1.488985000
H	-0.048078000	-0.669665000	2.190314000
H	-2.015466000	0.534975000	2.626373000
H	-3.740052000	0.756940000	2.496999000
H	-3.052429000	-0.833534000	2.272476000
H	-5.152202000	-1.018635000	-2.385701000
H	-5.481191000	-2.333059000	-1.252578000
H	-6.699161000	-1.122716000	-1.536248000
H	-6.718930000	-1.081050000	0.918856000
H	-5.216477000	-1.936059000	1.248187000
H	-5.463656000	-0.306098000	1.868120000
H	-7.353755000	0.730278000	-1.068032000

Icajine (Conformer 1, *E* = -1187.9893928 Hartree), solvent: Chloroform

O	-0.137219000	-2.855822000	1.239003000
O	2.082759000	3.027227000	0.674943000
O	-2.554537000	3.191600000	-0.399704000
N	-1.453550000	1.200587000	-0.469733000
N	2.102349000	-2.078910000	-0.553813000
C	-0.155808000	0.525782000	-0.302505000
C	0.212141000	-1.726641000	0.976443000
C	1.378292000	-1.722417000	-1.762115000
C	-0.125740000	-1.745167000	-1.492359000
C	-0.483755000	-0.987980000	-0.193678000
C	-1.992568000	-0.978647000	-0.020156000
C	-2.847304000	-2.033779000	0.235109000
C	-4.218656000	-1.796803000	0.275799000
C	-4.716701000	-0.520653000	0.053538000

C	-3.869435000	0.550545000	-0.210677000
C	-2.505744000	0.298531000	-0.239762000
C	1.212015000	-1.013443000	1.868642000
C	1.751820000	0.306581000	1.312268000
C	0.513184000	1.118087000	0.930907000
C	0.727014000	2.638698000	0.773496000
C	2.822776000	2.643162000	-0.459597000
C	3.159439000	1.188835000	-0.579029000
C	2.739236000	0.163101000	0.153617000
C	3.220581000	-1.225004000	-0.193244000
C	-0.145646000	3.211128000	-0.359781000
C	-1.513677000	2.562067000	-0.409466000
C	2.348702000	-3.495266000	-0.410834000
H	0.456433000	0.717679000	-1.182369000
H	1.621734000	-2.399531000	-2.587761000
H	1.687103000	-0.726504000	-2.075681000
H	-0.477863000	-2.770972000	-1.376350000
H	-0.671775000	-1.306977000	-2.329563000
H	-2.446839000	-3.021924000	0.404786000
H	-4.898163000	-2.612139000	0.477499000
H	-5.783379000	-0.349480000	0.084236000
H	-4.250055000	1.542567000	-0.383110000
H	1.990832000	-1.735187000	2.108147000
H	0.694142000	-0.799408000	2.806675000
H	2.267690000	0.823224000	2.122727000
H	-0.211126000	0.997627000	1.742659000
H	0.410484000	3.110081000	1.701856000
H	3.753175000	3.209704000	-0.399193000
H	2.337011000	2.973313000	-1.385284000
H	3.846201000	0.982638000	-1.394750000

H	3.701118000	-1.677511000	0.677321000
H	3.981354000	-1.168296000	-0.982444000
H	-0.275397000	4.282509000	-0.248814000
H	0.329615000	3.025457000	-1.323976000
H	3.108008000	-3.863292000	-1.112251000
H	1.422561000	-4.042885000	-0.579353000
H	2.676526000	-3.710767000	0.606188000

Icajine (Conformer 2, *E* = -1187.9903544 Hartree), solvent: Chloroform

O	-0.061645000	-2.767115000	1.333297000
O	1.629081000	2.914576000	-0.584732000
O	-2.857191000	3.024749000	-0.229876000
N	-1.541128000	1.186681000	-0.342098000
N	2.183943000	-2.029413000	-0.485794000
C	-0.195678000	0.580037000	-0.276591000
C	0.262684000	-1.641953000	1.027798000
C	1.482904000	-1.640938000	-1.696676000
C	-0.019515000	-1.701703000	-1.455282000
C	-0.439194000	-0.954869000	-0.166625000
C	-1.947852000	-1.049156000	-0.052765000
C	-2.733127000	-2.173454000	0.116263000
C	-4.118059000	-2.037870000	0.104685000
C	-4.695709000	-0.791331000	-0.088767000
C	-3.917468000	0.347460000	-0.267709000
C	-2.537662000	0.197654000	-0.238856000
C	1.243012000	-0.871801000	1.895269000
C	1.798391000	0.415381000	1.276184000
C	0.565071000	1.232436000	0.867658000
C	0.778399000	2.707476000	0.524438000
C	2.999720000	2.663095000	-0.329833000

C	3.378325000	1.219591000	-0.493646000
C	2.845479000	0.208423000	0.184735000
C	3.306314000	-1.196242000	-0.106990000
C	-0.554739000	3.383077000	0.197854000
C	-1.750552000	2.519170000	-0.160885000
C	2.414136000	-3.449559000	-0.360415000
H	0.333057000	0.800293000	-1.204994000
H	1.774867000	-0.625696000	-1.961571000
H	1.756389000	-2.279170000	-2.544252000
H	-0.344599000	-2.737400000	-1.346456000
H	-0.562710000	-1.280849000	-2.302918000
H	-2.270176000	-3.137698000	0.262784000
H	-4.744020000	-2.908122000	0.239570000
H	-5.772226000	-0.696056000	-0.104723000
H	-4.362609000	1.314572000	-0.419663000
H	0.701825000	-0.603977000	2.806315000
H	2.017449000	-1.574486000	2.196679000
H	2.299786000	0.968822000	2.077093000
H	-0.097308000	1.230792000	1.740812000
H	1.223835000	3.204187000	1.393540000
H	3.251859000	3.017571000	0.677708000
H	3.554267000	3.272919000	-1.038640000
H	4.133274000	0.998599000	-1.238939000
H	3.744806000	-1.633430000	0.793670000
H	4.098022000	-1.173817000	-0.867649000
H	-0.899309000	3.966638000	1.050068000
H	-0.399868000	4.091771000	-0.614238000
H	3.173427000	-3.816434000	-1.062788000
H	1.484255000	-3.986757000	-0.540161000
H	2.734765000	-3.680389000	0.655434000

Iguesterin (*E* = -1239.0871068 Hartree), solvent: Chloroform

O	-6.527085000	0.802564000	-0.975730000
O	-5.270657000	2.639354000	0.398451000
C	1.661620000	0.217380000	0.427840000
C	1.046506000	-1.221163000	0.556508000
C	3.202574000	0.150133000	0.639399000
C	3.966210000	-0.900859000	-0.219052000
C	1.727509000	-2.149931000	-0.464407000
C	1.011063000	1.085259000	1.518362000
C	3.234261000	-2.248298000	-0.248806000
C	-0.457409000	-1.113257000	0.309372000
C	-1.277112000	-0.064086000	1.053424000
C	-0.494347000	1.241950000	1.323741000
C	3.903690000	1.515334000	0.527404000
C	1.306669000	0.845100000	-0.931211000
C	4.203882000	-0.444759000	-1.671150000
C	1.275641000	-1.856296000	1.948680000
C	5.342715000	-1.125201000	0.420145000
C	4.395019000	1.896412000	-0.842517000
C	-2.611745000	0.248339000	0.384877000
C	4.530135000	1.007229000	-1.818435000
C	-1.098802000	-1.956857000	-0.517382000
C	-1.711889000	-0.687246000	2.419734000
C	-3.248286000	-0.771052000	-0.435457000
C	-2.496773000	-1.832258000	-0.820691000
C	4.739987000	3.344903000	-1.017681000
C	-3.277008000	1.392602000	0.658513000
C	-4.619929000	-0.607478000	-0.890873000
C	-4.624470000	1.612680000	0.184730000

C	-5.261305000	0.537174000	-0.589220000
C	-5.287836000	-1.677028000	-1.702494000
H	3.330839000	-0.167583000	1.678018000
H	1.309854000	-3.153897000	-0.381806000
H	1.509847000	-1.806059000	-1.476931000
H	1.240098000	0.685096000	2.505202000
H	1.431954000	2.088978000	1.501049000
H	3.670054000	-2.856180000	-1.045914000
H	3.438607000	-2.791362000	0.673945000
H	-0.666173000	1.923431000	0.492214000
H	-0.917216000	1.726741000	2.203637000
H	3.254907000	2.312661000	0.889924000
H	4.763363000	1.535016000	1.204758000
H	0.237281000	0.798051000	-1.131000000
H	1.595432000	1.896835000	-0.939181000
H	1.806382000	0.365944000	-1.763879000
H	5.030237000	-1.040793000	-2.070774000
H	3.351240000	-0.699950000	-2.305709000
H	2.326815000	-2.058377000	2.126311000
H	0.934690000	-1.244542000	2.772246000
H	0.742455000	-2.806004000	1.995489000
H	5.244633000	-1.372711000	1.478210000
H	5.976920000	-0.243909000	0.329307000
H	5.851916000	-1.953718000	-0.074001000
H	4.894588000	1.336776000	-2.785635000
H	-0.569112000	-2.752427000	-1.016169000
H	-0.866929000	-0.808725000	3.087005000
H	-2.184932000	-1.656254000	2.269252000
H	-2.426189000	-0.016085000	2.892565000
H	-2.938071000	-2.596055000	-1.446638000

H	5.463416000	3.664114000	-0.264473000
H	3.853136000	3.968625000	-0.884258000
H	5.157077000	3.542811000	-2.002655000
H	-2.852648000	2.183884000	1.258346000
H	-5.255473000	-2.637287000	-1.188415000
H	-4.794958000	-1.804497000	-2.666832000
H	-6.325937000	-1.419546000	-1.885158000
H	-6.719593000	1.689287000	-0.629918000

Itoaic acid (Conformer 1, *E* = -1623.5139697 Hartree), solvent: Chloroform

C	-3.415649000	-1.104545000	-0.270712000
C	-2.562960000	0.164890000	0.056228000
C	-1.257818000	-0.105613000	0.899207000
C	-0.420935000	-1.133181000	0.061185000
C	-1.215450000	-2.423923000	-0.121481000
C	-2.494866000	-2.161047000	-0.900290000
C	-0.435239000	1.211636000	1.003893000
C	1.055108000	1.014826000	1.296784000
C	1.779005000	0.048950000	0.346048000
C	1.064240000	-1.341222000	0.477354000
C	3.294769000	-0.066202000	0.749650000
C	4.089830000	-1.285599000	0.183456000
C	3.235827000	-2.554886000	-0.049698000
C	1.768881000	-2.365288000	-0.431131000
C	4.129555000	1.246872000	0.658531000
C	5.087272000	1.520497000	-0.532774000
C	5.778164000	0.231605000	-0.987801000
C	4.805564000	-0.928825000	-1.132216000
C	-3.384991000	1.339750000	0.599231000
C	-4.459131000	1.912699000	-0.334313000

C	-5.717615000	1.069999000	-0.297327000
O	-5.668796000	-0.170491000	-0.750327000
C	-4.478468000	-0.740379000	-1.345494000
O	-4.787695000	3.218152000	0.063987000
O	-6.742857000	1.500608000	0.168921000
C	-5.023361000	-1.901295000	-2.159785000
C	-4.142184000	-1.710962000	0.936508000
C	-1.600527000	-0.570216000	2.329190000
O	-0.940710000	2.128549000	1.967602000
C	1.166927000	-1.906483000	1.907106000
C	5.148679000	-1.660258000	1.234721000
C	6.144416000	2.511275000	-0.036510000
C	4.408867000	2.165639000	-1.746254000
C	1.685879000	0.612080000	-1.074990000
O	1.509324000	1.943750000	-1.088403000
O	1.789884000	0.009071000	-2.112089000
H	-2.183003000	0.497761000	-0.918727000
H	-0.366471000	-0.697533000	-0.943531000
H	-1.436464000	-2.896156000	0.836270000
H	-0.635286000	-3.146800000	-0.688418000
H	-3.046098000	-3.093505000	-1.020867000
H	-2.211534000	-1.828923000	-1.904077000
H	-0.533906000	1.736677000	0.053817000
H	1.171940000	0.646036000	2.316546000
H	1.504140000	2.003101000	1.278708000
H	3.212105000	-0.246970000	1.823005000
H	3.275680000	-3.171306000	0.847284000
H	3.724841000	-3.149167000	-0.823526000
H	1.289058000	-3.335711000	-0.314209000
H	1.665397000	-2.078824000	-1.472271000

H	4.758624000	1.251695000	1.549664000
H	3.475166000	2.112207000	0.768791000
H	6.563174000	-0.035851000	-0.278665000
H	6.281950000	0.419794000	-1.938665000
H	5.350681000	-1.814816000	-1.463798000
H	4.064801000	-0.715500000	-1.903569000
H	-2.724796000	2.177613000	0.786720000
H	-3.852129000	1.109537000	1.558545000
H	-4.077284000	1.946336000	-1.358356000
H	-4.051148000	-0.011147000	-2.035948000
H	-5.707620000	3.196265000	0.359271000
H	-4.261565000	-2.318719000	-2.810008000
H	-5.409976000	-2.690080000	-1.517268000
H	-5.839366000	-1.537332000	-2.778973000
H	-4.636083000	-0.959605000	1.549327000
H	-4.910798000	-2.403942000	0.596742000
H	-3.462394000	-2.268416000	1.572171000
H	-2.558046000	-0.170036000	2.652289000
H	-0.863365000	-0.230667000	3.054570000
H	-1.644541000	-1.649880000	2.425512000
H	-0.766950000	1.786830000	2.848118000
H	0.633205000	-2.856799000	1.944085000
H	0.748708000	-1.270302000	2.673156000
H	2.194625000	-2.109961000	2.190684000
H	5.783851000	-0.822082000	1.512870000
H	4.665627000	-2.022351000	2.142790000
H	5.790730000	-2.457264000	0.855479000
H	6.857472000	2.738652000	-0.829861000
H	5.682615000	3.447913000	0.280524000
H	6.697329000	2.102007000	0.809543000

H	5.176303000	2.479345000	-2.454962000
H	3.750479000	1.478337000	-2.272436000
H	3.840157000	3.047766000	-1.454342000
H	1.544234000	2.229888000	-2.011408000

Itoaic acid (Conformer 2, *E* = -1623.5142836 Hartree), solvent: Chloroform

C	-3.484662000	-1.101921000	0.155140000
C	-2.633636000	0.204376000	0.012745000
C	-1.239774000	0.175887000	0.751155000
C	-0.488495000	-1.057778000	0.145718000
C	-1.260224000	-2.337650000	0.456754000
C	-2.614552000	-2.310286000	-0.232493000
C	-0.420875000	1.448495000	0.391639000
C	1.080809000	1.317083000	0.654286000
C	1.739047000	0.135495000	-0.073625000
C	1.040119000	-1.176710000	0.400444000
C	3.274281000	0.063730000	0.255416000
C	3.971649000	-1.277552000	-0.199266000
C	3.015590000	-2.176400000	-0.992571000
C	1.624198000	-2.391075000	-0.378801000
C	4.057997000	1.298025000	-0.215960000
C	5.557864000	1.272773000	0.116147000
C	6.187128000	-0.049803000	-0.402587000
C	5.168185000	-0.939872000	-1.105101000
C	-3.425643000	1.489164000	0.283791000
C	-4.605096000	1.777620000	-0.653470000
C	-5.831755000	0.987469000	-0.246685000
O	-5.798472000	-0.330851000	-0.330856000
C	-4.661415000	-1.054472000	-0.860418000
O	-4.922947000	3.144491000	-0.609411000

O	-6.814864000	1.538601000	0.182198000
C	-5.259771000	-2.396614000	-1.245732000
C	-4.069183000	-1.323300000	1.556237000
C	-1.408643000	0.150265000	2.283549000
O	-0.864791000	2.627587000	1.051498000
C	1.292370000	-1.428536000	1.899037000
C	4.514425000	-2.089890000	0.993941000
C	5.783891000	1.429871000	1.620773000
C	6.209968000	2.461635000	-0.591920000
C	1.497385000	0.388986000	-1.574126000
O	1.725165000	1.668433000	-1.921564000
O	1.110400000	-0.397486000	-2.397705000
H	-2.366243000	0.243649000	-1.051321000
H	-0.574758000	-0.931017000	-0.935411000
H	-1.384649000	-2.481388000	1.529460000
H	-0.710379000	-3.207004000	0.100444000
H	-3.159918000	-3.228359000	-0.014147000
H	-2.442358000	-2.291685000	-1.313144000
H	-0.573570000	1.655408000	-0.671857000
H	1.255872000	1.213307000	1.725831000
H	1.530288000	2.258604000	0.355582000
H	3.295614000	0.089246000	1.345998000
H	3.497785000	-3.148278000	-1.120508000
H	2.901199000	-1.768051000	-1.993019000
H	1.650348000	-3.245956000	0.298322000
H	0.951925000	-2.663136000	-1.190453000
H	3.626184000	2.199559000	0.220746000
H	3.961132000	1.412646000	-1.293389000
H	6.629358000	-0.600626000	0.429012000
H	7.007547000	0.174277000	-1.085195000

H	5.638298000	-1.873140000	-1.421444000
H	4.812601000	-0.453474000	-2.016408000
H	-2.770498000	2.341005000	0.151021000
H	-3.783622000	1.546413000	1.313438000
H	-4.331854000	1.515996000	-1.679348000
H	-4.325607000	-0.557280000	-1.772072000
H	-5.807995000	3.212727000	-0.227414000
H	-4.559969000	-2.988607000	-1.826293000
H	-5.558758000	-2.964623000	-0.366823000
H	-6.144014000	-2.220024000	-1.852880000
H	-4.506816000	-0.419549000	1.975275000
H	-4.858129000	-2.073194000	1.515326000
H	-3.316757000	-1.683149000	2.249962000
H	-2.317262000	0.665022000	2.586458000
H	-0.584108000	0.649542000	2.790302000
H	-1.448387000	-0.853861000	2.691205000
H	-0.630004000	2.571090000	1.981332000
H	0.728834000	-2.308665000	2.205869000
H	0.988750000	-0.609729000	2.539467000
H	2.333892000	-1.633390000	2.110314000
H	4.919065000	-1.461505000	1.785598000
H	3.752530000	-2.729906000	1.431308000
H	5.315580000	-2.746461000	0.651757000
H	5.323517000	0.625038000	2.192178000
H	6.851056000	1.424384000	1.845759000
H	5.368989000	2.374274000	1.976769000
H	6.096979000	2.375723000	-1.673541000
H	5.756135000	3.402508000	-0.276885000
H	7.276180000	2.505905000	-0.366591000
H	1.542966000	1.745436000	-2.867978000

Matopensine (*E* = -1767.5283337 Hartree), solvent: Chloroform

O	-0.000007000	-0.000010000	2.586138000
N	-1.329980000	-0.895361000	0.939685000
N	-3.835403000	2.159537000	-1.041269000
C	-2.077819000	0.338182000	1.224374000
C	-3.334599000	0.214217000	0.328651000
C	-1.137328000	1.534806000	1.040072000
C	-0.191972000	-1.157767000	1.793974000
C	-3.115766000	0.879999000	-1.070156000
C	-0.979252000	1.985244000	-0.445413000
C	-1.647119000	1.004207000	-1.424794000
C	-4.585382000	0.936560000	0.870528000
C	-3.456324000	-1.289552000	0.228809000
C	-2.248838000	-1.880170000	0.618063000
C	-5.053046000	1.813745000	-0.307209000
C	-1.674767000	3.302380000	-0.704597000
C	-3.137073000	3.267960000	-0.362522000
C	-4.511860000	-2.075839000	-0.173224000
C	-2.093511000	-3.256711000	0.642980000
C	-1.142939000	4.368111000	-1.298431000
C	-4.369161000	-3.467222000	-0.164973000
C	-3.176341000	-4.041307000	0.244487000
C	0.250942000	4.549111000	-1.811795000
H	-2.413045000	0.317662000	2.268137000
H	-1.557393000	2.365338000	1.605558000
H	-0.437119000	-1.950751000	2.505268000
H	-3.626022000	0.258390000	-1.809200000
H	0.074561000	2.061568000	-0.690338000
H	-1.537961000	1.393033000	-2.436976000

H	-1.184404000	0.019054000	-1.389177000
H	-4.322999000	1.550758000	1.732235000
H	-5.350454000	0.236708000	1.198478000
H	-5.706495000	1.239544000	-0.965912000
H	-5.587201000	2.709719000	0.002308000
H	-3.616844000	4.197983000	-0.664523000
H	-3.254257000	3.194113000	0.727240000
H	-5.448545000	-1.625288000	-0.477744000
H	-1.157835000	-3.713335000	0.940435000
H	-1.804758000	5.212000000	-1.468897000
H	-5.192985000	-4.095466000	-0.470173000
H	-3.077467000	-5.117868000	0.253980000
H	0.217457000	4.871911000	-2.852813000
H	0.777707000	5.328413000	-1.258455000
H	0.848173000	3.644130000	-1.757279000
N	1.329979000	0.895354000	0.939703000
N	3.835400000	-2.159530000	-1.041271000
C	2.077812000	-0.338194000	1.224387000
C	3.334599000	-0.214226000	0.328675000
C	1.137321000	-1.534814000	1.040065000
C	0.191962000	1.157752000	1.793984000
C	3.115771000	-0.879987000	-1.070143000
C	0.979248000	-1.985228000	-0.445426000
C	1.647126000	-1.004182000	-1.424790000
C	4.585373000	-0.936587000	0.870550000
C	3.456335000	1.289543000	0.228858000
C	2.248848000	1.880162000	0.618109000
C	5.053040000	-1.813756000	-0.307198000
C	1.674755000	-3.302366000	-0.704624000
C	3.137060000	-3.267960000	-0.362546000

C	4.511882000	2.075830000	-0.173146000
C	2.093532000	3.256705000	0.643052000
C	1.142917000	-4.368087000	-1.298467000
C	4.369194000	3.467214000	-0.164869000
C	3.176373000	4.041300000	0.244588000
C	-0.250968000	-4.549070000	-1.811828000
H	2.413029000	-0.317685000	2.268153000
H	1.557382000	-2.365355000	1.605540000
H	0.437105000	1.950730000	2.505286000
H	3.626034000	-0.258370000	-1.809175000
H	-0.074564000	-2.061542000	-0.690356000
H	1.537970000	-1.392992000	-2.436978000
H	1.184415000	-0.019027000	-1.389161000
H	4.322979000	-1.550797000	1.732245000
H	5.350448000	-0.236745000	1.198517000
H	5.706496000	-1.239549000	-0.965887000
H	5.587188000	-2.709738000	0.002309000
H	3.616827000	-4.197981000	-0.664561000
H	3.254242000	-3.194133000	0.727219000
H	5.448567000	1.625278000	-0.477663000
H	1.157857000	3.713330000	0.940507000
H	1.804727000	-5.211981000	-1.468940000
H	5.193026000	4.095458000	-0.470045000
H	3.077509000	5.117862000	0.254103000
H	-0.217488000	-4.871856000	-2.852850000
H	-0.777739000	-5.328373000	-1.258496000
H	-0.848192000	-3.644084000	-1.757299000

Naucleidinal (*E* = -1109.3963312 Hartree), solvent: Chloroform

O	-3.460213000	1.454703000	1.374937000

O	-1.252090000	-1.995018000	1.061859000
O	-6.773683000	0.044257000	-1.571501000
N	-3.004983000	-3.184473000	0.272660000
N	-5.070754000	-2.967878000	-2.766923000
C	-4.338804000	-3.249738000	-0.329106000
C	-4.557490000	-0.784456000	-0.097900000
C	-5.238588000	-2.121662000	0.142222000
C	-5.391013000	0.404903000	0.352134000
C	-4.133505000	-3.300416000	-1.817318000
C	-3.253088000	-0.794656000	0.647329000
C	-2.136847000	-4.279840000	-0.153660000
C	-2.974716000	-3.699318000	-2.412642000
C	-2.416456000	-2.019578000	0.683165000
C	-1.764880000	-4.119524000	-1.634790000
C	-4.502181000	1.658104000	0.415140000
C	-3.185984000	-3.594776000	-3.827830000
C	-2.834009000	0.267844000	1.337285000
C	-4.506328000	-3.124383000	-4.011523000
C	-6.588020000	0.630493000	-0.538016000
C	-5.218267000	2.925701000	0.820162000
C	-2.391617000	-3.847872000	-4.951228000
C	-5.040241000	-2.892837000	-5.276472000
C	-2.919151000	-3.622229000	-6.206725000
C	-4.230830000	-3.147094000	-6.366520000
H	-4.781993000	-4.194027000	-0.001369000
H	-4.376667000	-0.683193000	-1.172048000
H	-6.194967000	-2.181075000	-0.374958000
H	-5.432690000	-2.248225000	1.208521000
H	-5.776939000	0.245118000	1.366369000
H	-2.687191000	-5.208751000	-0.005588000

H	-1.255994000	-4.290930000	0.476949000
H	-1.363046000	-5.062138000	-2.008519000
H	-0.971198000	-3.375764000	-1.735124000
H	-4.022308000	1.784164000	-0.559976000
H	-5.952132000	-2.518302000	-2.584637000
H	-1.939514000	0.244430000	1.941984000
H	-7.318261000	1.377269000	-0.186369000
H	-5.905646000	3.249030000	0.041728000
H	-5.776193000	2.773546000	1.743071000
H	-4.490413000	3.717277000	0.979582000
H	-1.379414000	-4.211238000	-4.835300000
H	-6.050239000	-2.528237000	-5.400167000
H	-2.316748000	-3.811525000	-7.083648000
H	-4.614909000	-2.978187000	-7.362340000

Physalin D (*E* = -1913.8389166 Hartree), solvent: Dimethylsulfoxide

C	-6.160456000	0.472901000	-0.579570000
C	-5.535426000	-0.872714000	-0.391025000
C	-4.018498000	-0.825908000	-0.552731000
C	-3.391693000	0.343341000	0.265025000
C	-3.989399000	1.605279000	-0.367601000
C	-5.458537000	1.605102000	-0.565402000
C	-3.386699000	-2.174816000	-0.221807000
C	-1.876728000	-2.124756000	-0.365582000
C	-1.246835000	-1.022004000	0.492201000
C	-1.846726000	0.354389000	0.124237000
O	-3.326532000	2.561012000	-0.708830000
C	0.299487000	-1.057244000	0.379398000
C	1.105182000	-0.771589000	1.664806000
C	2.356881000	-0.033881000	1.225798000

C	1.771557000	0.778277000	0.066007000
C	1.209563000	2.137532000	0.580458000
C	-0.229017000	2.405490000	0.160639000
C	-1.226401000	1.517670000	0.926204000
O	0.795996000	-1.111036000	2.768838000
O	0.780817000	-0.068893000	-0.517081000
O	0.668686000	-2.325521000	-0.086032000
C	3.528004000	-0.971226000	0.850169000
C	3.153788000	-2.136373000	-0.092540000
C	1.901870000	-2.922639000	0.287880000
C	4.564792000	-0.104724000	0.139993000
C	4.012714000	0.364132000	-1.184841000
C	2.740295000	1.215234000	-1.068001000
C	2.225016000	3.147018000	0.039868000
O	3.176060000	2.533445000	-0.675647000
O	2.247999000	4.316070000	0.284482000
C	3.143456000	-1.854241000	-1.587375000
O	3.735157000	-0.745117000	-2.048570000
C	2.052748000	1.352528000	-2.418555000
O	2.711002000	-2.644400000	-2.380005000
C	4.113029000	-1.535527000	2.144199000
O	-3.696529000	-0.638790000	-1.933754000
O	-3.778883000	-2.549132000	1.089486000
C	-3.829874000	0.329784000	1.745538000
O	1.319366000	2.229551000	1.991737000
H	-7.228156000	0.506472000	-0.755145000
H	-5.938819000	-1.573405000	-1.124662000
H	-5.803275000	-1.271698000	0.587714000
H	-5.916722000	2.566292000	-0.752938000
H	-3.789420000	-2.889178000	-0.946350000

H	-1.627294000	-1.966425000	-1.414876000
H	-1.479574000	-3.097197000	-0.087124000
H	-1.464995000	-1.239996000	1.541454000
H	-1.639528000	0.518718000	-0.933058000
H	2.701682000	0.601153000	2.032716000
H	-0.352134000	2.280447000	-0.912466000
H	-0.422957000	3.456650000	0.379375000
H	-2.013120000	2.173477000	1.276876000
H	-0.758004000	1.134544000	1.834169000
H	3.978139000	-2.852560000	-0.005150000
H	1.912452000	-3.134961000	1.358029000
H	1.907809000	-3.870239000	-0.239375000
H	4.825423000	0.757984000	0.753650000
H	5.477769000	-0.674071000	-0.036236000
H	4.744873000	0.957292000	-1.727590000
H	2.783392000	1.703736000	-3.144761000
H	1.249331000	2.083634000	-2.360037000
H	1.640816000	0.408931000	-2.755474000
H	4.448345000	-0.723233000	2.787241000
H	3.381973000	-2.119773000	2.700847000
H	4.967997000	-2.173485000	1.925432000
H	-4.322870000	-0.024580000	-2.328643000
H	-3.419925000	-3.420996000	1.273738000
H	-3.538675000	1.253772000	2.238828000
H	-4.907608000	0.239053000	1.845211000
H	-3.375667000	-0.502157000	2.273032000
H	1.021057000	3.108062000	2.254242000

Strychnobaillonine (Conformer 1, *E* = -1920.223208 Hartree), solvent: Chloroform

O	1.187203000	2.835547000	1.746958000

O	-1.253732000	3.442731000	0.840803000
N	-2.377591000	1.613904000	0.112926000
N	-4.151517000	-2.415040000	-1.249426000
N	1.065715000	-0.639423000	0.391865000
N	5.064860000	-0.134792000	-1.542977000
C	-5.474105000	3.159232000	1.505271000
C	-6.374882000	2.213573000	1.033702000
C	-5.922790000	1.136049000	0.278829000
C	-4.572638000	1.022189000	0.007202000
C	-3.683163000	1.974134000	0.487084000
C	-4.112401000	3.058841000	1.240157000
C	-3.871594000	-0.052113000	-0.782962000
C	-2.383281000	0.434344000	-0.786532000
C	-1.249374000	2.324582000	0.342749000
C	0.015355000	1.568356000	-0.017471000
C	-0.176930000	0.112501000	0.467036000
C	-1.238161000	-0.562584000	-0.427005000
C	-4.480614000	-0.242386000	-2.199490000
C	-4.997953000	-1.697432000	-2.202662000
C	-4.069717000	-1.461188000	-0.140591000
C	-3.010169000	-1.795913000	0.886216000
C	-1.661592000	-1.921762000	0.180681000
C	0.153283000	-4.466489000	-0.112131000
C	-1.010098000	-4.074788000	-0.968530000
C	-1.777954000	-2.993073000	-0.871042000
C	-2.853982000	-2.710466000	-1.875251000
C	1.297878000	2.282214000	0.450553000
C	2.481069000	1.315426000	0.505657000
C	2.203325000	0.022463000	-0.259329000
C	1.591476000	-1.399461000	1.432746000

C	3.355205000	-1.001212000	-0.111760000
C	2.943381000	-1.678379000	1.175267000
C	0.980140000	-1.872851000	2.584724000
C	1.731686000	-2.664654000	3.453634000
C	3.055955000	-2.971772000	3.188514000
C	3.669503000	-2.467193000	2.038293000
C	3.849735000	1.931750000	0.182789000
C	4.900696000	0.935416000	0.671302000
C	4.756655000	-0.340587000	-0.127123000
C	4.674303000	-1.412865000	-2.145077000
C	3.451763000	-1.944144000	-1.336284000
C	4.344613000	0.988563000	-2.171266000
C	4.102116000	2.197199000	-1.283417000
C	4.120429000	3.412004000	-1.829426000
C	3.818534000	4.723572000	-1.173085000
H	-3.415131000	3.794550000	1.600380000
H	-2.983773000	-1.005854000	1.642193000
H	-5.053166000	-1.457462000	0.333586000
H	-6.029201000	-1.733524000	-1.850397000
H	-5.275692000	0.473589000	-2.391344000
H	-2.986285000	-3.555875000	-2.548508000
H	-6.621488000	0.398944000	-0.096485000
H	-7.428062000	2.316246000	1.250499000
H	-5.833151000	3.994949000	2.088663000
H	0.063445000	1.534851000	-1.111885000
H	-0.520814000	0.133971000	1.505851000
H	1.049100000	-4.577214000	-0.725046000
H	0.376839000	-3.747700000	0.671131000
H	-2.171626000	0.813366000	-1.787615000
H	-0.903737000	-2.217321000	0.897507000

H	-2.517575000	-1.862778000	-2.495530000
H	-1.235493000	-4.763341000	-1.777697000
H	-3.723882000	-0.093569000	-2.967468000
H	-4.959176000	-2.165089000	-3.184121000
H	-3.257648000	-2.728660000	1.390623000
H	-0.711055000	-0.769176000	-1.360503000
H	-0.026128000	-5.435233000	0.355818000
H	1.502338000	3.078245000	-0.275189000
H	0.419301000	3.422183000	1.730198000
H	3.019241000	5.236490000	-1.709483000
H	3.509094000	4.623401000	-0.136642000
H	4.688461000	5.380737000	-1.207575000
H	4.373833000	3.478352000	-2.883741000
H	4.914454000	1.304438000	-3.044819000
H	3.376295000	0.646763000	-2.557859000
H	4.757706000	0.729349000	1.732379000
H	5.907463000	1.329418000	0.535255000
H	3.938608000	2.858545000	0.745362000
H	2.533153000	1.018410000	1.557667000
H	1.961452000	0.211193000	-1.302803000
H	-0.049666000	-1.642395000	2.818280000
H	1.265247000	-3.040604000	4.353367000
H	3.616964000	-3.588272000	3.875099000
H	4.709034000	-2.690647000	1.834918000
H	3.624037000	-2.969757000	-1.016242000
H	5.505145000	-2.111315000	-2.052121000
H	2.526233000	-1.934116000	-1.910864000
H	4.465121000	-1.286677000	-3.204861000
H	5.467941000	-1.086738000	0.239389000

Strychnobaillonine (Conformer 2, *E* = -1920.2230913 Hartree), solvent: Chloroform

O	1.233913000	2.715778000	1.812136000
O	-1.115432000	3.436747000	0.752730000
N	-2.306836000	1.616390000	0.121065000
N	-4.629672000	-2.394839000	-0.262266000
N	1.112015000	-0.740556000	0.328727000
N	5.200536000	-0.156696000	-1.441884000
C	-5.380352000	3.609181000	0.852523000
C	-6.308443000	2.672563000	0.419490000
C	-5.882917000	1.443267000	-0.075042000
C	-4.529150000	1.173283000	-0.138545000
C	-3.610236000	2.125400000	0.292364000
C	-4.013864000	3.353637000	0.796898000
C	-3.831232000	-0.091427000	-0.578541000
C	-2.365419000	0.393782000	-0.712631000
C	-1.152837000	2.289943000	0.325345000
C	0.092812000	1.486029000	0.002710000
C	-0.120006000	0.028447000	0.462377000
C	-1.225451000	-0.608512000	-0.408085000
C	-4.366475000	-0.681920000	-1.895586000
C	-5.279168000	-1.818292000	-1.434623000
C	-4.077715000	-1.247650000	0.474358000
C	-2.811054000	-1.627645000	1.225420000
C	-1.715084000	-1.934513000	0.201346000
C	-0.035472000	-3.195715000	-2.055829000
C	-1.492456000	-3.444860000	-1.807131000
C	-2.231295000	-2.933296000	-0.826137000
C	-3.634345000	-3.426426000	-0.567981000
C	1.373828000	2.175947000	0.512992000
C	2.537864000	1.191594000	0.587507000

C	2.274148000	-0.053681000	-0.255503000
C	1.607687000	-1.589038000	1.315857000
C	3.415697000	-1.095353000	-0.148712000
C	2.962444000	-1.862824000	1.071170000
C	0.965581000	-2.138749000	2.416926000
C	1.691193000	-2.992320000	3.247922000
C	3.020276000	-3.289386000	2.994880000
C	3.663458000	-2.713379000	1.896317000
C	3.926048000	1.806290000	0.359902000
C	4.943612000	0.767324000	0.828080000
C	4.823804000	-0.450902000	-0.059166000
C	4.831637000	-1.391570000	-2.138758000
C	3.551406000	-1.941741000	-1.440530000
C	4.504814000	1.004667000	-2.026335000
C	4.238843000	2.158892000	-1.075960000
C	4.283902000	3.404731000	-1.544867000
C	3.959927000	4.674563000	-0.820285000
H	-3.293526000	4.081528000	1.125658000
H	-2.511271000	-0.803357000	1.874791000
H	-4.838179000	-0.894282000	1.171488000
H	-6.253420000	-1.425003000	-1.136995000
H	-4.869702000	0.068171000	-2.502702000
H	-3.588069000	-4.105511000	0.288383000
H	-6.606564000	0.710503000	-0.405535000
H	-7.364002000	2.897049000	0.467850000
H	-5.719695000	4.560069000	1.237680000
H	0.164150000	1.466738000	-1.091324000
H	-0.423194000	0.039941000	1.514397000
H	0.452918000	-2.715294000	-1.209870000
H	0.475837000	-4.137740000	-2.253334000

H	-2.249502000	0.732887000	-1.745297000
H	-0.859039000	-2.395842000	0.692419000
H	-3.992199000	-4.017967000	-1.408534000
H	-1.972952000	-4.149099000	-2.479435000
H	-3.538761000	-1.092803000	-2.475519000
H	-5.449606000	-2.576582000	-2.194973000
H	-3.008933000	-2.483120000	1.870220000
H	-0.740820000	-0.824538000	-1.359069000
H	0.119972000	-2.565803000	-2.935367000
H	1.610393000	2.976793000	-0.197131000
H	0.501307000	3.343924000	1.768795000
H	3.597128000	4.510817000	0.190558000
H	4.835165000	5.323524000	-0.768488000
H	3.192440000	5.229066000	-1.361695000
H	4.578199000	3.534292000	-2.582500000
H	5.102579000	1.367557000	-2.862060000
H	3.547060000	0.691126000	-2.461075000
H	4.750243000	0.493726000	1.865687000
H	5.959383000	1.156205000	0.764184000
H	4.001559000	2.695477000	0.981839000
H	2.534557000	0.842789000	1.625201000
H	2.060807000	0.198848000	-1.292021000
H	-0.066894000	-1.913672000	2.643791000
H	1.201108000	-3.424298000	4.109143000
H	3.561310000	-3.954373000	3.651646000
H	4.706465000	-2.929629000	1.703625000
H	3.667530000	-2.996897000	-1.201412000
H	5.644897000	-2.108356000	-2.031130000
H	2.662614000	-1.846468000	-2.062679000
H	4.692790000	-1.204109000	-3.201089000

H	5.509723000	-1.228626000	0.288642000

Equilibrium geometries of molecules from set **1** calculated at the **DFT(M06-2X)** level of theory with **pecG-1** basis set within the IEF-PCM model. Cartesian coordinates are given for standard orientation in form (atomic label, X, Y, Z). The X, Y, Z are given in Å.

12-28-oxaircinal (*E* = -1272.0966516 Hartree), solvent: Chloroform

O	1.586646000	-0.783051000	1.815662000
O	-0.268410000	4.709938000	1.507807000
N	1.764606000	-0.492128000	-0.467300000
N	-2.184610000	0.479016000	-1.525945000
C	0.059993000	1.175425000	-0.861054000
C	0.388322000	-0.130584000	-0.107705000
C	0.392971000	-0.098401000	1.446759000
C	-0.592630000	2.284446000	-0.001486000
C	1.446401000	1.630548000	-1.377843000
C	2.480051000	0.780788000	-0.626849000
C	-0.865922000	0.817572000	-2.026026000
C	2.176033000	-1.339285000	0.636520000
C	-2.115603000	2.161046000	0.198745000
C	0.067169000	2.375912000	1.343626000
C	0.497080000	1.294418000	1.996258000
C	-0.851265000	-0.816575000	1.995009000
C	-2.835438000	1.689932000	-1.052968000
C	3.664687000	-1.483756000	0.862405000
C	3.788771000	0.722858000	-1.359616000
C	-2.973862000	-0.309385000	-2.456054000
C	-0.802279000	-2.338943000	1.806452000
C	4.367541000	-2.297061000	-0.221575000
C	0.160067000	3.694420000	1.995779000
C	4.472374000	-0.326522000	-1.807746000

C	4.121816000	-1.782796000	-1.649759000
C	-4.277567000	-0.824078000	-1.839994000
C	-2.148156000	-2.970144000	2.044887000
C	-4.140731000	-1.348966000	-0.410382000
C	-3.116961000	-2.476848000	-0.245400000
C	-3.133126000	-3.029671000	1.151563000
H	-0.294003000	-0.919141000	-0.429508000
H	-0.423736000	3.230734000	-0.526714000
H	1.621297000	2.697721000	-1.233754000
H	1.530574000	1.417663000	-2.447643000
H	2.662944000	1.248232000	0.355909000
H	-0.908674000	1.654146000	-2.747197000
H	-0.441644000	-0.043754000	-2.551449000
H	1.736360000	-2.331968000	0.460873000
H	-2.494050000	3.131836000	0.528796000
H	-2.324905000	1.448101000	0.998662000
H	0.942418000	1.384579000	2.984700000
H	-1.720184000	-0.430969000	1.453508000
H	-0.997734000	-0.579961000	3.053121000
H	-3.878405000	1.481326000	-0.812694000
H	-2.829337000	2.472124000	-1.833299000
H	3.791501000	-1.976971000	1.828483000
H	4.111095000	-0.491237000	0.954804000
H	4.214752000	1.709405000	-1.530439000
H	-2.353795000	-1.161557000	-2.750558000
H	-3.203488000	0.249138000	-3.379821000
H	-0.052930000	-2.758081000	2.480090000
H	-0.470231000	-2.572890000	0.792708000
H	4.038422000	-3.339309000	-0.165927000
H	5.440748000	-2.296909000	-0.012506000

H	0.654437000	3.694599000	2.987189000
H	5.412551000	-0.114567000	-2.309053000
H	3.077117000	-1.945413000	-1.912149000
H	4.726344000	-2.374413000	-2.339784000
H	-4.659109000	-1.617959000	-2.489442000
H	-5.036201000	-0.036864000	-1.855678000
H	-2.327904000	-3.380286000	3.034316000
H	-5.120207000	-1.701955000	-0.072614000
H	-3.858524000	-0.529966000	0.259416000
H	-2.126849000	-2.095929000	-0.506359000
H	-3.352748000	-3.277195000	-0.957690000
H	-4.070186000	-3.491737000	1.455401000

Anabsinthin (Conformer 1, *E* = -1618.3723392 Hartree), solvent: Acetonitrile

O	-2.287435000	1.224710000	-2.189008000
O	-2.552277000	-1.932699000	0.598921000
O	2.548615000	-0.002008000	3.042902000
O	3.014662000	-1.654955000	-1.204795000
O	-3.766736000	-3.402770000	1.746138000
O	4.439608000	-2.137127000	-2.847779000
C	-1.632265000	0.190921000	-0.220023000
C	-1.960810000	1.686868000	0.065029000
C	-0.479195000	2.139737000	0.013503000
C	-1.131828000	0.557012000	-1.654045000
C	-0.531891000	-0.125455000	0.820908000
C	0.150155000	1.241653000	1.091023000
C	-2.769442000	2.126695000	-1.170004000
C	-0.059500000	1.626899000	-1.382958000

C	-2.794813000	-0.800546000	-0.263186000
C	1.677816000	1.024232000	0.980199000
C	0.596592000	-1.028408000	0.406014000
C	-4.171459000	-0.296211000	0.162339000
C	-4.289480000	1.963631000	-1.010835000
C	1.770036000	-0.389979000	0.442821000
C	-0.703982000	-0.510188000	-2.630057000
C	-4.835922000	0.544674000	-0.911804000
C	2.518198000	1.201936000	2.288956000
C	-2.525810000	3.579353000	-1.581545000
C	-4.869457000	-1.612227000	0.498566000
C	3.082210000	-1.013115000	0.091981000
C	0.362002000	-2.468065000	0.079612000
C	3.989279000	1.517663000	1.970868000
C	-3.720064000	-2.442186000	1.035078000
C	4.264800000	-0.057450000	-0.031519000
C	4.809235000	0.414699000	1.305097000
C	1.977018000	2.343691000	3.151424000
C	-6.041826000	-1.547863000	1.458619000
C	5.211032000	-0.896255000	-0.888016000
C	4.233425000	-1.638012000	-1.780696000
C	6.271810000	-0.154075000	-1.677712000
H	-2.444779000	1.898139000	1.019439000
H	-0.302463000	3.202605000	0.179555000
H	-1.024688000	-0.527343000	1.710900000
H	-0.143530000	1.623500000	2.067133000
H	0.944938000	1.205843000	-1.400206000
H	-0.114199000	2.385740000	-2.163014000
H	-2.889794000	-1.198971000	-1.278957000
H	2.085793000	1.752314000	0.268308000

H	-4.069329000	0.279447000	1.092198000
H	-4.770949000	2.457270000	-1.860005000
H	-4.582143000	2.530418000	-0.120702000
H	-1.465670000	-1.278645000	-2.769187000
H	-0.522440000	-0.042635000	-3.600096000
H	0.223441000	-0.978706000	-2.297961000
H	-4.717130000	0.036286000	-1.874070000
H	-5.910065000	0.604757000	-0.716014000
H	-2.990267000	3.759977000	-2.552265000
H	-2.985808000	4.252057000	-0.853831000
H	-1.471660000	3.833303000	-1.655000000
H	-5.180055000	-2.092405000	-0.440197000
H	3.331888000	-1.791338000	0.825411000
H	-0.360355000	-2.575025000	-0.733951000
H	1.280000000	-2.979514000	-0.203185000
H	-0.086837000	-2.968367000	0.941561000
H	4.477572000	1.769168000	2.916298000
H	4.014931000	2.421870000	1.354149000
H	3.949847000	0.805378000	-0.634372000
H	4.885505000	-0.443094000	1.980732000
H	5.824756000	0.794460000	1.161278000
H	1.031399000	2.073143000	3.621021000
H	1.817903000	3.246647000	2.555890000
H	2.687633000	2.572052000	3.946525000
H	-5.750668000	-1.047972000	2.384398000
H	-6.381886000	-2.553426000	1.706768000
H	-6.874176000	-0.999289000	1.017054000
H	1.642749000	-0.305454000	3.163069000
H	5.681444000	-1.655923000	-0.247826000
H	6.786912000	-0.838846000	-2.351658000

H	7.007818000	0.296026000	-1.011298000
H	5.817693000	0.637592000	-2.276766000

Anabsinthin (Conformer 2, *E* = -1618.3724557 Hartree), solvent: Acetonitrile

O	-2.155369000	2.325071000	-0.983521000
O	-2.590610000	-1.861261000	-0.702204000
O	1.563840000	-1.736116000	3.031683000
O	2.984897000	-0.449887000	-2.007875000
O	-3.880413000	-3.675188000	-0.686045000
O	4.370998000	0.437946000	-3.509900000
C	-1.583221000	0.281286000	-0.051858000
C	-1.868056000	1.306762000	1.084668000
C	-0.371141000	1.638678000	1.316547000
C	-1.034213000	1.424328000	-0.968265000
C	-0.522926000	-0.651292000	0.584594000
C	0.202896000	0.249457000	1.624192000
C	-2.632677000	2.439496000	0.374298000
C	0.064902000	2.063898000	-0.103013000
C	-2.775608000	-0.428841000	-0.692238000
C	1.712347000	0.059210000	1.384945000
C	0.574320000	-1.154444000	-0.318816000
C	-4.144182000	-0.234094000	-0.045958000
C	-4.160792000	2.275715000	0.401822000
C	1.767218000	-0.701494000	0.078905000
C	-0.620002000	1.161156000	-2.394745000
C	-4.755862000	1.113411000	-0.383400000
C	2.415598000	-0.708849000	2.538579000
C	-2.330590000	3.831580000	0.931417000
C	-4.892805000	-1.452563000	-0.581032000
C	3.071161000	-0.897354000	-0.634675000

C	0.301592000	-2.047799000	-1.484547000
C	3.732031000	-1.383372000	2.103884000
C	-3.784646000	-2.483255000	-0.664389000
C	4.211503000	-0.063427000	-0.054019000
C	4.748929000	-0.579719000	1.283155000
C	2.641165000	0.232626000	3.711256000
C	-6.084460000	-1.937359000	0.222060000
C	5.165842000	-0.019986000	-1.242138000
C	4.184191000	0.031620000	-2.401662000
C	6.175187000	1.110861000	-1.282210000
H	-2.366271000	0.913087000	1.971868000
H	-0.165667000	2.373691000	2.095023000
H	-1.049669000	-1.487512000	1.052414000
H	-0.064803000	-0.043890000	2.639220000
H	1.055033000	1.697516000	-0.371556000
H	0.054564000	3.141603000	-0.262322000
H	-2.858420000	-0.119649000	-1.739649000
H	2.209114000	1.032574000	1.305363000
H	-4.043842000	-0.348403000	1.041577000
H	-4.604247000	3.203654000	0.028949000
H	-4.456705000	2.195371000	1.453149000
H	-0.418126000	2.118888000	-2.879054000
H	0.292843000	0.564437000	-2.423431000
H	-1.398809000	0.656567000	-2.968305000
H	-4.631963000	1.292247000	-1.456536000
H	-5.831699000	1.084832000	-0.189399000
H	-2.787133000	3.943210000	1.917760000
H	-1.266793000	4.032549000	1.028293000
H	-2.762861000	4.583961000	0.269739000
H	-5.197285000	-1.247726000	-1.617205000

H	3.354548000	-1.957212000	-0.665726000
H	-0.147278000	-2.981786000	-1.137324000
H	-0.427390000	-1.600305000	-2.164487000
H	1.209607000	-2.266867000	-2.045129000
H	3.458859000	-2.277074000	1.532067000
H	4.218879000	-1.753342000	3.010036000
H	3.822929000	0.953186000	0.070783000
H	5.601598000	-1.238312000	1.090729000
H	5.138670000	0.258496000	1.866332000
H	2.982584000	-0.331069000	4.580853000
H	1.715972000	0.749609000	3.974506000
H	3.393065000	0.983152000	3.461140000
H	-6.468327000	-2.868520000	-0.195010000
H	-6.884503000	-1.196685000	0.211896000
H	-5.797640000	-2.121222000	1.259242000
H	1.362309000	-2.324134000	2.294143000
H	5.681986000	-0.987051000	-1.317536000
H	6.906216000	1.003252000	-0.480313000
H	5.675044000	2.074385000	-1.167149000
H	6.702675000	1.112542000	-2.236197000

Betulinic acid (*E* = -1397.5327811 Hartree), solvent: Pyridine

C	4.187157000	1.199427000	0.359316000
C	3.158076000	0.290787000	-0.346624000
C	3.785709000	-1.111391000	-0.365839000
C	5.246327000	-0.780896000	-0.720141000
C	5.550660000	0.465069000	0.131810000
C	1.718927000	0.258896000	0.157002000
C	0.840829000	-0.696052000	-0.708153000
C	1.538244000	-2.068813000	-0.905046000

C	3.007764000	-1.985257000	-1.343066000
C	1.077830000	1.641329000	0.256260000
C	-0.362639000	1.584334000	0.778073000
C	-1.245226000	0.536421000	0.080822000
C	-0.548813000	-0.859017000	0.043683000
C	-2.735356000	0.543835000	0.569563000
C	-3.498884000	-0.448122000	-0.357262000
C	-2.904089000	-1.852651000	-0.253511000
C	-1.443950000	-1.865846000	-0.701962000
C	-3.307848000	1.957278000	0.327291000
C	-4.827829000	2.022475000	0.422113000
C	-5.495508000	1.047602000	-0.527392000
C	-5.050484000	-0.413807000	-0.293666000
C	4.236473000	2.624076000	-0.141051000
C	4.244190000	3.649969000	0.705452000
C	4.329950000	2.835510000	-1.627867000
C	3.821484000	-1.750194000	1.016782000
O	3.751248000	-1.176624000	2.072603000
O	4.014275000	-3.075866000	0.949386000
C	0.663458000	-0.113408000	-2.126922000
C	-0.325891000	-1.407447000	1.471965000
C	-2.874558000	0.230773000	2.072641000
C	-5.620870000	-1.253453000	-1.446465000
C	-5.648300000	-0.958211000	1.007014000
O	-6.892822000	1.219289000	-0.372286000
H	3.970310000	1.219686000	1.428401000
H	3.149752000	0.591233000	-1.400761000
H	5.284844000	-0.542442000	-1.787099000
H	5.932905000	-1.609457000	-0.536862000
H	6.288668000	1.112218000	-0.344285000

H	5.961131000	0.169269000	1.098154000
H	1.761634000	-0.151992000	1.169743000
H	0.979887000	-2.644262000	-1.648028000
H	1.511024000	-2.658291000	0.014597000
H	3.100799000	-1.545141000	-2.339381000
H	3.431055000	-2.989339000	-1.397921000
H	1.108346000	2.137111000	-0.720203000
H	1.673149000	2.268567000	0.924027000
H	-0.809986000	2.573387000	0.665236000
H	-0.326621000	1.393278000	1.854006000
H	-1.336045000	0.850872000	-0.964191000
H	-3.266403000	-0.097722000	-1.376773000
H	-3.457894000	-2.547751000	-0.886946000
H	-3.001774000	-2.236431000	0.765603000
H	-1.432669000	-1.651919000	-1.774100000
H	-1.032128000	-2.872238000	-0.578517000
H	-3.002649000	2.296335000	-0.670940000
H	-2.877256000	2.661488000	1.043161000
H	-5.173389000	3.030795000	0.181622000
H	-5.173848000	1.816624000	1.439518000
H	-5.196407000	1.314896000	-1.553597000
H	4.327423000	4.672557000	0.355998000
H	4.163796000	3.494226000	1.775404000
H	3.369675000	2.638556000	-2.113211000
H	4.620658000	3.858886000	-1.862914000
H	5.057058000	2.154807000	-2.081502000
H	4.075974000	-3.399156000	1.858486000
H	0.212739000	0.875314000	-2.144318000
H	1.625865000	-0.024921000	-2.630973000
H	0.050167000	-0.767946000	-2.746394000

H	-1.217547000	-1.914912000	1.834685000
H	0.482317000	-2.137466000	1.503851000
H	-0.082829000	-0.633263000	2.197267000
H	-2.004354000	0.582367000	2.626426000
H	-3.737867000	0.739107000	2.500611000
H	-2.990745000	-0.831040000	2.284196000
H	-5.158536000	-0.984110000	-2.400223000
H	-5.473297000	-2.321196000	-1.284053000
H	-6.699631000	-1.101461000	-1.532580000
H	-6.736468000	-0.959531000	0.927713000
H	-5.317441000	-1.984770000	1.180601000
H	-5.384335000	-0.367769000	1.881316000
H	-7.331668000	0.849284000	-1.141368000

Icajine (Conformer 1, *E* = -1187.7402646 Hartree), solvent: Chloroform

O	-0.133851000	-2.880386000	1.199814000
O	2.067497000	3.004776000	0.754451000
O	-2.525293000	3.190480000	-0.439598000
N	-1.436303000	1.188212000	-0.538519000
N	2.093196000	-2.072415000	-0.567307000
C	-0.148011000	0.510293000	-0.327363000
C	0.205472000	-1.744807000	0.959532000
C	1.368258000	-1.730737000	-1.779616000
C	-0.137116000	-1.761622000	-1.506531000
C	-0.486565000	-0.999227000	-0.209179000
C	-1.993425000	-0.972463000	-0.018682000
C	-2.854411000	-2.009892000	0.288376000
C	-4.222996000	-1.754239000	0.347842000
C	-4.709698000	-0.478010000	0.094463000

C	-3.854659000	0.575243000	-0.218808000
C	-2.494276000	0.305991000	-0.264776000
C	1.201299000	-1.032962000	1.861880000
C	1.734166000	0.294929000	1.312916000
C	0.491872000	1.099462000	0.921914000
C	0.710571000	2.623129000	0.778065000
C	2.841097000	2.665546000	-0.368062000
C	3.160659000	1.211672000	-0.546618000
C	2.727955000	0.167009000	0.155874000
C	3.210463000	-1.214543000	-0.216912000
C	-0.114313000	3.190423000	-0.395025000
C	-1.492119000	2.559195000	-0.458385000
C	2.334103000	-3.488095000	-0.411491000
H	0.489799000	0.692456000	-1.192927000
H	1.619990000	-2.411898000	-2.602795000
H	1.672789000	-0.732438000	-2.099774000
H	-0.482558000	-2.791094000	-1.377668000
H	-0.691716000	-1.331976000	-2.345708000
H	-2.455667000	-2.995467000	0.488362000
H	-4.909648000	-2.555119000	0.589681000
H	-5.775484000	-0.292291000	0.140919000
H	-4.222345000	1.570637000	-0.410734000
H	1.986953000	-1.752646000	2.096782000
H	0.683280000	-0.825239000	2.803986000
H	2.242275000	0.820479000	2.125895000
H	-0.248222000	0.965962000	1.721418000
H	0.343989000	3.093001000	1.692219000
H	3.781187000	3.210868000	-0.243980000
H	2.399231000	3.053302000	-1.296302000
H	3.853962000	1.024112000	-1.364150000

H	3.707857000	-1.673881000	0.644134000
H	3.962569000	-1.145019000	-1.016681000
H	-0.230078000	4.268952000	-0.312061000
H	0.383930000	2.972309000	-1.343890000
H	3.075237000	-3.872238000	-1.126313000
H	1.395666000	-4.028714000	-0.547062000
H	2.681976000	-3.691363000	0.603620000

Icajine (Conformer 2, *E* = -1187.7414855 Hartree), solvent: Chloroform

O	-0.002839000	-2.788232000	1.296276000
O	1.533947000	2.924375000	-0.579588000
O	-2.897398000	2.976141000	-0.256884000
N	-1.540598000	1.164085000	-0.376874000
N	2.220098000	-1.986913000	-0.487823000
C	-0.189680000	0.577590000	-0.276835000
C	0.295072000	-1.650425000	1.017682000
C	1.517709000	-1.617246000	-1.704110000
C	0.014220000	-1.698000000	-1.463921000
C	-0.412728000	-0.960418000	-0.172229000
C	-1.919679000	-1.073622000	-0.052928000
C	-2.689501000	-2.205621000	0.142910000
C	-4.077265000	-2.087491000	0.130494000
C	-4.670123000	-0.851097000	-0.089696000
C	-3.906054000	0.294842000	-0.292433000
C	-2.523775000	0.163984000	-0.259819000
C	1.261536000	-0.871514000	1.896969000
C	1.790952000	0.434028000	1.287336000
C	0.539134000	1.229459000	0.888129000
C	0.727648000	2.710995000	0.556549000
C	2.913937000	2.715750000	-0.359626000

C	3.331711000	1.279808000	-0.506720000
C	2.835052000	0.256592000	0.185563000
C	3.329630000	-1.136332000	-0.111219000
C	-0.622667000	3.369592000	0.269916000
C	-1.787327000	2.495817000	-0.165474000
C	2.455867000	-3.403981000	-0.345914000
H	0.360369000	0.814874000	-1.190953000
H	1.796668000	-0.596855000	-1.973499000
H	1.805688000	-2.256769000	-2.549093000
H	-0.297200000	-2.740500000	-1.351900000
H	-0.537518000	-1.284304000	-2.312760000
H	-2.207541000	-3.158396000	0.315847000
H	-4.693869000	-2.963373000	0.286387000
H	-5.749820000	-0.769626000	-0.107239000
H	-4.360513000	1.256793000	-0.460554000
H	0.719659000	-0.619589000	2.814788000
H	2.052717000	-1.563004000	2.189686000
H	2.287013000	0.991384000	2.092468000
H	-0.133215000	1.198210000	1.756982000
H	1.192630000	3.210440000	1.417885000
H	3.191085000	3.098785000	0.634190000
H	3.433368000	3.325475000	-1.098684000
H	4.087991000	1.070410000	-1.257123000
H	3.779040000	-1.569777000	0.789027000
H	4.121060000	-1.095889000	-0.874659000
H	-0.991242000	3.869848000	1.167595000
H	-0.479501000	4.149189000	-0.479942000
H	3.203096000	-3.782539000	-1.057657000
H	1.518242000	-3.942295000	-0.494631000
H	2.793588000	-3.617228000	0.670551000

Iguesterin (*E* = -1238.8568142 Hartree), solvent: Chloroform

O	-6.523759000	0.810289000	-0.975917000
O	-5.272224000	2.635809000	0.403772000
C	1.664024000	0.215760000	0.430589000
C	1.049777000	-1.224643000	0.557979000
C	3.206239000	0.148988000	0.640623000
C	3.969564000	-0.899527000	-0.223335000
C	1.731521000	-2.151834000	-0.466035000
C	1.014252000	1.081944000	1.525048000
C	3.239626000	-2.249453000	-0.251732000
C	-0.455420000	-1.117736000	0.310144000
C	-1.276974000	-0.070401000	1.060204000
C	-0.493290000	1.236252000	1.334179000
C	3.907827000	1.515300000	0.531780000
C	1.303928000	0.845790000	-0.927497000
C	4.198478000	-0.439600000	-1.677207000
C	1.280396000	-1.860962000	1.950072000
C	5.351688000	-1.119364000	0.407934000
C	4.388262000	1.901883000	-0.841614000
C	-2.609842000	0.248080000	0.384860000
C	4.518636000	1.014601000	-1.822662000
C	-1.097718000	-1.960061000	-0.518886000
C	-1.716466000	-0.697300000	2.422319000
C	-3.249143000	-0.772640000	-0.438945000
C	-2.498152000	-1.835824000	-0.823526000
C	4.729847000	3.352043000	-1.016675000
C	-3.273698000	1.393296000	0.660469000
C	-4.621444000	-0.606081000	-0.895237000
C	-4.625729000	1.616035000	0.186865000

C	-5.264137000	0.540144000	-0.593235000
C	-5.293643000	-1.672902000	-1.708945000
H	3.336579000	-0.172861000	1.680748000
H	1.313410000	-3.158558000	-0.385452000
H	1.511712000	-1.804893000	-1.479957000
H	1.247655000	0.680513000	2.513266000
H	1.434634000	2.088765000	1.507868000
H	3.676501000	-2.859049000	-1.050380000
H	3.446196000	-2.793263000	0.673304000
H	-0.667902000	1.922181000	0.503242000
H	-0.915767000	1.719653000	2.218384000
H	3.259983000	2.311838000	0.905462000
H	4.773571000	1.530440000	1.205501000
H	0.232027000	0.791739000	-1.125170000
H	1.587765000	1.901268000	-0.934389000
H	1.806028000	0.370404000	-1.763781000
H	5.026901000	-1.033528000	-2.083000000
H	3.342013000	-0.698794000	-2.309464000
H	2.334273000	-2.059883000	2.130562000
H	0.934219000	-1.249569000	2.775070000
H	0.748949000	-2.814373000	1.995590000
H	5.262194000	-1.361241000	1.470341000
H	5.984391000	-0.235370000	0.305624000
H	5.859768000	-1.951419000	-0.086240000
H	4.875993000	1.348466000	-2.793252000
H	-0.566081000	-2.755442000	-1.020606000
H	-0.872266000	-0.826032000	3.093399000
H	-2.193394000	-1.665949000	2.264996000
H	-2.432247000	-0.025530000	2.897905000
H	-2.939860000	-2.601333000	-1.450814000

H	5.461554000	3.672229000	-0.268507000
H	3.842513000	3.976823000	-0.872964000
H	5.138201000	3.554096000	-2.006886000
H	-2.850177000	2.185432000	1.263573000
H	-5.280923000	-2.633762000	-1.189582000
H	-4.794647000	-1.813153000	-2.671004000
H	-6.328660000	-1.398738000	-1.900544000
H	-6.697700000	1.699371000	-0.620883000

Itoaic acid (Conformer 1, *E* = -1623.1685568 Hartree), solvent: Chloroform

C	-3.412805000	-1.106596000	-0.275566000
C	-2.561927000	0.162644000	0.063338000
C	-1.256483000	-0.109592000	0.903112000
C	-0.418945000	-1.136326000	0.062408000
C	-1.213639000	-2.426843000	-0.126919000
C	-2.490497000	-2.159090000	-0.910597000
C	-0.431621000	1.207184000	1.007451000
C	1.055766000	1.002831000	1.319333000
C	1.783426000	0.044446000	0.362047000
C	1.066174000	-1.347627000	0.480190000
C	3.300202000	-0.072325000	0.758861000
C	4.092956000	-1.287954000	0.178961000
C	3.238474000	-2.557677000	-0.048764000
C	1.774434000	-2.361537000	-0.439151000
C	4.131266000	1.243750000	0.662977000
C	5.077525000	1.523446000	-0.538316000
C	5.762572000	0.236072000	-1.011466000
C	4.787459000	-0.924284000	-1.147027000
C	-3.394377000	1.328628000	0.612264000
C	-4.451852000	1.916735000	-0.331996000

C	-5.713575000	1.075456000	-0.309543000
O	-5.664412000	-0.176701000	-0.742245000
C	-4.478563000	-0.732375000	-1.345362000
O	-4.777681000	3.213987000	0.078924000
O	-6.739960000	1.511764000	0.144131000
C	-5.023045000	-1.889644000	-2.169123000
C	-4.142479000	-1.721028000	0.926630000
C	-1.600289000	-0.571832000	2.334352000
O	-0.941437000	2.136360000	1.948004000
C	1.161979000	-1.924298000	1.906062000
C	5.167825000	-1.664405000	1.213676000
C	6.143242000	2.509584000	-0.046791000
C	4.389636000	2.176295000	-1.744218000
C	1.686538000	0.622477000	-1.053591000
O	1.537945000	1.957671000	-1.045617000
O	1.763913000	0.030006000	-2.099411000
H	-2.180438000	0.505176000	-0.911275000
H	-0.361544000	-0.696483000	-0.943278000
H	-1.440736000	-2.902228000	0.831033000
H	-0.630517000	-3.151348000	-0.693910000
H	-3.041865000	-3.093255000	-1.042335000
H	-2.201760000	-1.817923000	-1.913022000
H	-0.515057000	1.719719000	0.045058000
H	1.160456000	0.622515000	2.339238000
H	1.506310000	1.993864000	1.317256000
H	3.227390000	-0.257077000	1.835576000
H	3.270753000	-3.171450000	0.853922000
H	3.730696000	-3.159902000	-0.818316000
H	1.292170000	-3.335793000	-0.341058000
H	1.680119000	-2.054157000	-1.478069000

H	4.768928000	1.250886000	1.551211000
H	3.471648000	2.107511000	0.780438000
H	6.562434000	-0.035414000	-0.315929000
H	6.252384000	0.429755000	-1.971553000
H	5.328557000	-1.810313000	-1.493760000
H	4.031004000	-0.707053000	-1.906375000
H	-2.738505000	2.163385000	0.839520000
H	-3.883559000	1.076459000	1.558443000
H	-4.050709000	1.955862000	-1.352580000
H	-4.050435000	0.001146000	-2.035570000
H	-5.695840000	3.168029000	0.380204000
H	-4.265088000	-2.295844000	-2.835143000
H	-5.398392000	-2.691027000	-1.531260000
H	-5.851680000	-1.522961000	-2.774050000
H	-4.654142000	-0.972746000	1.532274000
H	-4.902100000	-2.424170000	0.579399000
H	-3.462231000	-2.272252000	1.571993000
H	-2.559188000	-0.168377000	2.658194000
H	-0.860628000	-0.231117000	3.061153000
H	-1.645405000	-1.653654000	2.435644000
H	-0.857001000	1.758111000	2.827876000
H	0.640037000	-2.884581000	1.930260000
H	0.724786000	-1.297363000	2.672501000
H	2.191214000	-2.116207000	2.200936000
H	5.803012000	-0.822903000	1.491995000
H	4.698698000	-2.037497000	2.127465000
H	5.812655000	-2.455742000	0.820437000
H	6.854132000	2.735571000	-0.845694000
H	5.687675000	3.449852000	0.276457000
H	6.701276000	2.094518000	0.796186000

H	5.155477000	2.504272000	-2.451811000
H	3.735605000	1.486004000	-2.277348000
H	3.811406000	3.051492000	-1.442410000
H	1.544782000	2.243317000	-1.969592000

Itoaic acid (Conformer 2, *E* = -1623.1675447 Hartree), solvent: Chloroform

C	-3.486541000	-1.100130000	0.143832000
C	-2.636567000	0.210185000	0.014347000
C	-1.239349000	0.179910000	0.746663000
C	-0.488354000	-1.058906000	0.151543000
C	-1.265193000	-2.336740000	0.459312000
C	-2.613228000	-2.307370000	-0.243743000
C	-0.418473000	1.448025000	0.371574000
C	1.082507000	1.319885000	0.646113000
C	1.743935000	0.128362000	-0.062718000
C	1.040090000	-1.178378000	0.421213000
C	3.277584000	0.064000000	0.269779000
C	3.981849000	-1.278483000	-0.174131000
C	3.027806000	-2.200991000	-0.946737000
C	1.628304000	-2.401847000	-0.342591000
C	4.049146000	1.302369000	-0.216372000
C	5.554591000	1.288393000	0.094032000
C	6.187492000	-0.033576000	-0.426009000
C	5.165142000	-0.943230000	-1.099844000
C	-3.438517000	1.484900000	0.303427000
C	-4.609679000	1.787592000	-0.641377000
C	-5.836724000	0.984484000	-0.253366000
O	-5.798655000	-0.337958000	-0.335319000
C	-4.662185000	-1.042038000	-0.874576000
O	-4.930648000	3.147698000	-0.566442000

O	-6.822321000	1.528339000	0.173533000
C	-5.253756000	-2.384423000	-1.277063000
C	-4.080536000	-1.328839000	1.541321000
C	-1.410079000	0.168978000	2.279277000
O	-0.863187000	2.639165000	0.996216000
C	1.284193000	-1.417820000	1.923495000
C	4.542613000	-2.064088000	1.028814000
C	5.799713000	1.456339000	1.595729000
C	6.190745000	2.478178000	-0.629364000
C	1.508274000	0.353237000	-1.570006000
O	1.709325000	1.632785000	-1.930244000
O	1.146607000	-0.454863000	-2.383857000
H	-2.372766000	0.260935000	-1.053447000
H	-0.564478000	-0.933974000	-0.933857000
H	-1.400884000	-2.477338000	1.534340000
H	-0.711981000	-3.210778000	0.111204000
H	-3.161679000	-3.229520000	-0.036028000
H	-2.429929000	-2.282708000	-1.325348000
H	-0.560191000	1.631619000	-0.700999000
H	1.254123000	1.235473000	1.722649000
H	1.530777000	2.261098000	0.334194000
H	3.302262000	0.098958000	1.363528000
H	3.513545000	-3.177361000	-1.045955000
H	2.918870000	-1.819123000	-1.960690000
H	1.641403000	-3.254290000	0.342826000
H	0.962868000	-2.676018000	-1.162566000
H	3.616505000	2.203278000	0.227472000
H	3.932197000	1.418207000	-1.294790000
H	6.656444000	-0.571983000	0.402964000
H	6.992956000	0.192352000	-1.129715000

H	5.640029000	-1.878143000	-1.413452000
H	4.790029000	-0.470319000	-2.013857000
H	-2.784702000	2.346243000	0.210588000
H	-3.814889000	1.511667000	1.330729000
H	-4.321498000	1.544660000	-1.671997000
H	-4.324282000	-0.533912000	-1.783435000
H	-5.811118000	3.184412000	-0.167426000
H	-4.554700000	-2.965188000	-1.874380000
H	-5.541284000	-2.969335000	-0.402508000
H	-6.148109000	-2.202056000	-1.871925000
H	-4.528123000	-0.426625000	1.958564000
H	-4.868531000	-2.083048000	1.491266000
H	-3.332093000	-1.688990000	2.243706000
H	-2.314277000	0.700833000	2.575956000
H	-0.577721000	0.660913000	2.786591000
H	-1.466788000	-0.834051000	2.694713000
H	-0.743454000	2.545518000	1.945935000
H	0.712232000	-2.292300000	2.240524000
H	0.987860000	-0.587411000	2.556239000
H	2.326464000	-1.630021000	2.136555000
H	4.975741000	-1.417220000	1.793173000
H	3.780166000	-2.680153000	1.504759000
H	5.327554000	-2.744938000	0.688130000
H	5.341819000	0.656383000	2.180342000
H	6.871577000	1.449685000	1.809335000
H	5.390363000	2.406287000	1.950504000
H	6.063975000	2.384946000	-1.711172000
H	5.734254000	3.420071000	-0.313023000
H	7.261773000	2.530964000	-0.417617000
H	1.529921000	1.683105000	-2.879120000

Matopensine (E = -1767.5283337 Hartree), solvent: Chloroform

O	-0.000020000	-0.000027000	2.593562000
N	-1.348718000	-0.858927000	0.940516000
N	-3.759854000	2.238373000	-1.064225000
C	-2.065737000	0.392088000	1.233656000
C	-3.326725000	0.299063000	0.341527000
C	-1.092208000	1.560354000	1.039632000
C	-0.221829000	-1.149691000	1.801365000
C	-3.082045000	0.934588000	-1.066937000
C	-0.912490000	1.978891000	-0.453315000
C	-1.607953000	1.002167000	-1.418662000
C	-4.549561000	1.074529000	0.872650000
C	-3.495935000	-1.202263000	0.258860000
C	-2.300111000	-1.822897000	0.639415000
C	-4.987691000	1.946559000	-0.321435000
C	-1.560044000	3.313989000	-0.741655000
C	-3.025353000	3.337426000	-0.407423000
C	-4.577721000	-1.963291000	-0.127780000
C	-2.178070000	-3.203562000	0.666066000
C	-0.986765000	4.348909000	-1.354067000
C	-4.470658000	-3.358299000	-0.112764000
C	-3.287169000	-3.962019000	0.284450000
C	0.416878000	4.468745000	-1.860557000
H	-2.393696000	0.378816000	2.282440000
H	-1.492565000	2.414409000	1.590728000
H	-0.488663000	-1.936994000	2.515207000
H	-3.611302000	0.317822000	-1.801190000
H	0.147425000	2.012969000	-0.692671000
H	-1.481025000	1.369038000	-2.439545000

H	-1.179530000	0.000695000	-1.356246000
H	-4.262411000	1.696280000	1.724308000
H	-5.340201000	0.407912000	1.217252000
H	-5.661628000	1.382738000	-0.972859000
H	-5.493768000	2.867366000	-0.029229000
H	-3.472783000	4.278128000	-0.735915000
H	-3.150716000	3.294609000	0.686291000
H	-5.507007000	-1.489018000	-0.426534000
H	-1.250330000	-3.684859000	0.958391000
H	-1.618000000	5.213830000	-1.548111000
H	-5.316020000	-3.967363000	-0.404530000
H	-3.216349000	-5.042772000	0.299896000
H	0.402616000	4.755391000	-2.915044000
H	0.963720000	5.253335000	-1.329897000
H	0.987360000	3.546741000	-1.767963000
N	1.348673000	0.858903000	0.940527000
N	3.759912000	-2.238361000	-1.064156000
C	2.065705000	-0.392103000	1.233664000
C	3.326710000	-0.299047000	0.341560000
C	1.092189000	-1.560379000	1.039613000
C	0.221793000	1.149652000	1.801389000
C	3.082080000	-0.934589000	-1.066903000
C	0.912519000	-1.978922000	-0.453344000
C	1.607999000	-1.002188000	-1.418670000
C	4.549555000	-1.074475000	0.872720000
C	3.495882000	1.202282000	0.258870000
C	2.300037000	1.822891000	0.639396000
C	4.987725000	-1.946521000	-0.321338000
C	1.560113000	-3.314006000	-0.741674000
C	3.025404000	-3.337417000	-0.407367000

C	4.577647000	1.963332000	-0.127784000
C	2.177948000	3.203554000	0.665992000
C	0.986910000	-4.348918000	-1.354171000
C	4.470538000	3.358338000	-0.112822000
C	3.287024000	3.962034000	0.284352000
C	-0.416672000	-4.468771000	-1.860822000
H	2.393648000	-0.378835000	2.282453000
H	1.492548000	-2.414430000	1.590714000
H	0.488627000	1.936945000	2.515242000
H	3.611349000	-0.317821000	-1.801146000
H	-0.147386000	-2.013016000	-0.692737000
H	1.481106000	-1.369062000	-2.439556000
H	1.179560000	-0.000722000	-1.356267000
H	4.262402000	-1.696215000	1.724385000
H	5.340174000	-0.407834000	1.217324000
H	5.661672000	-1.382702000	-0.972754000
H	5.493807000	-2.867316000	-0.029104000
H	3.472865000	-4.278114000	-0.735834000
H	3.150716000	-3.294588000	0.686352000
H	5.506950000	1.489079000	-0.426520000
H	1.250189000	3.684831000	0.958288000
H	1.618188000	-5.213814000	-1.548191000
H	5.315882000	3.967418000	-0.404607000
H	3.216165000	5.042785000	0.299751000
H	-0.402283000	-4.755386000	-2.915316000
H	-0.963556000	-5.253389000	-1.330248000
H	-0.987186000	-3.546783000	-1.768268000

Naucleidinal (*E* = -1109.3963312 Hartree), solvent = Chloroform

O	-3.448323000	1.441577000	1.254096000

O	-1.220062000	-2.009163000	0.957564000
O	-6.790952000	-0.185873000	-1.575753000
N	-3.013975000	-3.247282000	0.342502000
N	-5.090906000	-2.951243000	-2.678983000
C	-4.347939000	-3.312041000	-0.256471000
C	-4.568713000	-0.854504000	-0.103109000
C	-5.240098000	-2.179890000	0.220139000
C	-5.394484000	0.357514000	0.293586000
C	-4.152415000	-3.331962000	-1.745446000
C	-3.242588000	-0.831539000	0.600447000
C	-2.145076000	-4.332224000	-0.106425000
C	-2.995062000	-3.713744000	-2.355590000
C	-2.398313000	-2.050127000	0.650149000
C	-1.784038000	-4.156235000	-1.590027000
C	-4.497027000	1.606270000	0.297507000
C	-3.202370000	-3.540895000	-3.766214000
C	-2.811779000	0.261061000	1.237742000
C	-4.516713000	-3.044369000	-3.928513000
C	-6.588798000	0.517503000	-0.620816000
C	-5.202407000	2.893192000	0.664036000
C	-2.407362000	-3.747892000	-4.899441000
C	-5.041379000	-2.736696000	-5.181072000
C	-2.926988000	-3.446922000	-6.143699000
C	-4.230347000	-2.942204000	-6.281697000
H	-4.791720000	-4.260075000	0.067101000
H	-4.414981000	-0.807436000	-1.187867000
H	-6.219402000	-2.261351000	-0.254229000
H	-5.384523000	-2.269661000	1.300311000
H	-5.780711000	0.246052000	1.317472000
H	-2.689652000	-5.267410000	0.040923000

H	-1.256467000	-4.341485000	0.518471000
H	-1.384920000	-5.095866000	-1.981429000
H	-0.986740000	-3.410824000	-1.681869000
H	-4.027998000	1.693021000	-0.690987000
H	-5.907608000	-2.396553000	-2.472430000
H	-1.895717000	0.262467000	1.814758000
H	-7.297990000	1.325119000	-0.364317000
H	-5.904490000	3.190524000	-0.114812000
H	-5.743766000	2.778884000	1.604766000
H	-4.466900000	3.688441000	0.780330000
H	-1.399683000	-4.134509000	-4.800208000
H	-6.047076000	-2.349797000	-5.288743000
H	-2.323440000	-3.600526000	-7.029194000
H	-4.608301000	-2.711828000	-7.269658000

Physalin D (*E* = -1913.3823211 Hartree), solvent: Dimethylsulfoxide

C	-6.124214000	0.511988000	-0.640103000
C	-5.522079000	-0.840478000	-0.412572000
C	-3.999268000	-0.814578000	-0.540263000
C	-3.378134000	0.356736000	0.277652000
C	-3.936043000	1.617765000	-0.395346000
C	-5.402780000	1.634396000	-0.633256000
C	-3.394446000	-2.164016000	-0.166419000
C	-1.882465000	-2.136497000	-0.302860000
C	-1.239937000	-1.023970000	0.535482000
C	-1.832539000	0.351956000	0.155544000
O	-3.249907000	2.553593000	-0.739455000
C	0.304865000	-1.069453000	0.396522000
C	1.130189000	-0.794160000	1.669541000
C	2.377127000	-0.053570000	1.213601000

C	1.769167000	0.760459000	0.068637000
C	1.218271000	2.111176000	0.606167000
C	-0.216303000	2.402800000	0.183056000
C	-1.218097000	1.523497000	0.950799000
O	0.840484000	-1.152685000	2.773056000
O	0.775164000	-0.083303000	-0.509311000
O	0.649597000	-2.337214000	-0.083346000
C	3.532617000	-0.990265000	0.807345000
C	3.124788000	-2.134943000	-0.146099000
C	1.888250000	-2.933904000	0.259954000
C	4.563091000	-0.125430000	0.082638000
C	3.986266000	0.356890000	-1.228888000
C	2.723142000	1.216499000	-1.071803000
C	2.248255000	3.118877000	0.095429000
O	3.178194000	2.519451000	-0.662914000
O	2.293976000	4.272991000	0.397068000
C	3.036878000	-1.813810000	-1.631418000
O	3.680255000	-0.731402000	-2.101230000
C	2.014573000	1.378256000	-2.409805000
O	2.510783000	-2.553620000	-2.411191000
C	4.132086000	-1.573375000	2.086980000
O	-3.635234000	-0.654903000	-1.908337000
O	-3.798498000	-2.482633000	1.150181000
C	-3.846990000	0.367505000	1.747969000
O	1.312690000	2.171811000	2.017903000
H	-7.188731000	0.558278000	-0.844619000
H	-5.914290000	-1.545596000	-1.152294000
H	-5.814713000	-1.228930000	0.566671000
H	-5.841365000	2.600573000	-0.853291000
H	-3.801184000	-2.894118000	-0.878754000

H	-1.621321000	-2.004486000	-1.355794000
H	-1.493196000	-3.107672000	0.001942000
H	-1.447337000	-1.233084000	1.592357000
H	-1.618987000	0.504414000	-0.905537000
H	2.734173000	0.583984000	2.017514000
H	-0.347563000	2.280792000	-0.892129000
H	-0.393856000	3.458999000	0.406321000
H	-2.011718000	2.182889000	1.287083000
H	-0.749334000	1.151807000	1.866453000
H	3.959418000	-2.848139000	-0.108079000
H	1.924505000	-3.163486000	1.329160000
H	1.887915000	-3.873528000	-0.287102000
H	4.835122000	0.737942000	0.695524000
H	5.472374000	-0.699137000	-0.114595000
H	4.713446000	0.956865000	-1.777401000
H	2.740128000	1.719779000	-3.149325000
H	1.227677000	2.128197000	-2.330549000
H	1.577128000	0.442273000	-2.744612000
H	4.516702000	-0.768033000	2.715557000
H	3.387639000	-2.119687000	2.669224000
H	4.956213000	-2.249164000	1.851941000
H	-4.247615000	-0.037706000	-2.321856000
H	-3.454829000	-3.355285000	1.360309000
H	-3.518814000	1.277172000	2.249858000
H	-4.933546000	0.335088000	1.818014000
H	-3.452147000	-0.492114000	2.283320000
H	1.120445000	3.082325000	2.274395000

Strychnobaillonine (Conformer 1, *E* = -1919.8600944 Hartree), solvent: Chloroform

O	1.229673000	2.924331000	1.563692000

O	-1.215216000	3.477779000	0.676926000
N	-2.365945000	1.617189000	0.076489000
N	-4.222387000	-2.408600000	-1.170811000
N	1.051037000	-0.667470000	0.328517000
N	5.117816000	-0.165374000	-1.475002000
C	-5.405744000	3.238194000	1.505600000
C	-6.332656000	2.297072000	1.073312000
C	-5.914499000	1.197978000	0.327849000
C	-4.571526000	1.059682000	0.026685000
C	-3.656193000	2.007973000	0.466857000
C	-4.051447000	3.112671000	1.210122000
C	-3.901590000	-0.039269000	-0.759163000
C	-2.406939000	0.430608000	-0.807346000
C	-1.225421000	2.339036000	0.238637000
C	0.025385000	1.554178000	-0.114593000
C	-0.177569000	0.112922000	0.406909000
C	-1.261414000	-0.569043000	-0.456438000
C	-4.547121000	-0.248708000	-2.157264000
C	-5.083729000	-1.697076000	-2.116463000
C	-4.098280000	-1.432805000	-0.084159000
C	-3.014347000	-1.762625000	0.919721000
C	-1.686272000	-1.914421000	0.180074000
C	0.150191000	-4.430061000	-0.166368000
C	-1.055957000	-4.069140000	-0.977148000
C	-1.836570000	-2.997383000	-0.855564000
C	-2.945662000	-2.728654000	-1.828157000
C	1.321540000	2.274349000	0.320017000
C	2.470398000	1.280873000	0.469706000
C	2.213182000	-0.015241000	-0.297225000
C	1.552741000	-1.418640000	1.392059000

C	3.355261000	-1.046374000	-0.114036000
C	2.904907000	-1.719762000	1.160789000
C	0.918654000	-1.868648000	2.542932000
C	1.645017000	-2.664416000	3.431210000
C	2.968171000	-2.997383000	3.188099000
C	3.606923000	-2.512498000	2.042986000
C	3.862498000	1.882540000	0.238724000
C	4.879042000	0.865920000	0.756968000
C	4.759279000	-0.392152000	-0.074065000
C	4.729384000	-1.426970000	-2.115535000
C	3.492800000	-1.980290000	-1.340622000
C	4.441503000	0.983345000	-2.107103000
C	4.187872000	2.177315000	-1.205684000
C	4.245209000	3.407359000	-1.716297000
C	3.920014000	4.696711000	-1.023784000
H	-3.331528000	3.843838000	1.539762000
H	-2.959330000	-0.958772000	1.663440000
H	-5.070322000	-1.410452000	0.419463000
H	-6.107854000	-1.713932000	-1.735302000
H	-5.337802000	0.477018000	-2.344660000
H	-3.106959000	-3.588881000	-2.480839000
H	-6.633965000	0.463756000	-0.019139000
H	-7.380607000	2.420927000	1.312783000
H	-5.738271000	4.091630000	2.082992000
H	0.063091000	1.489489000	-1.211132000
H	-0.503759000	0.168330000	1.453246000
H	1.036585000	-4.468552000	-0.806146000
H	0.356988000	-3.726554000	0.637897000
H	-2.218258000	0.794765000	-1.821520000
H	-0.912195000	-2.211994000	0.881522000

H	-2.621351000	-1.895373000	-2.478928000
H	-1.300878000	-4.767078000	-1.775583000
H	-3.806198000	-0.124858000	-2.948393000
H	-5.078737000	-2.187100000	-3.090614000
H	-3.259161000	-2.687237000	1.445431000
H	-0.750750000	-0.796698000	-1.397415000
H	0.036041000	-5.425081000	0.270559000
H	1.559775000	2.995945000	-0.476031000
H	0.476447000	3.525921000	1.498094000
H	3.248422000	5.295645000	-1.643252000
H	3.437625000	4.553585000	-0.057483000
H	4.821369000	5.297014000	-0.871175000
H	4.550928000	3.505057000	-2.756310000
H	5.050190000	1.302958000	-2.956637000
H	3.478384000	0.668354000	-2.536296000
H	4.682359000	0.635603000	1.807114000
H	5.896745000	1.254151000	0.677657000
H	3.925981000	2.796769000	0.830029000
H	2.443255000	0.997133000	1.530133000
H	1.999034000	0.163921000	-1.351812000
H	-0.110629000	-1.615563000	2.764932000
H	1.158562000	-3.022506000	4.330128000
H	3.509951000	-3.617986000	3.889335000
H	4.648260000	-2.752679000	1.857662000
H	3.672726000	-3.007478000	-1.020291000
H	5.559945000	-2.130999000	-2.032458000
H	2.580049000	-1.982208000	-1.939628000
H	4.536827000	-1.270328000	-3.176966000
H	5.457473000	-1.149600000	0.303061000

Strychnobaillonine (Conformer 2, *E* = -1919.8615309 Hartree), solvent: Chloroform

O	1.287759000	2.818333000	1.650808000
O	-1.097257000	3.454516000	0.651979000
N	-2.303306000	1.604677000	0.130554000
N	-4.653674000	-2.380586000	-0.221424000
N	1.102273000	-0.761214000	0.278203000
N	5.231380000	-0.165812000	-1.403725000
C	-5.350444000	3.641132000	0.852521000
C	-6.292286000	2.713919000	0.425280000
C	-5.883521000	1.475877000	-0.065021000
C	-4.531670000	1.189388000	-0.130255000
C	-3.599348000	2.132616000	0.295379000
C	-3.986480000	3.368092000	0.795893000
C	-3.844361000	-0.084763000	-0.565460000
C	-2.378020000	0.392417000	-0.712675000
C	-1.141474000	2.296129000	0.270868000
C	0.093612000	1.477922000	-0.060638000
C	-0.117968000	0.028327000	0.425894000
C	-1.240280000	-0.613114000	-0.419779000
C	-4.390909000	-0.685203000	-1.874240000
C	-5.310954000	-1.807580000	-1.391411000
C	-4.086617000	-1.229483000	0.499072000
C	-2.813193000	-1.609416000	1.241691000
C	-1.728833000	-1.931376000	0.207497000
C	-0.062385000	-3.216561000	-2.031531000
C	-1.522761000	-3.457670000	-1.785793000
C	-2.260513000	-2.932820000	-0.808331000
C	-3.664361000	-3.416688000	-0.536730000
C	1.391746000	2.179083000	0.402656000
C	2.524135000	1.170932000	0.556353000

C	2.278282000	-0.076453000	-0.289056000
C	1.587800000	-1.601431000	1.281807000
C	3.415160000	-1.122452000	-0.163728000
C	2.941640000	-1.890058000	1.047598000
C	0.936818000	-2.133512000	2.387679000
C	1.650132000	-2.989732000	3.228322000
C	2.977335000	-3.305046000	2.982121000
C	3.631681000	-2.742946000	1.882552000
C	3.926615000	1.770859000	0.401096000
C	4.920720000	0.715789000	0.881895000
C	4.821742000	-0.482447000	-0.034930000
C	4.864479000	-1.383612000	-2.133322000
C	3.578412000	-1.956335000	-1.459161000
C	4.566726000	1.017689000	-1.981049000
C	4.288580000	2.153538000	-1.013748000
C	4.345071000	3.413142000	-1.445643000
C	3.983935000	4.651264000	-0.680518000
H	-3.252753000	4.087981000	1.119353000
H	-2.502081000	-0.776622000	1.879418000
H	-4.840182000	-0.867908000	1.203869000
H	-6.278722000	-1.400551000	-1.081384000
H	-4.890215000	0.063579000	-2.490441000
H	-3.616255000	-4.096097000	0.322593000
H	-6.618502000	0.750940000	-0.394974000
H	-7.346634000	2.953080000	0.473707000
H	-5.678608000	4.599669000	1.234214000
H	0.140936000	1.435141000	-1.158390000
H	-0.402060000	0.061642000	1.486018000
H	0.423375000	-2.704420000	-1.199262000
H	0.450111000	-4.167757000	-2.192350000

H	-2.271967000	0.738823000	-1.747101000
H	-0.869252000	-2.396404000	0.694278000
H	-4.034748000	-4.011228000	-1.373342000
H	-2.005055000	-4.167624000	-2.454380000
H	-3.567909000	-1.115176000	-2.452868000
H	-5.502384000	-2.572222000	-2.143703000
H	-3.009357000	-2.459468000	1.898535000
H	-0.770725000	-0.839305000	-1.379101000
H	0.099960000	-2.618686000	-2.934881000
H	1.650683000	2.905910000	-0.381732000
H	0.561708000	3.450226000	1.568931000
H	3.447383000	4.439784000	0.244119000
H	4.874446000	5.236199000	-0.434351000
H	3.347226000	5.294713000	-1.291855000
H	4.674593000	3.579227000	-2.469585000
H	5.193174000	1.389324000	-2.795830000
H	3.613297000	0.726708000	-2.448824000
H	4.690100000	0.418146000	1.907972000
H	5.942381000	1.100936000	0.860962000
H	3.981377000	2.645886000	1.049713000
H	2.456911000	0.835149000	1.600022000
H	2.081366000	0.171772000	-1.333267000
H	-0.094334000	-1.891905000	2.613328000
H	1.151114000	-3.409795000	4.092803000
H	3.509526000	-3.972771000	3.646578000
H	4.675686000	-2.970050000	1.696277000
H	3.705513000	-3.014302000	-1.225844000
H	5.680041000	-2.103692000	-2.039906000
H	2.695110000	-1.869030000	-2.095159000
H	4.734091000	-1.168837000	-3.194070000

H	5.501457000	-1.269716000	0.313452000

Equilibrium geometries of molecules from set **1** calculated at the **DFT(M06-2X)** level of theory with **pecG-2** basis set within the IEF-PCM model. Cartesian coordinates are given for standard orientation in form (atomic label, X, Y, Z). The X, Y, Z are given in Å.

12-28-oxaircinal (*E* = -1272.2787683 Hartree), solvent: Chloroform

O	1.590275000	-0.779245000	1.826498000
O	-0.162201000	4.731007000	1.505669000
N	1.763469000	-0.503118000	-0.456756000
N	-2.187906000	0.489535000	-1.534364000
C	0.064880000	1.166638000	-0.860626000
C	0.388742000	-0.135428000	-0.098721000
C	0.393210000	-0.096581000	1.455818000
C	-0.581193000	2.285472000	-0.009603000
C	1.449653000	1.610342000	-1.389259000
C	2.485797000	0.761717000	-0.643724000
C	-0.862493000	0.809529000	-2.024796000
C	2.180910000	-1.346265000	0.644875000
C	-2.104723000	2.170449000	0.191131000
C	0.078683000	2.379501000	1.335068000
C	0.496973000	1.298646000	1.995602000
C	-0.849011000	-0.808570000	2.015456000
C	-2.826757000	1.705434000	-1.060745000
C	3.669223000	-1.489501000	0.865688000
C	3.784389000	0.685935000	-1.392053000
C	-2.981934000	-0.302587000	-2.457341000
C	-0.834047000	-2.329500000	1.808059000
C	4.367682000	-2.316415000	-0.209670000
C	0.211635000	3.689772000	1.989992000
C	4.462391000	-0.370907000	-1.828204000

C	4.118136000	-1.825175000	-1.643896000
C	-4.291177000	-0.800479000	-1.840372000
C	-2.190917000	-2.931577000	2.055838000
C	-4.162770000	-1.321245000	-0.409656000
C	-3.162773000	-2.468946000	-0.239829000
C	-3.178133000	-2.994313000	1.166528000
H	-0.295130000	-0.921502000	-0.413286000
H	-0.409066000	3.222746000	-0.542771000
H	1.630779000	2.675147000	-1.253851000
H	1.522477000	1.391775000	-2.456070000
H	2.688505000	1.240990000	0.326065000
H	-0.890451000	1.642081000	-2.747602000
H	-0.445662000	-0.056337000	-2.542694000
H	1.735373000	-2.334961000	0.484811000
H	-2.480463000	3.140153000	0.518806000
H	-2.319459000	1.459689000	0.987528000
H	0.940934000	1.393890000	2.981884000
H	-1.719757000	-0.401802000	1.499881000
H	-0.966974000	-0.584849000	3.077199000
H	-3.869291000	1.507108000	-0.822308000
H	-2.810751000	2.486542000	-1.838229000
H	3.799130000	-1.971712000	1.834057000
H	4.115190000	-0.498549000	0.945259000
H	4.207337000	1.666994000	-1.587002000
H	-2.370253000	-1.160409000	-2.742257000
H	-3.206628000	0.247423000	-3.384363000
H	-0.095587000	-2.774260000	2.472772000
H	-0.517261000	-2.561629000	0.792507000
H	4.040101000	-3.355170000	-0.137938000
H	5.438751000	-2.310431000	-0.004438000

H	0.690007000	3.668635000	2.984521000
H	5.393349000	-0.166081000	-2.344920000
H	3.077021000	-1.997958000	-1.902504000
H	4.723734000	-2.422986000	-2.323222000
H	-4.679342000	-1.590692000	-2.485975000
H	-5.037951000	-0.005887000	-1.859202000
H	-2.376067000	-3.316543000	3.052204000
H	-5.145536000	-1.653542000	-0.069422000
H	-3.863029000	-0.507191000	0.253589000
H	-2.168984000	-2.118832000	-0.516948000
H	-3.427501000	-3.273327000	-0.932534000
H	-4.119344000	-3.433207000	1.483598000

Anabsinthin (Conformer 1, *E* = -1618.6356631 Hartree), solvent: Acetonitrile

O	-2.326533000	1.168341000	-2.232520000
O	-2.556604000	-1.950214000	0.629591000
O	2.366931000	0.207543000	3.124260000
O	3.074599000	-1.625086000	-1.236985000
O	-3.746760000	-3.384356000	1.839227000
O	4.562276000	-2.115968000	-2.816013000
C	-1.642223000	0.160884000	-0.255818000
C	-1.970173000	1.660333000	0.007041000
C	-0.494057000	2.116596000	-0.075184000
C	-1.163926000	0.500167000	-1.702828000
C	-0.520835000	-0.128566000	0.773917000
C	0.147719000	1.248226000	1.014234000
C	-2.801341000	2.082819000	-1.216917000
C	-0.087803000	1.571866000	-1.462080000

C	-2.807979000	-0.828710000	-0.254318000
C	1.678151000	1.048554000	0.914440000
C	0.618707000	-1.027294000	0.380458000
C	-4.172119000	-0.311982000	0.192448000
C	-4.316281000	1.918232000	-1.031194000
C	1.786311000	-0.381488000	0.417590000
C	-0.755879000	-0.583443000	-2.666073000
C	-4.862292000	0.503854000	-0.883335000
C	2.473981000	1.314501000	2.231067000
C	-2.566394000	3.529925000	-1.646109000
C	-4.869507000	-1.614847000	0.576109000
C	3.097657000	-1.017643000	0.086891000
C	0.403452000	-2.475245000	0.084160000
C	3.976251000	1.511373000	1.983845000
C	-3.714148000	-2.437200000	1.103650000
C	4.302607000	-0.092356000	0.032339000
C	4.776118000	0.349773000	1.403621000
C	1.970845000	2.586372000	2.919316000
C	-6.023725000	-1.511021000	1.553390000
C	5.270187000	-0.939878000	-0.789645000
C	4.319081000	-1.630898000	-1.746563000
C	6.394684000	-0.208679000	-1.495352000
H	-2.437339000	1.886738000	0.962992000
H	-0.320588000	3.181033000	0.062411000
H	-0.996799000	-0.517527000	1.675233000
H	-0.150073000	1.641202000	1.981408000
H	0.912237000	1.147036000	-1.476489000
H	-0.141611000	2.312401000	-2.255814000
H	-2.924578000	-1.252697000	-1.253688000
H	2.085475000	1.755746000	0.184981000

H	-4.048284000	0.282971000	1.103274000
H	-4.809301000	2.387086000	-1.884140000
H	-4.593585000	2.506243000	-0.153475000
H	-1.525700000	-1.343414000	-2.788482000
H	-0.571779000	-0.133049000	-3.641184000
H	0.165265000	-1.057117000	-2.332356000
H	-4.776888000	-0.030597000	-1.831582000
H	-5.926953000	0.574125000	-0.656951000
H	-3.079437000	3.713390000	-2.588932000
H	-2.978970000	4.206691000	-0.897667000
H	-1.516651000	3.768647000	-1.777511000
H	-5.197378000	-2.118364000	-0.340965000
H	3.306265000	-1.828078000	0.793224000
H	-0.309203000	-2.608965000	-0.730443000
H	1.326953000	-2.983318000	-0.177543000
H	-0.039814000	-2.959432000	0.955150000
H	4.418548000	1.776202000	2.944788000
H	4.089128000	2.383633000	1.336227000
H	4.038980000	0.784601000	-0.568866000
H	4.754113000	-0.502263000	2.086130000
H	5.817036000	0.668967000	1.335253000
H	0.999101000	2.435222000	3.382399000
H	1.886611000	3.406274000	2.205248000
H	2.666654000	2.879351000	3.703153000
H	-5.700398000	-1.029276000	2.475368000
H	-6.408486000	-2.498105000	1.799287000
H	-6.833371000	-0.924287000	1.124118000
H	1.436653000	0.001574000	3.256176000
H	5.677330000	-1.728470000	-0.146146000
H	6.981120000	-0.897700000	-2.098745000

H	7.056985000	0.261167000	-0.771083000
H	5.995035000	0.564909000	-2.149850000

Anabsinthin (Conformer 2, *E* = -1618.6355941 Hartree), solvent: Acetonitrile

O	-2.157333000	2.331060000	-1.019351000
O	-2.607332000	-1.867311000	-0.714439000
O	1.550492000	-1.748834000	3.011113000
O	3.035667000	-0.498877000	-1.999938000
O	-3.887054000	-3.682832000	-0.663199000
O	4.440395000	0.342733000	-3.504949000
C	-1.588323000	0.285228000	-0.082331000
C	-1.869960000	1.317090000	1.047921000
C	-0.375392000	1.650004000	1.275365000
C	-1.039491000	1.420115000	-1.006787000
C	-0.522588000	-0.641468000	0.558779000
C	0.191913000	0.263943000	1.599232000
C	-2.639108000	2.446614000	0.340752000
C	0.062725000	2.058682000	-0.146926000
C	-2.788445000	-0.428371000	-0.705054000
C	1.702225000	0.068267000	1.382784000
C	0.586167000	-1.150495000	-0.329488000
C	-4.146001000	-0.235534000	-0.037824000
C	-4.165064000	2.281106000	0.374007000
C	1.773264000	-0.697698000	0.080276000
C	-0.633852000	1.145148000	-2.431456000
C	-4.770394000	1.104021000	-0.380465000
C	2.393098000	-0.691686000	2.546935000
C	-2.335999000	3.835877000	0.899235000
C	-4.903158000	-1.460233000	-0.545255000
C	3.091303000	-0.901743000	-0.605027000

C	0.327181000	-2.062705000	-1.482857000
C	3.731749000	-1.333102000	2.138113000
C	-3.796079000	-2.486672000	-0.646793000
C	4.210854000	-0.036533000	-0.033898000
C	4.733060000	-0.502209000	1.328327000
C	2.571086000	0.242699000	3.730947000
C	-6.077970000	-1.930604000	0.289513000
C	5.190287000	-0.019535000	-1.201860000
C	4.235665000	-0.020997000	-2.381583000
C	6.188444000	1.120316000	-1.243842000
H	-2.363920000	0.929318000	1.936394000
H	-0.169735000	2.390902000	2.044048000
H	-1.044595000	-1.477248000	1.026474000
H	-0.092771000	-0.015444000	2.610588000
H	1.047563000	1.682589000	-0.410486000
H	0.064503000	3.132234000	-0.315145000
H	-2.885458000	-0.129497000	-1.750810000
H	2.201987000	1.036907000	1.308643000
H	-4.027992000	-0.336450000	1.045824000
H	-4.607847000	3.199492000	-0.014710000
H	-4.452811000	2.222170000	1.426038000
H	-0.417558000	2.094224000	-2.921382000
H	0.268378000	0.536844000	-2.457014000
H	-1.419130000	0.647074000	-2.997687000
H	-4.678625000	1.263379000	-1.456929000
H	-5.837851000	1.073347000	-0.157986000
H	-2.757465000	3.929680000	1.900323000
H	-1.273749000	4.046882000	0.957756000
H	-2.800690000	4.588328000	0.263783000
H	-5.226143000	-1.269358000	-1.575402000

H	3.385074000	-1.955468000	-0.604518000
H	-0.132977000	-2.983468000	-1.122437000
H	-0.382451000	-1.624588000	-2.185088000
H	1.241099000	-2.305291000	-2.018484000
H	3.496124000	-2.237588000	1.572609000
H	4.215437000	-1.676250000	3.053194000
H	3.810412000	0.976340000	0.050400000
H	5.615954000	-1.124891000	1.172950000
H	5.066613000	0.361528000	1.903530000
H	2.915538000	-0.319342000	4.597468000
H	1.629473000	0.729194000	3.983553000
H	3.304312000	1.013978000	3.499571000
H	-6.509409000	-2.836374000	-0.130310000
H	-6.850866000	-1.165455000	0.323207000
H	-5.759855000	-2.143199000	1.309474000
H	1.376884000	-2.341404000	2.271974000
H	5.714699000	-0.981127000	-1.239253000
H	6.864993000	1.062196000	-0.393372000
H	5.672718000	2.079265000	-1.208369000
H	6.778286000	1.081921000	-2.156747000

Betulinic Acid (*E* = -1397.7282477 Hartree), solvent: Pyridine

C	4.178173000	1.209961000	0.364043000
C	3.152136000	0.295428000	-0.337472000
C	3.791446000	-1.100549000	-0.367869000
C	5.243493000	-0.751559000	-0.738335000
C	5.544665000	0.487650000	0.121240000
C	1.712932000	0.256338000	0.164093000
C	0.839230000	-0.700023000	-0.700932000
C	1.544301000	-2.068128000	-0.887599000

C	3.008784000	-1.977841000	-1.338216000
C	1.067682000	1.635609000	0.260921000
C	-0.369025000	1.573503000	0.788837000
C	-1.249800000	0.530560000	0.085080000
C	-0.553889000	-0.863373000	0.044081000
C	-2.740933000	0.539236000	0.567268000
C	-3.504487000	-0.449346000	-0.359493000
C	-2.907786000	-1.852716000	-0.263673000
C	-1.447799000	-1.862317000	-0.710175000
C	-3.307230000	1.952419000	0.318143000
C	-4.826166000	2.023833000	0.407913000
C	-5.493026000	1.041832000	-0.531703000
C	-5.057077000	-0.417950000	-0.285552000
C	4.220800000	2.634905000	-0.134100000
C	4.244569000	3.658754000	0.712916000
C	4.294995000	2.847586000	-1.620830000
C	3.854379000	-1.753479000	1.004696000
O	3.728874000	-1.208189000	2.070695000
O	4.144212000	-3.063788000	0.925738000
C	0.665586000	-0.124597000	-2.122511000
C	-0.344880000	-1.418984000	1.469417000
C	-2.891901000	0.225580000	2.069298000
C	-5.626303000	-1.272244000	-1.427438000
C	-5.639697000	-0.955146000	1.025021000
O	-6.896366000	1.233609000	-0.390744000
H	3.971659000	1.230728000	1.432248000
H	3.141550000	0.598052000	-1.387297000
H	5.260208000	-0.500459000	-1.800069000
H	5.943231000	-1.569612000	-0.576861000
H	6.271590000	1.142797000	-0.354706000

H	5.965159000	0.189092000	1.079701000
H	1.754433000	-0.154029000	1.173289000
H	0.986981000	-2.656081000	-1.617147000
H	1.526673000	-2.642474000	0.038652000
H	3.090228000	-1.535807000	-2.331331000
H	3.435114000	-2.977175000	-1.399979000
H	1.090518000	2.125085000	-0.715800000
H	1.661200000	2.265235000	0.923530000
H	-0.816069000	2.560584000	0.684161000
H	-0.328555000	1.374124000	1.860127000
H	-1.336626000	0.849384000	-0.955388000
H	-3.279195000	-0.097111000	-1.376367000
H	-3.457585000	-2.542391000	-0.901517000
H	-3.004290000	-2.242412000	0.749973000
H	-1.434886000	-1.639422000	-1.777603000
H	-1.038961000	-2.867897000	-0.593910000
H	-2.998146000	2.286569000	-0.677268000
H	-2.880383000	2.655432000	1.033039000
H	-5.164282000	3.028279000	0.152616000
H	-5.174144000	1.832839000	1.424191000
H	-5.196776000	1.296214000	-1.558178000
H	4.327791000	4.679495000	0.363647000
H	4.176607000	3.503345000	1.781938000
H	3.329985000	2.650091000	-2.090740000
H	4.580403000	3.869786000	-1.856514000
H	5.015546000	2.168992000	-2.082004000
H	4.223804000	-3.402855000	1.827569000
H	0.181264000	0.845166000	-2.148810000
H	1.629531000	-0.001571000	-2.610439000
H	0.087633000	-0.800429000	-2.749045000

H	-1.261219000	-1.862532000	1.845364000
H	0.410113000	-2.200884000	1.489804000
H	-0.041822000	-0.662120000	2.186827000
H	-2.006331000	0.528565000	2.622208000
H	-3.729055000	0.769644000	2.499625000
H	-3.059708000	-0.828472000	2.272842000
H	-5.156026000	-1.023862000	-2.380192000
H	-5.485124000	-2.334904000	-1.242906000
H	-6.701908000	-1.122278000	-1.526562000
H	-6.718556000	-1.067509000	0.928063000
H	-5.221262000	-1.935993000	1.250471000
H	-5.452380000	-0.304649000	1.873638000
H	-7.350724000	0.760713000	-1.090812000

Icajine (Conformer 1, *E* = -1187.9288098 Hartree), solvent: Chloroform

O	-0.150553000	-2.868794000	1.222637000
O	2.093611000	3.008593000	0.717020000
O	-2.514004000	3.212057000	-0.437051000
N	-1.440552000	1.203731000	-0.489066000
N	2.080353000	-2.087477000	-0.565830000
C	-0.150910000	0.517779000	-0.306263000
C	0.202152000	-1.738724000	0.966026000
C	1.355854000	-1.729405000	-1.774370000
C	-0.148208000	-1.746259000	-1.500685000
C	-0.493499000	-0.991144000	-0.197701000
C	-2.001168000	-0.965142000	-0.014147000
C	-2.866246000	-2.006954000	0.261333000
C	-4.234498000	-1.753425000	0.310433000
C	-4.719695000	-0.474427000	0.076368000

C	-3.862379000	0.583755000	-0.207763000
C	-2.501924000	0.315785000	-0.243884000
C	1.202067000	-1.032381000	1.863474000
C	1.746289000	0.288729000	1.314313000
C	0.511519000	1.105583000	0.932199000
C	0.733860000	2.626194000	0.773988000
C	2.859680000	2.640712000	-0.406164000
C	3.170816000	1.183045000	-0.557351000
C	2.734537000	0.149539000	0.155540000
C	3.204131000	-1.238679000	-0.207480000
C	-0.104041000	3.193856000	-0.387788000
C	-1.482910000	2.568033000	-0.439838000
C	2.322251000	-3.505771000	-0.423879000
H	0.471204000	0.702623000	-1.179411000
H	1.595440000	-2.408986000	-2.598617000
H	1.669389000	-0.735653000	-2.089694000
H	-0.505394000	-2.770268000	-1.386177000
H	-0.695212000	-1.301509000	-2.333583000
H	-2.475406000	-2.997267000	0.440468000
H	-4.921355000	-2.558348000	0.527855000
H	-5.783986000	-0.290530000	0.113715000
H	-4.232383000	1.578300000	-0.389123000
H	1.978663000	-1.757371000	2.099845000
H	0.683342000	-0.821816000	2.801686000
H	2.261910000	0.801183000	2.127149000
H	-0.216553000	0.986226000	1.740375000
H	0.389288000	3.103479000	1.689087000
H	3.799679000	3.184195000	-0.299726000
H	2.409295000	3.010077000	-1.334270000
H	3.858163000	0.982799000	-1.373850000

H	3.689264000	-1.699162000	0.656116000
H	3.958753000	-1.179891000	-1.001819000
H	-0.214960000	4.269497000	-0.299622000
H	0.382243000	2.976643000	-1.339765000
H	3.085845000	-3.873471000	-1.120261000
H	1.395473000	-4.049407000	-0.600751000
H	2.640181000	-3.724167000	0.595625000

Icajine (Conformer 2, *E* = -1187.9300878 Hartree), solvent: Chloroform

O	-0.203790000	-2.756567000	1.294363000
O	1.916826000	2.849593000	-0.546249000
O	-2.710825000	3.153415000	-0.016910000
N	-1.518546000	1.251968000	-0.360749000
N	2.060166000	-2.118831000	-0.477035000
C	-0.196821000	0.590157000	-0.336855000
C	0.164927000	-1.643417000	0.992084000
C	1.405151000	-1.692738000	-1.701960000
C	-0.104216000	-1.698714000	-1.491418000
C	-0.502824000	-0.932855000	-0.210814000
C	-2.012209000	-0.965145000	-0.073755000
C	-2.842408000	-2.058376000	0.088620000
C	-4.219410000	-1.861208000	0.120383000
C	-4.746278000	-0.584558000	-0.018826000
C	-3.923331000	0.523406000	-0.188149000
C	-2.551522000	0.310053000	-0.208796000
C	1.139434000	-0.897390000	1.882895000
C	1.776673000	0.349284000	1.263118000
C	0.605675000	1.224716000	0.792482000
C	0.904997000	2.682295000	0.427053000
C	3.227164000	2.509756000	-0.126215000

C	3.530881000	1.058462000	-0.356498000
C	2.865431000	0.069720000	0.230441000
C	3.227431000	-1.359760000	-0.077673000
C	-0.338292000	3.368314000	-0.165588000
C	-1.638172000	2.594198000	-0.156008000
C	2.204939000	-3.551713000	-0.352637000
H	0.314235000	0.785091000	-1.281688000
H	1.744170000	-0.687072000	-1.948470000
H	1.674808000	-2.333695000	-2.548416000
H	-0.469137000	-2.721041000	-1.386943000
H	-0.617093000	-1.257257000	-2.347005000
H	-2.419408000	-3.045532000	0.197564000
H	-4.880505000	-2.705793000	0.249178000
H	-5.817374000	-0.441798000	0.001816000
H	-4.327756000	1.514163000	-0.297693000
H	0.572410000	-0.589026000	2.765207000
H	1.871924000	-1.623686000	2.229507000
H	2.268056000	0.894347000	2.075785000
H	-0.070795000	1.285715000	1.652282000
H	1.214442000	3.202501000	1.339179000
H	3.354720000	2.773419000	0.930660000
H	3.902300000	3.133906000	-0.705931000
H	4.313680000	0.818657000	-1.065709000
H	3.626512000	-1.834043000	0.822500000
H	4.027918000	-1.381860000	-0.828546000
H	-0.537673000	4.323746000	0.308708000
H	-0.112202000	3.571674000	-1.214099000
H	2.950619000	-3.958609000	-1.046624000
H	1.247115000	-4.031985000	-0.544663000
H	2.496703000	-3.802240000	0.666946000

Iguesterin (*E* = -1239.0240801 Hartree), solvent: Chloroform

O	-6.522687000	0.805976000	-0.980029000
O	-5.267365000	2.638340000	0.405864000
C	1.661060000	0.216381000	0.430447000
C	1.048015000	-1.223246000	0.558209000
C	3.202325000	0.149829000	0.639315000
C	3.965630000	-0.898011000	-0.223040000
C	1.728655000	-2.151030000	-0.463905000
C	1.012091000	1.082107000	1.523480000
C	3.236388000	-2.246818000	-0.252074000
C	-0.455633000	-1.116105000	0.310635000
C	-1.276377000	-0.069602000	1.057017000
C	-0.494633000	1.236033000	1.333454000
C	3.903098000	1.515409000	0.529050000
C	1.302007000	0.845821000	-0.926850000
C	4.198704000	-0.439850000	-1.675334000
C	1.277535000	-1.859938000	1.949817000
C	5.344630000	-1.121282000	0.411713000
C	4.387756000	1.900501000	-0.842440000
C	-2.609910000	0.245635000	0.387881000
C	4.520938000	1.013509000	-1.820969000
C	-1.096276000	-1.957276000	-0.519787000
C	-1.713966000	-0.699195000	2.419756000
C	-3.245756000	-0.770416000	-0.437313000
C	-2.493635000	-1.830755000	-0.824858000
C	4.727941000	3.350586000	-1.016123000
C	-3.275583000	1.389152000	0.664600000
C	-4.616340000	-0.605019000	-0.895004000
C	-4.622386000	1.611941000	0.188953000

C	-5.257582000	0.539364000	-0.590919000
C	-5.282868000	-1.671811000	-1.711890000
H	3.332629000	-0.170212000	1.676688000
H	1.313305000	-3.155623000	-0.379307000
H	1.507965000	-1.807713000	-1.475699000
H	1.245730000	0.683047000	2.509397000
H	1.429908000	2.086888000	1.504201000
H	3.671102000	-2.852641000	-1.051090000
H	3.444680000	-2.790290000	0.669382000
H	-0.669269000	1.921531000	0.506043000
H	-0.916379000	1.714739000	2.217005000
H	3.257246000	2.311871000	0.897983000
H	4.766557000	1.531208000	1.201564000
H	0.231934000	0.799711000	-1.123260000
H	1.591862000	1.897147000	-0.934117000
H	1.799309000	0.366978000	-1.761133000
H	5.026119000	-1.032930000	-2.076651000
H	3.345775000	-0.696920000	-2.308459000
H	2.328264000	-2.066681000	2.124400000
H	0.941183000	-1.247007000	2.774468000
H	0.740104000	-2.807184000	1.996666000
H	5.250297000	-1.366281000	1.470622000
H	5.978665000	-0.240460000	0.315639000
H	5.850785000	-1.951366000	-0.082836000
H	4.880701000	1.346030000	-2.788782000
H	-0.566352000	-2.751449000	-1.020449000
H	-0.869653000	-0.826945000	3.086581000
H	-2.189730000	-1.665819000	2.262720000
H	-2.426916000	-0.028426000	2.895076000
H	-2.933932000	-2.592027000	-1.454476000

H	5.450791000	3.670832000	-0.262852000
H	3.838831000	3.970635000	-0.881047000
H	5.143269000	3.550492000	-2.001323000
H	-2.853105000	2.178958000	1.267739000
H	-5.247583000	-2.634331000	-1.202412000
H	-4.790469000	-1.792443000	-2.677311000
H	-6.321596000	-1.415016000	-1.891435000
H	-6.716563000	1.691676000	-0.633151000

Itoaic acid (Conformer 1, *E* = -1623.4301014 Hartree), solvent: Chloroform

C	-3.419146000	-1.103661000	-0.264270000
C	-2.562055000	0.166089000	0.049720000
C	-1.258335000	-0.103167000	0.893668000
C	-0.422588000	-1.130550000	0.053849000
C	-1.217310000	-2.420875000	-0.128504000
C	-2.502673000	-2.160234000	-0.898673000
C	-0.434258000	1.212171000	0.996806000
C	1.055239000	1.012670000	1.293723000
C	1.779583000	0.046037000	0.343577000
C	1.061253000	-1.343336000	0.470228000
C	3.294092000	-0.072622000	0.749387000
C	4.088390000	-1.289309000	0.177418000
C	3.232865000	-2.555318000	-0.066650000
C	1.763709000	-2.365383000	-0.442012000
C	4.131099000	1.239627000	0.668317000
C	5.090376000	1.519805000	-0.519687000
C	5.782043000	0.233521000	-0.980282000
C	4.810177000	-0.926342000	-1.133029000
C	-3.377207000	1.352609000	0.579890000
C	-4.459223000	1.913210000	-0.351843000

C	-5.718097000	1.073237000	-0.288031000
O	-5.674658000	-0.172595000	-0.729258000
C	-4.490435000	-0.747047000	-1.332142000
O	-4.779211000	3.226431000	0.029102000
O	-6.736729000	1.507051000	0.188906000
C	-5.043982000	-1.913544000	-2.132827000
C	-4.137200000	-1.704318000	0.950887000
C	-1.596351000	-0.567162000	2.325101000
O	-0.941300000	2.132814000	1.956374000
C	1.159482000	-1.914586000	1.897454000
C	5.143247000	-1.673730000	1.228980000
C	6.147783000	2.507213000	-0.016502000
C	4.415316000	2.170509000	-1.732188000
C	1.690860000	0.613052000	-1.075175000
O	1.522238000	1.946175000	-1.084319000
O	1.791602000	0.013656000	-2.114685000
H	-2.181248000	0.488162000	-0.928331000
H	-0.366771000	-0.694754000	-0.950347000
H	-1.431903000	-2.896189000	0.828927000
H	-0.639357000	-3.140712000	-0.701476000
H	-3.052461000	-3.094111000	-1.013723000
H	-2.226889000	-1.828688000	-1.904683000
H	-0.530654000	1.733407000	0.044297000
H	1.170260000	0.643361000	2.313566000
H	1.504320000	2.001092000	1.276334000
H	3.208872000	-0.258770000	1.821546000
H	3.273551000	-3.180672000	0.824243000
H	3.719875000	-3.142169000	-0.847310000
H	1.286900000	-3.336974000	-0.323006000
H	1.654664000	-2.078002000	-1.482445000

H	4.758285000	1.237994000	1.560829000
H	3.476733000	2.104421000	0.782620000
H	6.566685000	-0.036972000	-0.271702000
H	6.286651000	0.426534000	-1.929726000
H	5.357116000	-1.811080000	-1.464816000
H	4.073116000	-0.710887000	-1.907427000
H	-2.713840000	2.193514000	0.742926000
H	-3.833625000	1.144590000	1.549541000
H	-4.088021000	1.932169000	-1.379630000
H	-4.070330000	-0.024335000	-2.033460000
H	-5.686963000	3.209624000	0.358457000
H	-4.290606000	-2.333553000	-2.791419000
H	-5.421919000	-2.699000000	-1.481075000
H	-5.868569000	-1.552639000	-2.742520000
H	-4.622616000	-0.948446000	1.564993000
H	-4.912345000	-2.393819000	0.618963000
H	-3.455659000	-2.264667000	1.582488000
H	-2.555980000	-0.173626000	2.649896000
H	-0.860648000	-0.218843000	3.047842000
H	-1.630605000	-1.646707000	2.425922000
H	-0.756820000	1.800518000	2.837845000
H	0.613322000	-2.857969000	1.930504000
H	0.749739000	-1.275830000	2.666322000
H	2.184944000	-2.133295000	2.177358000
H	5.778955000	-0.839021000	1.516826000
H	4.657459000	-2.043691000	2.132494000
H	5.785988000	-2.467896000	0.844645000
H	6.864461000	2.734671000	-0.806679000
H	5.687047000	3.443944000	0.302132000
H	6.696391000	2.092846000	0.829952000

H	5.186072000	2.493183000	-2.433136000
H	3.764981000	1.482068000	-2.266936000
H	3.839171000	3.046817000	-1.437346000
H	1.556814000	2.234739000	-2.006375000

Itoaic acid (Conformer 2, *E* = -1623.4299579Hartree), solvent: Chloroform

C	-3.485880000	-1.100151000	0.144997000
C	-2.631229000	0.205262000	0.009676000
C	-1.239982000	0.170059000	0.752978000
C	-0.488388000	-1.061081000	0.143479000
C	-1.262689000	-2.340878000	0.444840000
C	-2.615881000	-2.307678000	-0.246671000
C	-0.419565000	1.443618000	0.401344000
C	1.081611000	1.309927000	0.664142000
C	1.739389000	0.130370000	-0.066468000
C	1.039488000	-1.181825000	0.404718000
C	3.275804000	0.063664000	0.259251000
C	3.976487000	-1.280417000	-0.183549000
C	3.024426000	-2.186079000	-0.973727000
C	1.626257000	-2.395724000	-0.372719000
C	4.051818000	1.295194000	-0.231485000
C	5.551542000	1.282433000	0.099154000
C	6.187600000	-0.045251000	-0.398278000
C	5.174182000	-0.949762000	-1.090377000
C	-3.419693000	1.491358000	0.281720000
C	-4.602600000	1.782270000	-0.650679000
C	-5.828110000	0.990781000	-0.243002000
O	-5.798018000	-0.327138000	-0.342086000
C	-4.659960000	-1.048549000	-0.873224000
O	-4.918955000	3.149795000	-0.600385000

O	-6.807544000	1.537637000	0.198680000
C	-5.258617000	-2.388802000	-1.264491000
C	-4.075805000	-1.326217000	1.543609000
C	-1.415186000	0.137203000	2.284484000
O	-0.863083000	2.620554000	1.065866000
C	1.287347000	-1.435906000	1.903206000
C	4.517391000	-2.080639000	1.019266000
C	5.778342000	1.463360000	1.601027000
C	6.197396000	2.463320000	-0.627765000
C	1.498856000	0.385166000	-1.566746000
O	1.703290000	1.670788000	-1.908426000
O	1.132488000	-0.404307000	-2.396777000
H	-2.361500000	0.246688000	-1.053446000
H	-0.569394000	-0.928933000	-0.937317000
H	-1.388832000	-2.492368000	1.516340000
H	-0.713418000	-3.208582000	0.083311000
H	-3.162291000	-3.226372000	-0.033614000
H	-2.441500000	-2.283741000	-1.326782000
H	-0.572324000	1.655378000	-0.661020000
H	1.255885000	1.202633000	1.735184000
H	1.530902000	2.252884000	0.368884000
H	3.301371000	0.101224000	1.349396000
H	3.507629000	-3.158872000	-1.089986000
H	2.918190000	-1.785627000	-1.978211000
H	1.641073000	-3.252737000	0.302066000
H	0.961529000	-2.660818000	-1.192762000
H	3.615960000	2.200986000	0.191999000
H	3.952675000	1.393827000	-1.310696000
H	6.629739000	-0.581765000	0.443065000
H	7.009122000	0.171964000	-1.081757000

H	5.648855000	-1.885194000	-1.392840000
H	4.818914000	-0.477290000	-2.008960000
H	-2.763942000	2.342015000	0.142721000
H	-3.773084000	1.553029000	1.312654000
H	-4.333849000	1.526599000	-1.678992000
H	-4.323623000	-0.547840000	-1.782755000
H	-5.794076000	3.219651000	-0.197605000
H	-4.560925000	-2.976945000	-1.851519000
H	-5.553960000	-2.961458000	-0.387448000
H	-6.145428000	-2.206835000	-1.866486000
H	-4.508403000	-0.421376000	1.965858000
H	-4.870580000	-2.069637000	1.495018000
H	-3.329546000	-1.696587000	2.238899000
H	-2.324697000	0.651907000	2.585136000
H	-0.592948000	0.633658000	2.797875000
H	-1.456966000	-0.869561000	2.685246000
H	-0.629497000	2.560006000	1.995321000
H	0.735530000	-2.326069000	2.203250000
H	0.968640000	-0.624935000	2.545712000
H	2.331211000	-1.626512000	2.115671000
H	4.913576000	-1.443108000	1.808012000
H	3.756505000	-2.721847000	1.457302000
H	5.324321000	-2.735758000	0.687853000
H	5.320218000	0.665263000	2.183871000
H	6.845943000	1.463609000	1.824294000
H	5.360950000	2.411859000	1.943293000
H	6.085069000	2.358681000	-1.707847000
H	5.737911000	3.406424000	-0.327536000
H	7.263310000	2.516578000	-0.402585000
H	1.524218000	1.747125000	-2.855313000

Matopensine (*E* = -1767.4402386 Hartree), solvent: Chloroform

O	-0.000020000	0.000003000	2.591819000
N	-1.344439000	-0.879651000	0.949465000
N	-3.796793000	2.199837000	-1.054314000
C	-2.073553000	0.366813000	1.230245000
C	-3.330615000	0.258553000	0.335310000
C	-1.115286000	1.548568000	1.040636000
C	-0.206778000	-1.155972000	1.800244000
C	-3.097598000	0.908328000	-1.068906000
C	-0.945860000	1.984021000	-0.448239000
C	-1.626625000	1.005806000	-1.421647000
C	-4.566970000	1.008554000	0.870135000
C	-3.478077000	-1.243597000	0.244864000
C	-2.278572000	-1.851717000	0.632074000
C	-5.020940000	1.880133000	-0.316867000
C	-1.617171000	3.309580000	-0.724852000
C	-3.081357000	3.305761000	-0.387490000
C	-4.547162000	-2.015666000	-0.150291000
C	-2.142843000	-3.230576000	0.657811000
C	-1.064964000	4.357693000	-1.331706000
C	-4.425373000	-3.408846000	-0.138803000
C	-3.239192000	-3.999737000	0.266048000
C	0.333792000	4.509045000	-1.842438000
H	-2.408067000	0.355093000	2.274156000
H	-1.525536000	2.389777000	1.598100000
H	-0.458465000	-1.945808000	2.512520000
H	-3.616376000	0.288413000	-1.803268000
H	0.109951000	2.040320000	-0.689091000
H	-1.508953000	1.385388000	-2.436343000

H	-1.179349000	0.014168000	-1.375895000
H	-4.291706000	1.628271000	1.723722000
H	-5.343531000	0.326175000	1.207778000
H	-5.681250000	1.308181000	-0.970553000
H	-5.541970000	2.787345000	-0.018007000
H	-3.543662000	4.239394000	-0.705149000
H	-3.203219000	3.248871000	0.702694000
H	-5.478136000	-1.551896000	-0.452833000
H	-1.213461000	-3.700676000	0.954390000
H	-1.712090000	5.209739000	-1.517546000
H	-5.260458000	-4.025214000	-0.437584000
H	-3.156159000	-5.077632000	0.278042000
H	0.308591000	4.806428000	-2.891380000
H	0.866008000	5.296291000	-1.305789000
H	0.919564000	3.598542000	-1.762572000
N	1.344441000	0.879653000	0.949498000
N	3.796786000	-2.199835000	-1.054284000
C	2.073542000	-0.366818000	1.230286000
C	3.330617000	-0.258566000	0.335368000
C	1.115273000	-1.548565000	1.040659000
C	0.206755000	1.155977000	1.800246000
C	3.097604000	-0.908319000	-1.068860000
C	0.945855000	-1.984000000	-0.448219000
C	1.626634000	-1.005780000	-1.421613000
C	4.566955000	-1.008593000	0.870194000
C	3.478101000	1.243584000	0.244951000
C	2.278598000	1.851713000	0.632154000
C	5.020930000	-1.880152000	-0.316821000
C	1.617150000	-3.309565000	-0.724836000
C	3.081338000	-3.305763000	-0.387481000

C	4.547205000	2.015645000	-0.150167000
C	2.142893000	3.230573000	0.657930000
C	1.064917000	-4.357672000	-1.331675000
C	4.425439000	3.408826000	-0.138643000
C	3.239262000	3.999726000	0.266208000
C	-0.333861000	-4.509005000	-1.842355000
H	2.408041000	-0.355105000	2.274201000
H	1.525508000	-2.389782000	1.598122000
H	0.458428000	1.945811000	2.512529000
H	3.616391000	-0.288397000	-1.803211000
H	-0.109956000	-2.040287000	-0.689076000
H	1.508966000	-1.385351000	-2.436314000
H	1.179366000	-0.014139000	-1.375853000
H	4.291672000	-1.628325000	1.723765000
H	5.343520000	-0.326231000	1.207861000
H	5.681250000	-1.308193000	-0.970490000
H	5.541950000	-2.787374000	-0.017974000
H	3.543634000	-4.239397000	-0.705153000
H	3.203204000	-3.248892000	0.702705000
H	5.478177000	1.551867000	-0.452704000
H	1.213515000	3.700680000	0.954505000
H	1.712024000	-5.209729000	-1.517526000
H	5.260540000	4.025189000	-0.437392000
H	3.156247000	5.077622000	0.278232000
H	-0.308703000	-4.806401000	-2.891294000
H	-0.866073000	-5.296232000	-1.305675000
H	-0.919612000	-3.598489000	-1.762478000

Naucleidinal (*E* = -1109.3391891 Hartree), solvent: Chloroform

O	-3.462481000	1.450320000	1.343972000

O	-1.243219000	-2.000307000	1.053003000
O	-6.769686000	-0.025657000	-1.583211000
N	-3.002007000	-3.201822000	0.292589000
N	-5.076557000	-2.960678000	-2.736078000
C	-4.338156000	-3.270062000	-0.303999000
C	-4.557172000	-0.808807000	-0.096810000
C	-5.235661000	-2.142078000	0.169510000
C	-5.390566000	0.387455000	0.331978000
C	-4.138484000	-3.311976000	-1.792840000
C	-3.249750000	-0.806843000	0.642943000
C	-2.134631000	-4.296008000	-0.140371000
C	-2.981464000	-3.705576000	-2.395145000
C	-2.410344000	-2.030227000	0.685754000
C	-1.770328000	-4.135603000	-1.623918000
C	-4.500147000	1.639839000	0.375326000
C	-3.193376000	-3.576711000	-3.808854000
C	-2.831228000	0.264982000	1.319125000
C	-4.511228000	-3.096393000	-3.983793000
C	-6.584452000	0.592121000	-0.567364000
C	-5.214677000	2.915836000	0.754007000
C	-2.400844000	-3.813030000	-4.937338000
C	-5.043694000	-2.836847000	-5.243610000
C	-2.927280000	-3.560066000	-6.188282000
C	-4.235424000	-3.073461000	-6.338607000
H	-4.777964000	-4.215601000	0.023465000
H	-4.379596000	-0.726891000	-1.172939000
H	-6.198682000	-2.208500000	-0.333976000
H	-5.413481000	-2.256784000	1.239926000
H	-5.778825000	0.246390000	1.348084000
H	-2.683970000	-5.225044000	0.009663000

H	-1.250760000	-4.306338000	0.486119000
H	-1.377374000	-5.080496000	-2.001067000
H	-0.972513000	-3.396808000	-1.728472000
H	-4.014348000	1.746302000	-0.599259000
H	-5.945031000	-2.488516000	-2.545718000
H	-1.935607000	0.251786000	1.923073000
H	-7.314077000	1.350628000	-0.241391000
H	-5.901165000	3.223422000	-0.031779000
H	-5.772697000	2.782783000	1.679890000
H	-4.485375000	3.709325000	0.897168000
H	-1.390892000	-4.185226000	-4.829118000
H	-6.051129000	-2.462566000	-5.359310000
H	-2.326121000	-3.736433000	-7.068747000
H	-4.617737000	-2.880338000	-7.330704000

Physalin D (*E* = -1913.7425951 Hartree), solvent: Dimethylsulfoxide

C	-6.148385000	0.488544000	-0.606271000
C	-5.534314000	-0.859325000	-0.399635000
C	-4.015105000	-0.823271000	-0.547701000
C	-3.387648000	0.348203000	0.264898000
C	-3.970905000	1.607925000	-0.384022000
C	-5.438207000	1.615684000	-0.596868000
C	-3.394932000	-2.171398000	-0.196489000
C	-1.884046000	-2.133239000	-0.337232000
C	-1.246334000	-1.024775000	0.507737000
C	-1.841825000	0.351098000	0.133832000
O	-3.299628000	2.558263000	-0.724857000
C	0.299997000	-1.063994000	0.384206000
C	1.114757000	-0.784347000	1.665510000
C	2.364492000	-0.045910000	1.222587000

C	1.772006000	0.771395000	0.070798000
C	1.211613000	2.126821000	0.595593000
C	-0.225465000	2.402257000	0.175644000
C	-1.225246000	1.515160000	0.937570000
O	0.812608000	-1.129084000	2.769960000
O	0.781373000	-0.075390000	-0.513613000
O	0.662316000	-2.331875000	-0.086537000
C	3.530819000	-0.980975000	0.833289000
C	3.146007000	-2.134013000	-0.118973000
C	1.901836000	-2.928114000	0.269010000
C	4.565835000	-0.109747000	0.126132000
C	4.008813000	0.369470000	-1.192610000
C	2.736631000	1.218598000	-1.063840000
C	2.227135000	3.140040000	0.063336000
O	3.175542000	2.532851000	-0.661591000
O	2.251416000	4.306595000	0.318952000
C	3.113813000	-1.833469000	-1.609046000
O	3.727887000	-0.732773000	-2.064431000
C	2.045729000	1.366653000	-2.411204000
O	2.652074000	-2.602725000	-2.405257000
C	4.121682000	-1.560004000	2.118034000
O	-3.678329000	-0.651932000	-1.927348000
O	-3.794731000	-2.523641000	1.118708000
C	-3.838478000	0.350309000	1.741997000
O	1.320278000	2.207424000	2.007863000
H	-7.214313000	0.527769000	-0.791695000
H	-5.933925000	-1.563850000	-1.131742000
H	-5.813774000	-1.248983000	0.579656000
H	-5.887089000	2.579241000	-0.795669000
H	-3.799913000	-2.892882000	-0.912417000

H	-1.632732000	-1.989391000	-1.388123000
H	-1.493216000	-3.104437000	-0.045103000
H	-1.455645000	-1.234989000	1.560139000
H	-1.627626000	0.512760000	-0.922385000
H	2.713284000	0.584526000	2.031745000
H	-0.350395000	2.281251000	-0.897738000
H	-0.415486000	3.453445000	0.398469000
H	-2.013954000	2.171729000	1.281823000
H	-0.760640000	1.132804000	1.847785000
H	3.973848000	-2.848666000	-0.052391000
H	1.923685000	-3.149382000	1.337324000
H	1.904882000	-3.870608000	-0.267777000
H	4.828964000	0.748354000	0.745232000
H	5.477832000	-0.677977000	-0.059058000
H	4.738360000	0.967735000	-1.733543000
H	2.774885000	1.729242000	-3.133608000
H	1.240081000	2.094265000	-2.343394000
H	1.638857000	0.424122000	-2.757912000
H	4.462564000	-0.754519000	2.766767000
H	3.392645000	-2.148472000	2.673283000
H	4.974030000	-2.197210000	1.886620000
H	-4.307804000	-0.052738000	-2.338958000
H	-3.453374000	-3.400053000	1.312774000
H	-3.554663000	1.280837000	2.226997000
H	-4.916864000	0.256250000	1.832349000
H	-3.385990000	-0.474573000	2.282512000
H	1.025386000	3.085236000	2.275562000

Strychnobaillonine (Conformer 1, *E* = -1920.1268596 Hartree), solvent: Chloroform

O	1.187111000	2.835433000	1.727348000

O	-1.243261000	3.455342000	0.794368000
N	-2.375091000	1.617088000	0.102132000
N	-4.168023000	-2.410347000	-1.237390000
N	1.062071000	-0.644660000	0.379804000
N	5.080980000	-0.145644000	-1.522068000
C	-5.458865000	3.165214000	1.520692000
C	-6.365197000	2.223205000	1.051672000
C	-5.920361000	1.147290000	0.290479000
C	-4.572108000	1.031421000	0.009256000
C	-3.677584000	1.981468000	0.484100000
C	-4.099327000	3.063733000	1.244943000
C	-3.878461000	-0.046533000	-0.782211000
C	-2.388704000	0.435259000	-0.794482000
C	-1.243368000	2.329526000	0.315727000
C	0.018019000	1.564563000	-0.038245000
C	-0.179418000	0.112988000	0.452545000
C	-1.243679000	-0.563109000	-0.436665000
C	-4.497255000	-0.239496000	-2.193975000
C	-5.018241000	-1.693350000	-2.188836000
C	-4.077554000	-1.452011000	-0.132584000
C	-3.013012000	-1.787638000	0.888708000
C	-1.668751000	-1.919138000	0.176264000
C	0.154198000	-4.455221000	-0.135497000
C	-1.020017000	-4.069508000	-0.980450000
C	-1.791459000	-2.990843000	-0.874234000
C	-2.873876000	-2.706967000	-1.870934000
C	1.299655000	2.278105000	0.432210000
C	2.479187000	1.308623000	0.499036000
C	2.206990000	0.014776000	-0.265532000
C	1.581383000	-1.402790000	1.426607000

C	3.356970000	-1.009058000	-0.105788000
C	2.934908000	-1.682334000	1.179325000
C	0.962511000	-1.872988000	2.576241000
C	1.708345000	-2.661385000	3.453161000
C	3.034601000	-2.968847000	3.198304000
C	3.655359000	-2.468040000	2.050574000
C	3.851719000	1.924158000	0.190491000
C	4.896348000	0.927063000	0.690470000
C	4.759138000	-0.349301000	-0.109019000
C	4.693049000	-1.423957000	-2.127075000
C	3.464470000	-1.954874000	-1.327527000
C	4.366004000	0.977800000	-2.157641000
C	4.119931000	2.187582000	-1.273178000
C	4.149453000	3.402090000	-1.820140000
C	3.848572000	4.716663000	-1.168844000
H	-3.397725000	3.795898000	1.604832000
H	-2.978940000	-0.996877000	1.643707000
H	-5.058185000	-1.443192000	0.347008000
H	-6.047268000	-1.726199000	-1.830436000
H	-5.291708000	0.478367000	-2.380991000
H	-3.011274000	-3.551486000	-2.544150000
H	-6.622801000	0.412196000	-0.082208000
H	-7.416606000	2.327655000	1.276014000
H	-5.811182000	3.999181000	2.110732000
H	0.070949000	1.527150000	-1.132356000
H	-0.521258000	0.140686000	1.491649000
H	1.051794000	-4.526688000	-0.752245000
H	0.359551000	-3.749087000	0.663728000
H	-2.181295000	0.811232000	-1.797540000
H	-0.908057000	-2.215570000	0.889391000

H	-2.541335000	-1.858038000	-2.491357000
H	-1.248857000	-4.756880000	-1.789658000
H	-3.745724000	-0.094568000	-2.967597000
H	-4.985184000	-2.164799000	-3.168741000
H	-3.260472000	-2.719133000	1.395445000
H	-0.719579000	-0.773836000	-1.370579000
H	0.000056000	-5.439186000	0.308700000
H	1.507590000	3.071462000	-0.295295000
H	0.432083000	3.436848000	1.704106000
H	3.053371000	5.230563000	-1.710491000
H	3.534171000	4.620858000	-0.133441000
H	4.721264000	5.370257000	-1.201130000
H	4.412864000	3.465511000	-2.872161000
H	4.943165000	1.290797000	-3.027394000
H	3.400734000	0.635933000	-2.550905000
H	4.741042000	0.721006000	1.749781000
H	5.904721000	1.320442000	0.565118000
H	3.933778000	2.851094000	0.753336000
H	2.519471000	1.014042000	1.552330000
H	1.972473000	0.199394000	-1.311080000
H	-0.068637000	-1.641347000	2.802977000
H	1.236146000	-3.034231000	4.351225000
H	3.591913000	-3.582433000	3.890464000
H	4.696542000	-2.691613000	1.855331000
H	3.635709000	-2.979117000	-1.002448000
H	5.522706000	-2.122975000	-2.028760000
H	2.544154000	-1.948221000	-1.910407000
H	4.491744000	-1.295925000	-3.188147000
H	5.466289000	-1.095264000	0.264867000

Strychnobaillonine (Conformer 2, *E* = -1920.1268095 Hartree), solvent: Chloroform

O	1.227874000	2.676372000	1.844448000
O	-1.105533000	3.431972000	0.753503000
N	-2.303185000	1.612026000	0.129674000
N	-4.644655000	-2.381604000	-0.234577000
N	1.116511000	-0.750388000	0.313438000
N	5.200043000	-0.127173000	-1.458191000
C	-5.370853000	3.628878000	0.822137000
C	-6.300660000	2.697782000	0.381136000
C	-5.878538000	1.463535000	-0.104130000
C	-4.525916000	1.183204000	-0.150890000
C	-3.605281000	2.130795000	0.286421000
C	-4.005752000	3.363401000	0.782924000
C	-3.830405000	-0.087828000	-0.575951000
C	-2.363960000	0.391091000	-0.707575000
C	-1.146729000	2.283971000	0.331855000
C	0.095888000	1.476028000	0.010690000
C	-0.117201000	0.017270000	0.460958000
C	-1.228921000	-0.616271000	-0.404157000
C	-4.363383000	-0.691215000	-1.887523000
C	-5.289113000	-1.811860000	-1.413520000
C	-4.082491000	-1.230734000	0.489270000
C	-2.815874000	-1.613084000	1.240478000
C	-1.724791000	-1.935738000	0.215513000
C	-0.057217000	-3.231726000	-2.029717000
C	-1.515759000	-3.469323000	-1.775553000
C	-2.250395000	-2.941185000	-0.799828000
C	-3.655820000	-3.423501000	-0.531715000
C	1.372128000	2.162179000	0.534689000
C	2.538924000	1.180932000	0.595208000

C	2.277562000	-0.052167000	-0.264967000
C	1.619126000	-1.607840000	1.290435000
C	3.421315000	-1.091555000	-0.174381000
C	2.973688000	-1.875257000	1.037144000
C	0.984387000	-2.169770000	2.389749000
C	1.715339000	-3.031914000	3.207179000
C	3.043561000	-3.324199000	2.944158000
C	3.680241000	-2.733858000	1.849553000
C	3.924074000	1.803488000	0.376987000
C	4.945989000	0.761526000	0.826628000
C	4.827535000	-0.443350000	-0.078679000
C	4.829430000	-1.351618000	-2.174062000
C	3.556440000	-1.920196000	-1.476632000
C	4.503421000	1.044323000	-2.022963000
C	4.233685000	2.181274000	-1.052722000
C	4.271554000	3.435052000	-1.500955000
C	3.939783000	4.691593000	-0.756006000
H	-3.284231000	4.087089000	1.118922000
H	-2.508871000	-0.785549000	1.882162000
H	-4.838249000	-0.865746000	1.185276000
H	-6.257789000	-1.403691000	-1.118302000
H	-4.855791000	0.055056000	-2.508018000
H	-3.612051000	-4.092257000	0.332587000
H	-6.603698000	0.735804000	-0.442609000
H	-7.355074000	2.930136000	0.415557000
H	-5.708154000	4.583262000	1.200494000
H	0.172693000	1.464432000	-1.083092000
H	-0.412502000	0.023058000	1.515227000
H	0.433560000	-2.733424000	-1.195576000
H	0.449067000	-4.180928000	-2.204701000

H	-2.245376000	0.733623000	-1.738856000
H	-0.870125000	-2.398083000	0.707659000
H	-4.019177000	-4.022847000	-1.364335000
H	-2.000772000	-4.179716000	-2.438107000
H	-3.536751000	-1.120651000	-2.455685000
H	-5.468922000	-2.576755000	-2.164959000
H	-3.018531000	-2.462001000	1.892385000
H	-0.750195000	-0.839189000	-1.356429000
H	0.100141000	-2.623930000	-2.924435000
H	1.604529000	2.977616000	-0.159785000
H	0.509198000	3.319331000	1.808235000
H	3.589241000	4.510160000	0.256266000
H	4.807469000	5.350622000	-0.705771000
H	3.159134000	5.242214000	-1.282628000
H	4.562852000	3.583506000	-2.536936000
H	5.102389000	1.422713000	-2.850917000
H	3.546967000	0.736868000	-2.464813000
H	4.756692000	0.471945000	1.860624000
H	5.960171000	1.154845000	0.764691000
H	3.995475000	2.682007000	1.014170000
H	2.537614000	0.817497000	1.627867000
H	2.061060000	0.212683000	-1.297613000
H	-0.046442000	-1.946708000	2.626119000
H	1.230391000	-3.474194000	4.066065000
H	3.589807000	-3.995261000	3.590309000
H	4.723123000	-2.944933000	1.649878000
H	3.683245000	-2.977017000	-1.250466000
H	5.645868000	-2.067252000	-2.084807000
H	2.663191000	-1.824663000	-2.092553000
H	4.682110000	-1.145416000	-3.231718000

H	5.516926000	-1.223813000	0.255501000

Equilibrium geometries of molecules from set **2** calculated at the **CCSD** level of theory with **6-31G(2d,2p)** basis set within the IEF-PCM model. Cartesian coordinates are given for standard orientation in form (atomic label, X, Y, Z). The X, Y, Z are given in Å.

Acetaldehyde

C	0.000000	0.461720	0.000000
C	0.936593	-0.716002	0.000000
O	-1.206757	0.381543	0.000000
H	0.495504	1.448541	0.000000
H	1.581015	-0.663898	-0.878194
H	1.581015	-0.663898	0.878194
H	0.376958	-1.647392	0.000000

Acetonitrile

C	0.000000	0.000000	-1.185190
C	0.000000	0.000000	0.281229
N	0.000000	0.000000	1.439499
H	0.000000	1.023229	-1.550909
H	-0.886142	-0.511614	-1.550909
H	0.886142	-0.511614	-1.550909

Cyclopropane

C	0.000000	0.871934	0.000000
C	0.755117	-0.435967	0.000000
C	-0.755117	-0.435967	0.000000
H	0.000000	1.453120	-0.910237
H	-0.000000	1.453120	0.910237
H	1.258439	-0.726560	0.910237

H	1.258439	-0.726560	-0.910237
H	-1.258439	-0.726560	0.910237
H	-1.258439	-0.726560	-0.910237

DMAc

C	-0.196369	-0.753018	0.000000
C	1.037745	-1.637162	0.000000
C	1.290938	1.247139	0.000000
C	-1.142268	1.484720	0.000000
O	-1.315823	-1.250284	0.000000
N	0.000000	0.591202	0.000000
H	1.649496	-1.465785	-0.883404
H	1.649496	-1.465785	0.883404
H	0.696822	-2.667137	0.000000
H	1.393903	1.877123	0.885159
H	2.098977	0.526684	0.000000
H	1.393903	1.877123	-0.885159
H	-1.124177	2.120365	0.886579
H	-1.124177	2.120365	-0.886579
H	-2.047929	0.890828	0.000000

Fluorobenzene

C	0.000000	0.000000	0.929923
C	0.000000	-1.214211	0.260878
C	0.000000	-1.206569	-1.133397
C	0.000000	0.000000	-1.833006
C	0.000000	1.206569	-1.133397
C	0.000000	1.214211	0.260878
F	0.000000	0.000000	2.276262
H	0.000000	-2.133756	0.827311

H	0.000000	-2.145326	-1.669364
H	0.000000	0.000000	-2.913533
H	0.000000	2.145326	-1.669364
H	0.000000	2.133756	0.827311

Isoxazole

C	0.000000	1.123037	0.000000
C	-1.126888	0.372500	0.000000
C	-0.617099	-0.961834	0.000000
O	1.095406	0.342190	0.000000
N	0.689876	-0.990524	0.000000
H	0.181221	2.182666	0.000000
H	-2.148340	0.701961	0.000000
H	-1.161340	-1.890698	0.000000

Norbornadiene

C	-1.238757	-0.669193	-0.520361
C	-1.238757	0.669193	-0.520361
C	0.000000	1.119991	0.276589
C	1.238757	0.669193	-0.520361
C	1.238757	-0.669193	-0.520361
C	0.000000	-1.119991	0.276589
C	0.000000	-0.000000	1.350857
H	-1.924137	-1.331948	-1.025563
H	-1.924137	1.331948	-1.025563
H	0.000000	2.149675	0.619319
H	1.924137	1.331948	-1.025563
H	1.924137	-1.331948	-1.025563
H	0.000000	-2.149675	0.619319
H	-0.899966	0.000000	1.964032

H	0.899966	0.000000	1.964032

Oxetane

C	0.000000	1.037163	0.062823
C	0.000000	0.000000	-1.077468
C	0.000000	-1.037163	0.062823
O	0.000000	0.000000	1.074055
H	0.889372	1.663745	0.129259
H	-0.889372	1.663745	0.129259
H	0.889660	0.000000	-1.699276
H	-0.889660	0.000000	-1.699276
H	0.889372	-1.663745	0.129259
H	-0.889372	-1.663745	0.129259

Pyridine

C	-1.138373	0.720088	0.000000
C	-1.195578	-0.672035	0.000000
C	0.000000	-1.385072	0.000000
C	1.195579	-0.672028	0.000000
C	1.138374	0.720093	0.000000
N	-0.000002	1.422632	0.000000
H	-2.054390	1.297029	0.000000
H	-2.150787	-1.176382	0.000000
H	0.000013	-2.466008	0.000000
H	2.150784	-1.176380	0.000000
H	2.054387	1.297041	0.000000

Equilibrium geometries of molecules from set **2** calculated at the **CCSD** level of theory with **6-311G(3df,3pd)** basis set within the IEF-PCM model. Cartesian coordinates are given for standard orientation in form (atomic label, X, Y, Z). The X, Y, Z are given in Å.

Acetaldehyde

C	-0.000000	0.460349	-0.000000
C	0.930771	-0.711260	-0.000000
O	-1.199886	0.376788	0.000000
H	0.485980	1.450602	-0.000000
H	1.576501	-0.657218	-0.877243
H	1.576501	-0.657218	0.877243
H	0.375485	-1.645007	0.000000

Acetonitrile

C	-0.000000	-0.000000	-1.178228
C	0.000000	0.000000	0.280563
N	0.000000	0.000000	1.431646
H	0.000000	1.022754	-1.545176
H	-0.885731	-0.511377	-1.545176
H	0.885731	-0.511377	-1.545176

Cyclopropane

C	-0.000000	0.866252	0.000000
C	0.750196	-0.433126	-0.000000
C	-0.750196	-0.433126	0.000000
H	-0.000000	1.447740	-0.909858
H	-0.000000	1.447740	0.909858
H	1.253779	-0.723870	0.909858
H	1.253779	-0.723870	-0.909858
H	-1.253779	-0.723870	0.909858
H	-1.253779	-0.723870	-0.909858

DMAc

C	-0.196253	-0.751514	-0.000000
C	1.029122	-1.636981	-0.000000
C	1.289656	1.241007	0.000000
C	-1.135929	1.484538	0.000000
O	-1.312726	-1.245453	-0.000000
N	0.000000	0.588912	0.000000
H	1.643977	-1.465205	-0.882103
H	1.643977	-1.465205	0.882103
H	0.686016	-2.665921	-0.000000
H	1.391608	1.873958	0.883993
H	2.098303	0.521157	0.000000
H	1.391608	1.873958	-0.883993
H	-1.112427	2.122817	0.885347
H	-1.112427	2.122817	-0.885347
H	-2.048410	0.900562	-0.000000

Fluorobenzene

C	-0.000000	0.000000	0.924696
C	-0.000000	-1.212383	0.262074
C	-0.000000	-1.199866	-1.126763
C	0.000000	-0.000000	-1.826609
C	0.000000	1.199866	-1.126763
C	0.000000	1.212383	0.262074
F	-0.000000	0.000000	2.263128
H	-0.000000	-2.132934	0.826366
H	-0.000000	-2.138809	-1.663120
H	0.000000	-0.000000	-2.906892
H	0.000000	2.138809	-1.663120
H	0.000000	2.132934	0.826366

Isoxazole

C	0.000000	1.118328	-0.000000
C	-1.121109	0.367941	0.000000
C	-0.611670	-0.958955	0.000000
O	1.085595	0.339163	-0.000000
N	0.688854	-0.979720	-0.000000
H	0.182179	2.178238	-0.000000
H	-2.143594	0.695151	0.000000
H	-1.148655	-1.892534	0.000000

Norbornadiene

C	-1.233505	-0.666311	-0.518687
C	-1.233505	0.666311	-0.518687
C	0.000000	1.115648	0.274144
C	1.233505	0.666311	-0.518687
C	1.233505	-0.666311	-0.518687
C	-0.000000	-1.115648	0.274144
C	-0.000000	-0.000000	1.346669
H	-1.919270	-1.333015	-1.019359
H	-1.919270	1.333015	-1.019359
H	0.000000	2.146756	0.614759
H	1.919270	1.333015	-1.019359
H	1.919270	-1.333015	-1.019359
H	-0.000000	-2.146756	0.614759
H	-0.898302	-0.000000	1.963325
H	0.898302	0.000000	1.963325

Oxetane

C	0.000000	1.031647	0.064029
C	0.000000	0.000000	-1.071226
C	-0.000000	-1.031647	0.064029

O	-0.000000	-0.000000	1.064117
H	0.889641	1.657444	0.134301
H	-0.889641	1.657444	0.134301
H	0.888209	0.000000	-1.695569
H	-0.888209	-0.000000	-1.695569
H	0.889641	-1.657444	0.134301
H	-0.889641	-1.657444	0.134301

Pyridine

C	-1.137466	0.718094	0.000000
C	-1.191900	-0.668801	0.000000
C	-0.000003	-1.377220	-0.000000
C	1.191899	-0.668810	-0.000000
C	1.137464	0.718083	-0.000000
N	-0.000000	1.411613	0.000000
H	-2.051497	1.299026	0.000000
H	-2.146332	-1.174500	0.000000
H	0.000041	-2.458372	-0.000000
H	2.146316	-1.174542	-0.000000
H	2.051508	1.299021	-0.000000

Equilibrium geometries of molecules from set **2** calculated at the **CCSD** level of theory with **6-311G(d,p)** basis set within the IEF-PCM model. Cartesian coordinates are given for standard orientation in form (atomic label, X, Y, Z). The X, Y, Z are given in Å.

Acetaldehyde

C	0.000000	0.463607	0.000000
C	0.935905	-0.717581	0.000000
O	-1.205747	0.381537	0.000000
H	0.494927	1.455846	0.000000
H	1.582677	-0.666335	-0.883434

H	1.582677	-0.666335	0.883434
H	0.370259	-1.651630	0.000000

Acetonitrile

C	0.000000	0.000000	-1.185526
C	0.000000	0.000000	0.281754
N	0.000000	0.000000	1.440289
H	0.000000	1.028599	-1.553131
H	-0.890793	-0.514300	-1.553131
H	0.890793	-0.514300	-1.553131

Cyclopropane

C	0.000000	0.871757	0.000000
C	0.754964	-0.435879	0.000000
C	-0.754964	-0.435879	0.000000
H	0.000000	1.457524	-0.913782
H	0.000000	1.457524	0.913782
H	1.262253	-0.728762	0.913782
H	1.262253	-0.728762	-0.913782
H	-1.262253	-0.728762	0.913782
H	-1.262253	-0.728762	-0.913782

DMAc

C	-0.198206	-0.757939	0.000000
C	1.038381	-1.644257	0.000000
C	1.294221	1.252266	0.000000
C	-1.143731	1.490065	0.000000
O	-1.318126	-1.254419	0.000000
N	0.000000	0.591821	0.000000
H	1.654938	-1.468176	-0.886976

H	1.654938	-1.468176	0.886976
H	0.695782	-2.679079	0.000000
H	1.392962	1.887929	0.889184
H	2.109917	0.532217	0.000000
H	1.392962	1.887929	-0.889184
H	-1.121823	2.130576	0.890700
H	-1.121823	2.130576	-0.890700
H	-2.056837	0.897998	0.000000

Fluorobenzene

C	0.000000	0.000000	0.931917
C	0.000000	-1.218402	0.262425
C	0.000000	-1.210263	-1.135721
C	0.000000	0.000000	-1.836776
C	0.000000	1.210263	-1.135721
C	0.000000	1.218402	0.262425
F	0.000000	0.000000	2.280118
H	0.000000	-2.143171	0.829603
H	0.000000	-2.152938	-1.674649
H	0.000000	0.000000	-2.922279
H	0.000000	2.152938	-1.674649
H	0.000000	2.143171	0.829603

Isoxazole

C	0.000000	1.124974	0.000000
C	-1.128450	0.370708	0.000000
C	-0.614968	-0.964287	0.000000
O	1.091523	0.339984	0.000000
N	0.693451	-0.984649	0.000000
H	0.185079	2.188682	0.000000

H	-2.155794	0.697125	0.000000
H	-1.155117	-1.901507	0.000000

Norbornadiene

C	-1.240384	-0.670980	-0.521538
C	-1.240384	0.670980	-0.521538
C	0.000000	1.122034	0.275936
C	1.240384	0.670980	-0.521538
C	1.240384	-0.670980	-0.521538
C	0.000000	-1.122034	0.275936
C	0.000000	0.000000	1.352364
H	-1.929988	-1.340508	-1.023389
H	-1.929988	1.340508	-1.023389
H	0.000000	2.157688	0.619483
H	1.929988	1.340508	-1.023389
H	1.929988	-1.340508	-1.023389
H	0.000000	-2.157688	0.619483
H	-0.902039	0.000000	1.973049
H	0.902039	0.000000	1.973049

Oxetane

C	0.000000	1.034759	0.064984
C	0.000000	0.000000	-1.077502
C	0.000000	-1.034759	0.064984
O	0.000000	0.000000	1.071035
H	0.894694	1.663568	0.131243
H	-0.894694	1.663568	0.131243
H	0.894105	0.000000	-1.704025
H	-0.894105	0.000000	-1.704025
H	0.894694	-1.663568	0.131243

H -0.894694 -1.663568 0.131243

Pyridine

C -1.141793 0.722665 0.000000

C -1.198528 -0.673828 0.000000

C 0.000000 -1.389173 0.000000

C 1.198529 -0.673821 0.000000

C 1.141793 0.722670 0.000000

N -0.000002 1.424727 0.000000

H -2.060001 1.306012 0.000000

H -2.158264 -1.180445 0.000000

H 0.000011 -2.475299 0.000000

H 2.158258 -1.180451 0.000000

H 2.060000 1.306022 0.000000

Equilibrium geometries of molecules from set **2** calculated at the **CCSD** level of theory with **pecG-1** basis set within the IEF-PCM model. Cartesian coordinates are given for standard orientation in form (atomic label, X, Y, Z). The X, Y, Z are given in Å.

Acetaldehyde

C 0.000000 0.463716 0.000000

C 0.932964 -0.715081 0.000000

O -1.202737 0.380306 0.000000

H 0.495239 1.451488 0.000000

H 1.579386 -0.670284 -0.877432

H 1.579386 -0.670284 0.877432

H 0.370102 -1.645177 0.000000

Acetonitrile

C 0.000000 0.000000 -1.180807

C 0.000000 0.000000 0.279552

N	0.000000	0.000000	1.436628
H	0.000000	1.022054	-1.549621
H	-0.885124	-0.511027	-1.549621
H	0.885124	-0.511027	-1.549621

Cyclopropane

C	0.000000	0.869449	0.000000
C	0.752965	-0.434725	0.000000
C	-0.752965	-0.434725	0.000000
H	0.000000	1.451247	-0.909572
H	0.000000	1.451247	0.909572
H	1.256816	-0.725623	0.909572
H	1.256816	-0.725623	-0.909572
H	-1.256816	-0.725623	0.909572
H	-1.256816	-0.725623	-0.909572

DMAc

C	-0.193965	-0.757191	0.000000
C	1.042716	-1.632531	0.000000
C	1.284965	1.258012	0.000000
C	-1.150743	1.473173	0.000000
O	-1.307284	-1.255123	0.000000
N	0.000000	0.593569	0.000000
H	1.658400	-1.457444	-0.881305
H	1.658400	-1.457444	0.881305
H	0.709630	-2.665093	0.000000
H	1.381429	1.890528	0.884375
H	2.100130	0.545984	0.000000
H	1.381429	1.890528	-0.884375
H	-1.140191	2.111113	0.885208
H	-1.140191	2.111113	-0.885208

H -2.048601 0.867943 0.000000

Fluorobenzene

C 0.000000 0.000000 0.928855

C 0.000000 -1.213521 0.260620

C 0.000000 -1.205151 -1.132023

C 0.000000 0.000000 -1.830796

C 0.000000 1.205151 -1.132023

C 0.000000 1.213521 0.260620

F 0.000000 0.000000 2.274078

H 0.000000 -2.134011 0.824806

H 0.000000 -2.143147 -1.668474

H 0.000000 0.000000 -2.910890

H 0.000000 2.143147 -1.668474

H 0.000000 2.134011 0.824806

Isoxazole

C 0.000000 1.119964 0.000000

C -1.124597 0.367849 0.000000

C -0.615653 -0.961612 0.000000

O 1.092781 0.343588 0.000000

N 0.690059 -0.984667 0.000000

H 0.174025 2.179694 0.000000

H -2.145636 0.696926 0.000000

H -1.159548 -1.889864 0.000000

Norbornadiene

C -1.239716 -0.667944 -0.519879

C -1.239716 0.667944 -0.519879

C 0.000000 1.120438 0.273944

C 1.239716 0.667944 -0.519879

C	1.239716	-0.667944	-0.519879
C	0.000000	-1.120438	0.273944
C	0.000000	0.000000	1.351156
H	-1.927320	-1.331094	-1.020697
H	-1.927320	1.331094	-1.020697
H	0.000000	2.150483	0.614995
H	1.927320	1.331094	-1.020697
H	1.927320	-1.331094	-1.020697
H	0.000000	-2.150483	0.614995
H	-0.897710	0.000000	1.967812
H	0.897710	0.000000	1.967812

Oxetane

C	0.000000	1.033913	0.065498
C	0.000000	0.000000	-1.076038
C	0.000000	-1.033913	0.065498
O	0.000000	0.000000	1.069855
H	0.887117	1.664779	0.128598
H	-0.887117	1.664779	0.128598
H	0.887672	0.000000	-1.701491
H	-0.887672	0.000000	-1.701491
H	0.887117	-1.664779	0.128598
H	-0.887117	-1.664779	0.128598

Pyridine

C	-1.139643	0.718884	0.000000
C	-1.194838	-0.670878	0.000000
C	0.000000	-1.381747	0.000000
C	1.194839	-0.670872	0.000000
C	1.139644	0.718888	0.000000

N	-0.000002	1.419767	0.000000
H	-2.054838	1.295507	0.000000
H	-2.148865	-1.176343	0.000000
H	0.000007	-2.462350	0.000000
H	2.148861	-1.176346	0.000000
H	2.054835	1.295517	0.000000

Equilibrium geometries of molecules from set **2** calculated at the **CCSD** level of theory with **pecG-2** basis set within the IEF-PCM model. Cartesian coordinates are given for standard orientation in form (atomic label, X, Y, Z). The X, Y, Z are given in Å.

Acetaldehyde

C	0.000000	0.457869	0.000000
C	-0.930567	-0.708183	0.000000
O	1.199641	0.374667	0.000000
H	-0.482983	1.443960	0.000000
H	-1.573892	-0.648953	0.873618
H	-1.573892	-0.648953	-0.873618
H	-0.382957	-1.641501	0.000000

Acetonitrile

C	0.000000	0.000000	-1.175307
C	0.000000	0.000000	0.279622
N	0.000000	0.000000	1.428249
H	0.000000	1.019038	-1.541209
H	-0.882512	-0.509519	-1.541209
H	0.882512	-0.509519	-1.541209

Cyclopropane

C	0.000000	0.864330	0.000000
C	0.748532	-0.432165	0.000000

C	-0.748532	-0.432165	0.000000
H	0.000000	1.442281	-0.906235
H	0.000000	1.442281	0.906235
H	1.249052	-0.721141	0.906235
H	1.249052	-0.721141	-0.906235
H	-1.249052	-0.721141	0.906235
H	-1.249052	-0.721141	-0.906235

DMAc

C	0.564688	0.528706	0.000000
C	0.000000	1.927888	0.000000
C	-1.749052	-0.361089	0.000000
C	0.169679	-1.857745	0.000000
O	1.773143	0.354813	0.000000
N	-0.312681	-0.496303	0.000000
H	-0.610672	2.106816	-0.878064
H	-0.610672	2.106816	0.878064
H	0.831807	2.616801	0.000000
H	-2.168145	-0.839960	0.881871
H	-2.049125	0.674380	0.000000
H	-2.168145	-0.839960	-0.881871
H	-0.190092	-2.380931	0.882265
H	-0.190092	-2.380931	-0.882265
H	1.248313	-1.850577	0.000000

Fluorobenzene

C	0.000000	0.000000	0.920366
C	0.000000	-1.208443	0.259519
C	0.000000	-1.198247	-1.125589
C	0.000000	0.000000	-1.821004

C	0.000000	1.198247	-1.125589
C	0.000000	1.208443	0.259519
F	0.000000	0.000000	2.263567
H	0.000000	-2.125618	0.821306
H	0.000000	-2.132922	-1.660343
H	0.000000	0.000000	-2.897366
H	0.000000	2.132922	-1.660343
H	0.000000	2.125618	0.821306

Isoxazole

C	0.000000	1.115942	0.000000
C	-1.117608	0.368110	0.000000
C	-0.611211	-0.955144	0.000000
O	1.085387	0.338108	0.000000
N	0.685753	-0.980351	0.000000
H	0.178952	2.172144	0.000000
H	-2.136348	0.692384	0.000000
H	-1.153061	-1.880391	0.000000

Norbornadiene

C	-1.231930	-0.663806	-0.516849
C	-1.231930	0.663806	-0.516849
C	0.000000	1.113443	0.272266
C	1.231930	0.663806	-0.516849
C	1.231930	-0.663806	-0.516849
C	0.000000	-1.113443	0.272266
C	0.000000	0.000000	1.343075
H	-1.917058	-1.326844	-1.014340
H	-1.917058	1.326844	-1.014340
H	0.000000	2.139839	0.612036

H	1.917058	1.326844	-1.014340
H	1.917058	-1.326844	-1.014340
H	0.000000	-2.139839	0.612036
H	-0.894735	0.000000	1.956021
H	0.894735	0.000000	1.956021

Oxetane

C	0.000000	1.030544	0.062571
C	0.000000	0.000000	-1.068868
C	0.000000	-1.030544	0.062571
O	0.000000	0.000000	1.064911
H	0.886571	1.652417	0.130125
H	-0.886571	1.652417	0.130125
H	0.885321	0.000000	-1.688718
H	-0.885321	0.000000	-1.688718
H	0.886571	-1.652417	0.130125
H	-0.886571	-1.652417	0.130125

Pyridine

C	-1.133957	0.715133	0.000000
C	-1.189210	-0.667400	0.000000
C	0.000000	-1.372281	0.000000
C	1.189211	-0.667393	0.000000
C	1.133958	0.715138	0.000000
N	-0.000002	1.409359	0.000000
H	-2.044134	1.293221	0.000000
H	-2.139947	-1.171009	0.000000
H	0.000010	-2.449122	0.000000
H	2.139943	-1.171013	0.000000
H	2.044132	1.293232	0.000000

Table S1. ^{13}C NMR shielding constants of **12-28-oxaircinal A** calculated at the GIAO-DFT(B97-2)/pecS-2 level using the equilibrium geometries obtained within the DFT(M06-2X) method with different basis sets.

№[1]	cc-pVQZ	6-31G(2d,2p)	pecG-1	6-311G(3df,3pd)	pecG-2	cc-pVDZ	cc-pVTZ	6-311G(d,p)
5	138.92	138.31	138.73	138.71	138.91	137.76	138.82	137.94
6	101.82	101.05	101.03	101.66	101.76	100.04	101.71	100.58
7	102.25	101.82	102.14	102.16	102.25	101.10	102.13	101.36
8	143.57	142.82	142.78	143.36	143.52	141.84	143.44	142.78
9	132.27	131.63	131.71	132.03	132.25	130.49	132.06	131.48
10	118.42	117.49	117.74	118.22	118.37	116.57	118.23	117.41
11	113.56	112.73	112.95	113.36	113.54	111.20	113.22	112.64
12	84.37	83.86	84.29	84.34	84.29	82.85	84.14	83.41
13	149.00	148.52	148.52	148.96	149.00	147.24	148.98	148.08
14	32.64	32.08	32.12	32.65	32.64	31.01	32.78	31.82
15	15.91	16.07	17.41	16.51	15.99	14.74	15.95	15.92
16	142.00	141.37	141.40	141.89	141.99	140.19	141.89	141.39
17	134.47	133.46	133.67	134.21	134.43	131.91	134.10	133.17
18	155.23	154.60	154.64	155.01	155.22	153.35	155.08	154.45
19	42.97	42.53	42.82	43.02	42.96	40.97	42.94	42.24
20	130.10	129.25	129.48	129.89	130.09	127.81	129.82	129.05
21	159.39	158.69	158.83	159.26	159.38	157.43	159.24	158.58
22	156.82	156.25	156.36	156.66	156.84	154.82	156.64	155.99
23	-16.17	-16.24	-15.31	-15.60	-16.25	-17.15	-16.49	-16.32
24	52.11	51.49	51.70	52.02	52.08	49.97	51.98	51.13
25	158.31	157.59	157.77	158.13	158.31	156.17	158.12	157.46
26	156.57	155.94	156.02	156.38	156.56	154.55	156.40	155.65
27	48.34	47.94	48.23	48.39	48.37	46.36	48.29	47.71
28	155.46	154.77	155.00	155.31	155.45	153.32	155.25	154.57
29	155.32	154.65	154.66	155.16	155.31	153.32	155.15	154.37
30	41.38	40.76	40.92	41.34	41.36	39.08	41.26	40.33

[1] Numbers of carbon atoms correspond to the follow-up numbers of carbon atoms in all equilibrium geometries of 12-28-oxaircinal A given above.

Table S2-A. ^{13}C NMR shielding constants of **anabsinthin (conf. 1)** calculated at the GIAO-DFT(B97-2)/pecS-2 level using the equilibrium geometries obtained within the DFT(M06-2X) method with different basis sets.

№ [1]	cc-pVQZ	6-31G(2d,2p)	pecG-1	6-311G(3df,3pd)	pecG-2	cc-pVDZ	cc-pVTZ	6-311G(d,p)
7	118.12	117.72	118.18	117.94	118.14	117.40	118.12	117.37
8	117.54	117.34	117.50	117.52	117.59	116.54	117.49	116.97
9	138.91	137.99	138.03	138.61	138.88	137.23	138.74	137.83
10	91.41	91.13	91.61	91.42	91.38	90.46	91.30	90.61
11	127.07	126.40	126.65	126.81	127.05	125.65	126.87	126.26
12	138.09	137.79	137.95	138.08	138.13	137.01	138.01	137.53
13	101.45	101.14	101.71	101.41	101.47	100.53	101.28	100.74
14	147.68	147.05	147.15	147.58	147.67	145.90	147.58	146.91
15	97.53	97.48	98.19	97.75	97.51	96.57	97.29	96.86
16	122.57	121.59	121.82	122.32	122.63	120.83	122.43	121.60
17	28.88	29.34	30.52	29.23	28.77	28.50	28.90	28.86
18	132.16	131.42	131.41	132.10	132.13	130.59	132.17	131.44
19	142.82	142.04	142.10	142.61	142.78	140.73	142.64	141.84
20	41.21	40.02	40.29	40.97	41.20	38.89	41.02	40.26
21	166.65	165.96	166.02	166.49	166.61	164.48	166.47	165.83
22	156.05	155.43	155.61	155.78	156.09	154.13	155.73	155.47
23	105.08	105.76	106.51	105.34	105.08	105.51	105.04	105.04
24	157.12	156.55	156.65	156.98	157.08	155.08	156.92	156.50
25	138.11	137.58	137.73	138.08	138.10	136.68	138.01	137.50
26	98.31	98.21	98.87	98.55	98.31	97.22	98.11	97.74
27	165.11	164.23	164.38	164.79	165.06	162.78	164.76	164.24
28	138.70	137.81	137.73	138.33	138.70	136.44	138.43	137.79
29	-3.59	-4.00	-3.24	-3.16	-3.61	-5.28	-4.00	-4.06
30	131.76	131.08	131.06	131.56	131.76	130.23	131.59	131.00
31	157.35	156.21	156.21	157.09	157.37	154.80	157.14	156.18
32	153.29	153.01	153.19	153.19	153.27	151.57	153.12	152.79
33	171.43	170.68	170.67	171.26	171.44	169.20	171.22	170.46
34	138.64	138.15	138.39	138.60	138.59	137.33	138.52	137.87
35	-3.77	-4.17	-3.40	-3.30	-3.83	-5.38	-4.18	-4.23
36	171.63	170.68	170.63	171.36	171.60	169.18	171.34	170.59

[1] Numbers of carbon atoms correspond to the follow-up numbers of carbon atoms in all equilibrium geometries of anabsinthin (conf. 1) given above.

Table S2-B. ^{13}C NMR shielding constants of **anabsinthin (conf. 2)** calculated at the GIAO-DFT(B97-2)/pecS-2 level using the equilibrium geometries obtained within the DFT(M06-2X) method with different basis sets.

№ [1]	cc-pVQZ	6-31G(2d,2p)	pecG-1	6-311G(3df,3pd)	pecG-2	cc-pVDZ	cc-pVTZ	6-311G(d,p)
7	117.74	117.42	117.87	117.61	117.81	117.07	117.79	117.12
8	118.07	117.59	117.96	117.84	118.15	116.88	117.81	117.38
9	139.13	138.39	138.41	138.91	139.09	137.61	139.03	138.24
10	91.46	91.17	91.71	91.38	91.46	90.49	91.26	90.61
11	125.58	125.18	125.38	125.53	125.57	124.55	125.57	124.84
12	138.25	137.61	138.06	138.01	138.26	136.90	138.02	137.53
13	101.35	101.26	101.70	101.51	101.33	100.65	101.37	100.72
14	147.07	146.33	146.40	146.91	147.05	145.17	146.92	146.25
15	97.46	97.41	98.08	97.70	97.44	96.51	97.24	96.90
16	123.14	122.87	122.71	123.33	123.21	122.22	123.38	122.74
17	28.09	27.17	27.60	28.02	27.98	25.91	27.93	26.80
18	132.37	131.48	131.55	132.19	132.36	130.66	132.26	131.59
19	142.89	142.03	142.13	142.59	142.85	140.72	142.62	141.86
20	43.18	42.39	42.96	43.05	43.21	41.44	43.02	42.34
21	166.39	165.66	165.73	166.20	166.35	164.16	166.19	165.60
22	155.86	155.33	155.54	155.69	155.88	154.06	155.64	154.97
23	104.00	103.97	104.67	104.07	104.01	103.52	103.82	103.28
24	157.13	156.54	156.61	156.99	157.09	155.08	156.94	156.40
25	138.21	137.61	137.79	138.14	138.17	136.71	138.08	137.27
26	103.85	103.86	104.44	104.15	103.86	102.99	103.68	103.41
27	165.04	164.40	164.40	164.93	164.99	162.97	164.90	164.35
28	141.28	140.62	140.82	141.07	141.31	139.21	141.11	140.57
29	-3.58	-4.00	-3.22	-3.18	-3.62	-5.28	-4.02	-4.16
30	135.00	134.29	134.36	134.92	135.01	133.51	134.99	134.02
31	158.30	157.64	157.68	158.19	158.37	156.26	158.11	157.47
32	160.02	159.59	159.38	159.84	160.03	158.20	159.80	159.57
33	171.38	170.65	170.69	171.18	171.40	169.16	171.12	170.47
34	136.45	135.89	136.15	136.35	136.42	134.94	136.26	135.65
35	-3.55	-4.09	-3.33	-3.15	-3.59	-5.34	-4.02	-4.08
36	171.04	170.26	170.28	170.93	171.02	168.79	170.88	170.15

[1] Numbers of carbon atoms correspond to the follow-up numbers of carbon atoms in all equilibrium geometries of anabsinthin (conf. 2) given above.

Table S3. ^{13}C NMR shielding constants of **betulinic acid** calculated at the GIAO-DFT(B97-2)/pecS-2 level using the equilibrium geometries obtained within the DFT(M06-2X) method with different basis sets.

№ [1]	cc-pVQZ	6-31G(2d,2p)	pecG-1	6-311G(3df,3pd)	pecG-2	cc-pVDZ	cc-pVTZ	6-311G(d,p)
1	131.56	130.84	130.64	131.39	131.52	129.88	131.43	130.51
2	132.12	131.72	131.61	132.17	132.16	130.84	132.11	131.35
3	123.66	123.18	123.24	123.56	123.66	122.60	123.68	122.86
4	143.47	143.15	143.28	143.50	143.51	141.85	143.33	142.88
5	150.10	149.64	149.65	150.06	150.03	148.31	150.04	149.11
6	144.05	143.33	143.22	143.89	144.06	142.43	143.98	143.17
7	138.32	137.51	137.87	137.90	138.32	137.40	138.16	137.76
8	152.55	151.69	151.98	152.19	152.48	150.96	152.40	151.36
9	149.78	149.25	149.45	149.67	149.86	147.88	149.53	149.10
10	157.15	156.81	156.73	157.24	157.17	155.40	157.17	156.21
11	162.15	161.39	161.74	161.92	162.15	160.27	161.97	161.22
12	131.64	130.90	131.09	131.31	131.61	130.22	131.44	130.60
13	140.84	140.32	140.57	140.47	140.80	139.99	140.75	140.14
14	144.04	143.26	143.52	143.75	144.05	142.92	143.96	143.10
15	126.92	126.42	126.40	126.86	126.89	125.56	126.92	125.80
16	163.58	162.75	162.89	163.37	163.56	161.52	163.35	162.57
17	149.34	148.53	148.63	149.15	149.35	147.36	149.14	148.40
18	143.64	143.24	143.26	143.47	143.55	142.04	143.48	142.80
19	157.11	156.23	156.01	156.69	157.03	155.11	156.87	156.11
20	101.60	101.53	102.07	101.81	101.55	100.77	101.48	101.18
21	141.93	141.13	141.26	141.53	141.85	140.83	141.76	141.08
22	18.84	18.34	18.70	18.77	18.76	17.10	18.79	18.01
23	70.61	69.98	70.07	70.67	70.67	68.25	70.48	69.87
24	164.49	163.88	164.12	164.25	164.43	162.50	164.23	163.67
25	-0.79	-1.54	-0.63	-0.51	-0.83	-2.71	-1.33	-1.36
28	171.39	170.63	170.86	171.13	171.31	169.69	171.30	170.21
29	168.97	167.77	167.89	168.87	168.93	166.42	168.76	168.15
30	167.16	166.51	166.57	166.99	167.04	165.03	166.96	166.26
31	157.48	156.88	156.87	157.35	157.49	155.17	157.25	156.70
32	167.98	167.44	167.80	167.89	167.98	165.81	167.82	167.42

[1] Numbers of carbon atoms correspond to the follow-up numbers of carbon atoms in all equilibrium geometries of betulinic acid given above.

Table S4-A. ^{13}C NMR shielding constants of **icajine (conf. 1)** calculated at the GIAO-DFT(B97-2)/pecS-2 level using the equilibrium geometries obtained within the DFT(M06-2X) method with different basis sets.

№ [1]	cc-pVQZ	6-31G(2d,2p)	pecG-1	6-311G(3df,3pd)	pecG-2	cc-pVDZ	cc-pVTZ	6-311G(d,p)
6	118.73	118.52	118.67	118.76	118.77	117.81	118.70	117.96
7	-20.28	-19.99	-19.02	-19.39	-19.82	-21.01	-20.07	-19.16
8	137.72	136.70	136.72	137.26	137.47	135.25	137.20	136.37
9	133.71	133.71	134.33	134.00	134.03	132.73	133.84	133.42
10	123.59	123.14	123.29	123.60	123.74	122.67	123.74	122.97
11	42.77	42.40	43.00	42.77	42.78	41.45	42.72	42.12
12	52.11	51.60	51.95	52.13	52.11	50.29	52.04	51.24
13	55.45	54.97	55.36	55.48	55.48	53.73	55.33	54.76
14	50.39	49.88	50.20	50.37	50.40	48.59	50.29	49.64
15	63.70	63.17	63.48	63.74	63.75	61.90	63.65	62.91
16	36.86	35.94	36.04	36.55	36.69	34.85	36.68	35.75
17	138.49	138.01	138.44	138.53	138.70	137.01	138.50	138.02
18	148.74	148.02	148.30	148.53	148.80	147.20	148.61	147.81
19	131.11	130.62	130.98	131.29	131.46	129.99	131.35	130.46
20	108.09	107.76	108.09	108.07	108.04	106.83	107.87	107.27
21	116.74	116.11	116.36	116.39	116.43	114.88	116.23	115.85
22	47.42	46.93	47.49	47.81	47.74	45.64	47.72	46.87
23	37.99	36.81	36.95	37.55	37.73	35.55	37.67	36.88
24	119.10	118.31	118.68	118.91	119.10	116.99	118.78	118.12
25	148.18	147.34	147.50	147.57	147.78	146.30	147.56	147.34
26	8.77	8.02	8.04	8.90	8.59	6.80	8.36	8.03
27	144.45	143.78	143.88	144.27	144.34	141.96	144.10	143.41

[1] Numbers of carbon atoms correspond to the follow-up numbers of carbon atoms in all equilibrium geometries of icajine (conf. 1) given above.

Table S4-B. ^{13}C NMR shielding constants of **icajine (conf. 2)** calculated at the GIAO-DFT(B97-2)/pecS-2 level using the equilibrium geometries obtained within the DFT(M06-2X) method with different basis sets.

№ [1]	cc-pVQZ	6-31G(2d,2p)	pecG-1	6-311G(3df,3pd)	pecG-2	cc-pVDZ	cc-pVTZ	6-311G(d,p)
6	121.85	121.56	121.91	119.98	120.09	120.71	119.94	120.99
7	-21.23	-20.91	-20.02	-19.08	-19.27	-21.92	-19.79	-20.33
8	137.99	137.23	137.61	137.12	137.26	135.94	137.03	136.91
9	131.42	131.10	131.37	132.63	132.64	130.02	132.48	130.85
10	124.25	123.76	124.07	123.95	124.13	123.27	124.11	123.72
11	42.63	42.17	42.73	42.61	42.54	41.22	42.55	41.93
12	52.36	51.81	52.22	52.01	52.05	50.51	51.91	51.52

13	55.42	54.96	55.39	55.60	55.59	53.75	55.46	54.84
14	50.67	50.17	50.49	50.65	50.68	48.90	50.56	49.95
15	63.75	63.14	63.81	63.23	63.22	61.86	63.16	63.18
16	35.99	35.12	35.26	36.24	36.40	34.03	36.36	34.88
17	138.35	137.79	138.00	139.01	139.11	136.79	138.97	137.70
18	143.05	142.20	142.18	143.97	144.19	141.39	144.06	141.95
19	136.27	135.67	136.35	134.16	134.38	134.94	134.24	135.84
20	100.27	99.55	99.68	101.73	101.62	98.49	101.46	99.23
21	114.19	113.55	113.68	116.14	115.97	112.25	115.89	113.10
22	46.03	45.14	45.57	45.90	45.94	43.59	45.81	45.25
23	29.92	29.17	29.67	29.37	29.49	28.09	29.40	29.08
24	119.18	118.49	118.78	119.47	119.52	117.22	119.36	118.18
25	140.56	139.63	139.90	138.83	139.10	138.68	138.97	139.77
26	10.18	9.70	9.99	10.77	10.46	8.55	10.21	9.52
27	144.91	144.30	144.50	144.78	144.80	142.54	144.60	143.92

[1] Numbers of carbon atoms correspond to the follow-up numbers of carbon atoms in all equilibrium geometries of icajine (conf. 2) given above.

Table S5. ^{13}C NMR shielding constants of **iguesterin** calculated at the GIAO-DFT(B97-2)/pecS-2 level using the equilibrium geometries obtained within the DFT(M06-2X) method with different basis sets.

№ [1]	cc-pVQZ	6-31G(2d,2p)	pecG-1	6-311G(3df,3pd)	pecG-2	cc-pVDZ	cc-pVTZ	6-311G(d,p)
3	139.25	138.66	138.88	139.05	139.24	138.14	139.23	138.31
4	134.75	134.09	134.42	134.44	134.78	133.52	134.57	134.00
5	138.40	137.72	137.88	138.24	138.37	136.87	138.33	137.48
6	150.69	149.90	150.19	150.40	150.68	149.31	150.56	150.01
7	153.79	152.98	153.26	153.38	153.78	151.69	153.34	152.92
8	152.21	151.44	151.59	152.01	152.19	150.19	152.03	151.26
9	146.90	146.40	146.43	146.90	146.90	145.04	146.85	146.16
10	-4.12	-4.74	-3.96	-3.82	-4.31	-6.20	-4.25	-5.25
11	135.82	135.18	135.48	135.65	135.81	134.48	135.72	135.00
12	147.12	146.46	146.55	147.06	147.08	145.13	146.98	146.40
13	154.09	153.42	153.65	153.86	154.10	152.21	153.82	153.31
14	166.61	165.79	165.97	166.44	166.58	164.25	166.38	165.82
15	144.37	143.85	143.89	144.41	144.40	142.50	144.34	143.62
16	161.49	160.86	161.06	161.31	161.52	159.35	161.23	160.80
17	151.20	150.77	150.81	151.19	151.15	149.06	151.08	150.32
18	40.54	39.75	40.16	40.45	40.43	38.52	40.42	39.48
19	8.61	7.66	7.90	8.48	8.61	6.80	8.58	7.51

20	57.28	56.38	56.73	57.22	57.26	54.89	57.16	56.38
21	60.85	60.07	60.39	60.62	60.84	58.57	60.54	59.94
22	142.22	141.66	141.98	141.77	142.21	140.11	141.61	141.55
23	51.88	51.12	51.38	51.67	51.86	50.15	51.79	50.94
24	42.73	42.16	42.69	42.93	42.70	40.92	42.61	42.05
25	159.17	158.60	158.73	159.05	159.15	157.16	158.97	158.39
26	59.83	59.36	59.32	59.66	59.71	58.04	59.75	58.99
27	62.40	62.20	62.44	62.34	62.30	61.09	62.23	61.49
28	1.52	1.41	2.02	2.02	1.55	0.63	1.41	1.27
29	31.59	31.00	31.35	31.67	31.54	29.82	31.44	30.72
30	173.36	172.72	172.94	173.28	173.35	171.36	173.17	172.65

[1] Numbers of carbon atoms correspond to the follow-up numbers of carbon atoms in all equilibrium geometries of iguesterin given above.

Table S6-A. ^{13}C NMR shielding constants of **itoaic acid (conf. 1)** calculated at the GIAO-DFT(B97-2)/pecS-2 level using the equilibrium geometries obtained within the DFT(M06-2X) method with different basis sets.

№ [1]	cc-pVQZ	6-31G(2d,2p)	pecG-1	6-311G(3df,3pd)	pecG-2	cc-pVDZ	cc-pVTZ	6-311G(d,p)
1	140.97	140.38	140.63	140.73	141.04	139.95	140.94	140.04
2	119.94	119.24	119.37	119.86	120.10	118.29	119.84	119.04
3	136.72	136.26	136.64	136.51	136.82	135.92	136.70	135.90
4	129.19	128.62	128.82	129.07	129.23	127.84	129.08	128.57
5	164.11	163.46	163.66	163.94	164.08	162.31	163.95	163.15
6	144.65	143.99	144.20	144.56	144.72	142.73	144.49	143.85
7	103.70	103.18	103.32	103.67	103.72	102.41	103.47	102.84
8	143.57	142.94	143.03	143.47	143.61	141.78	143.43	142.87
9	127.45	127.01	127.22	127.32	127.48	126.52	127.46	126.79
10	138.54	138.02	138.22	138.31	138.58	137.65	138.47	137.88
11	134.84	134.48	134.83	134.79	134.94	133.45	134.72	134.36
12	149.82	148.99	149.37	149.56	149.96	148.48	149.76	149.27
13	145.33	144.53	144.62	145.16	145.35	143.13	145.14	144.46
14	154.08	153.50	153.65	153.85	153.96	152.27	153.83	153.07
15	150.39	149.73	149.91	150.26	150.41	148.45	150.23	149.67
16	151.39	150.64	151.00	151.11	151.43	149.94	151.26	151.18
17	147.22	146.53	146.62	147.09	147.20	145.08	147.05	146.22
18	148.66	148.24	148.19	148.48	148.57	146.78	148.42	147.85
19	149.59	148.95	148.90	149.24	149.25	147.84	149.40	148.52
20	112.03	111.62	111.96	112.11	112.11	110.72	111.90	111.30
21	-1.15	-2.18	-1.52	-0.75	-1.05	-3.42	-1.59	-1.75

23	97.22	97.15	98.00	97.55	97.40	96.11	96.99	96.39
26	167.57	166.80	166.84	167.35	167.50	165.43	167.36	166.75
27	170.28	169.62	169.82	170.15	170.25	168.09	170.09	169.45
28	167.78	167.14	167.50	167.75	167.74	165.51	167.60	166.91
30	166.21	165.54	165.84	166.08	166.29	164.07	166.00	165.57
31	151.74	151.30	151.45	151.65	151.64	149.57	151.47	151.32
32	147.50	146.87	146.91	147.33	147.41	145.08	147.23	146.64
33	153.36	152.54	152.54	153.17	153.31	150.87	153.13	152.45
34	-1.80	-2.78	-2.08	-1.61	-1.77	-3.98	-2.31	-2.30

[1] Numbers of carbon atoms correspond to the follow-up numbers of carbon atoms in all equilibrium geometries of itoaic acid (conf. 1) given above.

Table S6-B. ^{13}C NMR shielding constants of **itoaic acid (conf. 2)** calculated at the GIAO-DFT(B97-2)/pecS-2 level using the equilibrium geometries obtained within the DFT(M06-2X) method with different basis sets.

№ [1]	cc-pVQZ	6-31G(2d,2p)	pecG-1	6-311G(3df,3pd)	pecG-2	cc-pVDZ	cc-pVTZ	6-311G(d,p)
1	140.41	139.85	140.08	140.17	140.38	139.45	140.37	140.06
2	120.50	119.90	120.02	120.42	120.54	118.92	120.40	119.66
3	136.40	136.00	136.36	136.19	136.46	135.68	136.38	135.81
4	129.65	129.04	129.15	129.49	129.59	128.11	129.51	128.84
5	163.41	162.79	163.13	163.26	163.50	161.69	163.25	162.58
6	144.41	143.79	144.05	144.33	144.44	142.54	144.24	143.76
7	102.87	102.15	102.37	102.80	102.83	101.43	102.64	101.84
8	143.24	142.65	142.72	143.16	143.21	141.47	143.09	142.51
9	125.75	125.38	125.38	125.61	125.68	124.81	125.76	124.91
10	141.58	141.04	141.28	141.33	141.65	140.62	141.52	140.69
11	137.06	136.75	137.16	136.92	136.95	135.89	136.90	136.42
12	149.55	148.95	149.28	149.32	149.60	148.51	149.50	148.86
13	147.58	146.88	146.91	147.38	147.57	145.56	147.38	146.87
14	149.58	148.77	148.84	149.41	149.54	147.49	149.39	148.53
15	145.17	144.66	144.74	145.07	145.01	143.29	144.99	144.43
16	152.52	151.84	152.19	152.26	152.51	151.28	152.39	151.51
17	150.78	150.08	150.20	150.64	150.69	148.80	150.62	150.00
18	142.83	142.34	142.40	142.67	142.74	140.81	142.60	142.07
19	149.56	149.02	149.02	149.24	149.38	147.90	149.35	148.54
20	112.03	111.61	111.92	112.11	112.02	110.71	111.90	111.34
21	-1.16	-2.21	-1.48	-0.76	-1.06	-3.43	-1.59	-1.74
23	97.14	97.08	97.92	97.48	97.19	96.03	96.92	96.23
26	167.42	166.64	166.68	167.20	167.35	165.28	167.20	166.65

27	170.18	169.56	169.73	170.07	170.16	168.01	169.99	169.46
28	169.02	168.73	169.08	169.09	168.98	166.94	168.87	168.31
30	161.59	160.92	160.95	161.58	161.53	159.23	161.43	160.93
31	152.57	152.01	152.16	152.41	152.37	150.09	152.24	151.54
32	154.52	153.89	153.89	154.35	154.45	152.20	154.27	153.71
33	149.08	148.55	148.51	148.91	149.09	146.82	148.84	148.35
34	-1.09	-1.98	-1.14	-0.89	-1.07	-3.12	-1.60	-1.66

[1] Numbers of carbon atoms correspond to the follow-up numbers of carbon atoms in all equilibrium geometries of itoaic acid (conf. 2) given above.

Table S7. ^{13}C NMR shielding constants of **matopensine** calculated at the GIAO-DFT(B97-2)/pecS-2 level using the equilibrium geometries obtained within the DFT(M06-2X) method with different basis sets.

№ [1]	cc-pVQZ	6-31G(2d,2p)	pecG-1	6-311G(3df,3pd)	pecG-2	cc-pVDZ	cc-pVTZ	6-311G(d,p)
4	115.65	115.09	115.23	115.56	115.72	114.08	115.47	114.95
5	127.44	126.95	127.23	127.24	127.53	126.57	127.46	126.89
6	139.66	138.97	139.55	139.52	139.74	138.27	139.61	138.96
7	94.71	93.90	94.43	94.69	94.72	92.91	94.40	93.65
8	112.83	112.22	112.37	112.71	112.78	111.31	112.70	111.92
9	153.40	152.75	152.91	153.27	153.40	151.97	153.29	152.54
10	153.21	152.51	152.59	153.05	153.11	151.17	152.99	152.22
11	142.11	141.69	142.50	142.03	142.47	140.68	141.96	141.93
12	38.51	37.81	38.13	38.44	38.50	36.76	38.39	37.73
13	27.83	26.90	27.38	27.72	27.83	25.86	27.64	26.93
14	125.95	125.28	125.39	125.78	125.85	124.18	125.72	124.90
15	33.39	32.70	33.19	33.30	33.39	31.45	33.27	32.45
16	126.24	125.49	125.65	126.12	126.16	124.33	126.02	125.48
17	56.74	56.21	56.53	56.66	56.74	54.93	56.61	56.11
18	71.46	70.31	70.72	71.36	71.54	69.25	71.28	70.51
19	59.07	58.59	58.74	59.00	59.00	57.00	58.92	58.31
20	60.61	59.81	60.08	60.63	60.65	58.49	60.47	59.74
21	50.25	49.67	49.93	50.14	50.26	48.47	50.15	49.45
22	166.65	165.90	166.16	166.46	166.73	164.55	166.48	166.03
46	115.65	115.09	115.23	115.56	115.72	114.08	115.47	114.95
47	127.44	126.95	127.23	127.24	127.54	126.57	127.46	126.89
48	139.66	138.97	139.55	139.52	139.74	138.27	139.61	138.96
49	94.71	93.90	94.43	94.69	94.72	92.91	94.40	93.65
50	112.83	112.22	112.37	112.71	112.78	111.31	112.70	111.92
51	153.40	152.75	152.91	153.27	153.40	151.97	153.29	152.54
52	153.21	152.51	152.59	153.05	153.11	151.17	152.99	152.22

53	142.11	141.69	142.50	142.03	142.47	140.68	141.96	141.93
54	38.51	37.81	38.13	38.44	38.50	36.76	38.39	37.73
55	27.83	26.90	27.38	27.72	27.83	25.86	27.64	26.93
56	125.95	125.28	125.39	125.78	125.85	124.18	125.72	124.90
57	33.39	32.70	33.19	33.30	33.39	31.45	33.27	32.45
58	126.24	125.49	125.65	126.12	126.16	124.33	126.02	125.48
59	56.74	56.21	56.53	56.66	56.74	54.93	56.61	56.11
60	71.46	70.31	70.72	71.36	71.54	69.25	71.28	70.51
61	59.07	58.59	58.74	59.00	59.00	57.00	58.92	58.31
62	60.61	59.81	60.08	60.63	60.65	58.49	60.47	59.74
63	50.25	49.67	49.93	50.14	50.26	48.46	50.15	49.45
64	166.65	165.90	166.16	166.46	166.73	164.55	166.48	166.03

[1] Numbers of carbon atoms correspond to the follow-up numbers of carbon atoms in all equilibrium geometries of matopensine given above.

Table S8. ^{13}C NMR shielding constants of **naucleidinal** calculated at the GIAO-DFT(B97-2)/pecS-2 level using the equilibrium geometries obtained within the DFT(M06-2X) method with different basis sets.

№ [1]	cc-pVQZ	6-31G(2d,2p)	pecG-1	6-311G(3df,3pd)	pecG-2	cc-pVDZ	cc-pVTZ	6-311G(d,p)
6	126.77	126.15	126.44	126.60	126.74	125.29	126.62	125.83
7	154.13	153.15	153.12	153.80	153.93	152.06	153.91	152.78
8	153.30	152.52	152.44	153.09	153.23	151.39	153.12	152.31
9	123.63	123.41	123.67	123.65	123.74	122.67	123.59	123.17
10	43.50	41.73	41.48	43.05	43.03	40.90	43.10	41.58
11	69.32	68.56	68.44	69.08	68.96	67.27	69.07	67.72
12	137.56	136.96	136.89	137.27	137.44	135.86	137.29	136.66
13	67.71	66.69	66.74	67.53	67.60	66.02	67.61	66.66
14	13.69	12.53	12.34	13.59	13.46	11.64	13.35	12.85
15	161.00	160.50	161.02	160.97	161.02	159.09	160.81	160.04
16	107.38	107.26	107.75	107.49	107.27	106.26	107.19	106.78
17	50.25	49.31	49.51	50.07	50.14	48.46	50.10	49.16
18	26.26	25.70	26.28	26.44	26.23	24.32	26.03	25.55
19	42.82	41.55	41.67	42.58	42.58	40.95	42.58	41.51
20	-28.84	-29.98	-29.29	-28.55	-29.18	-31.29	-29.50	-29.98
21	164.46	163.81	163.97	164.28	164.52	162.41	164.26	163.80
22	61.06	60.54	60.77	60.99	61.03	59.30	60.96	60.26
23	68.64	67.78	67.99	68.53	68.56	66.50	68.48	67.63
24	59.97	59.35	59.54	59.97	59.96	58.14	59.88	59.14
25	57.73	57.21	57.49	57.72	57.77	56.00	57.64	56.94

[1] Numbers of carbon atoms correspond to the follow-up numbers of carbon atoms in all equilibrium geometries of naucleidinal given above.

Table S9. ^{13}C NMR shielding constants of **physalin D** calculated at the GIAO-DFT(B97-2)/pecS-2 level using the equilibrium geometries obtained within the DFT(M06-2X) method with different basis sets.

№ [1]	cc-pVQZ	6-31G(2d,2p)	pecG-1	6-311G(3df,3pd)	pecG-2	cc-pVDZ	cc-pVTZ	6-311G(d,p)
1	28.53	27.71	28.20	28.66	28.67	25.92	28.48	27.57
2	145.32	144.65	144.83	145.16	145.28	143.47	145.15	144.58
3	102.51	102.49	103.06	102.62	102.59	101.90	102.51	102.08
4	123.01	122.15	122.55	122.99	123.11	121.71	123.07	122.04
5	-31.25	-31.99	-31.26	-30.88	-31.37	-33.04	-31.71	-31.94
6	49.39	48.67	48.83	49.20	49.27	47.44	49.24	48.30
7	104.55	104.02	104.48	104.48	104.53	103.32	104.39	103.94
8	151.04	150.20	150.31	150.86	151.12	149.23	150.91	150.27
9	142.06	141.46	141.54	141.76	142.05	140.69	141.96	141.40
10	139.14	138.57	138.99	139.08	139.30	137.76	139.06	138.58
12	75.08	74.73	75.09	75.13	74.95	73.83	74.88	74.09
13	-38.63	-39.46	-38.91	-38.43	-38.78	-40.88	-39.17	-39.29
14	126.90	126.51	126.75	126.78	126.93	125.78	126.83	126.40
15	96.84	96.76	97.10	96.75	96.78	96.10	96.80	96.20
16	98.91	98.99	99.45	99.00	98.97	98.44	98.88	98.68
17	144.42	143.61	143.58	144.04	144.31	142.20	144.19	143.47
18	159.38	158.20	158.41	159.11	159.30	157.24	159.19	158.41
22	148.33	148.03	148.39	148.17	148.37	147.47	148.34	147.53
23	131.16	130.56	130.83	131.06	131.35	129.71	131.12	130.63
24	119.68	119.50	120.01	119.76	119.76	118.40	119.45	119.16
25	149.69	149.25	149.28	149.53	149.68	148.02	149.56	148.88
26	104.36	104.14	104.53	104.50	104.29	103.24	104.18	103.61
27	97.60	97.37	97.81	97.63	97.55	96.52	97.51	96.75
28	-1.09	-2.14	-1.70	-0.91	-1.14	-3.35	-1.53	-1.70
31	7.38	6.75	7.43	7.75	7.27	5.46	6.98	6.85
33	161.11	160.44	160.63	160.91	161.13	159.12	160.93	160.51
35	156.45	156.14	156.46	156.46	156.54	154.67	156.23	156.05
38	167.51	167.45	167.30	167.42	167.41	165.55	167.27	166.90

[1] Numbers of carbon atoms correspond to the follow-up numbers of carbon atoms in all equilibrium geometries of physalin D given above.

Table S10-A. ^{13}C NMR shielding constants of **strychnobaillonine (conf. 1)** calculated at the GIAO-DFT(B97-2)/pecS-2 level using the equilibrium geometries obtained within the DFT(M06-2X) method with different basis sets.

№ [1]	cc-pVQZ	6-31G(2d,2p)	pecG-1	6-311G(3df,3pd)	pecG-2	cc-pVDZ	cc-pVTZ	6-311G(d,p)
7	50.76	50.21	50.54	50.74	50.75	48.96	50.64	50.04
8	54.25	53.74	54.14	54.31	54.33	52.45	54.14	53.55
9	55.62	55.00	55.30	55.53	55.58	53.81	55.49	54.85
10	35.74	35.19	35.52	35.65	35.62	34.13	35.60	34.82
11	36.95	36.24	36.67	36.75	36.89	35.19	36.83	35.99
12	63.45	62.91	63.39	63.45	63.52	61.53	63.34	62.62
13	126.54	126.01	126.26	126.36	126.58	125.64	126.54	125.75
14	113.74	113.49	113.87	113.78	113.78	112.49	113.60	113.04
15	5.69	5.06	5.42	5.96	5.69	3.87	5.35	5.14
16	135.00	134.66	134.64	134.85	134.86	133.83	134.86	134.33
17	125.77	124.96	125.07	125.59	125.73	124.10	125.56	124.77
18	131.31	130.13	130.21	131.12	131.20	129.61	131.22	130.26
19	133.28	132.70	132.91	133.25	133.38	131.45	133.10	132.58
20	126.00	125.31	125.51	125.81	125.92	124.21	125.77	125.01
21	111.57	110.95	111.29	111.48	111.45	110.03	111.40	110.59
22	158.76	158.34	158.51	158.68	158.85	156.97	158.60	158.08
23	151.92	151.42	152.05	151.78	152.02	150.53	151.82	151.34
24	170.18	169.55	169.65	170.03	170.11	167.98	169.97	169.31
25	58.61	58.31	58.42	58.56	58.63	56.52	58.45	57.94
26	35.01	34.13	34.61	34.95	34.90	33.06	34.92	34.11
27	127.53	127.06	127.17	127.50	127.51	125.75	127.32	126.84
28	112.76	112.65	113.23	112.82	112.75	111.85	112.66	112.28
29	130.79	130.26	130.29	130.55	130.74	129.28	130.77	130.15
30	112.31	111.88	111.73	112.37	112.18	110.66	112.22	111.10
31	25.49	24.30	24.32	25.26	25.28	23.41	25.32	24.22
32	126.70	126.29	126.66	126.56	126.68	125.83	126.71	125.88
33	41.79	41.12	41.29	41.65	41.75	40.03	41.67	40.77
34	73.44	72.35	72.40	73.25	73.29	71.13	73.23	72.18
35	50.93	50.45	50.76	50.89	50.95	49.16	50.81	50.19
36	61.37	60.68	60.72	61.34	61.31	59.32	61.24	60.25
37	55.61	55.07	55.24	55.50	55.56	53.74	55.48	54.70
38	150.50	150.20	150.75	150.46	150.51	149.15	150.41	149.65
39	151.86	151.70	152.20	151.90	151.86	150.13	151.77	151.37
40	118.26	117.64	117.69	118.18	118.21	116.66	118.13	117.22
41	127.30	126.62	126.74	127.13	127.24	125.41	127.05	126.24
42	141.31	140.76	140.81	141.08	141.31	139.51	141.14	140.42

43	121.98	121.24	121.71	122.02	121.89	120.04	121.81	121.14
44	37.38	36.59	36.78	37.27	37.34	35.37	37.24	36.53
45	56.09	55.69	56.26	56.21	55.97	54.09	56.02	55.55
46	169.59	169.02	169.31	169.44	169.58	167.44	169.37	168.95

[1] Numbers of carbon atoms correspond to the follow-up numbers of carbon atoms in all equilibrium geometries of strychnobaillonine (conf. 1) given above.

Table S10-B. ^{13}C NMR shielding constants of **strychnobaillonine (conf. 2)** calculated at the GIAO-DFT(B97-2)/pecS-2 level using the equilibrium geometries obtained within the DFT(M06-2X) method with different basis sets.

№ [1]	cc-pVQZ	6-31G(2d,2p)	pecG-1	6-311G(3df,3pd)	pecG-2	cc-pVDZ	cc-pVTZ	6-311G(d,p)
7	51.04	50.50	50.85	51.02	51.07	49.25	50.93	50.33
8	53.77	53.26	53.58	53.82	53.79	51.99	53.66	52.92
9	55.95	55.31	55.65	55.86	55.97	54.09	55.83	55.14
10	33.71	33.15	33.55	33.66	33.69	32.11	33.59	33.00
11	36.99	36.20	36.50	36.76	36.90	35.15	36.83	36.09
12	62.47	61.95	62.31	62.46	62.46	60.57	62.34	61.64
13	125.77	125.19	125.73	125.60	125.94	124.66	125.78	124.98
14	118.58	118.30	118.56	118.48	118.63	117.36	118.40	117.79
15	5.97	5.12	5.14	6.14	6.03	3.90	5.58	5.34
16	134.96	134.46	134.62	134.74	134.86	133.74	134.79	134.26
17	126.51	125.63	125.66	126.34	126.42	124.67	126.31	125.42
18	134.82	133.72	133.93	134.64	134.84	132.92	134.72	134.00
19	137.47	136.87	137.31	137.41	137.85	135.86	137.31	137.13
20	122.30	121.53	121.78	122.19	122.29	120.31	122.08	121.36
21	112.41	111.83	112.34	112.33	112.61	110.99	112.30	111.58
22	160.96	160.20	160.33	160.81	160.87	159.01	160.78	160.10
23	151.64	151.24	151.37	151.45	151.47	150.31	151.56	150.87
24	168.29	167.81	167.90	168.16	168.38	166.32	168.14	167.72
25	57.29	56.38	56.81	57.23	57.13	54.91	57.11	56.17
26	29.48	28.89	29.09	29.34	29.51	27.80	29.41	28.72
27	122.99	122.26	122.77	122.98	122.98	121.14	122.80	122.09
28	112.92	112.83	113.36	112.99	112.94	112.07	112.83	112.43
29	131.17	130.72	130.91	131.01	131.33	129.74	131.18	130.77
30	111.36	110.79	110.77	111.40	111.25	109.60	111.25	110.23
31	25.32	24.10	24.39	25.09	25.22	23.16	25.11	24.16
32	127.08	126.64	127.13	126.92	127.17	126.23	127.09	126.16
33	42.05	41.27	41.53	41.92	41.93	40.24	41.92	40.99
34	72.90	71.82	71.93	72.75	72.75	70.52	72.69	71.66

35	51.16	50.70	51.00	51.11	51.20	49.40	51.05	50.44
36	61.25	60.38	60.59	61.22	61.20	59.08	61.10	60.13
37	55.82	55.23	55.46	55.71	55.75	53.95	55.70	54.99
38	150.92	150.61	151.19	150.84	150.95	149.61	150.83	150.03
39	152.11	151.95	152.54	152.15	152.18	150.47	152.04	151.61
40	117.75	117.05	117.27	117.73	117.73	116.09	117.62	116.77
41	127.18	126.47	126.63	127.03	127.11	125.28	126.94	126.19
42	139.75	139.28	139.33	139.63	139.93	138.02	139.62	139.14
43	122.36	121.76	122.22	122.39	122.30	120.58	122.21	121.46
44	37.64	36.75	36.93	37.51	37.63	35.55	37.50	36.64
45	55.86	55.42	56.05	55.99	55.88	53.87	55.79	55.36
46	169.61	169.08	169.38	169.46	169.55	167.49	169.40	168.91

[1] Numbers of carbon atoms correspond to the follow-up numbers of carbon atoms in all equilibrium geometries of strychnobaillonine (conf. 2) given above.

Table S11. ^{13}C NMR shielding constants and chemical shifts (in ppm) of molecules in set **2** calculated at the GIAO-CCSD(T)/pecS-2 level on equilibrium geometries obtained at the CCSD level with 6-311G(d,p) basis set.

**Molecule**	**№ of carbon atom** [1]	**Shielding constant, σ**	**Solvent correction to σ, Δ_{sol}**	**Total shielding, $\sigma + \Delta_{sol}$**	**Chemical shift from linear model, $\tilde{\delta}$**[2]	**Exp.** [3]
Acetaldehyde	1	5.60	-5.67	-0.07	196.19	199.97
	2	161.83	-0.63	161.20	34.92	30.99
Acetonitrile	1	190.91	0.26	191.17	4.95	1.91
	2	86.09	-5.04	81.05	115.07	116.33
Cyclopropane		197.16	0.57	197.73	-1.61	-3.15
DMAc	1	37.74	-2.36	35.38	160.74	170.66
	2	171.10	-0.56	170.54	25.58	21.58
	3	155.72	0.05	155.77	40.35	38.05
	4	161.53	0.27	161.80	34.32	35.20
Fluorobenzene	1	39.82	0.32	40.14	155.98	162.86

	2	84.22	0.05	84.27	111.85	115.32
	3	65.45	-0.48	64.97	131.15	129.96
	4	69.75	-0.74	69.01	127.11	123.98
Isoxazole	1	39.66	-1.54	38.12	158.00	157.64
	2	91.23	-1.15	90.08	106.04	103.47
	3	49.18	-1.78	47.40	148.72	149.02
Norbornad iene	1	51.46	-0.38	51.08	145.04	143.43
	2	147.96	-0.12	147.84	48.28	50.26
	3	121.66	-0.21	121.45	74.67	75.32
Oxetane	1	123.17	-0.85	122.32	73.80	72.55
	2	172.78	-0.07	172.71	23.41	22.35
Pyridine	1	45.69	0.25	45.94	150.18	149.74
	2	72.08	-1.11	70.97	125.15	123.78
	3	60.30	-1.71	58.59	137.53	136.09

[1] Numbers of atoms are depicted in Fig. 3 of main text.
[2] Linear model: $\tilde{\delta}(\sigma_i, \alpha) = -\sigma_i + \alpha$, with α =196.12
[3] For experiment, see the reference in the main text.

Table S12. ^{13}C NMR shielding constants and chemical shifts (in ppm) of molecules in set **2** calculated at the GIAO-CCSD(T)/pecS-2 level on equilibrium geometries obtained at the CCSD level with 6-31G(2d,2p) basis set.

**Molecule**	**№ of carbon atom** [1]	**Shielding constant, σ**	**Solvent correction to σ, Δ_{sol}**	**Total shielding, $\sigma + \Delta_{sol}$**	**Chemical shift from linear model, $\tilde{\delta}$**[2]	**Exp.** [3]
Acetaldehy de	1	5.32	-5.67	-0.35	197.17	199.97
	2	162.75	-0.63	162.12	34.70	30.99
Acetonitril e	1	191.72	0.26	191.98	4.84	1.91

	2	86.13	-5.04	81.09	115.73	116.33
Cyclopropane		197.91	0.57	198.48	-1.66	-3.15
DMAc	1	38.15	-2.36	35.79	161.03	170.66
	2	172.13	-0.56	171.57	25.25	21.58
	3	157.02	0.05	157.07	39.75	38.05
	4	162.81	0.27	163.08	33.74	35.20
Fluorobenzene	1	40.44	0.32	40.76	156.06	162.86
	2	85.12	0.05	85.17	111.65	115.32
	3	66.41	-0.48	65.93	130.89	129.96
	4	70.68	-0.74	69.94	126.88	123.98
Isoxazole	1	39.64	-1.54	38.10	158.72	157.64
	2	92.01	-1.15	90.86	105.96	103.47
	3	49.42	-1.78	47.64	149.18	149.02
Norbornadiene	1	51.93	-0.38	51.55	145.27	143.43
	2	148.67	-0.12	148.55	48.27	50.26
	3	122.84	-0.21	122.63	74.19	75.32
Oxetane	1	123.62	-0.85	122.77	74.05	72.55
	2	173.80	-0.07	173.73	23.09	22.35
Pyridine	1	46.50	0.25	46.75	150.07	149.74
	2	72.96	-1.11	71.85	124.97	123.78
	3	61.12	-1.71	59.41	137.41	136.09

[1] Numbers of atoms are depicted in Fig. 3 of main text.
[2] Linear model: $\tilde{\delta}(\sigma_i, \alpha) = -\sigma_i + \alpha$, with α = 196.82
[3] For experiment, see the reference in the main text.

Table S13. ^{13}C NMR shielding constants and chemical shifts (in ppm) of molecules in set **2** calculated at the GIAO-CCSD(T)/pecS-2 level on equilibrium geometries obtained at the CCSD level with 6-311G(3df,3pd) basis set.

Molecule	№ of carbon atom [1]	Shielding constant, σ	Solvent correction to σ, Δ_{sol}	Total shielding, $\sigma + \Delta_{sol}$	Chemical shift from linear model, $\tilde{\delta}$[2]	Exp. [3]
Acetaldehyde	1	7.23	-5.67	1.56	196.25	199.97
	2	163.35	-0.63	162.72	35.09	30.99
Acetonitrile	1	192.70	0.26	192.96	4.85	1.91
	2	87.28	-5.04	82.24	115.57	116.33
Cyclopropane		198.61	0.57	199.18	-1.37	-3.15
DMAc	1	39.11	-2.36	36.75	161.06	170.66
	2	172.25	-0.56	171.69	26.12	21.58
	3	157.16	0.05	157.21	40.60	38.05
	4	162.93	0.27	163.20	34.61	35.20
Fluorobenzene	1	41.88	0.32	42.20	155.61	162.86
	2	86.25	0.05	86.30	111.51	115.32
	3	67.20	-0.48	66.72	131.09	129.96
	4	72.03	-0.74	71.29	126.52	123.98
Isoxazole	1	41.80	-1.54	40.26	157.55	157.64
	2	93.18	-1.15	92.03	105.78	103.47
	3	51.43	-1.78	49.65	148.16	149.02
Norbornadiene	1	53.02	-0.38	52.64	145.17	143.43
	2	149.64	-0.12	149.52	48.29	50.26
	3	122.68	-0.21	122.47	75.34	75.32

Oxetane	1	124.70	-0.85	123.85	73.96	72.55
	2	174.42	-0.07	174.35	23.46	22.35
Pyridine	1	47.65	0.25	47.90	149.91	149.74
	2	73.93	-1.11	72.82	124.99	123.78
	3	62.31	-1.71	60.60	137.21	136.09

[1] Numbers of atoms are depicted in Fig. 3 of main text.
[2] Linear model: $\tilde{\delta}(\sigma_i, \alpha) = -\sigma_i + \alpha$, with α = 197.81
[3] For experiment, see the reference in the main text.

Table S14. ^{13}C NMR shielding constants and chemical shifts (in ppm) of molecules in set **2** calculated at the GIAO-CCSD(T)/pecS-2 level on equilibrium geometries obtained at the CCSD level with pecG-1 basis set.

**Molecule**	**№ of carbon atom** [1]	**Shielding constant, σ**	**Solvent correction to σ, Δ_{sol}**	**Total shielding, $\sigma + \Delta_{sol}$**	**Chemical shift from linear model, $\tilde{\delta}$** [2]	**Exp.** [3]
Acetaldehyde	1	6.48	-5.67	0.81	196.37	199.97
	2	162.91	-0.63	162.28	34.90	30.99
Acetonitrile	1	192.31	0.26	192.57	4.61	1.91
	2	86.31	-5.04	81.27	115.91	116.33
Cyclopropane		198.24	0.57	198.81	-1.63	-3.15
DMAc	1	38.85	-2.36	36.49	160.69	170.66
	2	172.40	-0.56	171.84	25.34	21.58
	3	157.27	0.05	157.32	39.86	38.05
	4	163.03	0.27	163.30	33.88	35.20
Fluorobenzene	1	40.77	0.32	41.09	156.09	162.86
	2	85.41	0.05	85.46	111.72	115.32

	3	66.66	-0.48	66.18	131.00	129.96
	4	71.03	-0.74	70.29	126.89	123.98
Isoxazole	1	40.44	-1.54	38.90	158.28	157.64
	2	92.38	-1.15	91.23	105.95	103.47
	3	50.13	-1.78	48.35	148.83	149.02
Norbornadiene	1	52.51	-0.38	52.13	145.05	143.43
	2	148.67	-0.12	148.55	48.63	50.26
	3	122.26	-0.21	122.05	75.13	75.32
Oxetane	1	124.16	-0.85	123.31	73.87	72.55
	2	173.84	-0.07	173.77	23.41	22.35
Pyridine	1	46.64	0.25	46.89	150.29	149.74
	2	73.34	-1.11	72.23	124.95	123.78
	3	61.56	-1.71	59.85	137.33	136.09

[1] Numbers of atoms are depicted in Fig. 3 of main text.
[2] Linear model: $\tilde{\delta}(\sigma_i, \alpha) = -\sigma_i + \alpha$, with α = 197.18
[3] For experiment, see the reference in the main text.

Table S15. ^{13}C NMR shielding constants and chemical shifts (in ppm) of molecules in set **2** calculated at the GIAO-CCSD(T)/pecS-2 level on equilibrium geometries obtained at the CCSD level with pecG-2 basis set.

**Molecule**	**№ of carbon atom [1]**	**Shielding constant, σ**	**Solvent correction to σ, Δ_{sol}**	**Total shielding, $\sigma + \Delta_{sol}$**	**Chemical shift from linear model, $\tilde{\delta}$[2]**	**Exp. [3]**
Acetaldehyde	1	7.24	-5.67	1.57	196.97	199.97
	2	164.15	-0.63	163.52	35.02	30.99
Acetonitrile	1	193.63	0.26	193.89	4.65	1.91

	2	87.65	-5.04	82.61	115.93	116.33
Cyclopropane		199.54	0.57	200.11	-1.57	-3.15
DMAc	1	39.40	-2.36	37.04	161.50	170.66
	2	173.00	-0.56	172.44	26.10	21.58
	3	157.88	0.05	157.93	40.61	38.05
	4	163.67	0.27	163.94	34.60	35.20
Fluorobenzene	1	42.29	0.32	42.61	155.93	162.86
	2	87.00	0.05	87.05	111.49	115.32
	3	68.08	-0.48	67.60	130.94	129.96
	4	72.73	-0.74	71.99	126.55	123.98
Isoxazole	1	42.46	-1.54	40.92	157.62	157.64
	2	94.04	-1.15	92.89	105.65	103.47
	3	52.36	-1.78	50.58	147.96	149.02
Norbornadiene	1	53.95	-0.38	53.57	144.97	143.43
	2	150.39	-0.12	150.27	48.27	50.26
	3	123.25	-0.21	123.04	75.50	75.32
Oxetane	1	125.45	-0.85	124.60	73.94	72.55
	2	175.70	-0.07	175.63	22.91	22.35
Pyridine	1	48.31	0.25	48.56	149.98	149.74
	2	74.96	-1.11	73.85	124.69	123.78
	3	63.14	-1.71	61.43	137.11	136.09

[1] Numbers of atoms are depicted in Fig. 3 of main text.
[2] Linear model: $\tilde{\delta}(\sigma_i, \alpha) = -\sigma_i + \alpha$, with α = 198.54
[3] For experiment, see the reference in the main text.
